# The 2025 *Lancet* Countdown Latin America report: moving from promises to equitable climate action for a prosperous future

**DOI:** 10.1016/j.lana.2025.101276

**Published:** 2025-10-29

**Authors:** Stella M. Hartinger, Yasna Palmeiro-Silva, Camila Llerena-Cayo, Rayana Santos Araujo Palharini, Christian García-Witulski, Maria Fernanda Salas, Nicolas Valdés-Ortega, Avriel Diaz, Luis E. Escobar, Carolina Gil Posse, Juliana Helo Sarmiento, Andres G. Lescano, Oscar Melo, Mónica Pinilla-Roncancio, David Rojas-Rueda, Tatiana Souza de Camargo, Bruno Takahashi, Luciana Blanco-Villafuerte, Nicolas Borchers-Arriagada, Marcia Chame, Francisco Chesini, Carole Dalin, Francisco Estrada, Marcelo Firpo Porto, Renata Gracie, Nelson Gouveia, Magali Hurtado-Díaz, Harry Kennard, Eliane Lima e Silva, Aline Martins de Carvalho, Zaray Miranda-Chacon, Nahid Mohajeri, Rômulo Paes-Sousa, Chrissie Pantoja, Tim Repke, Luiza Ribeiro Alves Cunha, Antonella Risso, Matilde Rusticucci, Alejandro Saez Reale, Raquel Santiago, Mauricio Santos-Vega, Enzo Sauma, Sol Saliva, Milena Sergeeva, Cecilia Sorensen, Juan D. Umaña, Armando Valdés-Velásquez, Maria Walawender, Juliana W. Rulli Villardi, Daniel Buss, Marina Romanello

**Affiliations:** a Centro Latino Americano de Excelencia en Cambio Climático y Salud, Universidad Peruana Cayetano Heredia, Lima, Peru; b Institute for Global Health, University College London, London, United Kingdom; c Departamento de Prevención de Riesgos y Medio Ambiente, Facultad de Ciencias de la Construcción y Ordenamiento Territorial, Universidad Tecnológica Metropolitana, Santiago, Chile; d Facultad de Ciencias Económicas, Pontificia Universidad Católica Argentina, Buenos Aires, Argentina; e Human Rights Centre Antonio Papisca, University of Padova, Padova, Italy; f Faculty of Medicine, Pontificia Universidad Católica de Chile, Santiago, Chile; g Global Council for Science and the Environment (GCSE), Washington DC, 20006, USA; h Department of Fish and Wildlife Conservation, Virginia Tech, Blacksburg, VA, USA; i Centro de Estudios de Historia de la Ciencia y de la Técnica José Babini, Escuela de Humanidades, Universidad Nacional de San Martín, San Martín, Argentina; j Facultad de Economía, Universidad de los Andes, Bogotá, Colombia; k Centro Interdisciplinario de Cambio Global, Pontificia Universidad Católica de Chile, Santiago, Chile; l School of Medicine and Sustainable Development Goals Centre for Latin America and the Caribbean, Universidad de los Andes, Bogotá, Colombia; m Department of Environmental and Radiological Health Sciences, Colorado State University, Fort Collins, USA; n Faculdade de Educação, Universidade Federal do Rio Grande do Sul, Porto Alegre, Brazil; o School of Journalism, Michigan State University, Michigan, USA; p Menzies Institute for Medical Research, University of Tasmania, Hobart, Tasmania, Australia; q Institutional Platform for Biodiversity and Wildlife Health and Wildlife Health Information System - Fundação Oswaldo Cruz (Fiocruz), Rio de Janeiro, RJ, Brazil; r Consejo Nacional de Investigaciones Científicas y Técnicas (CONICET) y Universidad Nacional de Avellaneda (UNDAV) Argentina, Buenos Aires, Argentina; s Institute for Sustainable Resources, University College London, London, UK; t Instituto de Ciencias de la Atmósfera y Cambio Climático y Programa de Investigación en Cambio Climático, Universidad Nacional Autónoma de Mexico, DF, Mexico; u Fundação Oswaldo Cruz (Fiocruz), Rio de Janeiro, RJ, Brazil; v Instituto de Comunicação e Informação Científica e Tecnológica em Saúde - ICICT/Fiocruz Rio de Janeiro, Rio de Janeiro, Brazil; w Departamento de Medicina Preventiva, Faculdade de Medicina, Universidade de São Paulo, São Paulo, Brazil; x Environmental Health Department, National Institute of Public Health (Mexico), Cuernavaca, Mexico; y University of Texas at Austin, Austin, TX, USA; z Departamento de Geografia, Universidade de Brasília, Brasilia, Brazil; aa Department of Nutrition, Faculty of Public Health, University of São Paulo (USP), São Paulo, 01246-904, Brazil; ab Escuela de Medicina, Universidad de Costa Rica, San Jose, Costa Rica; ac Institute of Environmental Design and Engineering, Bartlett School of Environment, Energy and Resources, University College London, London, UK; ad Instituto René Rachou, Fiocruz Minas, Fundação Oswaldo Cruz, Belo Horizonte, MG, Brazil; ae Nicholas School of the Environment and Sanford School of Policy, Duke University, Durham, NC, USA; af Potsdam Institute for Climate Impact Research, Potsdam, Germany; ag Vice-presidência de Ambiente, Atenção e Promoção da Saúde, Fundação Oswaldo Cruz (Fiocruz), Rio de Janeiro, RJ, Brazil; ah Consejo Nacional de Investigaciones Científicas y Técnicas (CONICET) y Universidad de Buenos Aires (UBA) Argentina, Buenos Aires, Argentina; ai Global Heat Health Information Network, World Meteorological Organization, Geneva, Switzerland; aj Faculdade de Nutrição, Universidade Federal de Goiás, Goiânia, Goiás, Brazil; ak Grupo de Biología Matemática y Computacional (BIOMAC), Universidad de los Andes, Bogotá, Colombia; al Industrial and Systems Engineering Department, Pontificia Universidad Católica de Chile, Santiago, Chile; am Mailman School of Public Health, Columbia University, New York, NY, United States; an Grupo de Investigación en Sistemas Socio-Ecológicos, Laboratorios de Investigación y Desarrollo, Universidad Peruana Cayetano Heredia, Lima, Perú; ao Pan American Health Organization, Washington DC, USA

**Keywords:** Climate change and health, Latin America, Adaptation, Mitigation and health co-benefits, Economic and finance, Public engagement

## Executive summary

Globally, 2024 was the warmest year on record, with the average near-surface temperature reaching 1.55 °C above pre-industrial levels. This record is part of a continued warming trend, with temperatures staying above the 1.5 °C threshold for nearly two years. This warming, largely driven by economic activities, is leading to concurrent hazards across Latin America, including heatwaves, wildfires, and floods, which are devastating communities, disrupting livelihoods and leading to a wide range of health consequences for the people of the region.

Amidst this escalating threat, human health has become a central focus in international climate diplomacy, with the COP28 UAE Framework including a specific health target and operationalising the Global Goal on Adaptation. While these steps are important, significant challenges remain. The world remains off-track to meet its climate goals, despite a decade since the Paris Agreement. The continued reliance on fossil fuels and the failure of many countries to meet emissions targets and climate finance commitments are some of the major obstacles undermining efforts to protect human health and progress to a thriving future.

The 2025 *Lancet* Countdown Latin America report, a collaboration of 25 regional academic institutions and UN agencies, tracks 41 indicators across 17 Latin American countries. It provides clear evidence on the escalating impacts of climate change on human health. For this iteration, several methodological improvements were implemented, including the refinement of existing indicators and the incorporation of six new ones. Additionally, efforts were made to integrate a sub-national perspective wherever data allows, recognising that climate impacts and the effectiveness of responses vary significantly among and within countries. The following section summarises the main findings, structured around three key messages that reflect the central themes of the report.

## Key messages 1: Addressing climate change is essential to protecting human health

### Human health will continue to deteriorate in the future due to the compounding effects of climate change

The 2025 LCLA report demonstrates the escalating health impacts of anthropogenic climate change across Latin America, noting alarming trends of intensifying climate hazards that put individuals and society at risk. Individuals are increasingly exposed to extreme heat, extreme events and an elevated risk of infectious disease transmission.

The mean ambient temperature in Latin America has followed a persistent warming trend since 2000. Average annual exposure rose from 23.3 °C in 2001–2010 to 23.8 °C in 2015–2024, reaching a record-high 24.3 °C in 2024. These increases are not homogeneous; we observe higher temperature exposures in countries such as Bolivia (+2 °C), Venezuela (+1.7 °C), Mexico (+1.6 °C), Paraguay (+1.5 °C), Ecuador (+1.4 °C), Guatemala (+1.3 °C), Brazil (+1.2 °C) and Colombia (+1 °C) and even higher temperature extremes within these countries. The health impacts of this rise are profound. Infants experienced a staggering 450% increase in heatwave exposure days, while adults over 65 faced an even more concerning 1000% increase in exposure compared to the 1981–2000 baseline. For older adults, these relative increases reach catastrophic levels in Venezuela (5116%) and Colombia (5910%). Overall, heat-related mortality increased by 103%, resulting in approximately 13,000 deaths annually. This incurred an average annual monetised cost of US$855 million during 2015–2024, representing a significant 229% increase compared to the previous decade. Furthermore, heat-related labour losses in 2024 totalled a substantial US$52 billion, a 12.6% rise from 2023, disproportionately impacting agriculture and construction.

The increase in frequency and intensity of extreme events such as droughts and wildfires have affected most countries in the region. The proportion of Latin American land experiencing meteorological drought conditions (one month) saw a significant rise of 275%, expanding from 15.8% in 1981–1990 to 59.1% in 2015–2024 with Brazil, Bolivia, and Mexico showing the most significant impacts. This escalation trend holds true for more prolonged droughts as well: land affected by agricultural droughts (three months) expanded from 6.3% to 40.7%, while land facing persistent hydrological droughts (six months), expanded from 2.1% to 20.8% across Latin America in the same period. This likely contributed to the extreme wildfire risk observed in 2024, causing a 10% total increase across the region (in 9 out of 17 countries). The most substantial increases were seen in Chile (30.5 days, 105% rise), Mexico (17.6 days, 28.5% rise), and Bolivia (16.7 days, 82.6% rise).

These extreme climate events led to significant out-of-pocket economic losses totalling nearly 19.2 billion in 2024 (0.3% of the region's GDP), a sum made more severe by the fact that less than 5% of them are insured. Brazil accounted for two-thirds of the regional total economic loss, followed by Mexico and Chile. Relative to national output, Chile and Brazil shared the highest proportional losses (both approximately 0.63% of GDP), followed by Mexico (0.14%), Panama (0.13%), Ecuador (0.08%) and Peru (0.07%).

It is essential to note that many of these extreme climate events occur simultaneously and consecutively (e.g., prolonged drought, intense heatwaves, and wildfires). Addressing climate change is therefore essential to protecting human health, as these overlapping events create cascading risks and economic shocks that slow recovery and undermine resilience.

## Key message 2: Adaptation is no longer optional, it is an essential and non-negotiable necessity

### A multilevel strategy must be prioritised to reduce climate risks, increase resilience, and address existing socioeconomic inequalities

Action at the highest level—governance and national policy—remains inadequate, indicating a systemic failure to prioritise health resilience. The LCLA 2025 Report demonstrates that crucial planning efforts are limited. Fewer than half of countries in the region (41.2%) have publicly reported completing a Vulnerability and Adaptation Assessment since 2020, and only nine countries (53%) have developed a Health National Adaptation Plan. Furthermore, the integration of health into broader Nationally Determined Contributions (NDCs) remains insufficient.

This lack of political drive extends to international forums and funding. While NDCs increasingly incorporate equity concerns; however, their visibility at the United Nations General Assembly (UNGA) has dropped significantly. Mentions of health in UNGA addresses by Latin American countries fell from a peak of 10 in 2010 to only three countries (Bolivia, Brazil, and Chile) in 2024. Undermining the regional imperative to highlight the interlinkage between climate change and health, vulnerable populations and environmental justice on the global stage.

Bridging this lack of prioritisation demands targeted investment in health adaptation planning and implementation. Bilateral donors committed US$197 million to health adaptation projects in 2024, but 68% was allocated to Brazil alone. Furthermore, of the US$3.4 billion approved by the Green Climate Fund for projects incorporating a health dimension since 2017, only US$77.7 million (2.3%) targets direct health adaptation.

To fortify the Health System, robust data and strong inter-institutional collaboration is vital. While 10 of 17 World Meteorological Organization members in Latin America report providing climate services for health, these services remain focused on immediate data and monitoring, with less emphasis on long-term climate projections—a critical component needed for strategic planning. Strong institutional preparedness is a key cornerstone for resilient health systems and it works. Countries with climate-informed Health Early Warning Systems (HEWS) saw a dramatic 92.5% decline in mortality from floods and storms, showcasing the life-saving impact of effective systems. Unfortunately, overall self-reported emergency preparedness has declined since 2022—a significant concern for dengue-vulnerable nations, like Bolivia, Brazil, and Peru.

At the same time, low human resource capacity and underfunded knowledge generation further weaken the region's ability to respond. Only 17% of surveyed public health students are receiving training on climate change, hindering the workforce's ability to manage future risks. Knowledge generation is also a bottleneck. Despite increasing scientific publications on climate and health since 2015, Latin America still accounts for only 5.5% of global output. Furthermore, the research often neglects crucial equity-related themes, which is mirrored in research funding for projects explicitly addressing climate change and health nexus. Without this locally relevant evidence, it is difficult to push regional needs into international agendas.

Implementation of adaptation strategies at the local and community scale continues to be limited and lacking in strategic focus. Poor urban planning is evident, as all Latin American cities with more than 500,000 inhabitants were classified as having low or exceptionally low greenness levels, missing a vital opportunity to build urban health resilience through natural infrastructure. Of the surveyed local administrative units, only 54 recognised specific health issues driven by climate hazards. The majority focusing on floods, storms, and heavy precipitation events, followed by drought-related hazards and extreme temperatures.

Conversely, merging climate and fundamental human development agendas -emphasising compliance with the Sustainable Development Goals (SDGs)- creates an essential foundation for resilience, demonstrated by the fact that improvements in basic water and sanitation services drove a nearly 60% reduction in the Mosquito Risk Index since 2000.

At individual level, protection is often contingent on economic status. The rising use of Air Conditioning (AC) demonstrates a clear mitigation-adaptation trade-off, as its high energy use exacerbates GHG emissions when uncoupled from clean energy sources. Its low adoption rate (27% of households) underscores significant inequality in who is protected during extreme heat events. This is mirrored in the disparity of indoor air pollution, which is far higher in rural, often lower-income, households.

Media and social engagement currently reflect the political turmoil we are experiencing. We observe a mixed signal, with a concerning risk of misinformation and an overall decline in reporting on the climate-health nexus. While coverage related to health is modestly increasing in both news media and social platforms (where engagement has grown substantially since 2017), overall climate news coverage declined in 2024, likely due to a shift toward more politicised issues.

## Key message 3: Effective and integrated climate and health governance must be defined by tangible progress

### Governments need to make decisions leading to action, accountability and impact regarding climate change and health

Throughout Latin America, funding, support for a Just Energy Transition, and collective climate action remain critically low. A key factor driving this stagnation is the region's continued fossil fuel dependency. Latin American countries exhibit a net-negative carbon price, reflecting a substantial net fossil fuel subsidy of US$38.6 billion- a sum that outweighs carbon pricing revenues by nearly fifty to one.

Building a resilient future requires fundamentally transforming our energy systems and shifting away from our dependency on fossil fuels, which demands greater mitigation commitments explicitly outlined in each country's NDCs. This transformation includes household energy use and the adoption of sustainable, healthy transportation as these sectors are vital due to their direct relation to GHG emissions and health implications. Furthermore, the way we produce and consume food, and how we manage our forests, also critically impact our ability to thrive throughout this crisis.

While replacing coal and other fossil fuels with renewable energy is fundamental for a healthy and sustainable future, the 2025 LCLA report indicates that progress in the electricity generation mix is diverse across countries and sectors. Regionally speaking, Latin America's overall electricity generation mix experienced a net decrease in its low-carbon share, falling from 67.6% to 58.9%, and renewable source generation (solar and wind) showed significant growth, increasing from 2.7% to 11.8% and overtaking coal's share in 2014. Simultaneously, coal-fired electricity's share also nearly doubled, rising from 2.6% to 5.2%, with a notable resurgence following the pandemic. Fossil fuels still overwhelmingly dominate Latin America's road transport, powering 96.7% of the sector.

Failure to tackle these emissions sources sustains high levels of PM2.5 air pollution exposure. The national Latin America average indoor PM_2.5_ concentration from polluting solid fuels for cooking and heating was estimated at 245 μg/m^3^ in 2022, but concentrations are more than doubled in rural (314 μg/m^3^) compared to urban households (145 μg/m^3^), underscoring significant social-economic inequalities. This disparity is driven by a lack of access to cleaner fuels and continued use of biomass fuels (31% rural vs. 5% urban). Furthermore, 79% of Latin American households now use liquified petroleum gas (LPG) for cooking, underscoring a missed opportunity to transition directly to clean renewable sources and highlighting future challenges for a region still heavily dependent on fossil fuels.

Fossil fuel-related PM_2.5_ (coal and gas) from ambient sources is responsible for 360,000 premature deaths among working-age individuals, imposing substantial social and economic burdens. Premature deaths attributed to PM_2.5_ from biomass were estimated at 140,000 in 2018–2022, representing a concerning increase of 17,000 deaths compared to 2007–2011. The monetisation costs of Years Life Lost attributable to PM_2.5_ in 11 Latin American countries amounted to US$160 billion. This figure is equivalent to 2.8% of their aggregate GDP, corresponding to the average annual income of about 15.8 million people in the region.

Other regional emission sources—such as agricultural GHG emissions and tree cover loss driven by commodity production and deforestation—have already caused the eastern regions of the Amazon to become a net carbon source. Mitigation strategies, such as regenerative agriculture, cattle ranching and agroecology initiatives, offer solutions by avoiding expansion, restoring land, and conserving biodiversity essential to maintain human health. Additionally, the transformation of the food system is a key mitigation strategy that yields immediate health co-benefits and environmental advantages, ensuring equitable access to sustainably produced food, boosting biodiversity, and strengthening food security.

Latin America does not have the luxury to wait for a global political will and must push ahead with national actions that protect both people and nature. It is time for countries to deliver on their Nationally Determined Contributions and National Adaptation Plans through more effective governance—defined by action, accountability, and measurable health impacts—rather than just hopeful promises. This is particularly crucial given the current geopolitical tensions and shifting donor priorities, which raise serious concerns that promised resources may not materialise at the required pace or scale to safeguard public health.

As COP30 in Belém do Pará rapidly approaches, the region has a unique opportunity to champion health-prioritised and equitable climate adaptation initiatives and push for rapid, health-co-beneficial mitigation strategies that are fair to all.

## Introduction

Globally, 2024 was confirmed as the warmest year in the 175-year observational record, with the global mean near-surface temperature reaching 1.55 (±0.13) °C above the 1850–1900 pre-industrial average.^1^ This warming trend has been consistent, with global temperatures remaining above 1.5 °C for nearly two years by April 2025.^2^ Recent analysis attributes 1.36 °C of this warming (88%) to human activity, with the current rate of warming at 0.27 °C per decade.^3^ In Latin America, climate change has led to concurrent hazards including extreme heatwaves, devastating wildfires, prolonged and severe droughts, and heavy rainfall events—affecting lives, livelihoods, food supply chains, and a wide range of social and ecological systems.^4^ For instance, in 2024, widespread drought across Amazonia and the Pantanal brought rainfall 30–40% below normal, leading to record low river levels.^4^ Meanwhile, unprecedented floods in Rio Grande do Sul, Brazil, became the nation's most severe climate-related disaster in 2024.^4^ Compounding these crises, glacier retreat has accelerated alarmingly. With the disappearance of the Humboldt glacier in 2024, Venezuela became the second country globally and the first country in the Americas to lose all of its glaciers.^1^^,^^5^ These hazards and climate change-driven ecological shifts almost certainly contributed to important negative health outcomes, including record-breaking epidemic waves of dengue fever that swept across the region in 2023/2024.^6^

Amidst this escalating threat, health has gained unprecedented prominence in international climate diplomacy. The UAE Framework for Global Climate Resilience, adopted at the 28th Conference of the Parties (COP28) includes a specific thematic target focused on health^7^; the Global Goal on Adaptation (GGA) has been operationalised; and the UAE-Belém work programme is developing up to 100 adaptation indicators, including those vital for tracking health.^8^ While these steps forward in monitoring human health in climate change are positive, they are shadowed by persistent obstacles. For example, the continued use of fossil fuels obstructs the ability to achieve international agreements to reduce dependency on them.^9^ As a consequence, it is now a decade since the Paris Agreement, yet the world is not on track to meet its goals, with many countries failing to meet emissions targets and climate finance commitments. A clear, robust, and equitably structured financing framework, appropriately scaled to the true magnitude of mitigation and adaptation needs, remains elusive. While the New Collective Quantified Goal was agreed at COP29 by consensus of all parties,^10^ and its USD 300 billion annual target by 2035 represents an important potential down payment for climate action, its full realisation and ultimate adequacy are yet to be seen. This is compounded by the yet unmet $1.3 trillion annual commitment from all actors (public and private) and the optimal operationalisation of the Loss and Damage Fund.

The 2025 *Lancet* Countdown Latin America report is being released at a crucial moment for the region and marks a new and more ambitious five-year commitment (2024–2029) within the *Lancet* Countdown on Health and Climate Change collaboration, supported by the Wellcome Trust. As a result of strong coordinated efforts among 25 regional academic institutions and United Nations agencies, and 51 researchers from 13 countries, this report aims to be of service to a diverse range of actors — from government decision-makers and public health professionals to journalists, civil society organisations, and the wider community. Through 41 indicators (Panel 1), it provides clear evidence on the evolving impacts of climate change on the health and wellbeing of millions of Latin Americans. Furthermore, it tracks progress (or lack thereof) in adaptation, mitigation, climate finance, and political and public engagement with this critical nexus, providing complementary analyses of such a complex problem.

The 2025 report covers 17 Latin American countries: Argentina, Bolivia, Brazil, Chile, Colombia, Costa Rica, Ecuador, El Salvador, Guatemala, Honduras, Mexico, Nicaragua, Panama, Paraguay, Peru, Uruguay and Venezuela. For the 2025 report, several improvements were made: the methodologies of two indicators were improved (i.e., indicator 1.2.1 and 4.1.1), one new indicator was developed specifically for this report (i.e., indicator 5.4.2), and five additional indicators were incorporated from the global Lancet Countdown reporting (i.e., indicators 1.2.2; 2.3.2; 2.2.2; 2.2.5 and 3.5). Additionally, efforts have been made to integrate a sub-national perspective wherever data allow, recognising that climate impacts and the effectiveness of responses vary significantly among and across countries.

A significant development for the 2025 report is the partnership with the Oswaldo Cruz Foundation (Fiocruz), a leading public health institution in Brazil and globally. This collaboration has enabled the incorporation of dedicated analytical panels offering a detailed examination of the climate and health landscape in Brazil. Given Brazil's role as host of the forthcoming 30th Conference of Parties (COP30) to the United Nations Framework Convention on Climate Change in Belém, this in-depth analysis is particularly timely and vital.

Overall, the 2025 report is, first and foremost, for the people of Latin America and a direct contribution to the COP30 presidency's spirit: the “Mutirão Global” (originally from Tupi-Guarani language, meaning “collective efforts”).^11^ It lays bare the profound challenges that a changing climate imposes on people's health, wellbeing, and livelihoods, while is also a call to moving from promises to equitable collective mobilisation for ambitious climate action that safeguards health and builds a prosperous and healthy future for all.

Panel 1 **The indicators of the 2025 report of the Lancet Countdown Latin America on health and climate change.**

**Section 1: Health hazards, exposures, and impacts**

1.1 Health and heat
  1.1.1 Exposure to warming
  1.1.2 Exposure of vulnerable populations to heatwaves
  1.1.3 Heat and physical activity
  1.1.4 Heat-related mortality
1.2 Health and extreme weather-related events.
  1.2.1 Wildfires
  1.2.2 Droughts
1.3 Climate suitability for infectious disease transmission.
  1.3.1 Dengue
  1.3.2 Vibriosis

**Section 2: adaptation, planning, and resilience for health**

2.1 Assessment and planning of health adaptation.
  2.1.1 National assessments of climate change impacts, vulnerability, and adaptation for health
  2.1.2 National adaptation plans for health
  2.1.3 City-level climate change risk assessments
2.2 Enabling conditions, adaptation delivery, and implementation.
  2.2.1 Climate information for health
  2.2.2 Benefits and harms of air conditioning
  2.2.3 Urban greenspace
  2.2.4 Detection, preparedness, and response to health emergencies
  2.2.5 Climate and health education and training
2.3 Vulnerabilities, health risks, and resilience to climate change.
  2.3.1 Vulnerability and risk to mosquito-borne diseases
  2.3.2 Lethality of extreme weather events

**Section 3: mitigation actions and health co-benefits**

3.1 Energy use, energy generation, and health.
  3.1.1 Energy systems and health
  3.1.2 Household energy use
  3.1.3 Sustainable and healthy transport
3.2 Air quality and health co-benefits.
  3.2.1 Premature mortality from ambient air pollution
  3.2.2 Household air pollution
3.3 Food, agriculture, and health co-benefits.
  3.3.1 Emissions from agricultural production and consumption.
  3.3.2 Diet and health co-benefits.
3.4 Tree cover loss and health.
3.5 Healthcare sector emissions.

**Section 4: economics and finance**

4.1 Economic impacts of climate change and its mitigation.
  4.1.1 Economic losses due to weather-related extreme events.
  4.1.2 Costs of heat-related mortality.
  4.1.3 Loss of earnings from heat-related reduction in labour capacity.
  4.1.4 Costs of the health impacts of air pollution.
4.2 The transition to net zero-carbon, health-supporting economies.
  4.2.1 Country preparedness for the transition to net zero.
4.3 Economics of the transition to zero-carbon economies.
  4.3.1 Net value of fossil fuel subsidies and carbon prices.
  4.3.4 Health adaptation finance flows and disclosed needs.

**Section 5: public and political engagement with health and climate change**

5.1 Media engagement with health and climate change.
5.2 Social media engagement with health and climate change.
5.3 Scientific articles on health and climate change.
5.4 Political engagement with health and climate change.
  5.4.1 Government commitment with health and climate change in UNGA & NDCs.
  5.4.2 Funding for science on health and climate in Latin America.
5.5 Corporate sector engagement with health and climate change.

## Section 1: health hazards, exposures, and impacts

The health impacts of climate change are the product of complex interactions between evolving climate hazards, population exposure, and underlying vulnerabilities of the population that is affected. During 2023 and 2024, Latin American countries witnessed an alarming intensification and confluence of climate hazards,^4^ highlighting the region's exposure to climate change and leading to a wide range of health consequences for the people of the region (Panel 2).

Tracking the evolving climate hazards and their relationship with human health is key for informing preparedness measures and responses in the short- and long-term. To support decision-making and further climate actions, this section analyses eight indicators covering heat and health, wildfires, droughts (new to the report), and climate suitability for dengue and vibriosis (Panel 1). Detailed methods and additional analyses for each indicator are presented in the Appendix.

### 1.1 Health and heat

#### Indicator 1.1.1 Exposure to warming

*Headline finding: in 2024, Latin American populations were exposed to an average increase in mean ambient temperature of 1 °C compared to a 2001–2010 baseline, rising from 23.3 °C to a* *record-high* *24.3 °C.*

Climate change has increased global temperatures in Latin America, impacting both human and ecosystem health.^12^ This indicator calculates annual population-weighted ambient temperature (to represent population exposure to warming) and temperature anomalies, using 2000–2024 2 m temperature data from ERA5-Land^13^ and the Global Human Settlement Layer.^14^

Overall, mean ambient temperature has followed a persistent warming trend since 2000. During the decade of 2001–2010, Latin Americans were exposed, on average, to an annual mean temperature of 23.3 °C. This increased to 23.8 °C in the decade 2015–2024, with a record-high 24.3 °C in 2024, having diverse impacts at local levels. The ten countries with the highest net increases in annual mean temperature between 2001 and 2010 and 2024 were Bolivia (+2 °C), Venezuela (+1.7 °C), Mexico (+1.6 °C), Paraguay (+1.5 °C), Ecuador (+1.4 °C), Guatemala (+1.3 °C), Brazil (+1.2 °C), Colombia (+1 °C), Honduras (+1 °C), and Peru (+1 °C).

#### Indicator 1.1.2 Exposure of vulnerable populations to heatwaves

*Headline finding: comparing 2015–2024 to 1981–2000, vulnerable Latin American populations experienced a substantial increase in heatwave exposure: infants (<1 yr) recorded an increase exceeding 450% in heatwave* *person-days,* *and older adults (>65 yrs) an increase exceeding 1000%, respectively.*

Heatwaves, defined as prolonged periods of unusual heat, pose a significant threat to health and wellbeing,^15^ from discomfort to death. Risk increases with age, co-morbidities, and certain treatments and is exacerbated by inadequate infrastructure, such as limited green spaces, high impervious surface cover, and other social determinants of health.^15^

This indicator tracks exposure of vulnerable populations (infants younger than 1 year old and adults above 65 years old) to heatwaves in Latin American countries, drawing from the 2025 global Lancet Countdown report.

Analysis of heatwave exposure, measured in person-days –a metric totalling the number of days each person experienced heatwave conditions– across Latin American countries indicates a pronounced increase when comparing the 2015–2024 period against the 1981–2000 baseline. Infants younger than 1 year and people older than 65 years experienced an increase in total heatwave person-days by more than 450% and 1000%, respectively. Important differences are seen across countries ranging from 96% in Chile and 132% in Uruguay to 1775% in Guatemala and nearly 1900% in Venezuela. For older adults (>65 years), the relative increases are exceptionally high, surpassing 1000% in 12 countries, and reaching over 5000% in countries such as Venezuela (5116%) and Colombia (5910%).

#### Indicator 1.1.3 Heat and physical activity

*Headline finding: in 2015–* *2024, annual individual exposure to at least moderate heat stress risk increased 298 (+29%) additional hours while walking and 289 (+24%) additional hours while running, compared to 1991–* *2000.*

Engaging in physical activity under high ambient temperatures elevates the risk of heat stress, potentially impacting health and reducing the capacity for safe exercise or outdoor work.^16^ This indicator, drawing from the 2025 global Lancet Countdown report, tracks the annual hours when walking (Class 1) and running (Class 3) outdoors posed at least moderate heat stress risk.

**Fig. 1:**
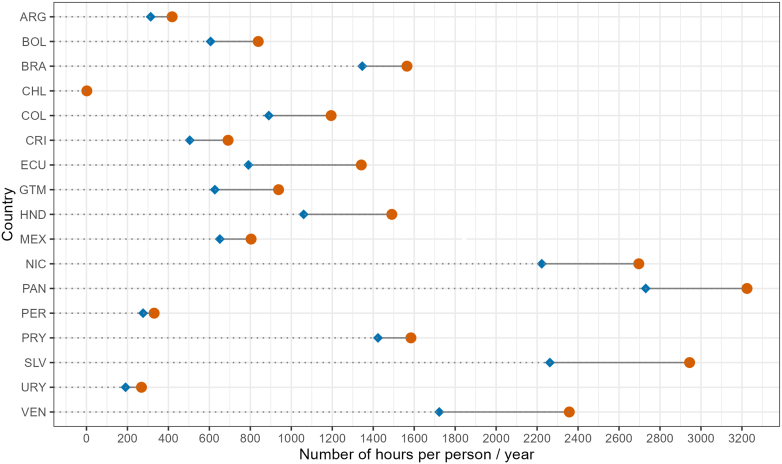
Average annual hours per person that walking activity entailed at least a moderate heat stress risk in 1991–2000 (blue diamond) and in 2015–2024 (orange circle) in Latin American countries.

An analysis of trends in Latin America comparing 2015–2024 to 1991–2000 reveals a widespread increase in the number of hours during which walking and running posed a heat stress risk. Overall, there was an average increase across Latin American countries of 298 (+29%) and 289 (+24%) additional hours per person per year during which ambient heat posed at least moderate risk of heat stress while walking and running, respectively (Fig. 1). Countries with the largest net increases are El Salvador (+682 h for walking and +549 h for running), Venezuela (+634 h for walking and +588 h for running), and Ecuador (+551 h for walking and +531 h for running). Chile had the lowest net increase but largest relative increase, adding 1.5 h of heat stress risk while walking (+548%) and 7 h while running (+276%). These figures indicate a concerning trend that increasingly limits opportunities for safe physical activity across the region.

#### Indicator 1.1.4 Heat-related mortality

*Headline finding: In 2012–2021,* *heat-related* *mortality reached an estimated average of 2.2 deaths per 100,000 people annually in Latin America, up 103% from 1990 to 1999.*

Extreme heat poses serious, and potentially fatal, health risks to vulnerable individuals.^15^ This indicator draws from the 2025 global Lancet Countdown report and estimates heat-related deaths for all age groups.

Overall, there has been a sustained increase in heat-related mortality in Latin America from 1990 to 2021, with a particularly sharp and noticeable increase in the number of deaths from 2008 onward. In 2012–2021, heat-related mortality reached an estimated average of 2.2 deaths per 100,000 people annually (∼13,000 heat-related deaths annually), an increase of 103% compared to 1990–1999 (1.1 deaths per 100,000 people annually; ∼5000 heat-related deaths annually).

### 1.2 Health and extreme weather-related events

#### Indicator 1.2.1 Wildfires

*Headline finding: during 2020–2024, Latin Americans experienced an increase in days exposed to very or extremely high fire danger (+26.4%), in total* *person-days* *of wildfire exposure (+10%), and in* *wildfire-related* *PM*_*2.5*_ *levels (+6.7%), compared to 2003–2007.*

While wildfires are a natural part of Earth's ecosystems, their increasing intensity and frequency, exacerbated by climate change,^17^ pose significant threats to human health and wellbeing in Latin America. Impacts range from direct physical harm to severe health issues stemming from worsened air quality due to smoke.^18^ The severe wildfires of 2024, particularly in the Amazon^19^ underscore the urgent need for enhanced preparedness through improved emergency response and early warning systems.

This indicator combines data by country and sub-national reanalyses and presents three sub indicators: i) number of days people were exposed to very high or extremely high fire danger risk, ii) number of person-days people were exposed to active wildfires, and iii) exposure to wildfire-PM_2.5_ (fine particles ≤2.5 μm).^20^

**Fig. 2:**
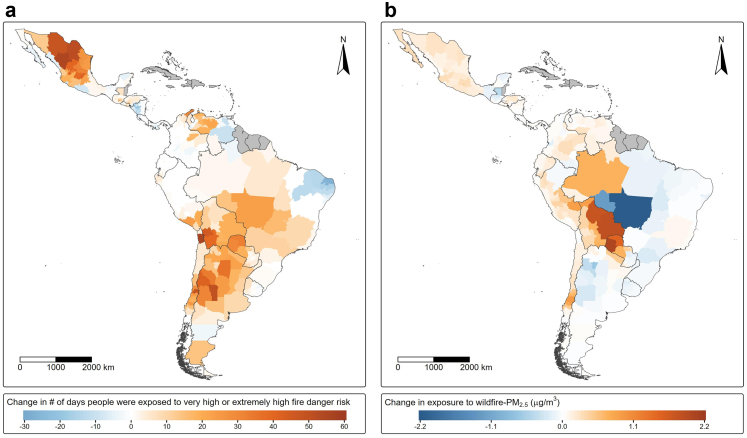
a) Change in annual number of days people were exposed to very high or extremely high fire danger risk; and b) Change in annual exposure to wildfire-related PM_2.5_ (μg/m^3^) in Latin America between 2020–2024 (comparison period) and 2003–2007 (baseline), by sub-national level.

Exposure to very high or extremely high fire danger increased an average of 8.2 days (26.4%) in 2020–2024, compared to 2003–2007. The number of days increased by at least 1 day in 13 out of 17 countries, with almost two thirds (65%) of the Latin American population suffering an increase during this period. The highest increases were seen in Chile (30.5 days, +105%), Mexico (17.6 days, +28.5%), and Bolivia (16.7 days, +82.6%). On the other hand, Costa Rica and Ecuador experienced an increase of less than 1 day, while Panama (−33.4%) and Nicaragua (−20.7%) experienced a decrease. At a sub-national level, the highest increase was observed in northern Chile (region of Tarapacá) and northern Mexico (states of Durango and Coahuila) with more than 50 additional days per year (Fig. 2). Average wildfire exposure (total person-days exposed to wildfires) also increased for the whole region (+10%), and in 9 out of 17 countries. The highest increase was seen in Venezuela (+200%) and Honduras (+27%). Wildfire-related PM_2.5_ also increased, from a population-weighted mean of 0.73 μg/m^3^–0.77 μg/m^3^ (+6.7%) between 2003–2007 and 2020–2024. Wildfire-PM_2.5_ increased in 13 out of 17 countries, with the highest increase in Bolivia (1.1 μg/m^3^, +53.7%). At a sub-national level, increases above 1.5 μg/m^3^ were observed in Bolivia (departments of Beni and Santa Cruz) and Paraguay (departments of Boquerón and Alto Paraguay) (Fig. 2).

#### Indicator 1.2.2 Droughts

*Headline finding: between 2015 and 2024, an average of 20.8% of land in Latin America experienced at least 6 months of extreme drought annually, which corresponds to 18.6% more land under extreme drought annually compared to 1981–* *1990.*

Assessing drought over different time periods, such as one, three, and six months, reveals its evolving nature and sector-specific impacts. A one-month precipitation deficit typically indicates meteorological dryness, primarily affecting topsoil moisture, stressing shallow-rooted agriculture, and increasing fire risk. Drought persisting three months, often signifies developing agricultural and hydrological stress, leading to reduced streamflow and broader crop damage. Droughts lasting six-month or longer typically lead to severe and prolonged water scarcity. This results in depleted reservoirs and groundwater, extensive agricultural failure, significant ecological disruption, and considerable socio-economic consequences. These can include migration, loss of livelihoods, and increased pressure on urban services.^21^^,^^22^

This indicator, new to the *Lancet* Countdown Latin America report, draws on the 2025 global Lancet Countdown report, and uses the Standardised Precipitation Evapotranspiration Index (SPEI) to monitor extreme drought intensity and duration across all land areas.^23^ An extreme drought is defined as a prolonged period of moisture deficit, where the SPEI value is lower than −2.00.

There has been a significant increase in the area affected by extreme droughts in Latin America since 1981, whether for at least 1, 3, or 6 months. For extreme droughts lasting at least 1 month, the annual average land area affected increased from 15.8% in 1981–1990 to 59.1% in 2015–2024. This is a net increase of 43.4 percentage-points and a relative rise of 275%. Brazilian states of Rondonia and Mato Grosso recorded the largest increases in the area affected by extreme droughts lasting more than one month, a relative percentage increase exceeding 900% considering the base period (1981–1990) and the recent period (2015–2024), and departments of Santa Cruz in Bolivia and Chihuahua in Mexico a percentage increase exceeding 500%.

**Fig. 3:**
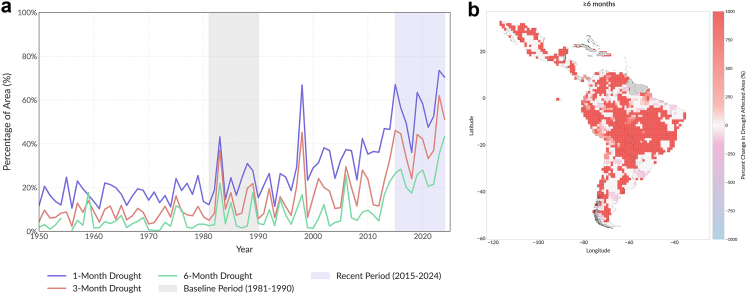
a) Temporal series of annual average percentage of land area affected by at least 1 month (blue), 3 months (red) or 6 months (green) of extreme drought per year in Latin America from 1950 to 2024. b) Map of percent increase in land area affected by drought ≥6 month, between 1981–1990 and 2015–2024.

The trend sustains for more prolonged drought. The land area experiencing at least 3 months of extreme drought annually expanded from 6.3% to 40.7% between these periods, representing an increase of 34.4 percentage-points and a relative increase of 543%. The department of Santa Cruz and Conchabamba in Bolivia and the Brazilian states of Rondonia, Tocantis, Goiás and Mato Grosso recorded the largest increases in the area affected by extreme droughts lasting more than three months, a relative percentage increase exceeding 3000% considering the base period and the recent period. For the most persistent extreme droughts, those lasting at least 6 months annually, the affected land area in Latin America grew from 2.1% to 20.8%. This geographic expansion equates to a net rise of 18.6 percentage-points, and a significant relative increase of 869.8% (Fig. 3). The states of Rondônia and Mato G rosso in Brazil had the largest percentage increase of over 3000% in the area affected by extreme drought lasting longer than 6 months. In 2024 the area affected by a drought lasting longer than 6 months reached a record-breaking 40.2%.

### 1.3 Climate suitability for infectious disease transmission

#### Indicator 1.3.1 Dengue

*Headline finding: the average* *climate-defined* *transmission potential of dengue by Aedes aegypti increased by 66% from 1951 to 1960 to 2020–2024 in Latin America, with an estimated basic reproduction number (R*_*0*_*) growing from 1.5 to 2.5.*

Climate change is altering environmental conditions (e.g., temperature, humidity) that, in turn, alter how and where mosquitoes grow and survive, thereby affecting their capacity to transmit diseases like dengue, zika, chikunguña, and yellow fever.^24^ This indicator, drawing from the 2025 global Lancet Countdown report, tracks the climatic suitability for transmission potential of dengue virus by *A. aegypti*, estimated by the basic reproduction number (R_0_).

**Fig. 4:**
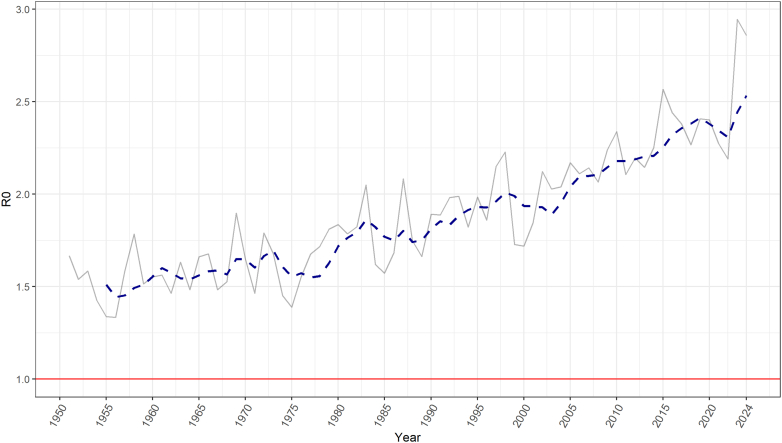
Annual estimated reproduction number (R_0_) for *A. aegypti* in Latin America from 1950 to 2024. Dotted line represents 5-year simple moving average. Red line represents (R_0_) = 1, where there is no epidemic potential.

The estimated regional average R_0_ for *A. aegypti* has steadily increased between 1951 and 2024 (Fig. 4), with a relative increase of 66% between 1951–1960 and 2020–2024, aligning with record-breaking dengue outbreaks in Latin America in the 2023–2024 period. Virtually all Latin American countries have experienced an increased in the estimated R_0_ over time, with Bolivia (+135%), Brazil (+108%), Honduras (+98%), Guatemala (+96%), and Peru (+95%) having the highest relative increases between 1951–1960 and 2020–2024.

#### Indicator 1.3.2 Vibriosis

*Headline finding: between 2015 and 2024, the total length of coastline with environmental conditions favourable for* *non-cholera* *Vibrio transmission in Latin America increased by 6.7% (5,948,286 km*^*2*^*) compared to 1990–1999.*

Climate change impacts, such as warming coastal waters in Latin America, create more favourable conditions for *Vibrio* spp. bacteria, potentially increasing the risk of water borne infections like vibriosis.^25^ This indicator estimates the area (km^2^) of the coastal waters with suitable seawater conditions for the transmission of *Vibrio* spp. bacteria.

In Latin America, between 2015 and 2024, the total length of coastline with environmental conditions suitable for non-cholera *Vibrio* transmission increased by 6.7% compared with 1990–1999. Over the same period, the population living within 100 km of these suitable coastal waters more than doubled, increasing by 101.7%, from 50,949,824 to 102,781,673 people at risk. Estimated non-cholera vibriosis cases also saw a record increase of 101.8%, climbing from 21,851 in the baseline period to 44,085 in the recent period. For *Vibrio cholerae*, the causal agent of cholera disease, the percentage of coastal waters suitable for the water-borne pathogen in Latin America have remained higher than expectations since 2000 to date.

### Conclusion

This section provided evidence on priority climate-related risks to human health at a regional, national, and sub-national level, in line with the Belém Health Action Plan.^26^ The indicators convey a concerning overview of escalating climate-related hazards, exposures, and health impacts across Latin American countries. Populations are experiencing substantially higher mean temperatures (indicator 1.1.1) and unprecedented exposure of vulnerable groups to heatwaves (indicator 1.1.2), leading to increased heat-related mortality (indicator 1.1.4) and significant heat stress related to physical activity (indicator 1.1.3). Furthermore, the region faces increasing threats from wildfires, evidenced by greater exposure to high fire danger and harmful PM_2.5_ (indicator 1.2.1), alongside a dramatic expansion of land area affected by extreme and prolonged droughts (indicator 1.2.2). Concurrently, environmental conditions are becoming increasingly suitable for the transmission of vector-borne diseases like dengue (indicator 1.3.1) and water-borne pathogens such as *Vibrio* species (indicator 1.3.2). These multifaceted findings unequivocally demonstrate a substantial and worsening threat to public health and population health throughout Latin America associated with a rapidly changing climate.

Panel 2 **Compounded climate hazards and cascading health risks in Latin America: the need for comprehensive climate-and-health surveillance.**

In 2023–2024, Latin America experienced compounded climate and environmental events, which led to multiple cascading health risks. The central threat of these events lies in their synergistic nature: the combined impact is often far greater than the sum of the impacts of individual hazards.^27^

During the 2023–2024, Brazil was affected by multiple and severe disasters, where the interaction of different hazards magnified the impacts on the environment and the population. The combination of prolonged drought, intense heatwaves, and devastating wildfires created a critical situation in vast regions of Brazil.

Widespread drought was observed in the Amazon and Pantanal in 2023–2024, with rainfall 30%–40% below normal and the Rio Negro in Manaus reaching a record low.^4^ Between August and December 2023, Brazil and other South American countries reported record maximum temperatures (even temperatures 10 °C above normal) and multiple heatwave episodes, aggravated by El Niño, which worsened the Amazonian drought.^28^ The 2023–2024 record-breaking drought in the Brazilian Amazon was likely driven by climate change, deforestation, and El Niño.^29^ This means human land-use change is a pre-existing condition interacting with climate hazards to worsen outcomes.

The increase in mean temperature and other ecological changes is related to an increase of dengue cases. In the state of Rio Grande do Sul, which had not previously experienced high levels of dengue, cases surged dramatically in 2024, increasing from 3988 in 2020 to 197,077.^30^

Simultaneously, the drought (which reduces air humidity, decrease forest evapotranspiration, and increase flammability of vegetation) and extreme heatwaves fuelled unprecedented wildfires in the Amazon and Pantanal regions in 2024.^4^ The Pan-Amazonian region impacted by forest disturbances (due to fire) increased by 152% from 2023 to 2024 (6.64 million hectares), of which Brazil suffered the largest absolute large-scale degradation in 2024.^31^ In 2024 alone, 22.38 million hectares burned in Brazil.^32^ Similar degradation has been observed in the Peruvian (779,960 million hectors) and Bolivian Amazon (47,574 million hectors).^33^ These figures illustrate the devastating scale of the fires, directly linked to the preceding drought and heat, having important consequences to health due to PM2.5 smoke exposure. Based on the data from the Climate and Health Observatory from Fiocruz, wildfires caused an increase in hospitalisations for respiratory problems, from 3300 in 2021 to 9300 in 2023 in the Brazil's Legal Amazon area–comprised of the nine northern states.

Parallel to the droughts and fires, Brazil also suffered intense rainfall events and subsequent flooding. Heavy rainfall events and flooding in Rio Grande do Sul became one of Brazil's worst climate disaster. Above-average rainfall from late April to early May 2024 affected numerous municipalities.^34^ The impacts were severe: over 180 deaths, hundreds of thousands displaced, and other less recorded water-borne diseases, such as leptospirosis.^4^ From an environmental perspective, drought conditions cause soil to dry and compact, reducing its absorption capacity and making areas more susceptible to flooding and landslides when heavy rains occur.

Compound climate events intensify and complicate their consequences for human health and wellbeing (and health systems as well): the synergistic and cascading effects far exceed what would be expected from isolated hazards. The problem is that traditional risk assessments and health surveillance systems often fail to capture these amplified implications and co-exposures (i.e., to extreme heat and air pollution simultaneously).

There is an urgent need for systems capable of tracking multiple hazards, population exposure, vulnerability factors, and health impacts in near real-time. Indicators must span hazards (e.g., heat intensity, rainfall anomalies, air quality), exposure (e.g., populations in affected areas, duration of exposure), and impacts (e.g., hospital admissions for specific conditions, mental health service use, food insecurity levels).

The Belém Health Action Plan, to be finalised at COP30 in Brazil, aims to foster ambitious commitments in the health sector to address climate change.^26^ A central pillar is “surveillance and monitoring”, which focuses on strengthening health surveillance and monitoring systems to effectively detect, prevent, and monitor climate-related health threats. It is expected that international agreements on indicators allow national and local governments and public health agencies to effectively strengthen their monitoring and responding capacities considering compounded and cascading health risks.

## Section 2: adaptation, planning, and resilience for health

Escalating and rapidly evolving climate hazards and their health impacts (Section 1) require a robust focus on health adaptation, strategic and long-term planning, and enhanced resilience within current health systems and overall society. Considering the global sociopolitical landscape, it is vital that adaptation and resilience actions for health in Latin American countries, both current and future, align with global discussions (e.g., GGA). It is also crucial to move beyond planning and translate commitments into concrete actions that protect health. (Panel 3). At the same time, adaptation and resilience actions for health in Latin America should prioritise local perspectives and strong intersectoral and multi-agency collaboration that considers local knowledge and evidence.

To track the state and progress of health adaptation, planning, and resilience efforts in Latin America, this section analyses ten indicators covering assessments and planning of health adaptation, enablers for adaptation and implementation, and vulnerabilities and health risks (Panel 1). Detailed methods and additional analyses for each indicator are presented in the Appendix.

### 2.1 Assessment and planning of health adaptation

#### Indicator 2.1.1 National assessments of climate change impacts, vulnerability, and adaptation for health

*Headline finding: as of May 2025, seven of 17 (41.2%) Latin American countries have publicly reported having conducted a vulnerability and adaptation assessment since 2020.*

Vulnerability and adaptation assessments (V&A) are a critical first step for adaptation planning and implementation.^35^ This indicator collects data from the Alliance for Transformative Action on Climate and Health (ATACH),^36^ the 2021 WHO Health and Climate Change Global Survey,^37^ and the 2023 PAHO Climate Change and Health Survey, to track public V&A assessments in the region.

As of May 2025, seven of 17 Latin American countries (41.2%) have reported the development of V&A: Argentina, Brazil, Chile, Ecuador, Guatemala, and Panama, Peru. Interestingly, Panama indicated that its V&A assessment contributed to the development of new policies or influenced existing policies related to climate change and health. In 2023, Argentina and Panama reported allocating some human or financial resources to support V&A efforts. Key health priorities emerging from completed assessments include zoonotic diseases, preparedness for extreme events, heat-related health impacts, and building health system resilience. Five countries (i.e., Colombia, Costa Rica, El Salvador, Nicaragua, Paraguay) reported ongoing development of their V&A assessments. Bolivia, Honduras, Mexico, Uruguay, and Venezuela reported no V&A or provided no survey response.

#### Indicator 2.1.2 National adaptation plans for health

*Headline finding: as of May 2025, nine of 17 (53%) Latin American countries have publicly reported having developed a Health National Adaptation Plan (HNAP) since 2020.*

Despite acknowledging HNAPs as a critical policy tool for climate and health adaptation, Latin America has shown limited development and progress in integrating health into climate policies.^38^ This indicator collects data from ATACH,^36^ the 2021 WHO Health and Climate Change Global Survey,^37^ and the 2023 PAHO Climate Change and Health Survey.

As of May 2025, nine of 17 Latin American countries (53%) have reported the development of an HNAP since 2020: Argentina, Brazil, Chile, Colombia, Costa Rica, Guatemala, Paraguay, Peru, and Uruguay. El Salvador and Nicaragua continue to report their plans as under development, maintaining their 2021 status. Complementarily and at the broader adaptation-mitigation planning level, only Brazil, Ecuador, Panama, and Uruguay have submitted updates of their National Determined Contribution (NDC), which were due for all countries in early 2025. However, the integration of health considerations into these broader national climate commitments remains limited.^39^

#### Indicator 2.1.3 City-level climate change risk assessments

*Headline finding: in 2023, 203 of 321 local administrative units in Latin America voluntarily reported having a* *city-level* *climate change risk and vulnerability assessment, a 39% increase compared to the previous report.*

While national climate commitments are crucial, adaptation is implemented locally. In Latin America, where 80% of the population lives in cities,^40^ local governments are central to reducing climate risks through diverse interventions, including urban planning, active mobility, air quality management, and green infrastructure.^41^ This indicator tracks climate risk and vulnerability assessments at the local level using publicly available data from the 2023 CDP Annual Cities Survey.^42^ This database includes voluntary and self-reported data from 321 local administrative units (municipalities from different administrative levels in the region) in Latin America, which represents approximately 2% of the lower level administrative units in the region.^43^ Additionally, a rapid review was performed on Google to track other assessments not captured in the CDP Survey.

**Fig. 5:**
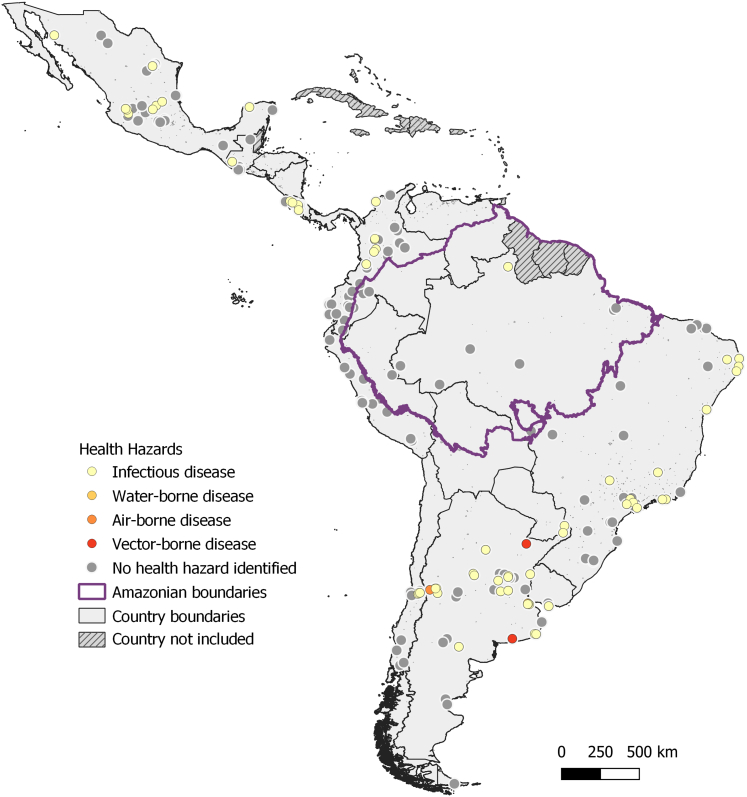
Local administrative units in Latin America that participated in the 2023 CDP Annual Cities Survey and responded to the “climate risk and vulnerability” section. Each point represents a local administrative unit. Administrative units are colour-coded to indicate the health hazards identified. The purple line represents the Amazonian boundaries.

City-level risk and vulnerability assessments remain limited across Latin America. Of those participating in the survey (n = 321), 203 local administrative units reported having completed an assessment, representing an increase from 149 local administrative units in 2022.^44^ Additionally, 34 administrative units reported they are conducting assessments, 58 are planning to do so in the next 2–3 years, 22 opted against future assessments, and four did not respond. The most frequently identified climate-related hazards were related to floods, storms, and heavy precipitation events (n = 186), followed by drought-related hazards (n = 129) and extreme temperatures (n = 124) (Fig. 5). Health issues driven by these climate hazards, such as infectious diseases or water-borne diseases, were recognised by only 54 administrative units. Notably, these health-related hazards were more frequently reported in administrative units located at higher latitudes rather than in tropical regions or the Amazon, suggesting potential gaps in recognition or reporting across different geographic contexts. An additional 26 local administrative units, not included in the CDP survey, had climate action plans or risk and vulnerability assessments via the Global Covenant of Mayors and are detailed in the Appendix.

### 2.2 Enabling conditions, adaptation delivery, and implementation

#### Indicator 2.2.1 Climate information for health

*Headline finding: in 2024, 10 of the 17 (58.8%) World Meteorological Organisation members in Latin America reported working with the health sector for the provision of climate services.*

Informing health decisions with robust climate data is vital for building climate-resilient health systems across Latin America. Achieving this requires strong inter-institutional collaboration between meteorological and health sectors, a principle supported by the Global Framework for Climate Services,^45^ and the Essential Public Health Functions, which includes environmental health surveillance and climate hazards as environmental determinants of human health.^46^ This indicator, utilising 2023 PAHO Climate Change and Health Survey and WMO data,^47^ assesses the extent of climate information for health.

As of 2024, 10 of the 17 World Meteorological Organization members in Latin America self-reported that their national meteorological and hydrological offices provide climate services for health services. These services are mainly focused on data services, climate monitoring, and climate analysis, with less emphasis on climate projections. Complementarily, seven of the 12 participant countries in the PAHO Climate Change and Health Survey indicated using meteorological information in their health surveillance systems, particularly for vector-borne diseases (n = 7), heat-related illness (n = 5), and airborne (n = 4), waterborne (n = 4), and zoonotic diseases (n = 4). Likewise, four countries reported having early warnings systems specifically for vector-borne diseases (i.e., Brazil, Guatemala, Paraguay and Peru) and heat-related illnesses (i.e. Argentina, Brazil, Guatemala, and Paraguay).

#### Indicator 2.2.2 Benefits and harms of air conditioning

*Headline finding: as of 2024, latest national data show that 27% of households in Latin America had air conditioning, with Paraguay reaching 60%. Overall, in 2019–2020, 18% of* *heat-related* *deaths were potentially averted by air conditioning use.*

Air conditioning (AC) use is rising across Latin America,^48^ potentially offering immediate relief from extreme heat. However, widespread reliance on house-based AC units represents a maladaptive strategy because it is energy-intensive and emits greenhouse gas (GHG), ultimately exacerbating the problem it seeks to address.^49^

This indicator, new to the *Lancet* Countdown Latin America report, uses data from seven national household surveys on AC use in Latin America and data from the 2025 global Lancet Countdown report at the regional level (indicator 1.1.4) for the potentially averted heat-related deaths due to AC.

Latest national data show that 27% of households in Latin America had air conditioning, with Paraguay reaching 60%. In 2019–2021, approximately 18% of heat-related deaths were potentially averted by air conditioning use. While this technology can play an important role in protecting populations during extreme heat events, its adoption is highly concentrated in high-income households.^49^ Most countries in the region lack widespread AC coverage, where it is often seen as a luxury. Furthermore, AC is energy-intensive and contributes to GHG emissions.^50^ Therefore, relying on AC can be considered a mitigation-adaptation trade off^51^ rather than a universally effective adaptation measure to prevent early mortality, especially among the most vulnerable populations.

#### Indicator 2.2.3 Urban greenspace

*Headline finding: in 2024, all 109 cities with over 500,000 inhabitants in Latin America were classified as having low, very low, or exceptionally low greenness levels.*

Rapid urbanisation and escalating climate hazards underscore the urgent need for strategic urban planning in Latin American cities. Integrating natural urban greenspaces is paramount for enhancing climate resilience and public health.^52^^,^^53^ However, green spaces will only be truly beneficial if they are designed with the local ecology in mind to avoid introducing unintended risks, such as new habitants for vectors or social inequities.^54^ This careful approach is vital amidst scarce resources and changing ecosystems.^55^ This indicator examines population exposure to urban greenspaces through population-weighted peak Normalised Difference Vegetation Index (NDVI) in 109 Latin American cities with over 500,000 inhabitants.

**Fig. 6:**
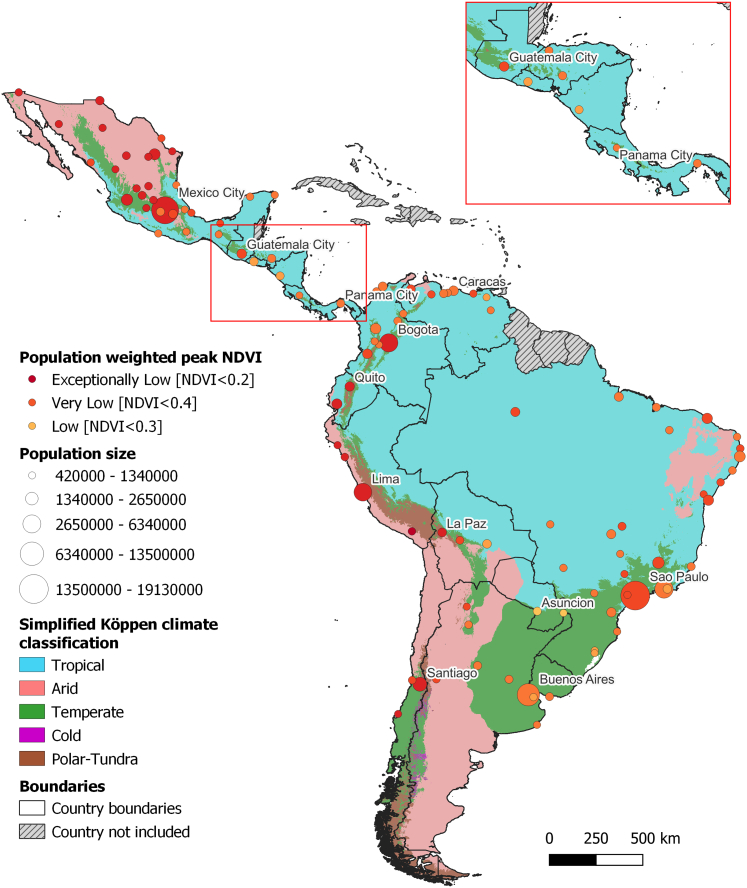
Greenness levels based on population-weighted peak NDVI in Latin American cities in 2024. City colours indicate greenness level and symbol size represents population size of each city. Background colours show the simplified Köppen climate classification.

Since 2015, none of the 109 Latin American cities analysed have reached high or above levels of urban greenness. Overall, regional greenness slightly declined by 2.7% between 2015–2017 and 2022–2024, with varied changes. The highest increases in greenness were seen in Venezuelan cities (e.g., Barquisimeto, Maracay) and the largest declines in Mexican cities (e.g., Toluca, Puebla). In 2024, all cities were classified as having low, very low, or exceptionally low greenness (Fig. 6), with levels of greenness remaining below global averages in all climatic zones. For the period 2022–2024, Asunción and Ciudad del Este (Paraguay) and Novo Hamburgo (Brazil) have the highest relative greenness, while Arequipa, Trujillo, and Lima (Peru) have the lowest.

#### Indicator 2.2.4 Detection, preparedness, and response to health emergencies

*Headline finding: in 2024, 11 of 17 Latin American countries* *self-reported* *high-to-very-high implementation of health emergency management capacity, with 3 countries downgrading since 2022.*

Effective plans for detecting, preparing for, and responding to health emergencies are paramount for dealing with climate-related hazards and fostering climate-resilient health systems.^56^ This indicator monitors the level of implementation of the legally-binding International Health Regulations core capacities 7 (health emergency management) and 3.2 (financing for public health emergency response) based on data reported by national authorities through the electronic IHR State Parties Self- Assessment Annual reporting Tool (e-SPAR).^57^^,^^58^ Core capacity 7 implementation scores classification are found in the Appendix.

The number of Latin American countries reporting high-to-very-high implementation of capacity 7 declined from 14 in 2022 to 11 in 2024. In 2024, Chile, El Salvador, Mexico, Uruguay, and Venezuela reported “very high” levels of implementation. Brazil, Costa Rica, Guatemala, Nicaragua, Panama, and Paraguay reported “high” implementation. Higher financial resources (capacity 3.2) positively correlated with higher health emergency management implementation in 2024, underscoring the critical role of investment in preparedness.

#### Indicator 2.2.5 Climate and health education and training

*Headline finding: in 2024, only 17% of students in* *survey-responding* *public health institutions and 63% in medical institutions received any training on climate change and health.*

Equipping health professionals to manage climate-related health impacts is crucial, yet dedicated education on this topic remains inconsistent across Latin America.^59^ This indicator, new to the *Lancet* Countdown Latin America report, relies on a self-reported, voluntary survey conducted from November 2024 to February 2025 among degree-granting institutions. The survey includes responses from 79 public health and 14 medical institutions, representing approximately 3% of medical institutions in the region.^60^

Among respondents, 45% of both public health and medical schools offered climate and health education. Mandatory training, however, was present in only 35% of these public health programmes, compared to 50% in medical schools. Of those participating in the survey, Brazil, Peru, and Argentina reported the highest number of public health institutions with climate education, while Colombia, Brazil, and Argentina provided most medical school responses. Institutions in Bolivia, El Salvador, Panama, and Venezuela reported no climate education in public health programmes. Of 104,699 enrolled students across public health institutions at the time of the survey, 17% received climate education, and 10% received mandatory training. Of 10,542 enrolled medical students, 63% received climate education, with 57% receiving mandatory training.

### 2.3 Vulnerabilities, health risks, and resilience to climate change

#### Indicator 2.3.1 Vulnerability and risk to mosquito-borne diseases

*Headline finding: overall, the drivers of vulnerability for* *mosquito-borne* *diseases decreased, leading to an historic, nearly 60% reduction since 2000 in the Mosquito Risk Index in Latin America.*

Vulnerability to mosquito-borne diseases, such as dengue, is influenced by biophysical, social, and economic factors, including access and quality of healthcare services, urbanisation, population density, and socioeconomic status.^24^^,^^61^ In a region with high variability in the climatic suitability for the transmission of dengue (indicator 1.3.1), combining vulnerability data with environmental suitability supports targeted resilience and adaptation efforts. The *Mosquito Risk Index* (MoRI) (0 representing the lowest risk and 1 representing the highest risk) compares the 2021–2023 average to historical, decade, and five-year averages, offering a clearer view of recent risk trends than single-year comparisons.

The overall MoRI score has declined in Latin America since 2000, mainly due to improvements in access to water, sanitation, and hygiene services. Nevertheless, Latin America has faced record-breaking dengue outbreaks in 2019, 2023, and 2024,^62^ highlighting the growing influence of climate and socioeconomic factors on mosquito-borne disease risk. Guatemala, Nicaragua, Panamá, and Venezuela account for the highest scores of MoRI. Venezuela's deterioration in the access to clean sources of drinking water exposes the country to a higher MoRI, compared to the region. Access to drinking water can bias MoRI by outweighing other socioeconomic drivers for mosquito-borne diseases, such as household conditions, water storage, and inadequate waste management.^63^ For example, even when Ecuador and Mexico had a very low MoRI since 2017, and 2020, respectively, due to reaching 100% coverage of clean drinking water services, both countries have experienced a resurgence of dengue transmission during the last years. This highlights that environmental factors –such as climate suitability-beyond water access significantly influence vector-borne disease outbreaks.

#### Indicator 2.3.2 Lethality of extreme weather events

*Headline finding: between 2000–* *2009 and 2015–* *2014, Latin American countries with Health Early Warning Systems (HEWS) experienced a 92.5% decline in mortality rate associated with floods and storms, while countries without HEWS experienced only a 43.4% decline.*

Climate change is intensifying the severity and frequency of floods and storms in Latin America, posing serious threats to health, wellbeing, and livelihoods.^12^

This indicator, new to the *Lancet* Countdown Latin America report, assesses mortality from such events using the EM-DAT database^64^ since 2000 in relation to self-reported climate-informed HEWS for injuries (2021 WHO Survey). However, analysis is restricted to selected countries (i.e., Argentina, Bolivia, Brazil, Costa Rica, El Salvador, Guatemala, Nicaragua, Paraguay, Uruguay) due to underreporting from other Latin American counties in the EM-DAT.

Between 2000–2009 and 2015–2024, virtually all countries evaluated experienced a decline in the observed mortality rate associated with floods and storms. However, countries with HEWS (i.e., Guatemala and El Salvador) experienced 92.5% decline, while countries without HEWS experienced 43.5% decline. Countries with the largest relative decrease between the two periods are Guatemala (−94.5%), El Salvador (−85.6%), Nicaragua (−78.9%), and Bolivia (−57.6%), while Uruguay had a slight increase (+29.7%, representing an increase from 0.09 to 0.12 deaths per 1000,000 people).

### Conclusion

The findings from this section illustrate a fragmented and uneven landscape: whilst Latin America is taking initial steps to adapt its health systems to a changing climate, progress is varied and unequal across the region. Nearly half of the countries have conducted V&A assessments for health (indicator 2.1.1), and over half have developed HNAPs since 2020 (indicator 2.1.2). These foundational plans are essential, yet the slow pace is mirrored in tangible adaptation measures: urban green spaces in all 109 major cities assessed remain low and have even seen a slight regional decline (indicator 2.2.3), depriving populations of a key strategy against extreme heat and other climate impacts. Furthermore, the number of countries reporting high levels of health emergency preparedness has decreased since 2022 (indicator 2.2.4), and there is limited integration of climate change into health professional training (indicator 2.2.5). This is particularly concerning in countries facing intersecting climatic and socioeconomic vulnerabilities to diseases such as dengue (indicator 2.3.1).

The capacity to manage climate-sensitive diseases, respond to increasingly frequent and intense extreme weather events, and protect vulnerable communities is being undermined. Yet, some progress is noted, such as an increase in city-level risk assessments (indicator 2.1.3) and the mortality reductions associated with HEWS for floods and storms in certain countries (indicator 2.3.2). These represent important steps forward, but further action is needed to achieve widespread and sustained progress across the region.

Panel 3 **Health adaptation under pressure: gaps between planning and action.**

Latin America has shown increasing institutional awareness in addressing the intersection of climate and health, with more countries and cities conducting vulnerability assessments and developing health adaptation plans. This may reflect an evolving understanding of climate change as a critical public health issue, leading to the gradual inclusion of health in regional adaptation agendas. However, progress is slower than expected. More critically, concrete adaptation implementation is largely stagnated or imperceptible, with few regional successes. This is reflected across South America, where most countries present health specific adaptation plans in their NAPs, yet 10 out of 12 countries provide no information on how to finance them, and 6 lack measurable indicators to track progress.^39^ This highlight the persistent gap between planning and effective implementation, driven by fragmented adaptation budgets, weak operationalisation across sectors, limited intersectoral coordination, misalignment between ministries, and a lack of shared objectives.

Latin American countries can learn from each other to accelerate the implementation of adaptation strategies. Brazil, given its geopolitical leadership in the region and COP-30 presidency is a key example to discuss. It was among the first in the region to advance health adaptation policies, evidenced by its National Policy on Climate Change from 2009 and the subsequent development of three editions (2013; 2016; 2025) of its HNAP.^65^ A key tool is the Fiocruz Climate and Health Observatory (Observatório de Clima e Saúde).^66^ This initiative effectively articulates data from climatic, environmental, socioeconomic, epidemiological, and public health sources to create early warning and monitoring systems, facilitate knowledge sharing, inform policy development, and enhance stakeholder collaboration. Complementing this effort, a recent institutional initiative is the Center for Synthesis on Climate Change, Pollution, and Biodiversity for strengthening innovation in the health system. The Center aims to generate integrated, interdisciplinary knowledge that supports health system innovation and informs territorial and intersectoral strategies for adaptation and mitigation. The Brazilian Ministry of Health is on the Interministerial Committee on Climate Change, coordinating multi-sector climate action. This is supported by Brazil's National Institute of Meteorology urban early warning system and ANDUS (Support for the National Agenda for Sustainable Urban Development). This intersectoral approach is key for impact and health needs. Increasing transparency through public consultation, including vulnerable groups—is integrated into policy reinforcing the principle of “leaving no one behind”.

However, despite progress, Brazil faces many challenges and implementation barriers of its climate policies due to a lack of formal prioritisation and budgetary integration.^67^ In the health sector, adaptation within the Unified Health System is not yet embedded in National Health Policies and Plans, leaving it vulnerable political shifts. Additionally, although the HNAP outlines relevant strategies, these are not yet fully integrated into official National Health Policies or multi-year health plans, resulting in a lack of dedicated budget lines or long-term financial commitments. Subnational implementation also remains weak due to limited or inconsistent agreements across levels of government. Brazil's experience underscores the necessity of robust governance mechanisms that transcend political cycles and ensure institutional memory. Embedding health adaptation into national and local policy frameworks, aligned with broader development plans, is crucial for translating plans into durable action.^56^

At this crucial juncture, Latin American governments should prioritise self-reliant knowledge sharing, strengthen internal communication, and concretely plan for a sustained shift. Multisectorial task forces with defined mandates and finance considerations (e.g., Colombia's Financial Management Committee, established under the Intersectoral Commission on Climate Change), as well as legislative frameworks that mandate subnational climate assessments and plans (e.g., Chile's 2022 Climate Change Law) are examples of measures that can support this shift. This integrative approach could enable governments to move towards concrete, measurable health adaptation implementation at all levels. Achieving tangible benefits requires long-term financing, technical capacity, robust regional cooperation, and stronger cross-sector coordination. Despite instability, health must remain a non-negotiable adaptation priority.

## Section 3: mitigation actions and health co-benefits

Efforts to decarbonise can significantly improve air quality in Latin America, which is a pressing concern for many cities grappling with high air pollution levels.^68^ Improved air quality directly benefits population health in the short- and long-term. Simultaneously, the region has historically been a vital global carbon sink, through its vast natural forests, though recent evidence indicates that parts of Amazonia, particularly eastern regions affected by deforestation and climate change, have shifted to become net carbon sources.^69^ In this context, protecting and enhancing natural ecosystems is not merely a global responsibility but also secures essential local ecosystem services and contributes to regional wellbeing.

To evaluate the progress on mitigation actions and their co-benefits to people's health, this section analyses eight indicators in several areas: energy use, energy generation, and health; air quality and health co-benefits; food, agriculture, and health co-benefits; tree cover loss and health; and healthcare sector emissions (Panel 1). Detailed methods and additional analyses for each indicator are presented in the Appendix.

### 3.1 Energy use, energy generation, and health

#### Indicator 3.1.1 Energy systems and health

*Headline finding: electricity generation from renewable sources had a net increase of 9.1* *percentage-points* *from 1991–2000 (2.7%) to 2014–2023 (11.8%), exceeding coal's share since 2014. Nevertheless, coal use also increased from 1991–2000 (2.6%) to 2014–2023 (5.2%).*

Replacing coal and other fossil fuels with renewable energy is fundamental for a healthy and sustainable future in Latin America. This transition not only reduces harm to human health and the environment but is also crucial for achieving climate goals. This indicator draws on data from the International Energy Agency (IEA) and the 2025 Lancet Countdown report and examines the share of energy sources (i.e., coal, low-carbon fuels as biogases or biofuels, and renewables) used for electricity generation.

**Fig. 7:**
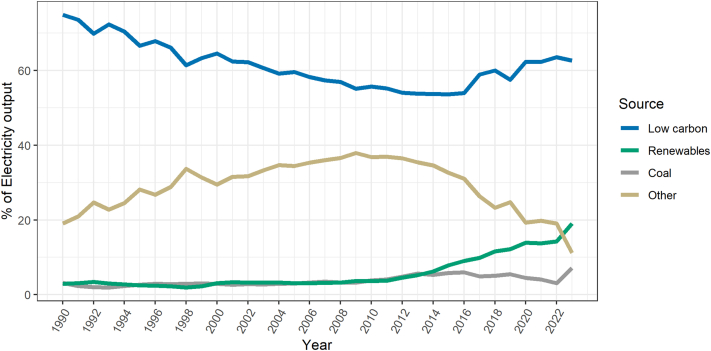
Annual percentage of electricity generation by source (low carbon, renewables, coal and other) in Latin America, from 1990 to 2023. Lines show the temporal trends for each energy source, expressed as share of total electricity output.

Between 1991–2000 and 2014–2023, Latin America's electricity generation sources changed. The share from low-carbon generation sources moved from 67.6% to 58.9% during this period, remaining the primary contributor (although with an increasing trend since 2016). Coal-fire's share increased from 2.6% to 5.2%, with a particular increase between 2022 and 2023. In contrast, renewable source generation (solar and wind) have steadily grown from 2.7% to 11.8%, overtaking coal's share in 2014 (Fig. 7). Overall, these figures demonstrate a mix shift towards renewable source generation, but still considerable use of coal. At a country level, almost all countries have increased their electricity generation from renewable sources between 1991–2000 and 2014–2023, with Uruguay (from 0% to 29.4%), Honduras (from 0% to 17.2%), El Salvador (from 15.3% to 32.3%), Nicaragua (from 15.9% to 32.9%), and Chile (from 0% to 14.4%), as the leading countries. Although the use of coal has decreased in the last decade, is still a significant source of electricity in Chile, Guatemala, and Mexico.

#### Indicator 3.1.2 Household energy use

*Headline finding: in 2023, 79% of the Latin American population relied on fossil fuels for cooking. 14% continue to use biomass, revealing stark inequities between urban (5%) and rural areas (31%).*

A “just energy transition”—a shift to a clean energy economy in a way that is fair and inclusive for everyone—is complex yet essential for Latin America. The region needs to shift from fossil fuels to equitable renewables for cooking, to meet global GHG goals and yield health co-benefits, such as reduced respiratory and cardiovascular disease, especially in women and children. This transition demands policy changes and investments. This indicator uses data from the WHO-SDG7 database, which tracks clean/non-polluting cooking fuel use (i.e., electricity, gas and other clean energy) and biomass (polluting fuels such as wood, dung, crops).^70^

Overall, there has been a decrease in biomass use since 2014 due to focused clean fuel policies,^71^ reducing PM_2.5_ exposure among vulnerable populations. Currently, 14% of the Latin American population still uses biomass (31% rural vs. 5% urban), usually lacking cleaner options. Significant regional disparities exist. In Central America, 46% of the rural population continue to use biomass, with Nicaragua (89%), Guatemala (81%), and Honduras (74%) showing the highest reliance. In South America, 20% of rural households lack access to clean fuels and continue to use biomass fuels, with Paraguay (44%), Colombia (34%) and Peru (30%) with the highest usage.

**Fig. 8:**
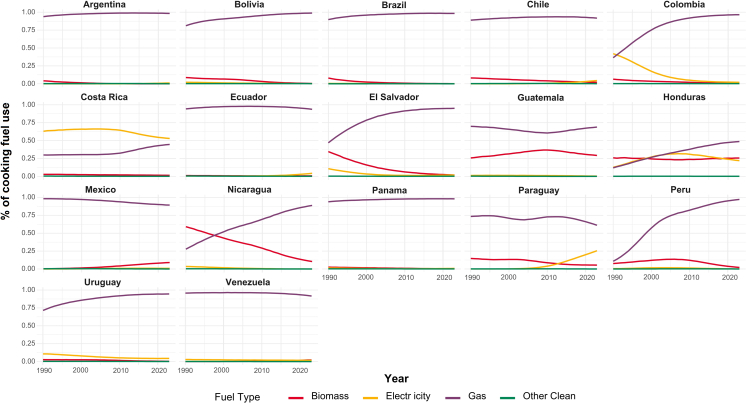
Annual proportion of household by cooking fuel source (electricity, gas, biomass, and other clean sources) in Latin American countries from 1990 to 2023. Lines show the temporal trends for each fuel type, expressed as share of total households.

As of 2023, 79% of Latin American households used liquified petroleum gas (LPG) for cooking, with notable urban-rural differences (85% vs. 63%), underscoring transition challenges. However, this dependence on fossil fuels for cooking is a major obstacle to reducing emissions. While WHO-SDG7 identifies LPG as a “cleaner” transitional fuel, the anticipated shift towards electricity is not materialising, with Paraguay exhibiting the only substantial adoption of electricity as the main source of fuel at approximately 25% of households. Interestingly, in countries like Costa Rica, Nicaragua, and Colombia, the increase in gas (LPG) use for cooking is accompanied by a decrease in electricity consumption, even in areas where electricity is available and has been used previously (Fig. 8).

#### Indicator 3.1.3 Sustainable and healthy transport

*Headline finding: fossil fuels are the predominant fuel for transport in Latin America, slightly declining from 98.9% in 1991–* *2000 to 96.7% in 2013–* *2022. Electric sources are slowly increasing, reaching only 0.04% in 2013–* *2022.*

Latin America faces an urgent need to transition to sustainable and healthy road transport systems to address severe air pollution, reduce emissions, and enhance urban liveability amid continuing urbanisation. This indicator draws on data from the IEA World Extended Energy Balances, as presented in the 2025 global *Lancet* Countdown report.

Despite modest changes, fossil fuels continue to dominate the region's transportation energy profile, powering 96.7% of road transport during 2013–2022, compared to 98.9% in 1991–2000. Biofuels have increased their share from 1.1% to 3.2% between these periods. Nevertheless, biofuels' contribution remains problematic due to concerns about lifecycle emissions, land-use impacts, and air pollutant production. Electricity's role in powering road transport remains minimal, growing from virtually 0% to just 0.04% over the study period. The most notable increases in electricity uptake occurred in Chile (+0.64%), Ecuador (+0.01%), Colombia (+0.007%), and El Salvador (+0.0002%).

### 3.2 Air quality and health co-benefits

#### Indicator 3.2.1 Premature mortality from ambient air pollution

*Headline finding: more than 360,000 premature deaths due to fossil* *fuel-related* *PM*_*2.5*_ *(coal and gas) were estimated in 2018–2022, 41,000 fewer deaths than in 2007–2011. However, more than 140,000 premature deaths due to PM*_*2.5*_ *from biomass were estimated in 2018–2022, 17,000 more deaths than in 2007–2011.*

Ambient PM_2.5_, primarily from fossil fuel combustion,^72^ caused over 90% of the 8.1 million air pollution-related deaths in 2021 globally.^73^ Since identifying specific pollution sources is crucial for effective health-promoting interventions, this indicator draws from the 2025 global *Lancet* Countdown report and presents estimates of premature mortality attributed to PM_2.5_ from coal, gas, and biomass combustion from 2007 to 2022.

**Fig. 9:**
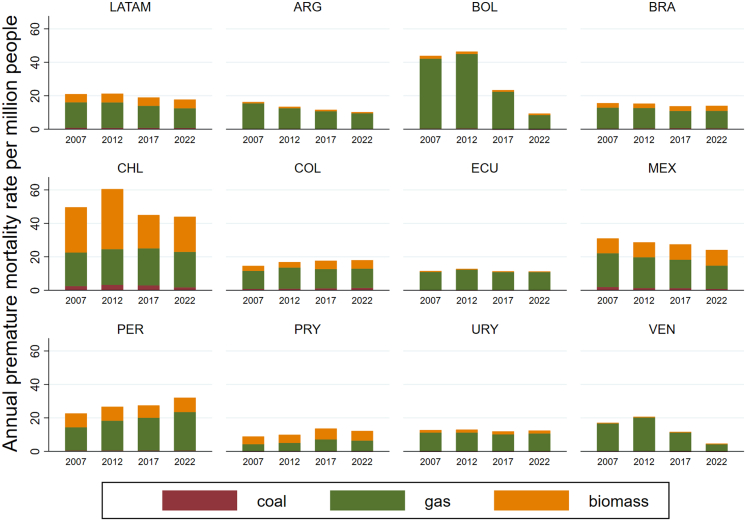
Annual attributable deaths per 100,000 people per fuel and country, from 2007 to 2022. Bars represent the total sum in mortality estimates, stratified by fuel type.

The rate of premature mortality attributable to PM_2.5_ from coal and gas combustion decreased by 17.7% and 20.3%, respectively, between 2007–2011 and 2018–2022 (∼363,000 premature deaths in 2018–2022, ∼41,500 fewer deaths than 2007–2011). Conversely, the mortality rate from biomass-related PM_2.5_ increased by 1.2% in the same period (∼144,000 premature deaths in 2018–2022, ∼17,400 more deaths than 2007–2011). Deaths attributable to coal largely decreased due to improvements from power plants (−26.1%), households (−10%), and industry (−7%) sectors. Deaths attributable to gas largely decreased due to improvements from industry (−63.8%), power plants (−59.2%), households (−26.8%), and transport (−12.3%). Nonetheless, biomass-related PM_2.5_ from power plants was associated with an increase of 206% in mortality rate, followed by households (+1.2%) (Fig. 9).

#### Indicator 3.2.2 Household air pollution

*Headline finding: in 2022, the use of polluting fuels and inefficient stoves in households for cooking and heating resulted in around 45,000 deaths and emitted 103 megatons (Mt) of CO* *_2._*

The use of polluting solid fuels (e.g., biomass, charcoal, coal) and inefficient cookstoves for house cooking and heating generate high levels of household air pollution (HAP) as well as CO_2_ emissions.^74^ For the purpose of analysing the HAP, a Bayesian hierarchical model^75^ was developed using sample data from an updated WHO Global Household Air Pollution database.^76^ The model was used to estimate annual average HAP-PM_2.5_ indoor concentrations for users of different fuel types (biomass, charcoal, coal), cookstoves (traditional and improved), and for separated rural and urban settings.

There is a large disparity in exposure to polluting solid fuels between urban and rural settings across Latin America. Annual average indoor concentrations of HAP-PM_2.5_ were estimated for 14 countries Argentina, Brazil, Bolivia, Colombia, Costa Rica, Ecuador, El Salvador, Honduras, Guatemala, Mexico, Nicaragua, Paraguay, Peru, and Venezuela. In 2022, the national average indoor concentration due to polluting solid fuels use for cooking and heating was estimated at 245 μg/m^3^ [95% CI 387–102]. This concentration was higher in rural households 314 μg/m^3^ [95% CI 498–129] compared to urban households 145 μg/m^3^ [95% CI 225–66].

Exposure to HAP from polluting solid fuels has a substantial public health impact. The PM_2.5_ attributable death rate (per 100,000 population) from this exposure at the national level averaged 61 [95% CI 75–46], with a rural average of 66 [95% CI 80–53] and an urban average of 47 [95% CI 58–36]. This resulted in an estimated 45,000 deaths across these 14 countries in 2022.

Achieving net-zero emissions for the cooking sector by 2050 requires a complete transition away from solid fuels (biomass, coal, and charcoal) to cleaner fuels. The total CO_2_ emissions from solid fuels use for cooking and heating across 14 Latin America countries are estimated at 131 Mt. Of this total, 102 Mt of CO_2_ emissions originated from rural areas and 29 Mt come from urban areas. When considering only the fraction of biomass harvested unsustainably,^77^, ^78^, ^79^ total CO_2_ emissions are estimated at 28 Mt (20 Mt rural, 8 Mt urban). With average HAP-PM_2.5_ exceeding WHO limits (5 μg/m^3^).

### 3.3 Food, agriculture, and health co-benefits

#### Indicator 3.3.1 Emissions from agricultural production and consumption

*Headline finding: although GHG emissions from cow and buffalo meat consumption have decreased by 20.4% and production by 1.7% between 2000–2009 and 2013–2022, they remain the leading source of* *agricultural-related* *GHG emissions in Latin America.*

Agriculture and livestock production significantly contributes to GHG emissions in Latin America, largely driven by livestock rearing for key products like beef and dairy, alongside other agricultural activities. Addressing these emissions is crucial for regional climate targets and sustainable food systems.^80^ This indicator draws from the 2025 global *Lancet* Countdown report and analyses GHG emissions from agricultural production and consumption.

In 2013–2022 in Latin America, consumption of beef and buffalo meat drove greatest carbon dioxide equivalent (CO_2_e) emissions per capita (0.5 tonnes of CO_2_e per capita, on average) of any food commodity, followed by dairy products (0.17 tonnes of CO_2_e per capita, on average). In terms of production, these emissions increased up to 0.74 and 0.18 tonnes of CO_2_e per capita, on average, respectively.

Comparing the overall trend between 2000–2009 and 2013–2022, the commodities with the largest increase in CO_2_e for consumption and production, on average, were palm oil (+51.3% and 70.3%, respectively) and poultry (+36.2% and 34.2%, respectively). Although consumption-based emissions of beef and buffalo meat, have decreased by 20.4%, and production-based emissions decreased by only 1.7%, beef and buffalo meat and dairy products are still the main contributors of CO_2_e emissions. For dairy products, these emissions have decreased by 17%, on average. Guatemala (consumption and production), Nicaragua (production), and El Salvador (consumption), and Paraguay (production) have importantly increased their CO_2_e emissions associated with beef and buffalo meat from 2000 to 2022.

#### Indicator 3.3.2 Diet and health co-benefits

*Headline finding: between 2021 and 2022,* *all-cause* *deaths related to unhealthy diets increased by 1% (from 1.28 to 1.3 per 1000 people), with deaths due to respiratory diseases, cancer, and coronary heart disease having the largest relative increases (over +4.5%).*

Adopting healthy, sustainable, plant-based, nutritious, and local diets is crucial for wellbeing and planetary health in Latin America. Current diets high in red and processed meats and ultra-processed foods, increase the risk of non-communicable diseases like cardiovascular disease and diabetes. This dietary pattern contributes to premature deaths, higher GHG emissions, and widespread environmental degradation.^81^ This indicator draws from the 2025 global *Lancet* Countdown report and estimates diet-related health burdens in Latin America.

Between 2021 and 2022, the rate of all-cause mortality associated with unhealthy diets in each Latin American country rose, on average, from 1.28 to 1.3 deaths per 1000 people (1% relative change). Venezuela (+4.5%), Argentina (+3.2%), Brazil (+2.7%), Colombia (+2.1%), and Panama (+2%) showed the highest relative increases. By cause, the highest increase was estimated for respiratory diseases (+3%), followed by type 2 diabetes (+2.2%), and coronary heart disease (CHD) (+1.2%). However, country level analyses showed important differences: deaths due to stroke increased by 10.5% in Guatemala and Argentina, CHD deaths increased by 6.2% in Venezuela, and respiratory diseases increased by 5.9% in Ecuador.

The largest relative increases in all-cause mortality rate attributable to excessive intake of animal-based products between 2021 and 2022 were estimated for dairy products (+8.1%), poultry (+2.9%), red meat (+1.9%), and processed meat (+0.4%). Respiratory diseases, cancer, and CHD associated with excessive intake of animal-based products increased by 6.3%, 4.5%, and 4.5%, respectively.

### Indicator 3.4 Tree cover loss and health

*Headline finding: from 2001–* *2010 to 2014–* *2023, tree cover loss due to shifting agriculture, wildfires, and commodity driven deforestation has increased by 31%, 29%, and 12%, respectively.*

Forests in Latin America serve as crucial carbon sinks, biodiversity reservoirs, and determinants of human health.^82^ Forest loss exacerbates climate change through carbon emissions, reducing natural filtration of air and water pollutants, and disrupting ecosystem services essential for Indigenous communities' health, livelihoods, and cultural practices.^83^

**Fig. 10:**
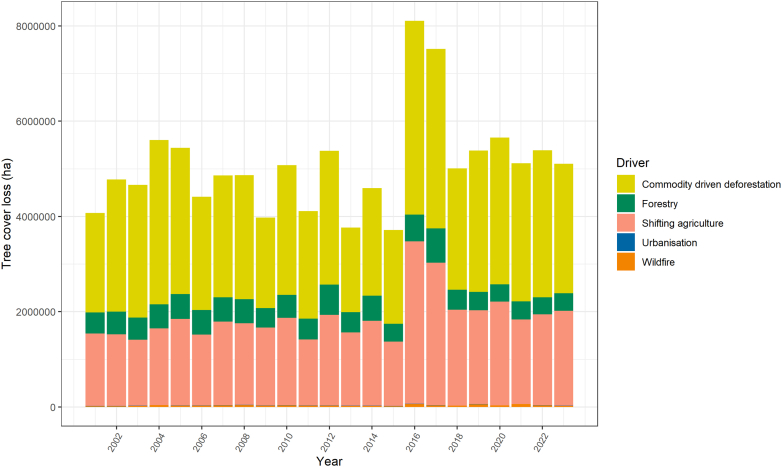
Annual tree cover loss (in hectares) in Latin America between 2001 and 2023 disaggregated by main driver (commodity-driven deforestation, forestry, shifting agriculture, urbanisation, and wildfire). Bars represent stacked contributions of each driver to total tree cover loss per year.

In 2014–2023, the main drivers of tree cover loss in Latin America are commodity driven deforestation (∼173,000 ha annually), shifting agriculture (∼126,000 ha annually), forestry (∼26,000 ha annually), and wildfires (∼2600 ha annually) (Fig. 10). From 2001–2010 to 2014–2023, tree cover loss due to shifting agriculture, wildfires, and commodity driven deforestation has increased by 31%, 29%, and 12%, respectively.

In 2023, Brazil (55%), Bolivia (13.6%), Peru (4.5%), Mexico (4.4%), and Argentina (4.2%) accounted for the highest proportions of the approximately 5.1 million hectares of tree cover lost across Latin America. Looking at the drivers, commodity driven deforestation and shifting agriculture in Brazil accounted for 32% (∼1.6 million ha) and 19% (∼1 million ha) of the region's tree cover loss, followed by commodity driven deforestation in Bolivia, accounting for 11% (∼578,000 ha).

Comparing 2023 vs. 2014–2022, Bolivia (53%), Nicaragua (22%) and Uruguay (12%) were the Latin American countries with the largest increase in tree cover loss. The subnational analysis reveals critical tree cover loss hotspots in 2023 compared to 2014–2022 averages: Bolivia's El Beni Province (305%) and La Paz Province (104%), and Honduras' Gracias a Dios Department (104%).

Additionally, the highest average losses during 2020–2023 were for Pará (743,439 ha) and Mato Grosso (562,463 ha) in Brazil and Santa Cruz (374227 ha) in Bolivia. The highest accumulated tree cover loss for the same period was 16,997,417 ha for Pará, 13,484,672 for Mato Grosso, and 6,292,802 for Maranhão.

### Indicator 3.5 Healthcare sector emissions

*Headline finding: from 2010–2014 to 2018–2022, Latin America's* *healthcare-related* *GHG emissions per capita increased by 12.4%.*

Globally, healthcare accounts for 4–5% of GHG emissions,^20^^,^^84^^,^^85^ making sector-wide decarbonisation vital for public health. In Latin America, whilst current healthcare emissions are comparatively lower, their concerning upward trend demands early action to avoid entrenching carbon-intensive systems.

This indicator, new to the 2025 *Lancet* Countdown Latin America report, draws from the 2025 global *Lancet* Countdown report. This indicator uses an environmentally-extended multi-region input–output (EE-MRIO) model, EXIOBASE, combined with national healthcare expenditure data to measure both direct emissions from healthcare facilities and indirect emissions from the consumption of goods and services supplied by other sectors. It summarises key pollutants: GHGs, ozone, and PM_2.5_. This indicator tracks change in average emissions for 2018–2022, using 2010–2014 as a reference, and also compares emissions with each country's life expectancy.

Overall, healthcare CO_2_e emissions per capita in each Latin American country have followed an increasing trend since 2010. Between 2010–2014 and 2018–2022, these emissions grew, on average, from 118.1 to 134.9 kg of CO_2_e per capita (+14.3%). Countries with the largest relative increases were Panama (+75.7%), Bolivia (+73.3%), Guatemala (+52.2%), Peru (+33.8%), and El Salvador (+31.2%). On the other hand, Brazil (−5.15%), Colombia (−6.18%) and Argentina (−9.99%) had a decrease in emissions. In 2018–2022, the top four countries with the highest annual healthcare CO_2_e emissions per capita were Uruguay (344 kg), Argentina (311 kg), Chile (274 kg), and Panama (274 kg).

**Fig. 11:**
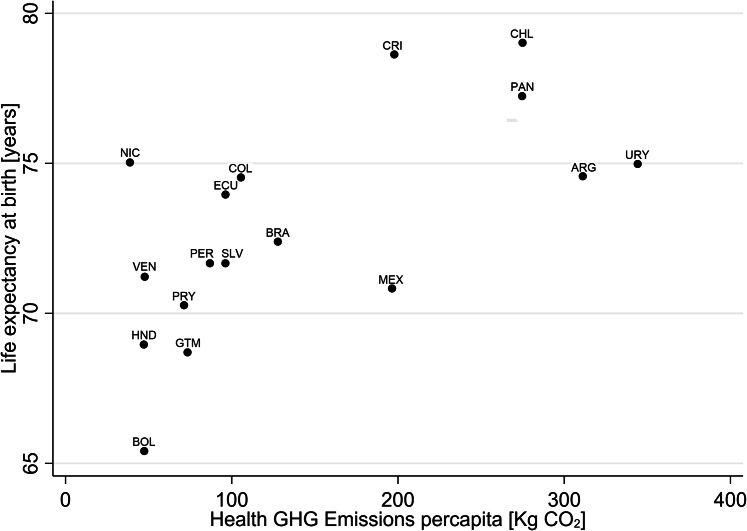
Relationship between Life Expectancy at Birth and healthcare-related GHG emissions per capita (kg CO_2_e) per country in Latin America (2022). Each point represents a country.

Complementary data obtain from the World Bank show that countries with higher healthcare emissions also tend to achieve better health outcomes, including higher healthy life expectancy (Fig. 11). This relationship shows that Latin American countries must navigate the challenge of decoupling the growth of their healthcare sectors from their environmental impact, ensuring that improved health services do not come at the cost of increased emissions.

### Conclusion

Section 3 reveals both challenges and opportunities in Latin America's climate mitigation landscape: demanding urgent attention to rising healthcare emissions (indicator 3.5), persistent air pollution (indicator 3.2.2), continued tree cover loss (indicator 3.4), and increasing diet-related mortality (indicator 3.3.2). These same areas offer pathways for transformative action with substantial health co-benefits. Unfortunately, these findings continue to highlight critical inequities that must be addressed: rural communities face disproportionate exposure to household air pollution (indicator 3.2.2), while forests experience intense deforestation pressure (indicator 3.4).

Addressing disparities must be central to mitigation planning. Key priorities emerge from this section: accelerating a just energy transition away from fossil fuels (indicators 3.1.1, 3.1.2, and 3.1.3), towards the regions abundant clean energy sources (solar, geothermal, hydro, wind) (indicator 3.1.1); addressing forest loss through integrated approaches, and satellite-based deforestation monitoring systems; and transforming food systems toward plant-forward diets and agricultural policies that deliver climate-health co-benefits (indicators 3.3.1 and 3.3.2). These transitions offer significant potential for simultaneous climate and health gains.

Importantly, healthcare sector emissions are rising, attached to higher investments and services demands, adding more pressure to the environment and the healthcare system itself. Unfortunately, no Latin American country has yet developed a roadmap to align the strategies and investments to strengthen public health while reducing GHG emissions. Some countries in the region are moving forward developing their own emission analysis, conducting bottom-up analysis like Mexico, Colombia, and Chile, but more region-wide action is needed to achieve climate-resilient and low-carbon healthcare systems.

These indicators offer essential evidence for developing policies that protect both human and planetary health. Regional successes (Panel 4) show that progress is achievable with political will and evidence-based approaches, even amidst economic and political pressures hindering a swift renewables energy transition.

Panel 4 **A systemic food system transformation for health, climate, and biodiversity.**

The expansion of agricultural frontiers, particularly for large-scale commodity production (i.e. soy bean, coffee, corn, sugar and meats) is primarily driven by economic interests to meet national and global demand and increase export revenues. These process leads to deforestation, land degradation, and biodiversity loss, exacerbating climate change by reducing carbon sinks and harming human health by damaging crucial ecosystem services. From 2001 to 2024, Brazil lost 73.3 Mha of tree cover, equivalent to a 14% decrease in tree cover and 38.2 Gt of CO_2_e emissions.^86^

Mitigation strategies, such as regenerative agriculture and agroecology offer solutions by avoiding expansion,^87^^,^^88^ restoring land, and conserving biodiversity. Forest restoration, however, requires a societal shift in consumption towards diverse, plant-based options to improve health outcomes and reduce environmental impacts. Governments play a crucial role in facilitating this transition through key policy frameworks, primarily by providing financial incentives for farmers to adopt regenerative practices and by implementing clear and comprehensive food labelling and other incentives to guide consumer choices towards sustainable and healthy options (e.g., ultra-processed foods taxes, controlling pesticide use).^89^^,^^90^

Brazil has taken on several initiatives in that direction, such as the Dietary Guidelines, the National School Feeding Programme (PNAE), and the Food Acquisition Programme (PAA), which support access to nutritious food while fostering sustainable agricultural practices. The PNAE reaches ∼40 million school students and is supported by an annual budget of around R$4 billion (∼US$764 million), with a legal requirement to source at least 30% of food from local family farmers, thereby bolstering sustainable agriculture and local economies.^91^ Similarly, the PAA has been shown to increase the gross income of family farmers, with 51% of beneficiaries being women.^92^

Other environmental policies, such as the Payment for Environmental Services (PES) and the Minimum Price Guarantee Programme for Socio-Biodiversity Products, support family farming and traditional communities to maximise environmental and support local livelihoods. PES uses financial mechanisms like government funding, user fees, and ecological sales tax revenues to promote conservation. In previously deforested areas, positive impact has been reported in the Brazilian Cerrado with an observed increase of 2.2% in native vegetation seven years after implementation.^93^ These programmes often focus on crucial ecosystem services like water provision, carbon sequestration and biodiversity protection, aiming to create direct economic link to environmental stewardship.

Ultimately, a systemic food systems transformation is vital to cut deforestation, prevent disease spillover, protect biodiversity, and lessen climate-related health risks. Strengthened monitoring and surveillance, including real-time alert systems like the Real-Time Deforestation Detection System are essential to avoid persistent deforestation. Tools like Brazil's SISS-Geo, a surveillance tool for early-disease-detection in wildlife, prevent potentially harmful effects on humans and can be included in Brazil's national monitoring system. Combined with enhanced epidemiological monitoring, Tools like Brazil's SISS-Geo, a surveillance tool for early-disease-detection in wildlife, prevent potentially harmful effects on humans and can be included in the national monitoring system. Combined with enhanced epidemiological monitoring, human-livestock-wildlife surveillance would allow for the early detection of sensitive vector-borne disease outbreaks.^94^^,^^95^

Transformation of the food system is a key mitigation strategy that yields immediate health co-benefits and significant environmental advantages by reducing emissions, capturing carbon and reducing pressure on ecosystems, ensuring equitable access to clean, sustainably produced food, significantly boosting biodiversity, strengthening food security, and supporting climate action.^96^

## Section 4: economics and finance

The escalating health impacts of climate change (Section 1) pose significant economic burdens. Strained healthcare systems, lost productivity, and damaged infrastructure generate substantial costs, demanding robust strategies to safeguard current and future national economies and to protect livelihoods. Addressing this emergency requires a dual approach that includes significant and sustained investment in adaptation and mitigation measures across the region (Sections 2 and 3). Far from mere expenditure, these investments are foundational for building resilient, equitable, and healthier societies capable of thriving amidst a changing climate.

To track progress in economic costs of climate change and related investments in Latin America, this section tracks six indicators on three main areas: the economic impacts of climate change and its mitigation; the transition to net zero-carbon, health-supporting economies; and financial transitions for a healthy future (Panel 1). Key results are summarised in Panel 5, which highlights the regional costs of climate-related damages, heat-related productivity losses, and gaps in adaptation and mitigation finance. Detailed methods and additional analyses for each indicator are presented in the Appendix.

### 4.1 The economic impact of climate change and its mitigation

#### Indicator 4.1.1 Economic losses due to weather-related extreme events

*Headline finding: in 2024, extreme weather disasters in Latin America cost the region US$19.2 billion, 0.3% of the region's GDP (gross domestic product), with Brazil bearing* *two-thirds* *of the cost.*

Climate-driven extreme events like storms, floods, droughts, extreme heat, frosts, hailstorms, and wildfires are steadily eroding economic resilience in Latin America. Drawing on loss estimates from the EM-DAT database^64^ and GDP figures from the IMF World Economic Outlook,^97^ every extreme weather-related event recorded in 17 countries was aggregated, and the resulting losses were expressed as a share of GDP for 2010–2024.

Economic losses reached US$19.2 billion in 2024, 0.3% of the region's combined GDP, the second largest burden since 2010. Despite this spike, medium-term averages remain flat; losses averaged US$9.2 billion a year in both 2010–2014 and 2018–2024, keeping the GDP ratio near 0.16%. Volatility has increased, with years in which damages exceeded 0.1% of GDP rising from four in 2010–2014 to six in 2018–2024. In 2024, Brazil accounted for US$13.6 billion, just over two-thirds of the regional total, after El Niño-driven floods in the south and record Amazon wildfires. Mexico followed with about US$2.6 billion, and Chile, hit by severe wildfires in February, recorded US$2.1 billion losses. Far smaller absolute figures were registered in Argentina (≈US$0.25 billion), Peru (≈US$0.19 billion) and Colombia (≈US$0.15 billion), while the remaining countries each incurred less than US$0.1 billion. Relative to national output, Chile (≈0.63% of GDP) and Brazil (≈0.63%) shared the highest proportional impacts, followed by Mexico (0.14%), Panama (0.13%), Ecuador (0.08%) and Peru (0.07%).

#### Indicator 4.1.2 Costs of heat-related mortality

*Headline finding: the average annual monetised value of* *heat-related* *mortality for 2015–2024 was US$855 million, an increase of 229% from 2000 to 2009.*

Quantifying the value of lives prematurely lost to heat strengthens the case for targeted public health interventions and for climate adaptation and mitigation investments. This indicator, sourced from the 2025 global *Lancet* Countdown report, calculates these costs by combining heat-related mortality data (indicator 1.1.4) with the value of a statistical life-year.

Between 2015 and 2024, the average annual monetised cost of heat-related mortality in Latin America reached US$855 million, representing a significant increase of 229% when compared to the average annual costs during the 2000–2009 period (US$260 million). This estimated cost underscores the escalating economic toll of rising temperatures in the region. Countries with the largest relative increases in monetised costs between these two periods include Ecuador (+668%, US$214 million), Guatemala (+454%, US$311 million), Honduras (+450%, US$118 million), Colombia (+374%, US$939 million), and El Salvador (+370%, US$149 million). In absolute terms, Brazil (US$3664 million), Mexico (US$2027 million), Argentina (US$1094 million), Colombia (US$939 million), and Venezuela (US$518 million) recorded the highest cost increases.

#### Indicator 4.1.3 Loss of earnings from heat-related labour capacity reduction

*Headline finding: Latin America's potential earnings lost due to* *heat-related* *labour capacity losses in 2024 reach US$52 billion (0.81% of GDP), a 12.6% increase from 2023, mostly impacting agriculture and construction workers.*

Excessive heat compromises workers’ wellbeing and productivity, reducing incomes and impacting broader socioeconomic conditions, including overall national development. Drawing on the 2025 global *Lancet* Countdown report, this indicator measures heat-induced labour capacity reductions, expressed as potential work hours lost, across four key sectors: services, manufacturing, construction (under the sun), and agriculture (under the sun). By multiplying these lost hours by the average hourly wage for each sector and year at the national level, the indicator calculates the corresponding earnings shortfall, reported in billions of real 2024 USD and as a percentage of GDP.

**Fig. 12:**
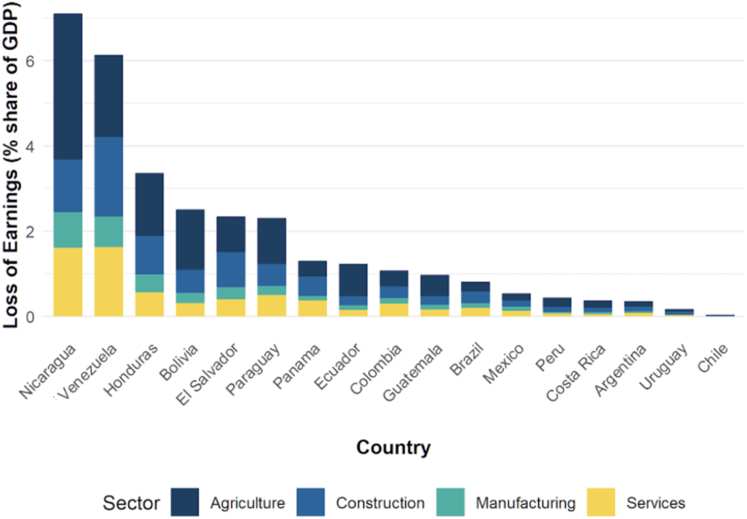
Loss of earnings from heat-related labour capacity reduction by sector (agriculture, construction, manufacturing and services) in Latin America in 2024.

In 2024, heat-related labour capacity reduction across 17 Latin American countries with available data resulted in a total potential income loss of US$52 billion, equivalent to 0.81% of the regional GDP, a 12.6% rise from US$46.2 billion in 2023. Nicaragua experienced the highest loss relative to GDP (7.1%), followed by Venezuela (6.1%), Honduras (3.4%), Bolivia (2.5%), and El Salvador (2.3%), while Chile recoded the lowest (0.03%) (Fig. 12).

Across the region, the agricultural sector accounted for the largest share of potential income losses (34.3% of total), followed by construction (29.1%), services (23.3%), and manufacturing (13.4%). Several countries experienced over half of their total losses in agriculture, notably Ecuador (67.0%), Bolivia (59.4%), Peru (58.2%), Paraguay (55.0%), Guatemala (53.6%), Nicaragua (52.6%), and Honduras (52.4%). In contrast, Chile lost the majority of its potential earnings (60.8%) in construction, followed by Uruguay (39.6%), Panama (39.0%), and El Salvador (34.9%).

#### Indicator 4.1.4 Costs of the health impacts of air pollution

*Headline finding: the monetised value of air pollution mortality in Latin American countries was US$160 billion (2.8% of GDP) in 2022—equivalent to 15.8 million people's annual income.*

Air pollution, particularly anthropogenic PM_2.5_, remains a significant environmental threat to public health in Latin America (indicator 3.2.1), with premature deaths among working-age individuals imposing substantial social and economic burdens. These losses reduce household income and diminish workforce productivity. Drawing from the 2025 global *Lancet* Countdown report, this indicator quantifies the economic impact of PM_2.5_ air pollution-related premature mortality by monetising years of life lost (YLLs). Due to limited data, the analysis only covers Mexico and South American countries.

In 2022, the monetisation of YLLs attributable to PM_2.5_ in 11 Latin American countries amounted to US$160 billion, equivalent to 2.8% of their aggregate GDP. This cost corresponded to the annual average income of about 15.8 million people in the region. This represents a 2.1% increase from 2007 and a 6.3% rise from 2021 to 2022. Brazil, Colombia, Ecuador, Mexico, Paraguay, and Peru recorded the highest absolute losses (around US$129.5 billion or 2.93% of their GDP), followed Argentina, Chile, and Uruguay with US$28.8 billion (2.7% of their GDP), and Bolivia and Venezuela with US$1.78 billion (1.2% of their GDP).

These estimates do not capture the full economic costs such as increased healthcare spending due to morbidity and reduced labour productivity, suggesting the true impact of air pollution is likely far greater.

### 4.2 The transition to net zero-carbon, health-supporting economies

#### Indicator 4.2.1 Country preparedness for the transition to net-zero

*Headline finding: in 2024, preparedness for the transition to* *net-zero* *in Latin America reached a score of 0.44, well below the world average (0.52). Uruguay, Chile, and Costa Rica led the regional ranking.*

Achieving net-zero GHG emissions is essential to protect population health and ensure long-term prosperity. To prepare for this transition, countries should reduce their reliance on fossil fuels, strengthen institutional capacity, expand domestic capabilities, and establish governance frameworks that support a fair and inclusive shift. Drawing on the 2025 global *Lancet* Countdown report, this indicator evaluates national transition preparedness using a composite index of 25 weighted variables (e.g., macroeconomic stability, access to renewable resources, reliance on fossil fuel, carbon intensity), yielding a score from 0 (least prepared) to 1 (most prepared).

In Latin America, preparedness for the transition to net-zero remains closely linked to the level of Human Development Index (HDI) (r ≈ 0.87). In 2024, the regional average score reached 0.44, below the world average (0.52). Uruguay (0.67), Chile (0.65), and Costa Rica (0.6) led the regionnas the most prepared countries for transition to net zero, while Nicaragua (0.31), Bolivia (0.33), and Guatemala (0.35) were the least prepared.

### 4.3 Financial transitions for a healthy future

#### Indicator 4.3.1 Net value of fossil fuel subsidies and carbon prices

*Headline finding: in 2023, all Latin American countries had* *net-negative* *carbon prices, with fossil fuels subsidies totalling US$38.6 billion—nearly 50 times the revenue from explicit* *carbon-pricing* *schemes—and averaging 38% of national health budgets.*

Fossil fuel subsidies divert public resources to activities that harm human health and the environment, undermining societal wellbeing. When these subsidies exceed the revenues generated by carbon-pricing schemes, the economy faces a net-negative carbon price—effectively paying to emit rather than charging for emissions. Progressively eliminating these subsidies and reallocating funds to support a net-zero transition can prioritise health and equity, reducing fossil fuel dependence sustainably while accounting for countries’ varying capacities. This indicator draws on the 2025 global *Lancet* Countdown report, and compares fossil fuel consumption subsidies with carbon-pricing revenues to estimate the net economy-wide average carbon prices and revenues.

In 2023, every country reported a negative net carbon price, resulting in a regional net subsidy to fossil fuels of US$38.6 billion. Venezuela (–US$13.8 billion), Argentina (–US$9.1 billion), Colombia (–US$7.5 billion), and Brazil (–US$4.6 billion) accounted for over 90% of this total. Although the regional net subsidy declined by 52% since 2010—driven largely by reductions in Brazil and Mexico—it remains approximately fifty times larger than the US$0.8 billion generated by explicit carbon-pricing schemes. Relative to national health budgets, net subsidies equalled 241% in Venezuela, 35% in Bolivia, 26% in Colombia, 18% in Ecuador, and 16% in Argentina, diverting resources that could strengthen health-system resilience and accelerate decarbonisation.

#### Indicator 4.3.2 Health adaptation finance flows and disclosed needs

*Headline finding: between 2020 and* *2022, countries received US$197 million in principal bilateral health adaptation finance and US$77.7 million from the Green Climate Fund for* *health-related* *adaptation projects.*

Adequate and accessible finance is fundamental for Latin American countries to implement essential health adaptation measures and build climate-resilient health systems. This is particularly vital given the region's long-standing underfunding in the climate and health sectors.^98^

This indicator draws from the 2025 global *Lancet* Countdown report, and tracks financial flows directed primarily towards health-related climate adaptation in Latin America. Data on financial supply are drawn from Organisation for Economic Co-operation and Development reports on sovereign bilateral, private, and philanthropic flows, supplemented with fund-level information from the Green Climate Fund.

Between 2020 and 2022, bilateral commitments for health-focused adaptation projects in Latin America totalled approximately US$197 million, supporting 10 countries. Brazil received the largest share (US$133), followed by Venezuela (US$8.6), Guatemala (US$8.5), Bolivia (US$8), and Peru (US$7.9). Since 2017, the GCF has supported 37 health-related adaptation projects in Latin America, including projects that also benefitted countries in other regions, allocating US$3.4 billion. Adaptation projects with potential health benefits with only Latin American countries as beneficiaries received a total of US$77.7 million (14% of total budget per project, on average) between 2020 and 2022. In 2024, this figure was US$36.9 million for four projects (17% of total budget per project).

### Conclusion

These indicators reveal that climate-related shocks are imposing a growing toll on Latin American economies and health systems. In 2024, weather-related disasters consumed 0.3% of GDP (indicator 4.1.1); heat-related deaths cost US$855 million per year (indicator 4.1.2); lost earnings due to heat equalled 0.8% of GDP (indicator 4.1.3); and deaths from air pollution absorbed 2.8% of GDP (indicator 4.1.4). Additionally, the level of preparedness to net-zero at the regional level is well-below the world average and varies across Latin American countries (indicator 4.2.1). Fossil fuel subsidies still exceed carbon pricing revenue by almost 50:1 (indicator 4.3.1), and health adaptation support covers just a fraction of the stated needs (indicator 4.3.2).

Progressively eliminating fossil fuel subsidies and aligning carbon prices with decarbonisation goals would release billions of dollars annually. Redirecting these funds to clean energy, climate-resilient infrastructure, and nature-based solutions could reduce emissions, mitigate extreme heat exposure, and lower air pollution mortality, easing pressure on health budgets. Complementary measures are essential to protect workers: occupational-heat standards, early-warning systems, and shaded workspaces can curb productivity losses and keep workers safe, while investments in clean transport and industrial processes would reduce both ambient PM_2.5_ levels and GHG emissions.

With COP30 on the horizon, Latin American governments can convert these findings into tangible pledges: schedules for phasing out fossil fuel subsidies, carbon pricing pathways consistent with net-zero, and investment plans with health co-benefits. Delivering these requires concessional finance and debt-relief instruments respecting fiscal constraints. By centring health in climate-finance negotiations, countries can argue that redirecting funds from fossil fuel support to adaptation and mitigation yields immediate benefits: fewer premature deaths, higher labour productivity, and lower long-term health costs. Accelerated, integrated action on these three fronts is indispensable to curb the rising economic costs of climate change and to secure a sustainable and equitable future for all.

Panel 5 **Economic costs of extreme weather events: health consequences and financial burdens.**

Climate change increasingly strains Latin America's health systems and communities, with extreme weather events like heatwaves, floods, and droughts occurring more frequently and intensively.^4^ Such events are burdening healthcare infrastructure and regional economics. Additionally, ongoing fossil fuel subsidies exacerbate air pollution and climate-related health risks, delaying clean energy transitions and escalating economics costs.

Brazil exemplifies these challenges. In May 2024, severe floods in Rio Grande do Sul affected 96% of municipalities, caused 183 deaths, displaced 600,000 people, impacted over 2.4 million residents, and generated economic losses of R$88.9 billion (∼US$17.3 billion), including R$1.5 billion (∼US$0.29 billion) in healthcare sector damages.^99^ Heatwaves in 2023 further strained Brazil's healthcare system, increasing demand for medical care, particularly among vulnerable groups like older adults, children, and individuals with chronic diseases.^100^ Similarly, prolonged droughts in Uruguay and Brazil triggered emergency expenditures for water provision and healthcare, while fuelled wildfires in the Amazon and Pantanal that drove hospitalisations for respiratory illnesses.^4^

Scientific evidence shows the widespread health and economic impacts of global warming in Latin America. In Colombia, climate change contributes to higher mortality rates, with projected health costs reaching US$7.8 billion annually by 2050 without robust adaptation.^101^^,^^102^ Argentina, Chile, and Paraguay face rising mortality and morbidity from extreme temperatures, accompanied by significant economic losses.^103^, ^104^, ^105^ Currently, less than 5% of economic losses from extreme events are insured, leaving countries highly vulnerable.^106^

Recent climate-finance negotiations reveal persistent gaps. At COP29 in Baku, parties adopted the New Collective Quantified Goal of US$ 300 billion per year by 2035, tripling the previous target but far below the trillions needed to limit warming to 1.5 °C and protect public health. The Loss & Damage Fund, with pledges of US$768 million as of April 2025, has transferred less than half and operational delays may postpone grants until 2026.^107^ Health-specific financing also faces challenges: the US$1 billion “climate-and-health” package announced at COP28 lacks a transparent disbursement schedule or monitoring framework, hindering progress tracking.^108^

Looking ahead to COP30 in Brazil, it is essential for Latin America to address three key priorities: (1) establish risk-transfer mechanisms to prevent disasters from escalating public debt; (2) progressively eliminate fossil-fuel subsidies and integrate climate-stress tests and health-climate taxonomies into national financial systems; and (3) bridge an annual health adaptation and resilience funding gap exceeding US$100 billion, even if the US$300 billion global goal is met. Without enforceable commitments, the rising health and economic cost of climate hazards will outpace financial remedies, making alignment of public, private, and multilateral flows with net-zero and resilience pathways the most rational strategy to safeguard the region's health and future.

## Section 5: public and political engagement with health and climate change

Advancing effective public policies and action plans at the intersection of health and climate change in Latin America requires empirical evidence and a deep understanding of how diverse actors such as governments, scientists, news media, corporations, and citizens engage with these interconnected issues over time. Synergistic political commitment and broad societal engagement are critical: while political will enables the creation and funding of innovative climate policies, active public participation fosters awareness, drives behavioural change, and creates momentum for sustained climate action. As underscored in Panel 6, this change must have equity at its core, as integrated, cross-cutting approaches lead to better health outcomes.

Understanding engagement dynamics is key to catalysing collective efforts and developing shared, effective solutions to safeguard public health from the threats posed by climate change. This section analyses six indicators on engagement with health and climate change across diverse areas: media and social media engagement; scientific articles on health and climate change; political engagement (including a new regional indicator: funding for science); and corporate sector engagement. Detailed methods and additional analyses for each indicator are presented in the Appendix.

### Indicator 5.1 Media engagement with health and climate change

*Headline finding: newspaper coverage of climate change decreased in 2024 compared to the previous two years, while the proportion of climate change articles mentioning health-related terms moderately increased.*

Mainstream news outlets play an important role in setting political and public agendas in Latin American countries. Drawing on data from the 2025 global *Lancet* Countdown report, this indicator identifies how frequent news articles in leading newspapers from ten Latin American countries included a set of keywords related to “climate change” and “climate change and health” between 2007 and 2024.

In 2024, coverage of climate change across all sources decreased by 14.1% from the 2022 record. While it remained at a similar level in 2023 compared to 2022, it decreased in 2024, with a total of 3941 articles in the ten Latin American newspapers. Although the overall coverage reduced, the proportion of climate-related articles that also mention health-related words has grown from 27.5% in 2022 to 30.9% in 2023 and 29.9% in 2024.

Mentions and trends per country vary extensively in volume, often with high, event-driven peaks. Although newspapers from Chile, Colombia, Ecuador, and Mexico showed an increase in overall coverage and health-related terms between 2022 and 2024. From these newspapers, *La Nación* from Argentina showed the highest proportion of articles with health-related terms across all countries in 2024, 40.2%, despite the fact that its overall climate coverage was reduced by 61.7% from 2022.

### Indicator 5.2 Social media engagement with health and climate change

*Headline finding: social media posts related to health and climate change increased by 256% from 2017 to 2023.*

Social media platforms shape public discourse, allowing users to access and engage with information in ways traditional media cannot. While social media may foster polarisation, they also convey societal awareness and evolving concerns related to climate change. This indicator tracks social media engagement with health and climate change in 17 Latin American countries using CrowdTangle data from 2017 to 2023.

The total number of posts grew by 256% from 2017 to 2023, with the highest number of posts in 2022. In 2023, Brazil, Mexico, Costa Rica, Chile, and Argentina generated the highest volume of posts relative to their population. User engagement (i.e., “likes”) was highest in Colombia, Brazil, Mexico, Argentina, and Chile. Media accounts represented the largest share of posts (55.7%), while political accounts made up only 9.8%. However, when looking at interactions rather than post volume, media accounts and political accounts generated the same amount of interaction (29.0%). This contrast highlights how political content, despite being less frequent, sparked disproportionately high engagement.

### Indicator 5.3 Scientific articles on health and climate change

*Headline finding: Latin American publications represent 5.5% of global output in 2024. Brazil remains the regional leader, and* *equity-related* *topics remain largely absent from the research.*

Scientific research on the relationship between climate change and health provides crucial evidence for informing public, private, and political responses.^109^ This indicator, drawing on the 2025 global *Lancet* Countdown report, measures the scientific engagement in this intersection by tracking the number of relevant publications.

**Fig. 13:**
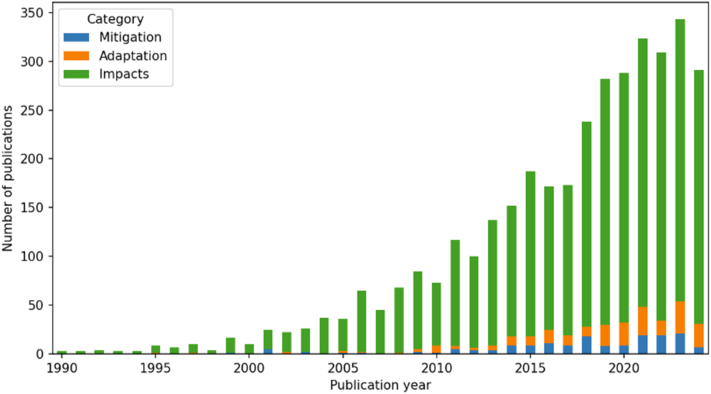
Estimated number of papers published on the nexus of climate and health in Latin America, from 1990 to 2024. Bars represent annual counts of publications.

The number of scientific publications on health and climate change has continued to increase (Fig. 13). However, Latin America contributes only 5.5% of the global output, with 70% of its research occurring post-2015. Despite a 67% increase in its own publications from 2015 to 2024, the region's share of the global total decreased by 2.1 percentage-points. On average, publication grew by 7% per year during this period, while their global share declined by 0.2 percentage-points annually. Research on health impacts largely outpaces studies on mitigation or adaptation.

Publications with at least one author affiliated with a Brazilian academic institution account for the increase in publications in the region, primarily focusing on vector-borne diseases and extreme weather impacts on health. Notably, research on mental health, food security, heat-related health issues, as well as equity themes like poverty, vulnerable populations, and the specific experiences of Indigenous and Afro-Latino communities, remain limited in the region's scientific literature.

### 5.4 Political engagement with health and climate change

#### Indicator 5.4.1 Government commitment with health and climate change in UNGA & NDCs

*Headline finding: health mentions in Latin American NDCs rose by over 1129% between the first and second rounds, increasing from 80 to 1112. Thematic growth was most pronounced in references to wellbeing, disease, and* *health-related* *infrastructure.*

Political and governmental commitment are key drivers for climate action and societal engagement.^110^ This indicator analyses United Nations General Assembly (UNGA) discourses and NDCs, drawing on the 2025 global *Lancet* Countdown report.

Mentions of climate change in UNGA speeches first peaked in 2007 (with 13 countries referencing it) and have since established, with 14 countries mentioning it in 2023 and 11 in 2024. In contrast, the intersection of climate and health received peak attention in 2010 (mentioned by 10 countries) but had a significant drop to only Bolivia, Brazil, and Chile in 2024.

General health mentions in NDCs surged by 1129% between the first and second editions. Argentina, Chile, Panama, and Uruguay showed the biggest increase in diverse health themes. Beyond general health, wellbeing and disease were most frequent, indicating a focus on prevention and quality of life. Notably, only Chile, Panama, and Uruguay consistently mentioned mental health, and only Uruguay and El Salvador addressed extreme heat. Uruguay consistently integrated health across its climate plans, while Brazil's health narrative remains limited. Analysis of the ongoing third NDC round will reveal if these trends continue.

#### Indicator 5.4.2 Funding for science on health and climate in Latin America

*Headline finding: less than 1% of publicly funded research projects in six Latin American countries addressed the intersection between climate change and health in 2024.*

Sustained government funding for climate and health research in Latin America is paramount. It generates essential, local evidence for tailored adaptation and mitigation policies while building crucial local scientific capacity.^44^

This new regional indicator tracks the proportion of publicly funded research explicitly focused on climate change and health in Latin America between 2020 and 2024. It is based on a two-stage review of national research funding databases from six countries (i.e., Argentina, Chile, Mexico, Panama, Peru, and Uruguay) with open and public reporting systems. First climate change projects were selected by title and then re-screened for health relevance.

The analysis identified a total of 2063 funded research projects between 2020 and 2024. Of these, 8.2% were explicitly related to climate change, and 0.63% addressed the intersection between climate change and health. These findings reveal a very low level of investment in climate change-related projects across the region, particularly those addressing its connection with health.

### Indicator 5.5 Corporate sector engagement with health and climate change

*Headline finding: despite the decline between 2023 and 2024, corporate mentions of climate and health in Latin America have doubled since 2011.*

The corporate sector can significantly impact GHG emissions and public health, with many joining the UN Global Compact to promote sustainability and social and environmental responsibility. This indicator draws from the 2025 global *Lancet* Countdown report and tracks mentions of climate change and health terms in the Global Compact Communication of Progress reports.

Over time, Latin American companies increasingly mention climate change and its health nexus in annual reports. “Climate change” mentions rose from 58% (2011–2015) to 70% (2020–2024), while “climate change and health” grew from 15% to 37%. “Health” mentions remained stable around 90%. Peak mentions for all were in 2023 (climate 84%, health 93%, intersection 52%), followed by a 2024 decline (climate 69%, health 89%, intersection 42%). Despite this recent decline, intersection mentions (42% in 2024) show significant progress from 2011 (17%) and 2022 when it was around 31%.

Regardless of the recent decrease in intersection mentions, the 2024 figure (42%) still represents substantial long-term progress compared to 2011 (17%) and even 2020–2022 averages (around 30%). The 2023 peak suggests a growing corporate awareness, though sustained commitment is needed.

### Conclusion

This section highlights the complex and evolving landscape of public and political engagement at the intersection of health and climate change in Latin America. While long-term trends across key indicators show a steady rise in engagement, recent setbacks in some indicators and persistent gaps highlight ongoing challenges.

While news coverage of climate change has generally followed an upward trend, it declined across the region in 2024. Health-related reporting continued to show a modest increase, suggesting a growing understanding of climate change as a public health crisis (indicator 5.1). This upward trend connecting health an climate change is mirrored in social media, where the volume of posts concerning health and climate has grown substantially since 2017, indicating increasing public engagement (indicator 5.2). However, analysis is limited to capturing interaction and cannot assess the veracity of these posts. The potential for polarisation and the spread of misinformation on these platforms remains a pressing concern.

Scientific publications on health and climate change have increased significantly in the region since 2015, though Latin America accounts for only 5.5% of global output (indicator 5.3). However, the lack of attention to equity-related themes is concerning. While some research addresses poverty, intersecting issues such as race, gender-based violence, and Indigenous rights remain largely overlooked. This underscores the urgent need for more inclusive, representative research agendas that reflect the region's diverse realities. At the same time, although national public funding mechanisms for research exist, few projects that explicitly address the relationship between climate change and health are funded. This highlights the need to strengthen the inclusion of this topic in research agendas to generate locally relevant evidence and respond to the region's specific challenges (indicator 5.4.2).

There are encouraging developments on the political and private fronts. An increasing number of governments are incorporating equity considerations into their NDCs (indicator 5.4.1), referencing vulnerable populations, environmental justice, and Indigenous rights. Similarly, corporations have increasingly engaged with the interaction of health and climate change, but 2024 saw a decrease in mentions, which suggests the need for them to more strongly commit to addressing this issue (indicator 5.5).

Panel 6 **Looking beyond Belém: from commitment to action.**

Health has gradually gained prominence in UN climate talks, particularly through Presidency-led programmes since COP26 in 2021. However, a paradigm shift is still urgently needed to move from siloed policymaking to integrated governance, where health considerations guide climate strategies. The Brazilian COP30 Presidency's initiative, the Belem Health Action Plan for Climate and Health, is designed to deliver a tangible health outcome by focusing on health sector adaptation. It operates through three strategic lines: health surveillance and monitoring; evidence-based policy and capacity building; and innovation and production. Guiding all of this is the COP30 Presidency's emphasis on equity and the elevation of underrepresented voices in climate and health policy processes, seeking to ensure UNFCCC processes are closely connected to people's daily lives.^26^

Equity is not just a matter of justice—it is also a direct determinant of better health outcomes. Climate policies that consider differential vulnerability and promote equitable access to clean energy, water, sanitation, and health services reduce avoidable harm and improve collective resilience.^111^ The true transformational potential of health can therefore only be achieved if it is integrated cross-cuttingly across climate negotiations and policies.

Health is a central “super-leverage point” for driving systemic change in climate action.^112^ As highlighted by the IPCC's Sixth Assessment Report, aligning objectives across sectors unlocks large-scale, multiple benefits while avoiding short-term harm. Integrating health into all dimensions of climate negotiations connects the UNFCCC process directly to people's lives, generating immediate health benefits while fostering long-term, sustainable change.

A decade after the Paris Agreement, achieving the ambitious and equitable joint action needed to safeguard the health of both people and the planet remains a distant goal. Nevertheless, various global governance institutions have made significant strides. The World Health Assembly, in particular, adopted the Global Action Plan on Climate and Health. This plan urges member states to incorporate health into their NDCs and embed climate considerations into national health strategies. It emphasises maximising the health co-benefits of mitigation and adaptation and commits to involving communities and civil society in the process.^113^

Moreover, both the Inter-American Court of Human Rights and the International Court of Justice have released their views on countries' obligations regarding climate change. They have established that climate change is a “common concern of humankind” because it gravely endangers human rights by impacting the environment, the very foundation of life.^114^ States are now legally obligated to prevent significant harm and may be held responsible for emissions that harm people beyond their borders.^115^ These judicial opinions, which align with the WHO Global Plan, provide a clear, cross-sectoral framework for climate action with people at its heart.

In a powerful display of independent, cross-sectoral collaboration, more than 50 organisations from Latin America and the Caribbean came together to launch a common position on Climate and Health. This collective effort urges governments and the international community to take urgent action rooted in equity and justice.^116^ The road to COP30 has shown that linking health to climate action is no longer optional; it is central to an effective and just response. Belem is a concrete platform to connect this cross-sectoral momentum to the urgent needs of Latin American countries, and its lasting legacy will depend on whether health is fully embraced as a central driver of ambition and implementation.

## Conclusion of the 2025 *Lancet* Countdown Latin America report

The *Lancet* Countdown Latin America report demonstrates the escalating health impacts of anthropogenic climate change across Latin America. Section 1 indicators reveal an alarming trend of intensifying climate hazards, putting individuals and society at risk. Individuals are increasingly exposed to extreme heat, with a mean ambient temperatures increase of 1 °C compared to 2001–2010, rising from 23.3 °C to a record-high 24.3 °C. Consequently, infants experienced a staggering 450% more days of heatwave exposure, while those over 65 faced an even more concerning 1000% increase in exposure compared to 1981–2000. For older adults, the relative increases reach catastrophic levels in Venezuela (5116%) and Colombia (5910%). Heat-related mortality increased in 103%, an approximately 13,000 deaths annually. This heat-related mortality incurred an average annual monetised cost of US$855 million during 2015–2024, a significant 229% increase compared to 2000–2009. Heat-related labour losses in 2024 totalled a substantial US$52 billion, a 12.6% rise from 2023, disproportionately impacting agriculture and construction.

Furthermore, the increasing frequency and intensity of droughts have affected most countries in the region. Subnational analysis reveals that areas affected by at least one month of extreme drought conditions increased from 15.8 to 59.1%, with Brazil, Bolivia, and Mexico showing the most significant percentage changes. A similar trend is evident for more prolonged 3- and 6-months droughts. This rise likely contributed to the extreme wildfire risk observed in 2024, which led to an increase in person-day exposure across Latin America. The most substantial increases were seen in Chile (30.5 days, 105% rise), Mexico (17.6 days, 28.5% rise), and Bolivia (16.7 days, 82.6% rise). These extreme events led to out-of-pocket economic losses, exacerbated by the fact that less than 5% of economic losses are insured, totalling nearly 19.2 billion in 2024. Brazil accounts for two-thirds of the regional total economic loss, followed by Mexico and Chile. While these findings are presented separately, many of these climate events occur simultaneously, leading to cascading health risks. This was observed in several countries where the combination of prolonged drought, intense heatwaves, and wildfires magnified the impacts on the environment and population (Panel 2).

While Latin American countries are taking initial steps to adapt health systems, improve cities, and reduce vulnerabilities to climate change, progress remains slow, varied, and unequal. Only seven countries have recent Vulnerability & Adaptation assessments, and nine have developed Health National Adaptation Plans since 2020. Limited action, stemming from slow planning, is evident in the region's shrinking urban green spaces and use of fossil fuels as the predominant fuel for transport, with only 0.04% of electric vehicles in the region. Consequently, the primary adaptation and mitigation strategy for heat and ambient air pollution, which are largely caused by transportation in Latin American cities, are progressing slowly. This limited progress is contributing to a significant portion of the 360,000 premature deaths attributed to fossil fuel-related PM_2.5_. Therefore, an equitable transition towards genuinely clean transport solutions, including expanded public transport networks and safe infrastructure for active travel, is essential.

Compounding this, self-reported high health emergency preparedness has decreased since 2022—a significant concern for dengue-vulnerable nations like Bolivia, Peru, and Brazil. Increases in city risk assessments and use of early warning systems reflect important progress. However, these efforts are insufficient for widespread impact. The limited climate change and health training for health professionals –medical and public health students–further widens the gap in building future resilience. Furthermore, Latin American countries bear a significant responsibility in stewarding a critical element of climate change resilience, the Amazon rainforest, one of the planet's richest terrestrial biomes. In 2023 alone, the Latin America region lost 5.16 million hectares of tree cover, primarily due to commodity-driven deforestation, shifting agriculture, and forestry. The highest losses were in Brazil, Bolivia, and Peru. To effectively reverse this trend and achieve better conservation outcomes, it is crucial to leverage the current discussions within the Belem Health Action Plan and other climate and health policy processes. This requires elevating the voices of Indigenous leaders, who can provide perspective on the disproportionate pressures their communities face from encroachment, which not only threatens their way of life but also contributes to the loss of their profound connection to the forest.

Latin America does not have the luxury of waiting for global political will and must push ahead with national actions. It needs to move from promises to action, taking the lead in protecting health through tailored adaptation and mitigating strategies. Geopolitical tension and shifting donor priorities further cloud the outlook, raising concerns that promised resources will not materialise at the pace or scale required to safeguard health. Health-centred adaptation finance remains scarce. Bilateral commitments for health-focused adaptation projects in Latin America totalled US$197 million, with Brazil receiving the largest share (US$133). Since 2017, the GCF allocated US$3.4 billion to health-related adaptation (37 projects), but only US$77.7 million directly targeted health adaptation actions. Closing this gap is essential to deliver the UAE-Framework's health objectives.

A shift away from fossil fuel dependency through the mitigation commitments outlined in each country's NDCs appears to be stagnant. Latin American countries continue to exhibit a net-negative carbon price, reflecting a substantial net fossil fuel subsidy of US$38.6 billion which outweighs carbon-pricing revenues by nearly fifty to one. This dependence on fossil fuels extends to cooking fuels, often further subsidised by local Liquefied Petroleum Gas voucher programmes.^117^ A rapid and just energy transition policy by 2030 is crucial to expand access to cleaner, renewable cooking in Latin America, thus following WHO Sustainable Development Goals 7 (SDGs-7) “Affordable and Clean Energy” recommendations. Despite Latin American countrie s’ renewable energy potential, the region continues to lag in its capacity to transition to a zero-carbon future, even reducing the average preparedness for the low–carbon transition by 2.5% in 2024.

As COP30 in Belém do Pará, Brazil, rapidly approaches, Latin American countries have the unique opportunity to champion health-prioritised climate adaptation for sustainable development and health for all and to push for rapid mitigation strategies with health co-benefits. This requires strong governance, inclusive participation from all levels of society -governments, scientists, news media, corporations, and citizens-to accelerate adaptation and mitigation efforts. The time for decisive, health centred climate action in Latin America is now.

## Contributors

The 2025 Latin America Report of the Lancet Countdown on health and climate change is an academic collaboration which builds on the work of the Lancet Countdown. The work of this paper follows the global structure of five working groups, which were responsible for the design, drafting, and review of their individual indicators and sections. All authors contributed to the overall paper structure and concepts and provided input and expertise to their relevant sections.

Authors contributing to:

• Section 1: Andres G. Lescano, Luis E Escobar, Rayana Santos Araujo Palharini, Yasna K Palmeiro-Silva, Nicolas Borchers-Arriagada.
• Section 2: Mónica Pinilla-Roncancio, Avriel Diaz, Camila Llerena-Cayo, Yasna K Palmeiro-Silva, Francisco Chesini, Nelson Gouveia, Mauricio Santos-Vega, Juan D Umaña, Zaray Miranda-Chacon, Cecilia Sorensen.
• Section 3: David Rojas Rueda, Nicolas Valdes-Ortega, Stella M. Hartinger, Raquel Santiago, Tatiana Souza de Camargo, Aline Martins de Carvalho, Nahid Mohajeri, Enzo Sauma, Luciana Blanco-Villafuerte, Magali Hurtado, Carole Dalin, Harry Kennerd.
• Section 4: Oscar Melo, Juliana Helo Sarmiento, Christian García-Witulski, Chrissie Pantoja.
• Section 5: Bruno Takahashi, Carolina Gil Posse, Milena Sergeeva, Maria Fernanda Salas, Yasna K Palmeiro-Silva, Luciana Blanco-Villafuerte, Tim Repke.
• Panels: Stella M. Hartinger, Yasna K Palmeiro-Silva, Marcia Chame, Marcelo Firpo Porto, Luiza Ribeiro Alves Cunha, Antonella Risso, Juliana W. Rulli Villardi, Eliane Lima, Renata Gracie, Rômulo Paes-Sousa.
• Provided critical review and constructive input: Daniel Buss, Antonella Risso, Marina Romanello, Sol Savila, Armando Valdés-Velásquez, Matilde Rusticucci, Alejandro Saez, Francisco Estrada, Stella M. Hartinger and Maria Walawender.

The conceptualisation, coordination, strategic direction, and editorial support for this paper was provided by: Stella M. Hartinger, Yasna K Palmeiro-Silva, Luciana Blanco-Villafuerte, Camila Llerena-Cayo, Marina Romanello.

## Data sharing statement

Data will be made available from the corresponding author upon reasonable request.

## Editor note

The Lancet Group takes a neutral position with respect to territorial claims in published maps and institutional affiliations.

## Declaration of interests

SH, YKPS, LBV, CLC, RSAP, CGW, MFS, NVO, MR, MW were supported by the Wellcome Trust (Grant Ref: 304972/Z/23/Z). CLC and LBV are also supported by Regional GEO health Hub Centered In Peru GRANT11785319. MR is also supported by the Horizon Europe programme through the projects IDAlert (Grant no. 101057554) and UK Research and Innovation project (ref no. 10056533); and CATALYSE (grant no. 101057131) and UK Research and Innovation project (ref no. 10041512). CATALYSE and IDAlert are part of the EU climate change and health cluster (https://climate-health.eu). CGW was supported by Pontificia Universidad Católica Argentina (UCA) and MPR by Universidad de Los Andes. AGL is sponsored by Emerge, the Emerging Diseases Epidemiology Research Training grant D43 TW007393 awarded by the Fogarty International Center of the US National Institutes of Health. EL, JW and LRAC were supported by Oswaldo Cruz Foundation. LEE was supported by National Science Foundation CAREER (2235295) and HEGS (2116748) awards, and Virginia Tech DA PPP, CeZAP, and ICTAS grants. AR Declares Consulting fees from World Health Organization, the Pan American Health Organization and the Universidad de Antioquia. Research reported in this publication was supported by the National Institute of Allergy and Infectious Diseases of the National Institutes of Health under Award Number K01AI168452. The content is solely the responsibility of the authors and does not necessarily represent the official views of the National institutes of Health. DB is a staff member of the Pan American Health Organization (PAHO). The author alone is responsible for the views expressed in this publication, and they do not necessarily represent the decisions or policies of PAHO. All other authors declare no competing interests.

## Appendix A Supplementary data

Appendix

Executive Summary in Spanish

Executive Summary in Portuguese
