# Supplementary material for: The 2025 Lancet Countdown Latin America report: moving from promises to equitable climate action for a prosperous future
Source: Lancet Reg Health Am. 2025 Oct 29;52:101276. doi: 10.1016/j.lana.2025.101276 (PMC12801031; doi:10.1016/j.lana.2025.101276)
Supplement: Appendix [file mmc1.pdf]

Appendix
Methods and additional analyses

The 2025 report of The *Lancet* Countdown Latin America on health and climate change

This appendix provides detailed information on the indicators presented in the main report, including methodological details and additional analyses.

The countries included in this report are:

- Central America: Costa Rica, El Salvador, Guatemala, Honduras, Mexico, Nicaragua, Panama.
- South America: Argentina, Bolivia, Brazil, Colombia, Chile, Ecuador, Paraguay, Peru, Uruguay, Venezuela.

Given the data availability in some countries, not all of them are included in all the indicators, which is stated in each indicator below.

Contents

Section 1: health hazards, exposures, and impacts 4
1.1.1: exposure to warming 4
1.1.2: exposure of vulnerable populations to heatwaves 4
1.1.3: heat and physical activity 5
1.1.4: heat-related mortality 6
1.2.1: wildfires 6
1.2.2: droughts 12
1.3.1: dengue 16
1.3.2: Vibrios 16
Section 2: adaptation, planning, and resilience for health 19
2.1.1: national assessments of climate change impacts, vulnerability, and adaptation for health 19
2.1.2: national adaptation plans for health 19
2.1.3: city-level climate change risk assessments 20
2.2.1: climate information for health 25
2.2.2: benefits and harms of air conditioning 27
2.2.3: urban greenspace 28
2.2.4: detection, preparedness, and response to health emergencies 38
2.2.5: climate and health education and training 39
2.3.1: risk to severe mosquito-borne diseases 43
2.3.2: lethality of extreme weather events 44
Section 3: mitigation actions and health co-benefits 45
3.1.1: energy system and health 45
3.1.2: household energy use 46
3.1.3: sustainable and healthy road transport 48
3.2.1: premature mortality from ambient air pollution 49
3.2.2: exposure to household air pollution 52
3.3.1: emissions from agricultural production and consumption 52
3.3.2: diet and health co-benefits 57
3.4: tree cover loss and health 58
3.5: healthcare sector emissions 59
Section 4: economics and finance 60
4.1.1: Economic losses due to weather-related extreme events 60
4.1.2: Costs of heat-related mortality 61
4.1.3: Loss of earnings from heat-related labour capacity reduction 61

4.1.4: costs of the health impacts of air pollution ... 62
4.2.1: country preparedness for the transition to net zero ... 62
4.3.1: Net value of fossil fuel subsidies and carbon prices ... 63
4.3.2: Health adaptation finance flows and disclosed needs ... 64
Section 5: public and political engagement ... 65
5.1: media engagement with health and climate change ... 65
5.2: social media engagement with health and climate change ... 71
5.4.1: government commitment with health and climate change in UNGA & NDCs ... 74
5.4.2: funding for science on health and climate in Latin America ... 74
5.5: corporate sector engagement with health and climate change ... 78
References ... 79

Section 1: health hazards, exposures, and impacts

1.1: health and heat

1.1.1: exposure to warming

Regional author(s)
Yasna Palmeiro Silva

Methods

This indicator calculates annual population-weighted ambient temperature (to represent population exposure to heat) and temperature anomalies, using 2000-2024 data from ERA5-Land and the Global Human Settlement Layer (GHSL).

Additional analysis

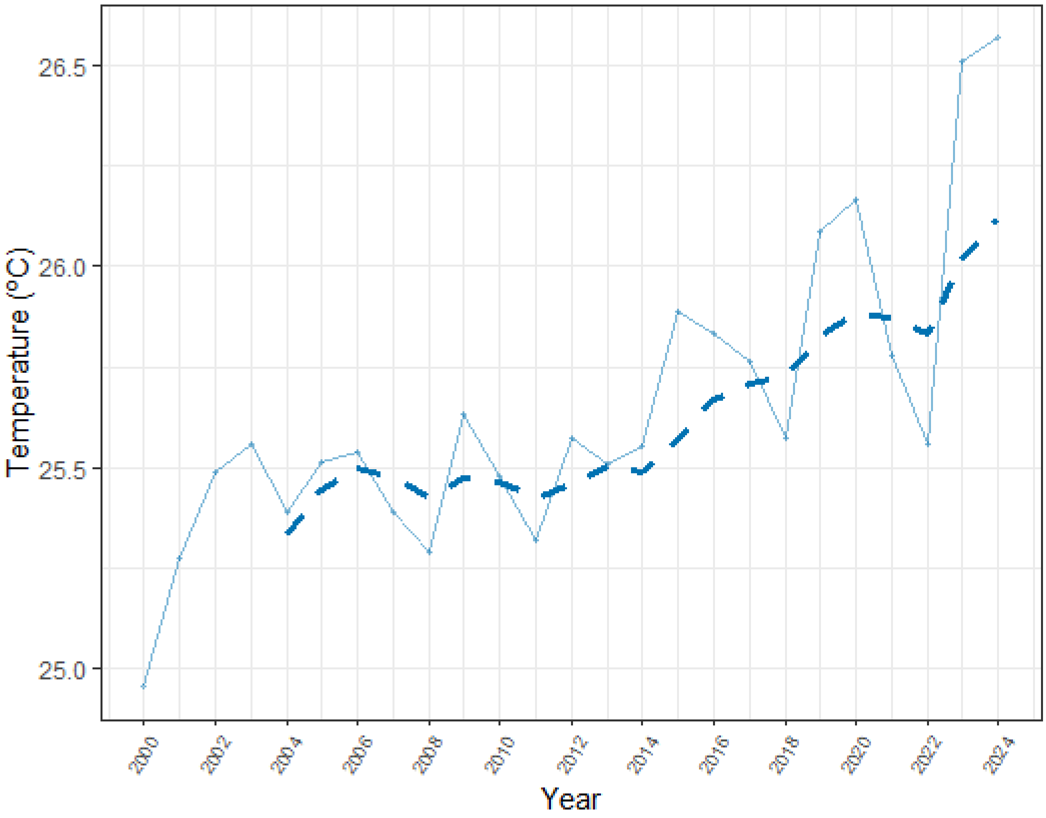

Figure A1.1. Annual mean temperature (ºC) in Latin America from 2000 to 2024, using simple average across countries. Dashed line represents 5-year moving average.

Future form of the indicator

Future iterations will update the baseline years and include subnational analyses.

1.1.2: exposure of vulnerable populations to heatwaves

Regional author(s)
Yasna Palmeiro Silva

Methods

This indicator follows the same methodology as the 2025 global *Lancet* Countdown report.

Additional analysis

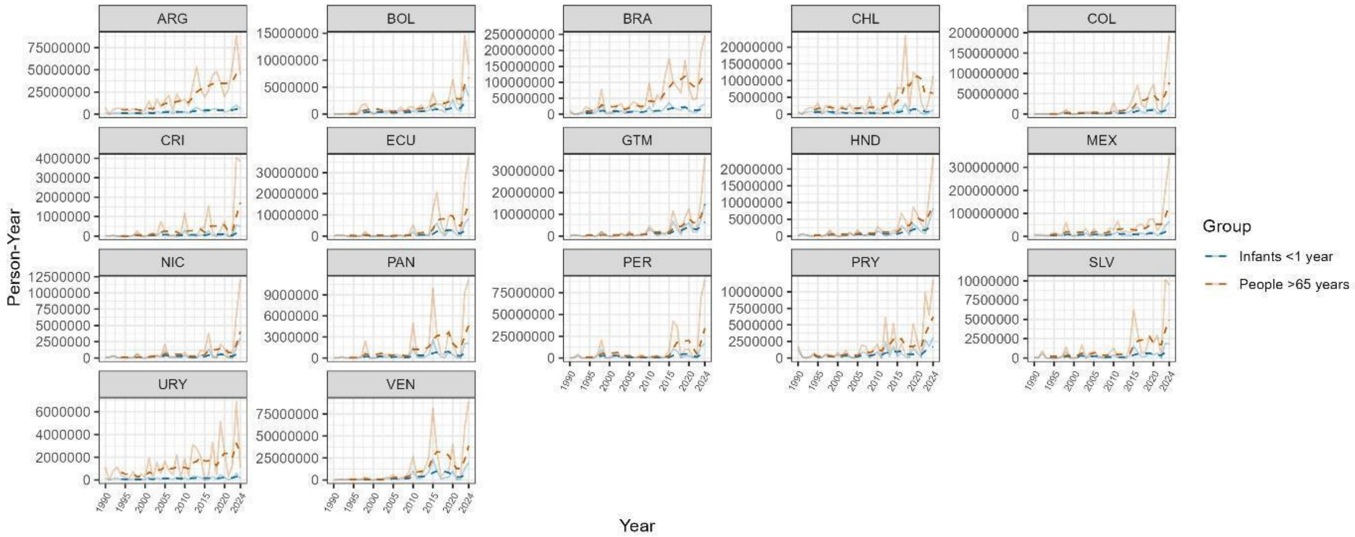

Figure A1.2. Total annual person-days of heatwave experienced by people over 65 and infants under 1 year old by country. Dotted lines represent 5-year simple moving average.

Future form of the indicator

Future iterations will update the baseline years to and include subnational analyses.

1.1.3: heat and physical activity

Regional author(s)

Yasna Palmeiro Silva

Methods

This indicator follows the same methodology as the 2025 global *Lancet* Countdown report.

Additional analysis

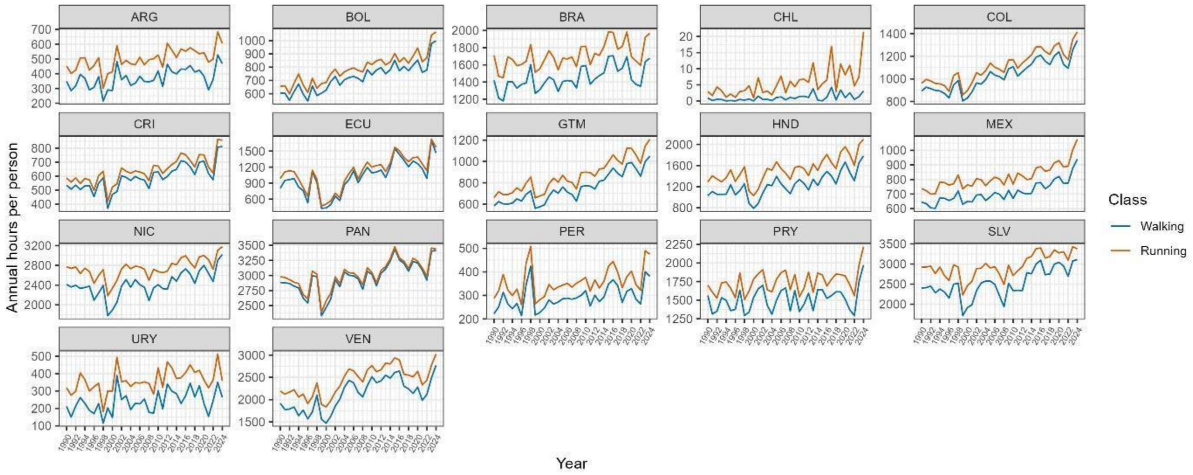

Figure A1.3. Average annual hours per person that physical activity (walking and running) entailed at least a moderate heat stress risk from 1990 to 2024. Walking (blue) and running (orange).

1.1.4: heat-related mortality

Regional author(s)

Yasna Palmeiro Silva

Methods

This indicator follows the same methodology as the 2025 global *Lancet* Countdown report.

Additional analysis

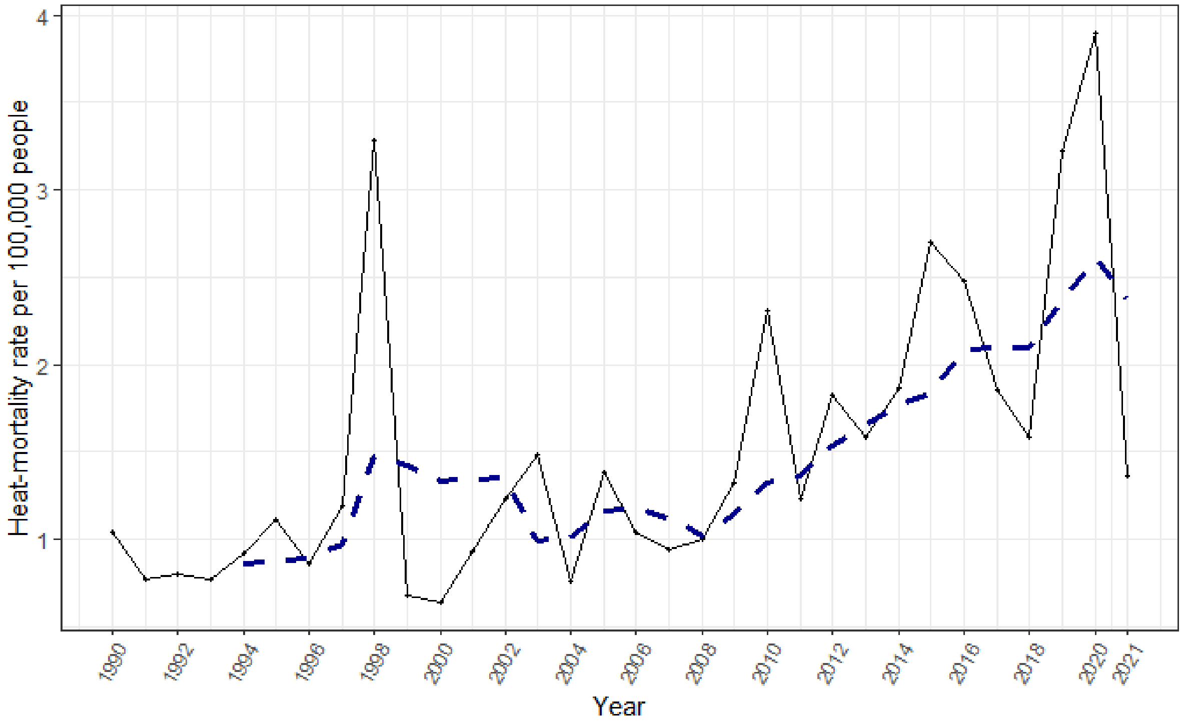

Figure A1.4. Estimated heat-related mortality rate per 100,000 people, in Latin America, from 1990 to 2021. Dashed line represents 5-year moving average.

1.2: health and extreme weather-related events

1.2.1: wildfires

Regional author(s)

Nicolas Borchers Arriagada

Methods

This indicator presents three sub-indicators:

1. Number of days people were exposed to very high or extremely high fire danger risk (fire danger risk),
2. Number of person-days people were exposed to wildfires (exposure to wildfires), and
3. Exposure to wildfire-$PM_{2.5}$ (wildfire smoke).

All sub-indicators follow the same methodology as the 2025 global *Lancet* Countdown report. The exposure to wildfires sub-indicator was derived using data from the global report. The fire danger risk and wildfire smoke sub-indicators had slight variations to achieve results at a sub-national level.

For the fire danger risk sub-indicator, we derived population density data from the NASA SEDAC Gridded Population of the World version 4 (GPWv4) at a 1km x 1km spatial resolution. We estimated annual population grids for 2003 to 2024 by interpolating available population density maps for the years 2000, 2005, 2010, 2015, and 2020. The annual mean number of days exposed to very high or extremely high fire danger risk (fire danger index ≥ 38, original spatial resolution of 0.25° x 0.25°)[1] were resampled at a 1km x 1km spatial grid, using bilinear interpolation. We then extracted the annual population-weighted number of days exposed to very high or extremely high fire danger risk at an administrative level 1 (the largest sub-national administrative unit within a country) for each country. Grid cells with a population density ≥ 400 people per square kilometre were omitted from the analysis.

In the case of the wildfire smoke sub-indicator, we followed a similar approach. We resampled the annual mean wildfire-related $PM_{2.5}$ [2] from its original spatial resolution of 0.2° x 0.2° to a 1km x 1km spatial grid, coinciding with the population density data. Then, we extracted the population-weighted annual exposure to wildfire-$PM_{2.5}$ at an administrative level 1 for each country.

Additional analysis

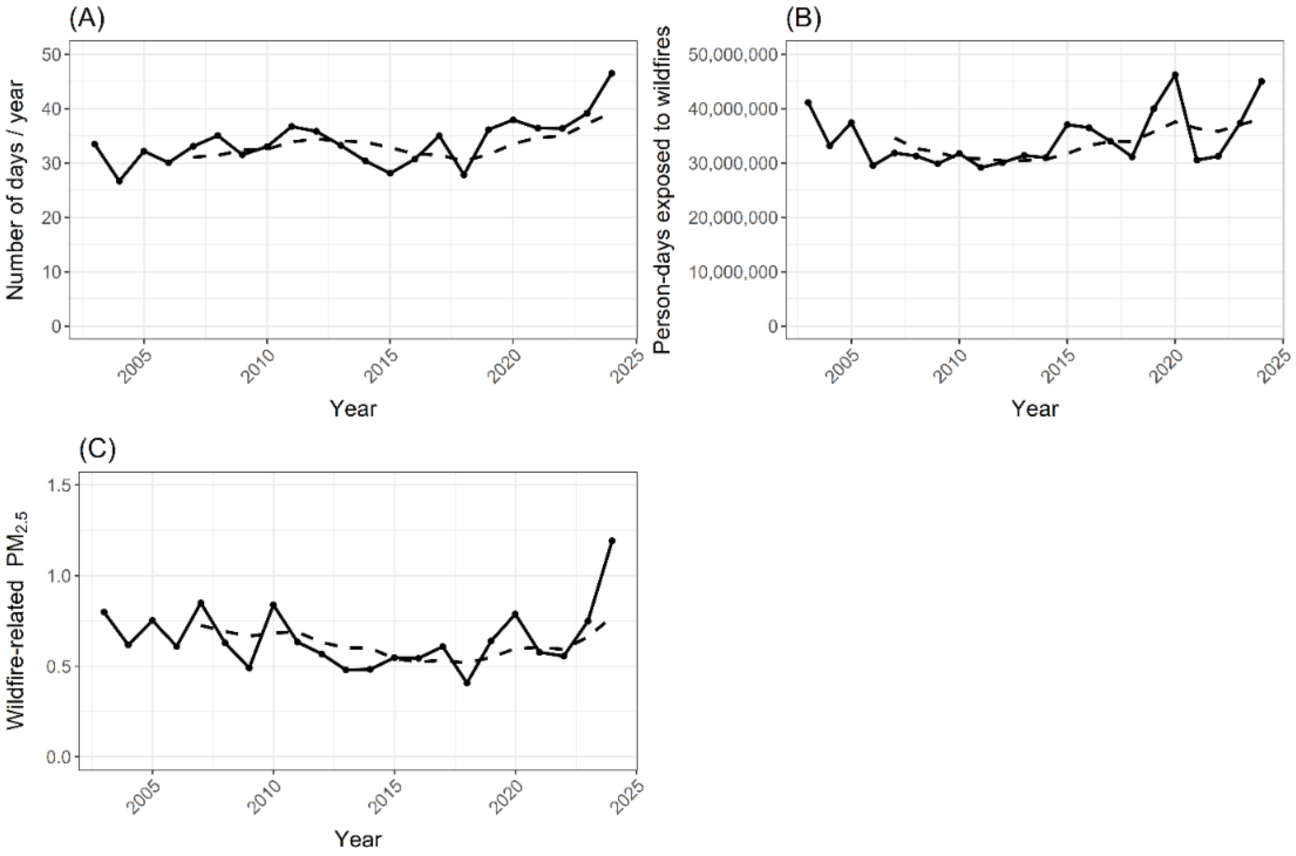

Figure A1.5. Wildfire indicators for 2003-2024: (A) annual number of days people were exposed to very high or extremely high fire danger risk, (B) annual mean number of person-days exposed to wildfires, and (C) annual exposure to wildfire-$PM_{2.5}$. Dotted lines represent the 5-year simple moving average.

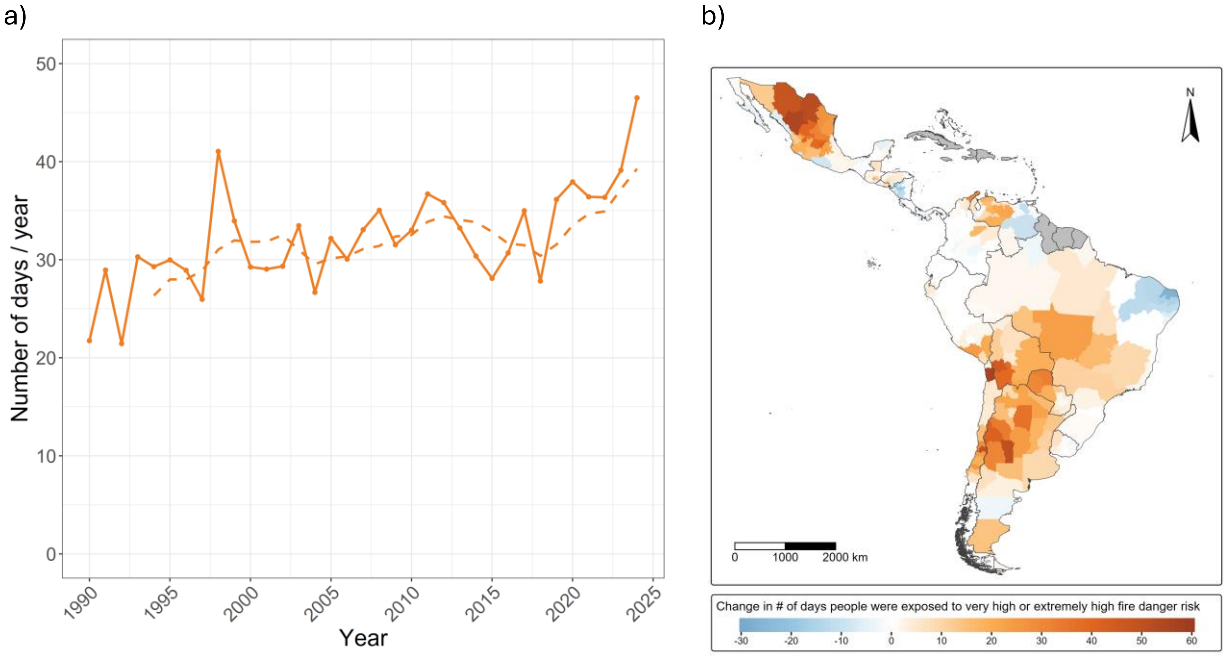

Figure A1.6. Annual number of days people were exposed to very high or extremely high fire danger risk in Latin America: (A) population-weighted mean across all 17 countries (dotted lines represent the 5-year simple moving average), and (B) change in # of days between 2020-2024 (comparison period) and 2003-2007 (baseline), by sub-national level. Positive values reddish colours and negative values blueish colours.

Table A1.1. Summary of annual number of days people were exposed to very high or extremely high fire danger risk in Latin America, by country.

Country	Baseline (2003-2007)	Comparison period (2020-2024)	Change	
	# of days/year	*# of days/year*	*# of days/year*	%

Chile	29.1	59.5	30.5	105.0%
Mexico	61.8	79.5	17.6	28.5%
Bolivia	20.2	36.9	16.7	82.6%
Argentina	19.7	34.6	14.9	75.7%
Guatemala	10.3	17.3	7.0	67.8%
Venezuela	20.1	25.3	5.1	25.4%
Paraguay	7.0	12.0	5.0	70.6%
Peru	6.5	10.5	3.9	60.7%
Brazil	32.3	35.7	3.4	10.6%
Honduras	18.0	20.1	2.1	11.6%
Colombia	4.2	6.1	1.9	44.5%
Uruguay	3.1	4.8	1.7	53.3%
El Salvador	47.8	49.2	1.4	2.9%
Costa Rica	4.7	5.0	0.3	6.5%
Ecuador	0.1	0.2	0.2	295.0%
Panama	6.3	4.2	-2.1	-33.4%
Nicaragua	71.2	56.5	-14.7	-20.7%
**Total**	**31.1**	**39.3**	**8.2**	**26.4%**

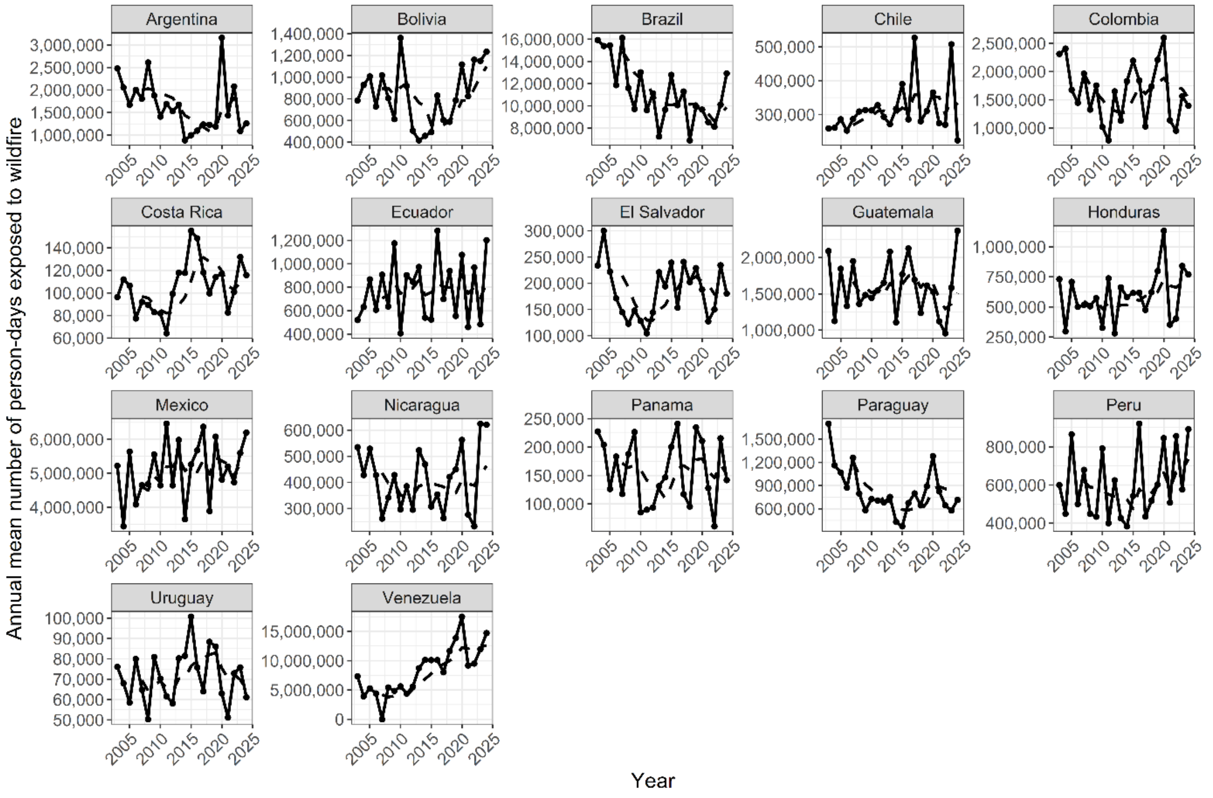

Figure A1.7. Annual mean number of person-days exposed to wildfires by country: 2003-2024. Dotted lines represent the 5-year simple moving average.

Table A1.2. Summary of annual mean number of person-days exposed to wildfires in Latin America, by country.

Country	Baseline (2003-2007)	Comparison period (2020-2024)	Change	
	# of person-days/year	*# of person-days/year*	*# of person-days/year*	**%**

Venezuela	4,189,309	12,576,340	8,387,030	200.2%
Mexico	4,606,638	5,309,256	702,618	15.3%
Bolivia	892,936	1,096,985	204,049	22.9%
Honduras	550,159	699,439	149,280	27.1%
Ecuador	706,490	837,905	131,415	18.6%
Peru	618,710	735,228	116,517	18.8%
Chile	268,972	327,940	58,967	21.9%
Nicaragua	436,278	463,301	27,023	6.2%
Costa Rica	96,862	109,651	12,790	13.2%
Uruguay	69,463	64,799	-4,664	-6.7%
Panama	171,772	151,349	-20,423	-11.9%
El Salvador	214,351	175,901	-38,450	-17.9%
Guatemala	1,669,103	1,513,002	-156,101	-9.4%
Argentina	2,003,287	1,804,434	-198,853	-9.9%
Paraguay	1,212,760	809,139	-403,621	-33.3%
Colombia	1,961,189	1,527,961	-433,228	-22.1%
Brazil	14,948,939	9,868,524	-5,080,415	-34.0%
**Total**	**34,617,221**	**38,071,154**	**3,453,933**	**10%**

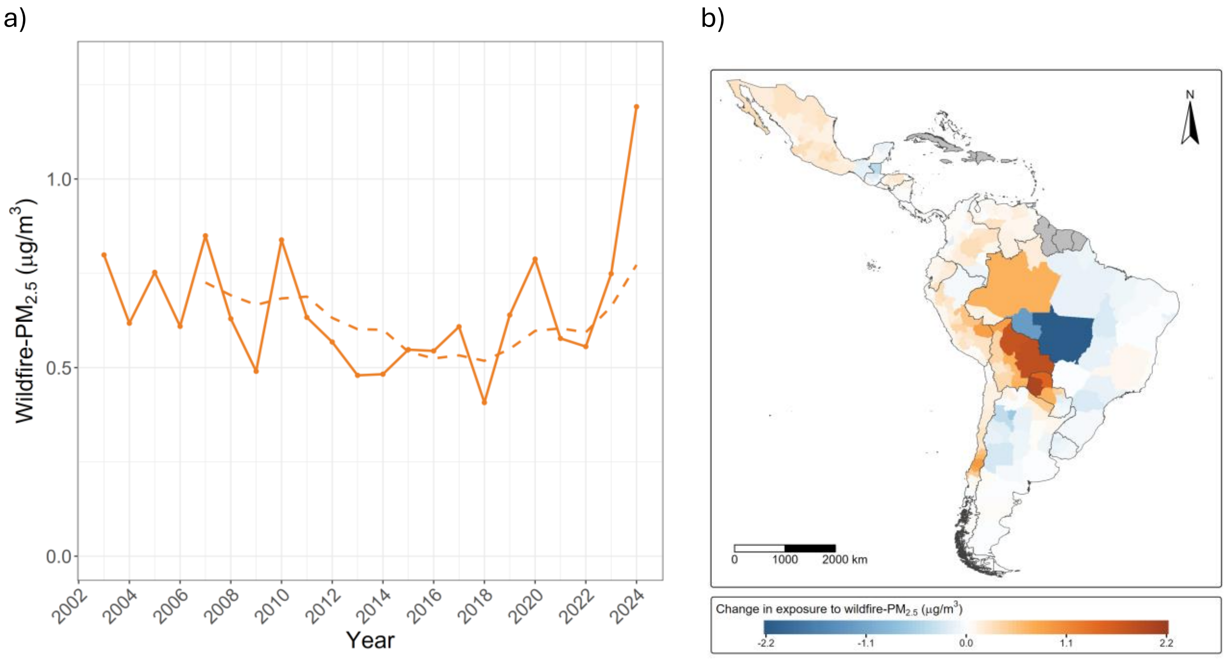

Figure A1.8. Annual exposure to wildfire-related $PM_{2.5}$ (μg/m^3) in Latin America: (A) population-weighted mean across all 17 countries (dotted lines represent the 5-year simple moving average), and (B) change in exposure (μg/m^3) between 2020-2024 (comparison period) and 2003-2007 (baseline), by sub-national level. Positive values reddish colours and negative values blueish colours.

Table A1.3. Summary of annual exposure to wildfire-related $PM_{2.5}$ (μg/m^3) in Latin America, by country.

Country	Baseline (2003-2007)	Comparison period (2020-2024)	Change	
	(μg/m^3)	*(μg/m^3)*	*(μg/m^3)*	**%**

Bolivia	2.01	3.09	1.08	53.7%
Chile	0.49	0.89	0.40	81.0%
Mexico	0.64	0.81	0.17	26.9%
Peru	0.30	0.42	0.12	41.0%
Honduras	0.45	0.56	0.11	23.2%
Ecuador	0.19	0.26	0.07	38.6%
Venezuela	0.40	0.46	0.07	16.3%
Colombia	0.33	0.38	0.05	14.7%
El Salvador	0.34	0.39	0.04	12.9%
Paraguay	2.64	2.67	0.04	1.3%
Nicaragua	0.18	0.20	0.02	11.0%
Panama	0.08	0.09	0.01	14.3%
Costa Rica	0.03	0.03	0.00	0.7%
Uruguay	0.47	0.40	-0.08	-16.0%
Brazil	0.92	0.84	-0.08	-8.5%
Argentina	1.04	0.95	-0.09	-8.8%
Guatemala	0.73	0.62	-0.11	-14.9%
**Total**	**0.726**	**0.774**	**0.0484**	**6.7%**

1.2.2: droughts

Regional author(s)
Rayana S. Araujo Palharini

Methods

This indicator follows the same methodology as the 2025 global *Lancet* Countdown report. This indicator, new to the *Lancet* Countdown Latin America report, draws on the 2025 global Lancet Countdown report, and uses the Standardised Precipitation Evapotranspiration Index (SPEI) to monitor extreme drought intensity and duration across all land areas.[20] An extreme drought is defined as a prolonged period of moisture deficit, where the SPEI value is lower than -2.00.

Additional analysis

Sub-national analysis reveals highly heterogeneous changes in Latin American land area experiencing short droughts (≥1 month). Regions in Bolivia, Brazil, Mexico, and Peru saw over 70% increases. These dramatic increases are linked to severe consequences: crop damages and losses have caused the Acute Food Insecurity to rise to crisis level (i.e. IPC Phase 3) in most of Central America.[3–5]Droughts are affecting indigenous and local communities' with subsistence agriculture livelihoods, diminishing their recovery capacity and resilience to future extreme climate events. Navigation is also affected, including in the Panama Canal and in the port of Manaus that reached a record low of 12.7 m of water levels in 2024, the lowest since 1902.[6,7]

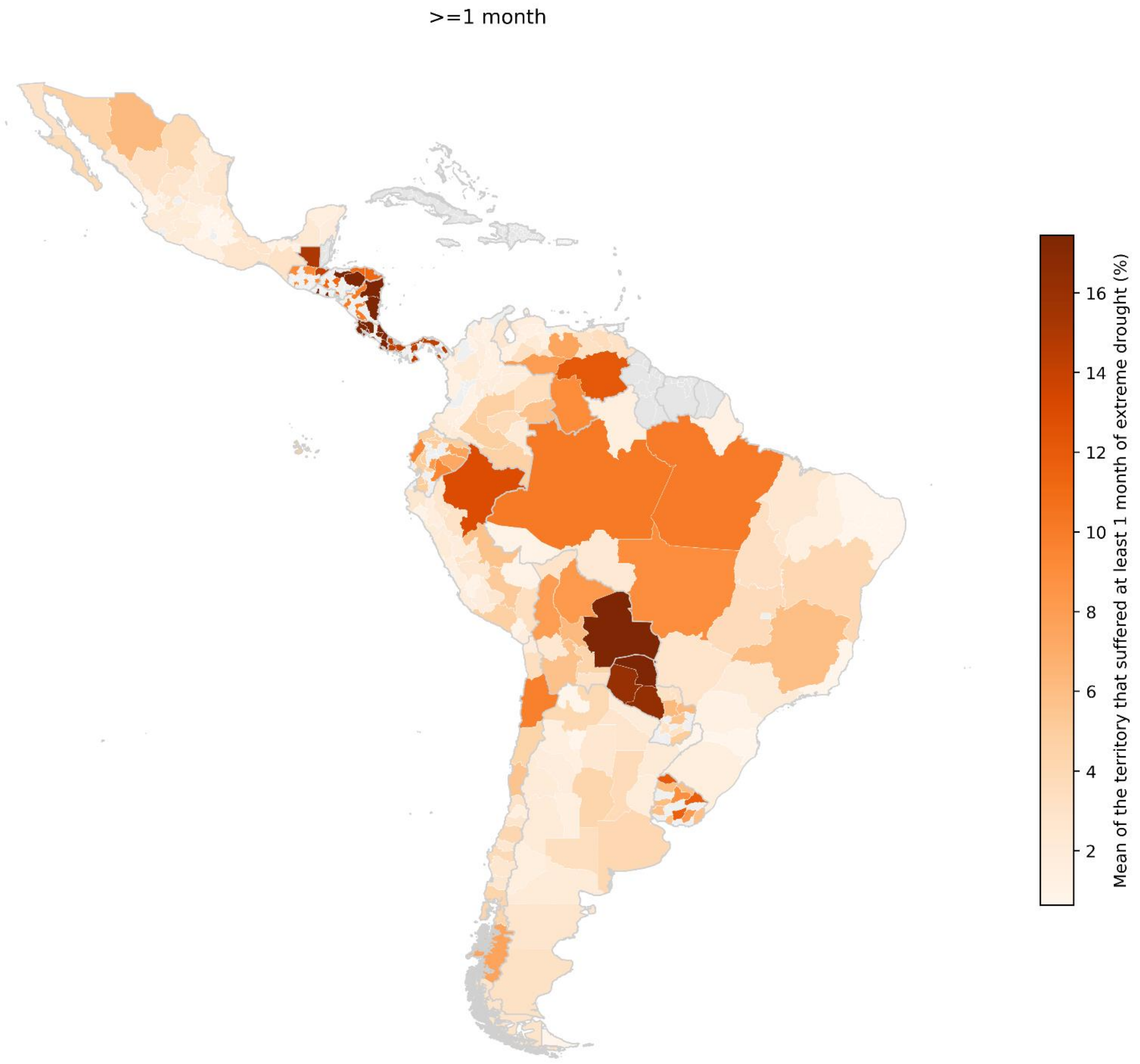

Figure A1.9. Mean of the territory that suffered at least ≥1 month of extreme drought, considering recent period between 2015-2024. Bigger values dark in orange colours and lower values in light orange colours.

>=3 months

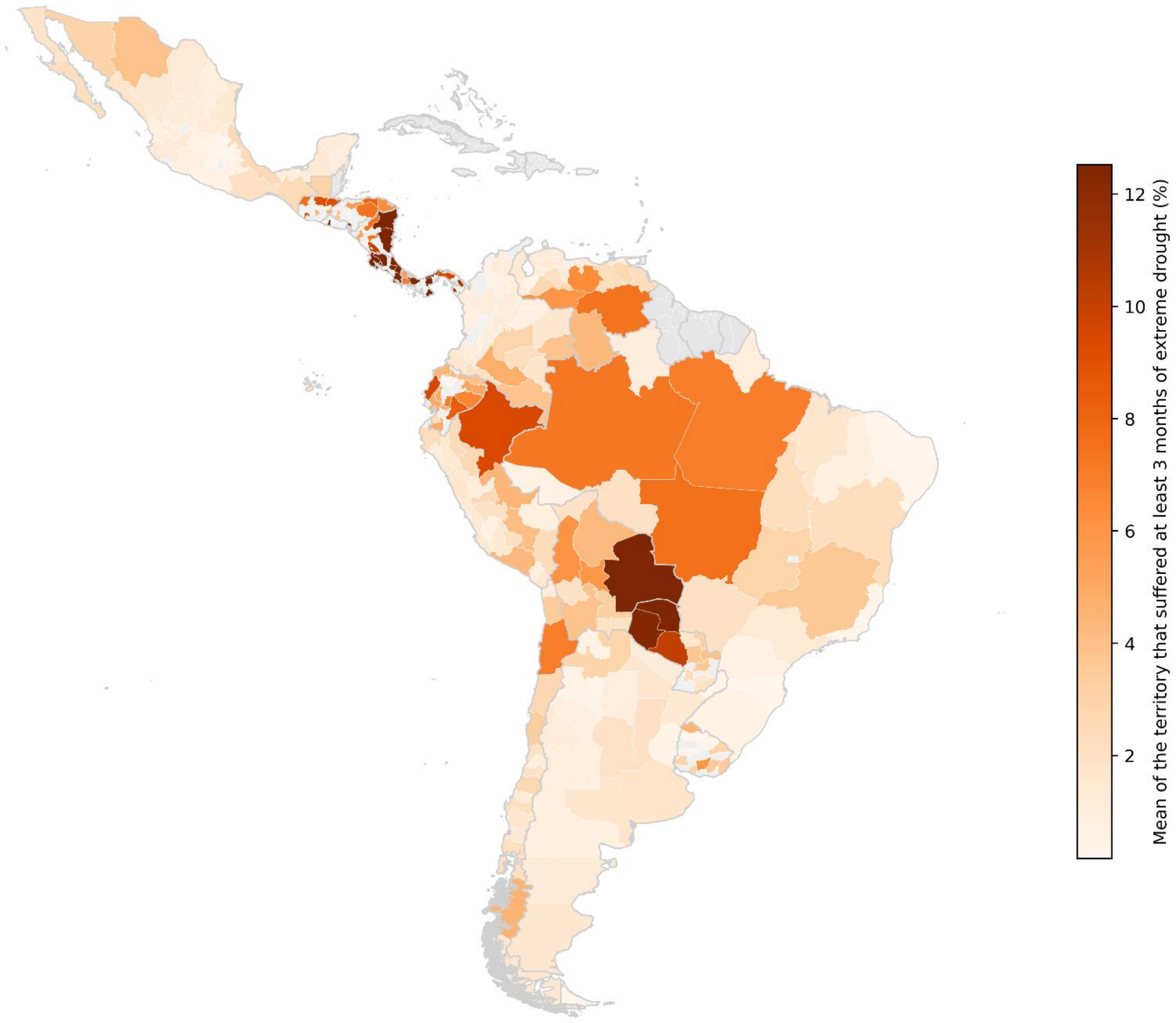

Figure A1.10. Mean of the territory that suffered at least ≥3 month of extreme drought, considering recent period between 2015-2024. Bigger values dark in orange colours and lower values in light orange colours.

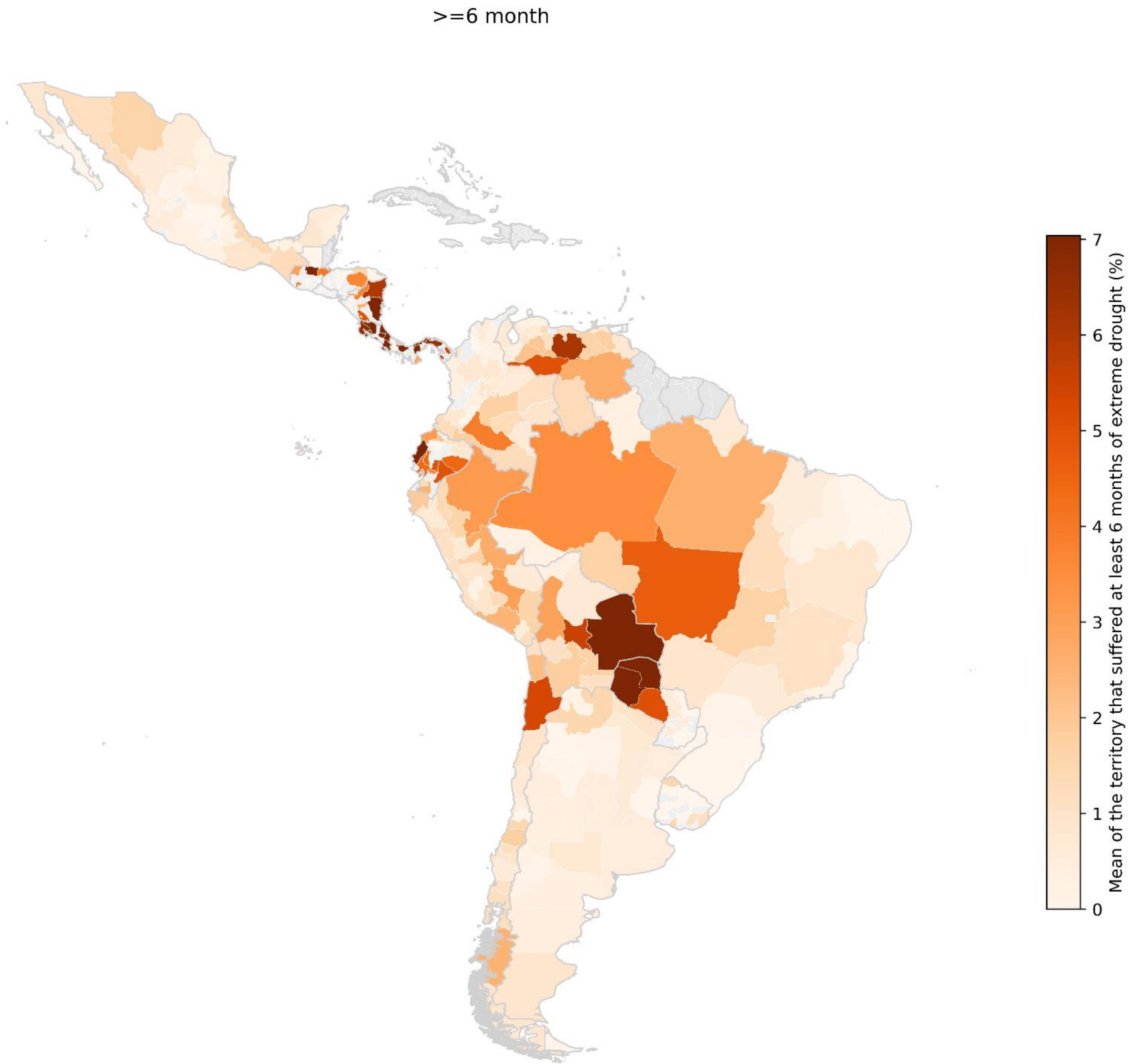

Figure A1.11. Mean of the territory that suffered at least ≥6 month of extreme drought, considering recent period between 2015-2024. Bigger values dark in orange colours and lower values in light orange colours.

1.3: climate suitability for infectious disease transmission

1.3.1: dengue

Regional author(s)

Andres G. Lescano

Methods

This indicator follows the same methodology as the 2025 global *Lancet* Countdown report.

Additional analysis

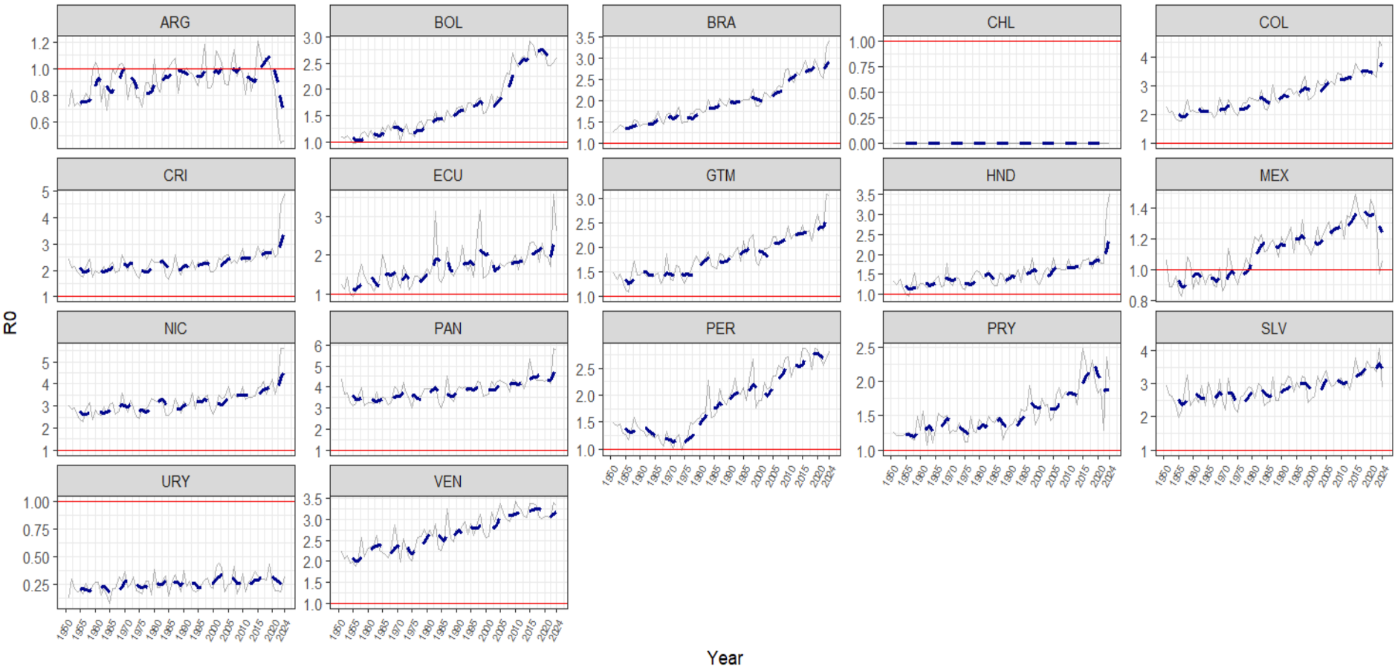

Figure A1.13. Annual estimated reproduction number for *Aedes aegypti* by Latin American country from 1950 to 2024. Dotted line represents 5-year simple moving average. Red line represents (R0) = 1, where there was no potential for an epidemic.

1.3.2: Vibrios

Regional author(s)

Luis Escobar

Methods

This indicator follows the same methodology as the 2025 global *Lancet* Countdown report.

Additional analysis

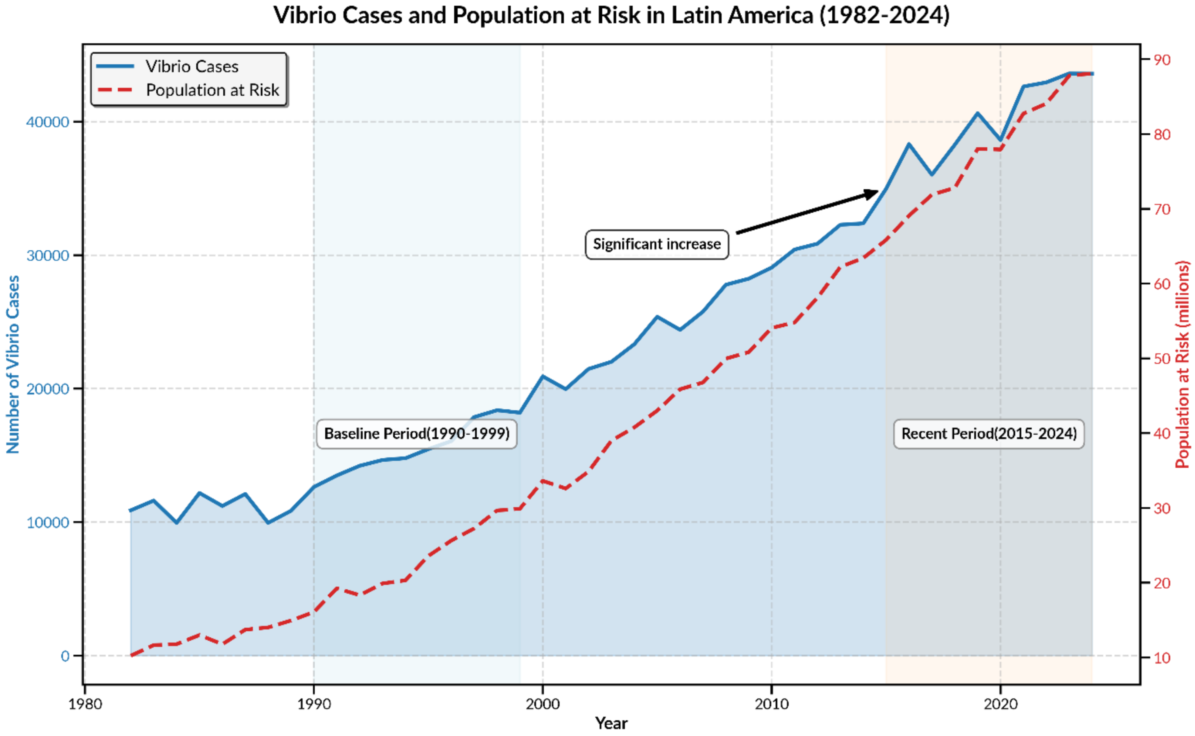

Figure A1.14. Evolution of Vibrio cases (blue line) and population risk (red line) in Latin America from 1982 to 2024, demonstrating a significant increase in recent years.

Table A1.4. Comparation between baseline period and recent period of Vibrio in Latin America

Metric	Baseline (1990–1999)	Recent (2015–2024)	% change
Coastline_km²	5 574 755 km²	5 948 286 km²	6.70 %
Population_at_risk	50 949 824 people	102 781 673 people	101.73 %
Cases	21 851 cases	44 085 cases	101.75 %

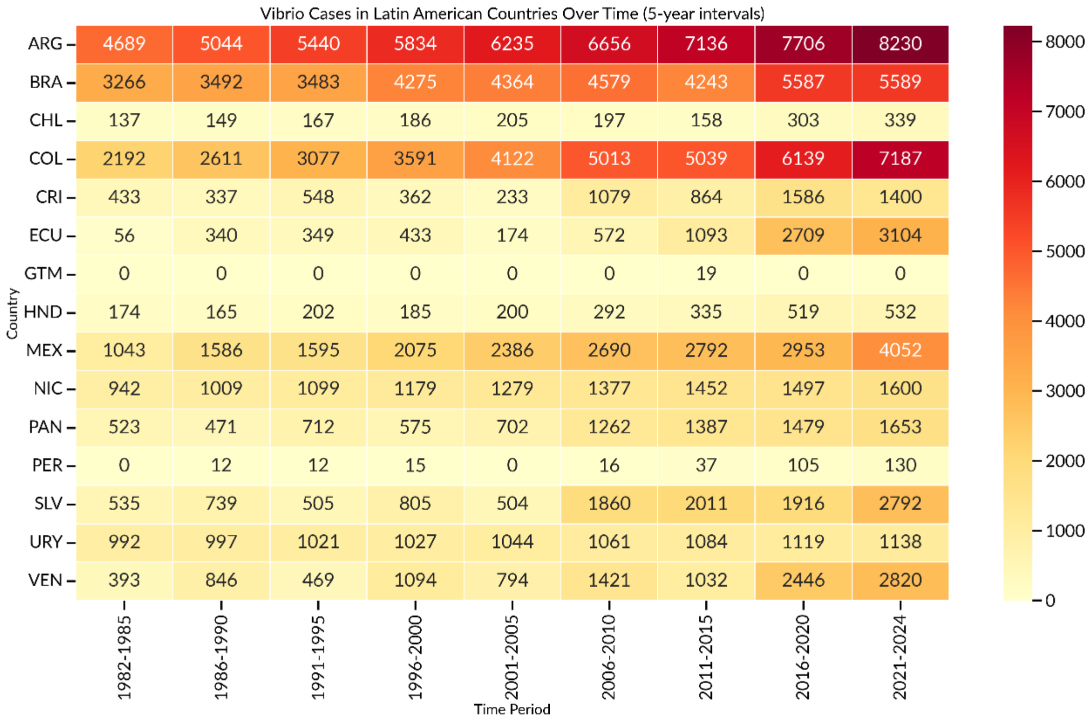

Figure A1.15. Represents the time series every 5 years of the number of vibrio cases for each country in Latam, considering that more reddish registered more cases and more yellowish registered fewer cases of vibrio.

Section 2: adaptation, planning, and resilience for health

2.1: assessment and planning of health adaptation

2.1.1: national assessments of climate change impacts, vulnerability, and adaptation for health

Regional author(s)
Zaray Miranda Chacon, Camila Llerena

Methods

This indicator follows the same methodology as the 2025 global Lancet Countdown report. We report information on Argentina, Bolivia, Brazil, Chile, Colombia, Ecuador, El Salvador, Guatemala, Nicaragua, Panama, Paraguay and Peru. No information was available for Honduras, Mexico, Uruguay and Venezuela because they did not respond to the PAHO or WHO surveys.

Additional analysis

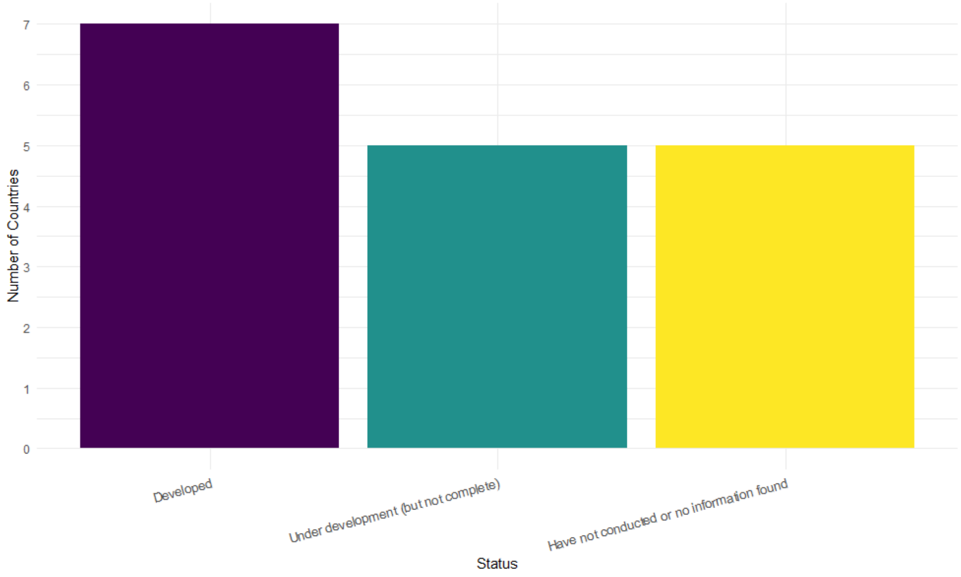

Figure A2.1. Status of vulnerability and adaptation (V&A) assessments in Latin America, based on the most recent information available from WHO, PAHO, and national reports.

Caveats and limitations

The main limitation of this indicator is its reliance on self-reported data from countries, which may introduce inaccuracies due to misinterpretation or inconsistent reporting. Additionally, participation in the survey was voluntary, potentially leading to gaps in information. Despite this, the inclusion of 12 out of 17 countries provides substantial regional coverage, offering valuable insights.

2.1.2: national adaptation plans for health

Regional author(s)
Zaray Miranda Chacon, Camila Llerena

Methods

This indicator follows the same methodology as the 2025 global Lancet Countdown report. We report information on Argentina, Bolivia, Brazil, Chile, Colombia, Ecuador, El Salvador, Guatemala, Nicaragua, Panama, Paraguay and Peru. No information was available for Honduras, Mexico, Uruguay and Venezuela because they did not respond to the PAHO or WHO surveys.

Additional analysis

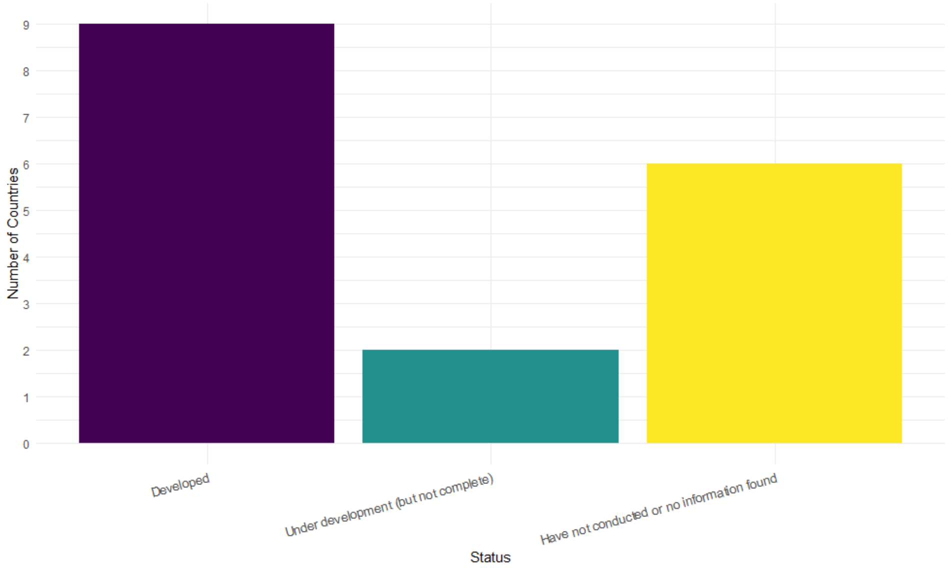

Figure A2.2. Status of HANP development in Latin America, based on the most recent information available from WHO and PAHO.

Caveats and limitations

The main limitation of this indicator is its reliance on self-reported data from countries, which may introduce inaccuracies due to misinterpretation or inconsistent reporting. Additionally, participation in the survey was voluntary, potentially leading to gaps in information. Despite this, the inclusion of 12 out of 17 countries provides substantial regional coverage, offering valuable insights.

2.1.3: city-level climate change risk assessments

Regional author(s)

Camila Llerena

Methods

The data come from the 2023 CDP Annual Cities Survey that provides self-reported data for risk assessments from 321 jurisdictions in Latin America, corresponding to 14 countries. The list of municipalities/jurisdictions per country that participated in the CDP 2023 survey or that have a plan uploaded in the Global Covenant of Mayors are presented in Table A2.1. No jurisdictions from Nicaragua, El Salvador or Bolivia participated in the survey or in the Global Covenant of Mayors.

Table A2.1. Country, number (N) and name of municipalities/jurisdictions participating in the 2023 CDP survey.[8]

Country	N	Municipalities/Jurisdictions (1)
Argentina	36	General Lavalle; Municipality of Guaymallén; Comodoro Rivadavia; Ciudad de Mendoza; Mar del Plata; Villa Carlos Paz; Cipolletti; Lujan de Cuyo; General Ramirez; Godoy Cruz; Municipalidad San Fernando del Valle de Catamarca; Arequito; Crespo; Totoras; City of Buenos Aires; Concordia; Balcarce; Municipalidad de Avellaneda; Municipio de San Luis; San Carlos Sud; Gualeguaychú; Gobierno Municipal de la Ciudad de Paraná; Municipalidad de Puerto Esperanza; Municipalidad de Vicente López; Ramona; Ushuaia; Tres Arroyos; Municipalidad de Rosario; Municipalidad de Montecarlo; Marcos Juárez; Empedrado; Santa FÉ Ciudad; Municipio de Bella Vista; Oberá; Trenque Lauquen; Municipality of Tres de Febrero , Municipalidad de Catriel*, Municipalidad de Villa El Chocón*, Municipio de Villarino*, Municipio de Oliva*, Municipalidad de Marcos Juarez*, Municipalidad de Justiniano Posse*, Municipalidad de Alberti*,

Brazil	95	Municipality of Fortaleza; Prefeitura Municipal de Ribeirão Preto; Prefeitura de Niterói; Prefeitura de Pedreira; Prefeitura da Cidade de Armação de Búzios; Prefeitura de Sirinhaém; Prefeitura de Santa Luzia; Prefeitura de Caucaia; Prefeitura de Apuí; Prefeitura Municipal de Santos; Distrito Federal (Brasília); Municipality of Recife; City of Salvador; Prefeitura de Presidente Prudente; Prefeitura de Sorocaba; Prefeitura de São Leopoldo; Prefeitura de Francisco Morato; Prefeitura de Guanhães; Municipality of Porto Alegre; Prefeitura de Barcarena; Prefeitura de Campo Grande; Prefeitura de Rio Branco; Prefeitura de Manaus; Prefeitura de Sobral; Prefeitura de Aracaju; Prefeitura de Humaitá; Prefeitura de Rio Grande; Municipality of Campinas; Prefeitura de São Roque do Canaã; Prefeitura de Dois Irmãos; Prefeitura de Guarulhos; Prefeitura de Porto Velho; Prefeitura de Itabirito; Prefeitura de Palmas; Prefeitura Municipal de Caieiras; Prefeitura de Cáceres (Brasil); Prefeitura da Cidade de São José do Rio Preto; Prefeitura Municipal de João Pessoa; Prefeitura de Extrema; Prefeitura de Serra Talhada; Prefeitura de Jandaíra; Municipality of Curitiba; Prefeitura de Canutama; Prefeitura de Curvelo; Prefeitura de Osasco; Prefeitura de Indiaroba; Prefeitura de Cuiabá; Prefeitura de Joaçaba; Prefeitura de São Gonçalo do Rio Abaixo; Prefeitura de Maués; Prefeitura de São Cristóvão; Prefeitura Municipal de Contagem; Prefeitura de São Sepé; Prefeitura de Cruzeiro do Sul; Prefeitura Municipal de Juruena; Prefeitura do Município de Piracicaba; Municipality of Belo Horizonte; Prefeitura de Abaetetuba; Prefeitura do Rio de Janeiro; Prefeitura de Quissamã; Prefeitura de Boa Ventura; Prefeitura Municipal de Canoas; Prefeitura de Varjota; Prefeitura de Formoso do Araguaia; Prefeitura de Iúna; Prefeitura de Brejo da Madre de Deus; Prefeitura de Brasiléia; Prefeitura de São Paulo; Prefeitura de Jundiaí; Prefeitura Municipal de Boa Vista; Prefeitura de Diamantina; Prefeitura de Lorena; Prefeitura de Carinhanha; Prefeitura de Itapipoca; Prefeitura de Nova Santa Rita; Prefeitura de Boca do Acre; Prefeitura de Palmeira das Missíµes; Prefeitura Municipal de Araçatuba; Prefeitura de Piraquara; Prefeitura da Estí¢ncia Turística de Itu; Prefeitura de Tangará da Serra; Prefeitura de Louveira; Prefeitura de Santa Bárbara; Prefeitura Municipal de Barueri; Prefeitura de Florianópolis; Prefeitura de Pindamonhangaba; Prefeitura de Parauapebas; Prefeitura de Itapoá; Prefeitura de Engenheiro Coelho; Prefeitura de Tapauá; Prefeitura de Uberlí¢ndia; Prefeitura de Cordeirópolis; Prefeitura de Itatiaiuçu; Prefeitura de Pau Brasil; Prefeitura de Ibiraçú
Chile	29	Región Metropolitana de Santiago; Municipalidad de Peñalolén; Municipalidad de Maipú; San Miguel (Chile); Renca; Ilustre Municipalidad de Ancud; San Bernardo; Municipalidad de Providencia; Municipalidad Cerro Navia; Municipalidad de Concepción; Municipalidad de Colina; Municipalidad de Lo espejo; Puerto Varas; Municipalidad de Vitacura; Municipalidad de Ñuñoa; Municipality of Placilla; Lo Barnechea; Municipalidad de Santiago; Municipalidad de Independencia; San Antonio; Alcaldia de Valdivia; Municipalidad de San Fernando; Municipalidad de Temuco; Municipalidad de Quilicura; Municipality of Lautaro; San Pedro de la Paz; Municipalidad de Talca; Algarrobo; Municipalidad de Puerto Montt , Municipalidad de El Quisco*, Municipalidad de Punta Arenas*, Comuna de Vallenar*,
Colombia	37	Alcaldia de Cartago; Medellín; Alcaldia de Pasto; Alcaldia de Pereira; Sabaneta; Gámeza; Itagí¼í; Beteitiva; Alcaldía Distrital de Barranquilla; Corrales; Bogotá Distrito Capital; Municipality of Tópaga; Ebéjico; Busbanza; Alcaldia de Manizales; Envigado; El Santuario; La Estrella; Municipio de Cajicá; Santiago de Cali; Alcaldía de San José de Guaviare; Alcaldía de Yopal; Alcaldia Distrital de Cartagena de Indias; Santander de Quilichao; Mongua; Copacabana; Jardín; Municipio de Bucaramanga; Floresta; Alcaldía Distrital

		de Santa Marta; Girardota; Belmira; Alcaldía de Villavicencio; Alcaldia de Rionegro; Mongui; Alcaldia de Tenjo; Municipality of La Cumbre, Valle del Cauca, Municipio de Gameza*, Munipicio de Florencia*
Costa Rica	18	Municipalidad de Cañas; Municipalidad de La Unión; Santa Ana (Costa Rica); Municipalidad de San José; Municipalidad San Pablo de Heredia; Oreamuno; Municipalidad de Quepos; Goicoechea; Municipalidad de Desamparados; Municipalidad de Belén; Santa Bárbara (Costa Rica); San Rafael de Heredia; Alajuela; Zarcero; Concejo Municipal de Distrito de Monte Verde; Montes de Oca; Tilarán; Paraíso
Ecuador	15	Distrito Metropolitano de Quito; Santa Cruz de Galápagos; Ibarra; Santiago de Guayaquil; Municipalidad de Portoviejo; Municipality of Ambato; Alcaldía de Cuenca; Latacunga; Municipio de Loja; Baños de Agua Santa; Nueva Loja; Manta; Municipio de Santa Elena; Municipio de Montecristi; Municipalidad de Puyo
Guatemala	10	Municipio de San Jose; Municipality of La Antigua Guatemala; Municipalidad de Ciudad Vieja; Municipio de Iztapa; Municipio de Escuintla; Municipalidad de San Lucas Sacatepéquez; La Democracia; La Esperanza; Municipalidad Flores; Guatemala City
Honduras	1	Alcaldía de Tegucigalpa
Mexico	48	Municipio de Torreón; Municipio de Querétaro; Municipio de Mérida; Junta Intermunicipal de Medio Ambiente del Ayuquila Alto; Uruapan; Ayuntamiento de San Cristóbal de las Casas; Municipality of Santa Catarina; San Pedro Garza García; Mexico City; Ayuntamiento de Celaya; Ayuntamiento de Apodaca; Ayuntamiento de Chihuahua; Playa del Carmen; Ayuntamiento de Hermosillo; Ayuntamiento de Zapopan; Region Metropolitana de Guadalajara; Municipality of Irapuato; Municipality of San Pedro Tlaquepaque; Ayuntamiento de Concepción de Buenos Aires; Ayuntamiento de Almoloya de Juárez; Tultitlán; Junta Intermunicipal de Medio Ambiente Altos Sur (JIAS); Junta Intermunicipal de la Región Norte del Estado de Jalisco (JINOR); Junta Intermunicipal de Medio Ambiente Altos Norte (JIAN); Junta Intermunicipal de Medio Ambiente Region Valles (JIMAV); City of Monterrey; Delicias; Gobierno Municipal de Toluca de Lerdo; Ayuntamiento de Tepatitlán de Morelos; Ocoyoacac; Municipality of Salamanca; Municipalidad de Tampico; Heroica Guaymas de Zaragoza; GENERAL ESCOBEDO; Gobierno Municipal de León de los Aldamas; Ayuntamiento de San Luis Potosí; Valle de Santiago; Aipromades Lago de Chapala; Junta Intermunicipal de Medio Ambiente de Sierra Occidental y Costa (JISOC); Ayuntamiento de Tlajomulco de Zúñiga; Ciudad Madero; Valladolid; San Francisco del Rincón; Junta Intermunicipal de Medio Ambiente de la Costa Sur (JICOSUR); Presidencia Municipal de Saltillo; Junta Intermunicipal de la Cuenca Baja del Rio Ayuquila (JIRA); Junta Municipal de Medio Ambiente Lagunas (JIMAL); Ayuntamiento de Jesús María, Municipio de Manzanillo*, Municipio de Mexicali*, Municipio de Tijuana*, Municipalidad de Morelia*
Panama	1	Municipio La Chorrera
Paraguay	1	Ciudad de Asunción
Peru	26	Municipalidad Distrital de Sayán; Municipalidad Distrital de Huanchaco; Municipalidad Distrital de Chimbote; MUNICIPALIDAD DE SURQUILLO; Municipalidad Provincial de Pasco; Municipalidad de San Isidro (Lima); Municipalidad de San Borja; Municipalidad Distrital de Moche; Municipalidad de Independencia (Peru); Municipalidad de Miraflores; Metropolitan Municipality of Lima; Municipalidad Distrital de Masisea; Municipalidad Provincial de Huamanga; Municipalidad de Provincial de Arequipa; Municipalidad de Magdalena del Mar; Municipalidad Distrital de Ate; Municipalidad de Comas; Municipalidad de Santiago de Surco; Municipalidad

		Distrital de Jesús María; Municipalidad Distrital de Yura; Municipalidad Provincial de Coronel Portillo; Municipalidad Provincial de Maynas (Iquitos); Municipalidad Distrital de La Molina; Puente Piedra; Municipalidad Provincial de Piura; Municipalidad de Machu Picchu; Municipalidad Provincia del Callao*, Municipalidad Distrital de Chaupimarca*, Municipalidad Distrital de Chiclayo*,Municipalidad Distrital de Chimbote*,Municipalidad Distrital de Curahuasi*, Municipalidad Provincial de Cusco*,Municipalidad Distrital de Lince*, Municipalidad Distrital de Santa Maria del Mar*, Municipalidad Distrital de Sayan*, Municipalidad Provincial de Trujillo*
Uruguay	2	Intendencia de Canelones; Intendencia de Montevideo
Venezuela	2	Municipio Manuel Plácido Maneiro; Municipio de Maneiro

(1)Names are reported as the municipality/jurisdiction itself indicated in the survey or the the Global Covenant of Mayors webpage.
(2) Municipalities/jurisdictions that did not respond to the CDP survey but had a climate action plan, a technical climate action fact sheet or a risk and vulnerability plan on the Global Covenant of Mayors website are marked with an (*).

Additional analysis

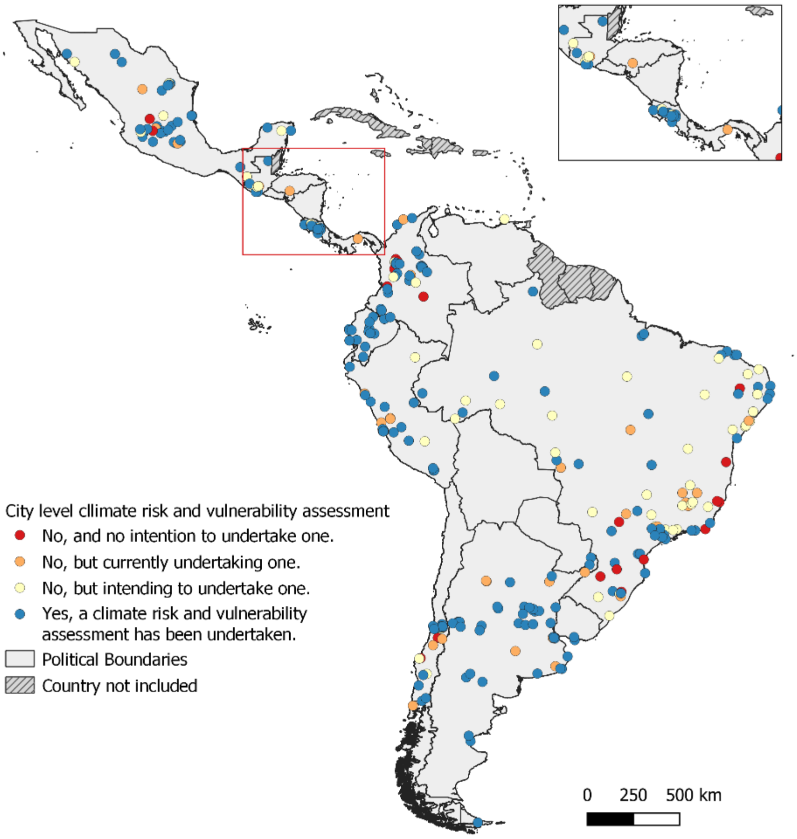

Figure A2.3. Local jurisdictions in Latin America that participated in the 2023 Carbon Disclosure Project (CDP) Annual Cities Survey and responded to “climate risk and vulnerability” section. Each point represents a local jurisdiction. Jurisdictions are color-coded to indicate their assessment status among those that responded to the survey.

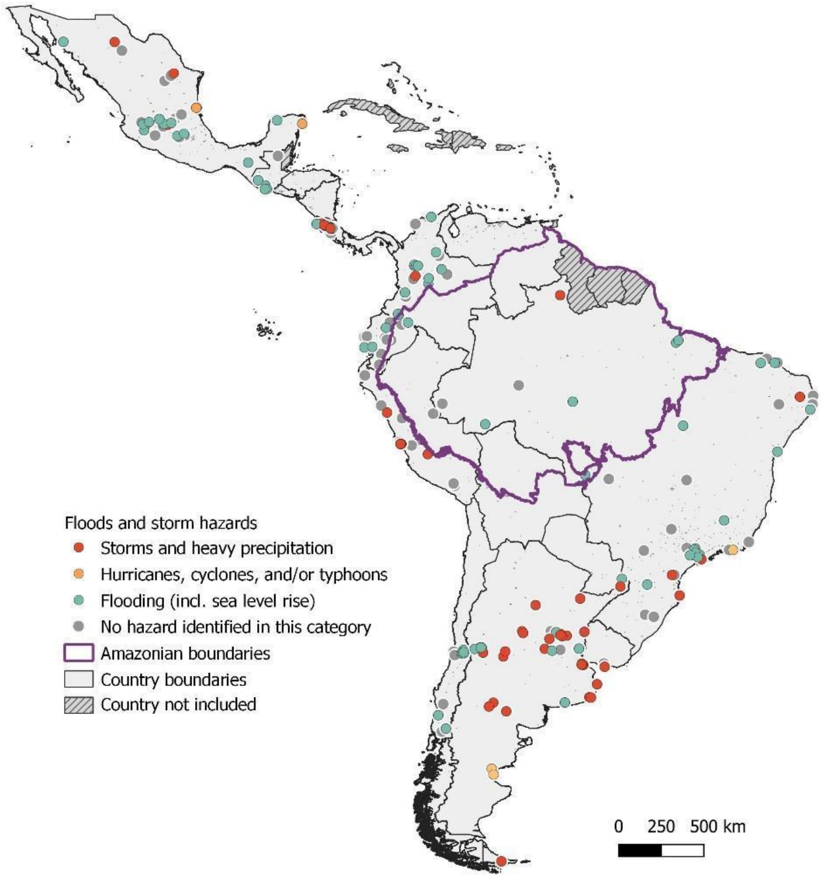

Figure A2.4. Local jurisdictions in Latin America that participated in the 2023 Carbon Disclosure Project (CDP) Annual Cities Survey and responded to "climate risk and vulnerability" section. Each point represents a local jurisdiction. Jurisdictions are color-coded to indicate their assessment status among those that responded to the survey.

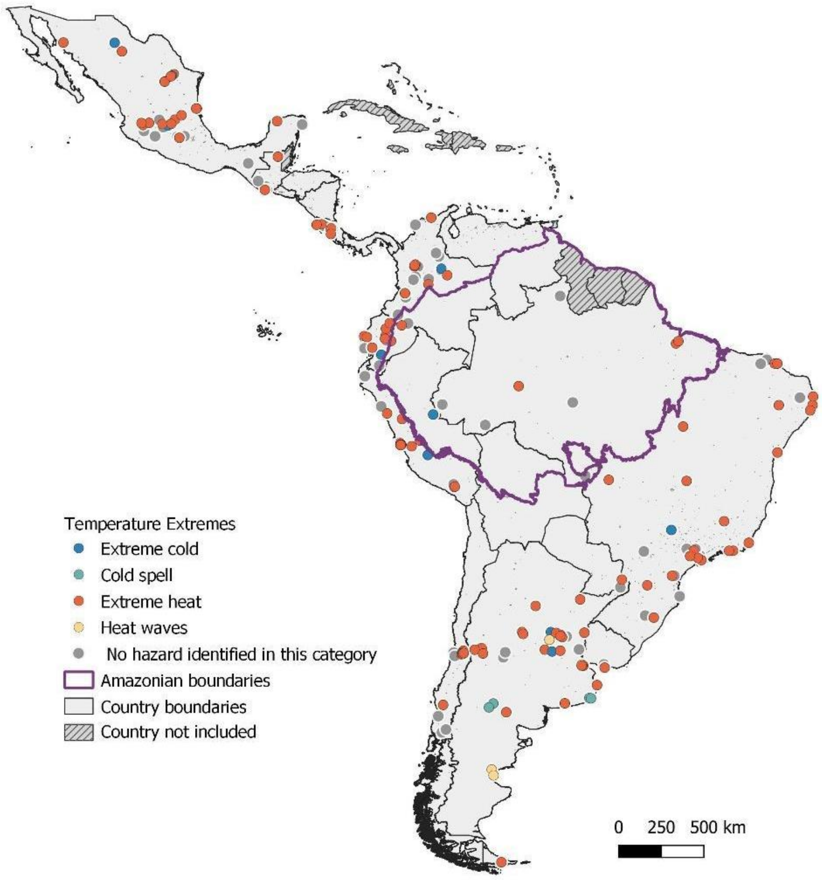

Figure A2.5. Local jurisdictions in Latin America that participated in the 2023 Carbon Disclosure Project (CDP) Annual Cities Survey and responded to "climate risk and vulnerability" section. Each point represents a local jurisdiction. Jurisdictions are color-coded to indicate their assessment status among those that responded to the survey.

Caveats and limitations

Participation in the survey is voluntary, resulting in limited coverage. In fact, responses represent approximately 2% of all municipalities in the region. The dataset encompasses various types of administrative divisions, such as local-level municipalities, city-wide municipalities, and in some cases, province-level municipalities. Despite this heterogeneity, the majority of responses pertain to local-level municipalities, offering a valuable, though partial, view of local climate risk assessments.

2.2: enabling conditions, adaptation delivery, and implementation

2.2.1: climate information for health

Regional author(s)

Francisco Chesini, Camila Llerena

Methods

This indicator follows the same methodology as the 2025 global Lancet Countdown report. Table A2.2 shows the participation status of Latin American countries in the PAHO 2023 survey on climate change and health and in the WMO State of Climate Services Report. It indicates whether countries submitted responses.

Table A2.2. PAHO 2023 survey and WMO State of Climate Services Report participating countries

**Country**	**WMO**	**PAHO 2023**
Argentina	Yes	Yes
Bolivia	Yes	Yes
Brazil	Yes	Yes
Chile	Yes	No
Colombia	Yes	Yes
Costa Rica	Yes	Yes
Ecuador	Yes	Yes
El Salvador	No data	Yes
Guatemala	Yes	Yes
Honduras	Yes	No
Mexico	Yes	No
Nicaragua	No Data	Yes
Panama	No Data	Yes
Paraguay	Yes	Yes
Peru	Yes	Yes
Uruguay	Yes	Yes
Venezuela	Yes	No

Additional analysis

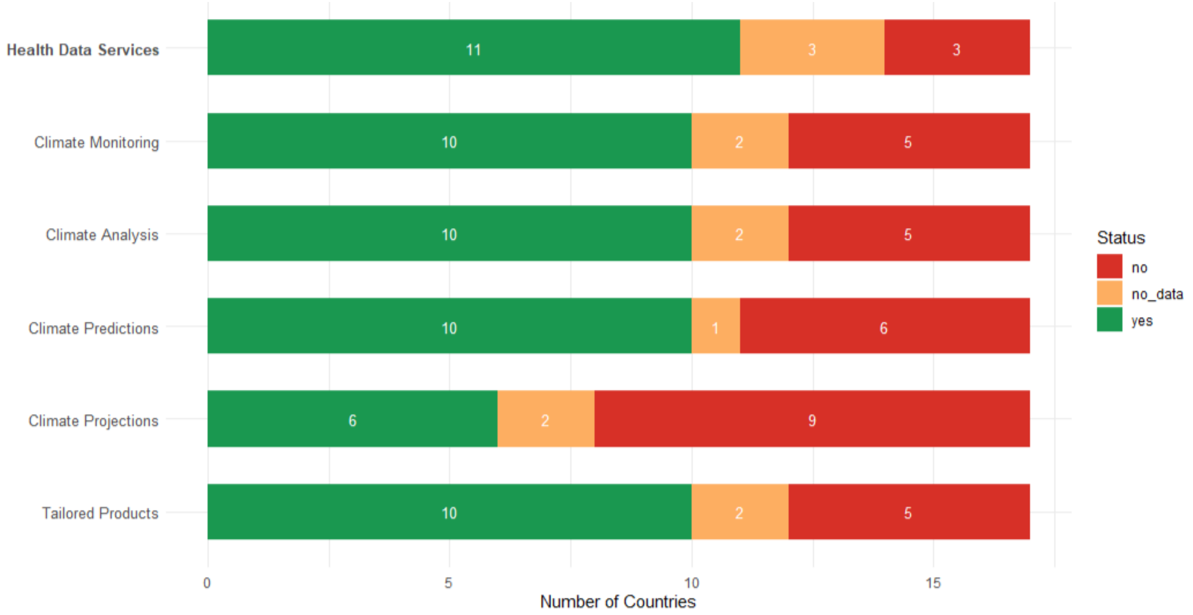

Figure A2.6. Number of countries providing six types of climate and health-related services to their respective health ministries based on WMO data.

Caveats and limitations

This indicator is based on self-reported information from national authorities, which may be subject to inaccuracies in reporting. Participation in the survey was voluntary, introducing the potential for response bias. However, the high participation rate ensures broad regional coverage. Consequently, the figures reported represent a snapshot of the information available up to a specific date and may change throughout the year as countries update their profiles at different times.

2.2.2: benefits and harms of air conditioning

Regional author(s)

Monica Pinilla-Roncancio, Camila Llerena

Methods

This indicator follows the same methodology as the 2025 global *Lancet Countdown* report at the regional level in relation to averted deaths due to air conditioning. However, instead of using IEA estimates—unavailable at the national level for publication—we relied on national household surveys. Mexico, Guatemala, Costa Rica, Venezuela, Brazil , Argentina, Peru , Nicaragua and Chile where not included since they did not had a specific question on air conditioning use in their National Household survey. Table A2.2 outlines the data sources used for each country and indicates whether a question on air conditioning availability was avialable.

Table A2.2. National Household survey reviewed and availability of an air conditioning question.

Country	Source	Year	Aircon question available
Mexico	Encuesta de Gastos: https://www.inegi.org.mx/programas/enigh/nc/2022/	2022	No
Guatemala	Encuesta Nacional de Condiciones de Vida ENCOVI https://www.proyectoencovi.com/	2023	No
Costa Rica	Encuesta Nacional de Hogares https://inec.cr/estadisticas-fuentes/encuestas/encuesta-nacional-hogares	2024	No
Honduras	Encuesta Permanente de Hogares de Propósitos Múltiples https://www.ine.gob.hn/EncuestaHogares.html	2022	Yes
Panama	No survey available		No
El Salvador	Encuesta de Hogares de Propósitos Múltiples (EHPM) https://onec.bcr.gob.sv/encuesta-de-hogares-de-propositos-multiples-ehpm/	2022	Yes
Colombia	Encuesta nacional de calidad de vida (ECV) https://www.dane.gov.co/index.php/estadisticas-por-tema/pobreza-y-condiciones-de-vida/calidad-de-vida-ecv	2023	Yes
Ecuador	Encuesta Nacional Multipropósito de Hogares https://www.ecuadorencifras.gob.ec/encuesta-nacional-multiproposito-de-hogares/	2023	Yes
Bolivia	Encuesta de Hogares https://www.ine.gob.bo/index.php/estadisticas-sociales/vivienda-y-servicios-basicos/encuestas-de-hogares-vivienda/	2023	Yes
Uruguay	Encuesta Continua de Hogares https://www4.ine.gub.uy/Anda5/index.php/catalog/735	2023	Yes
Paraguay	Encuesta Permanente de Hogares – EPH https://www.ine.gov.py/datos/encuestas/eph/	2024	Yes
Venezuela	No survey available		No

Brasil	PNAD Contínua - Pesquisa Nacional por Amostra de Domicílios Contínua https://www.ibge.gov.br/estatisticas/sociais/saude/17270-pnad-continua.html	2022	No
Argentina	Encuesta Permanente de Hogares (EPH) https://www.indec.gob.ar/indec/web/Institucional-Indec-BasesDeDatos	2022	No
Peru	Encuesta Nacional de Hogares sobre Condiciones de Vida y Pobreza https://proyectos.inei.gob.pe/iinei/srienaho/Descarga/DocumentosMetodologicos/2024-63/01_Ficha_Tecnica.pdf	2024	No
Nicaragua	Encuesta de Medición de Nivel de vida https://www.inide.gob.ni/Home/enmv	2016	No
Chile	Encuesta de Caracterización Socioeconómica Nacional (Casen) https://observatorio.ministeriodesarrollosocial.gob.cl/encuesta-casen	2022	No

Additional analysis

There are no additional analyses for this indicator.

Caveats and limitations

For a detailed description of the methodological caveats and limitations related to the estimation of the number of heat-related deaths averted by air conditioning, please refer to the Appendix of the global Lancet Countdown report.

2.2.3: urban greenspace

Regional author(s)

Nelson Gouveia, Camila Llerena

Methods

This indicator follows the same methodology as the 2025 global Lancet Countdown report.

Urban centres chosen for the indicator were identified as urban centres larger than 500,000 inhabitants. Green space was estimated using the normalised difference vegetation index (NDVI), the most commonly used satellite-based vegetation index. NDVI values range from -1·0 to 1·0 with values less than 0 indicating water and values close to 1 indicating high levels of vegetation density. Table A2.3 presents the greenness classification categories used for the indicator, based on population-weighted peak NDVI values.

Table A2.3. Greenness classification categories

Greenness level	Population weighted average peak NDV value
Extremely low	<0.20
Very low	0.20-0.29
Low	0.30-0.39
Medium	0.40-0.49
High	0.50-0.59
Very high	0.60-0.69
Extremely high	≥ 0.70

Additional analysis

Table A2.4 Distribution of Latin American cities across greenness categories for the full historical period (2015–2024).

Greenness level	2015	2016	2017	2018	2019	2020	2021	2022	2023	2024
Extremely low	26	26	28	25	23	27	25	27	25	25
Very low	67	70	69	71	74	69	71	70	74	74
Low	15	12	11	12	11	12	12	11	10	10
Medium	1	1	1	1	1	1	1	1	0	
High	0	0	0	0	0	0	0	0	0	0
Very high	0	0	0	0	0	0	0	0	0	0
Extremely high	0	0	0	0	0	0	0	0	0	0

Greenness per city: The following plots show the population weighted average peak NDV across all seasons from 2015-2017 (circle-green) versus 2022-2024 (diamond-red).

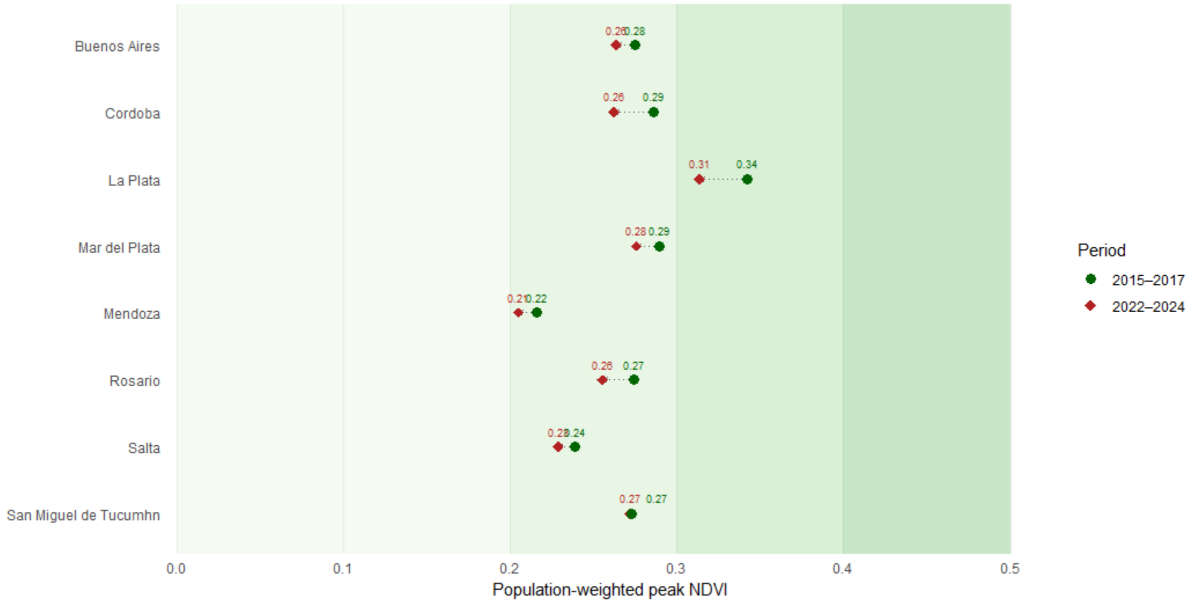

Figure A2.7. Population weighted average peak NDVI across all seasons in 2015-2017 (circle-green) versus 2022-2024 (diamond-red) in Argentina.

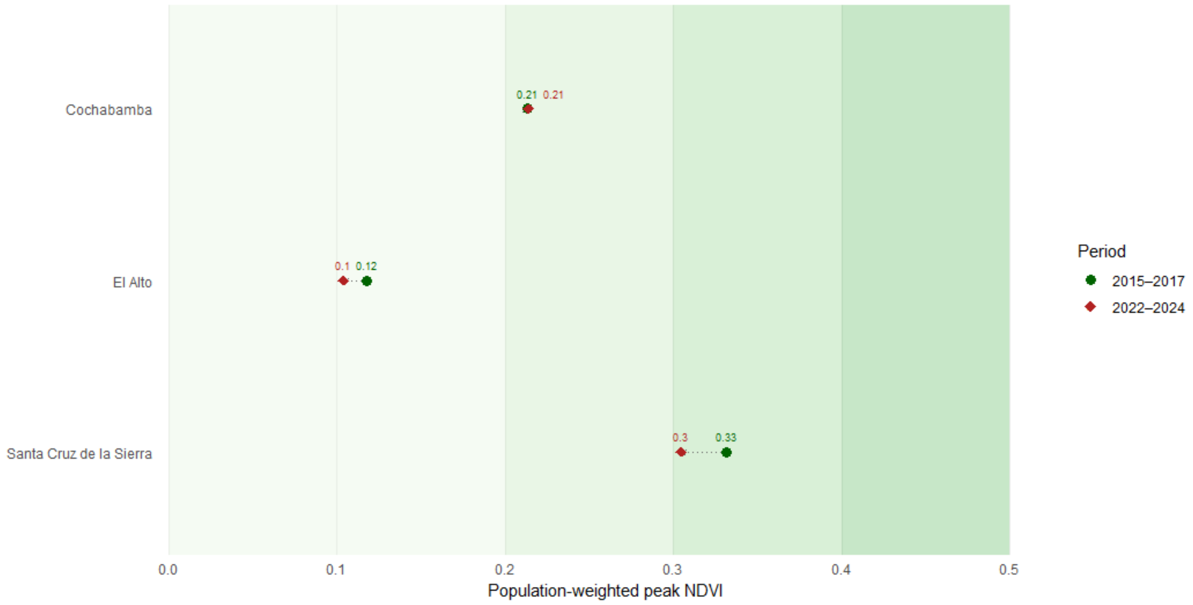

Figure A2.8 Population weighted average peak NDVI across all seasons in 2015-2017 (circle-green) versus 2022-2024 (diamond-red) in Bolivia.

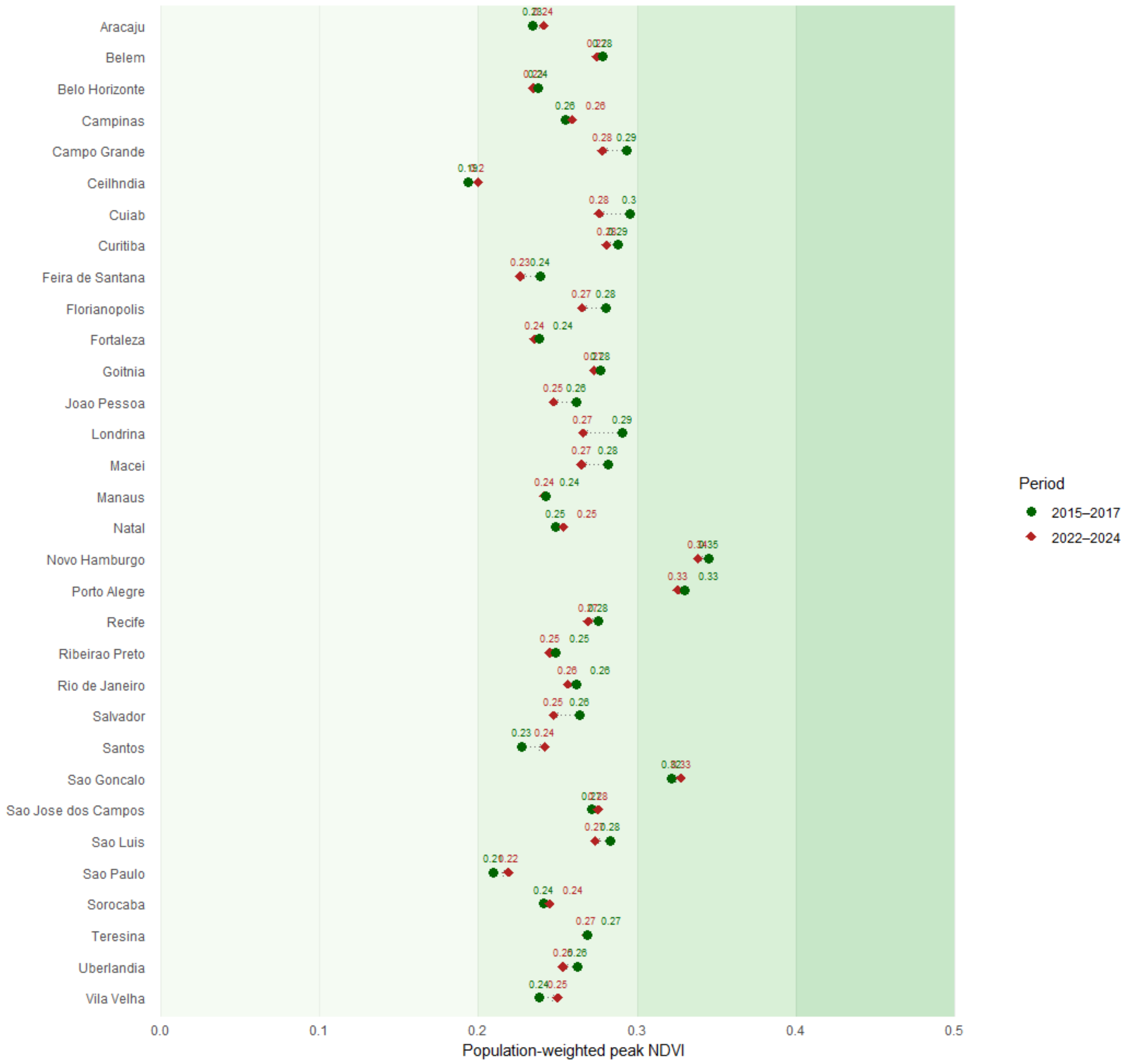

Figure A2.9 Population weighted average peak NDVI across all seasons in 2015-2017 (circle-green) versus 2022-2024 (diamond-red) in Brazil.

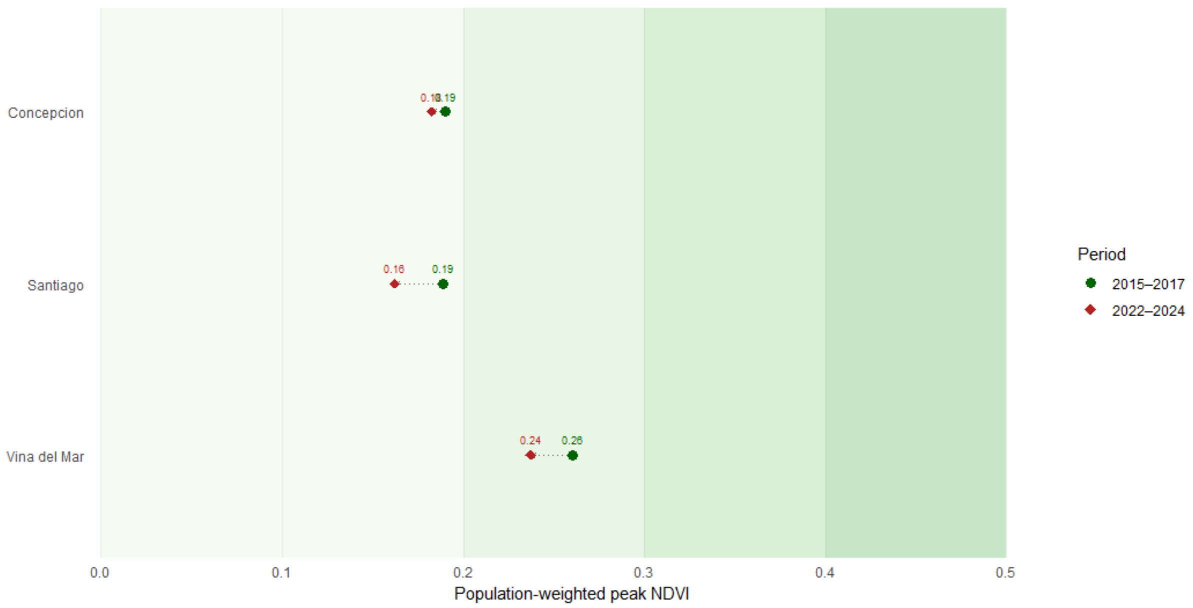

Figure A2.10 Population weighted average peak NDVI across all seasons in 2015-2017 (circle-green) versus 2022-2024 (diamond-red) in Chile.

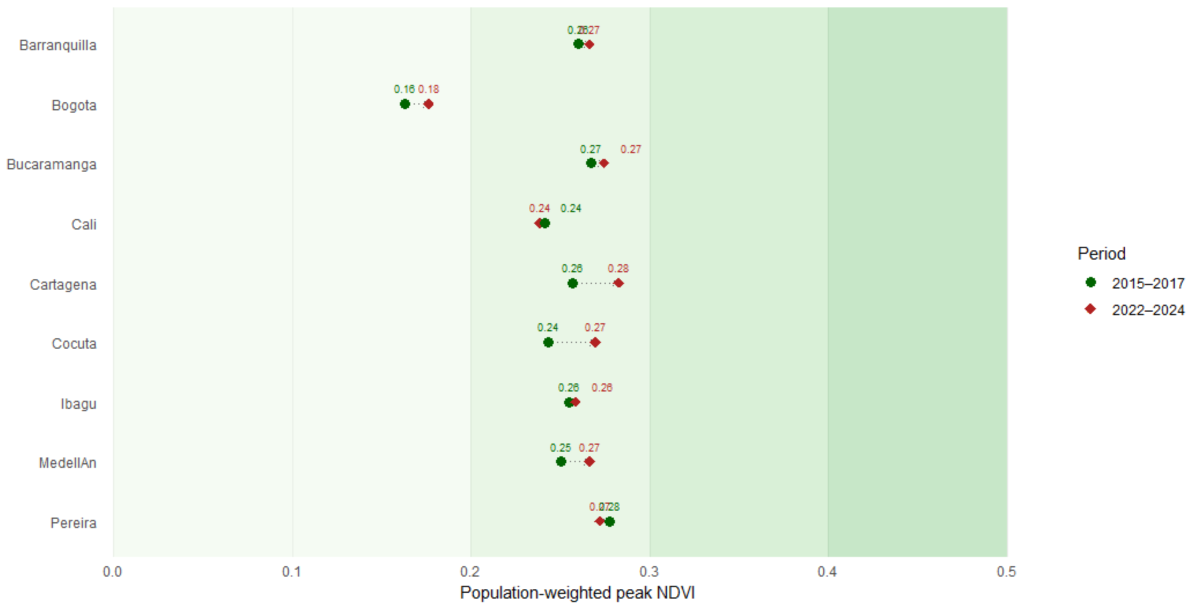

Figure A2.11 Population weighted average peak NDVI across all seasons in 2015-2017 (circle-green) versus 2022-2024 (diamond-red) in Colombia.

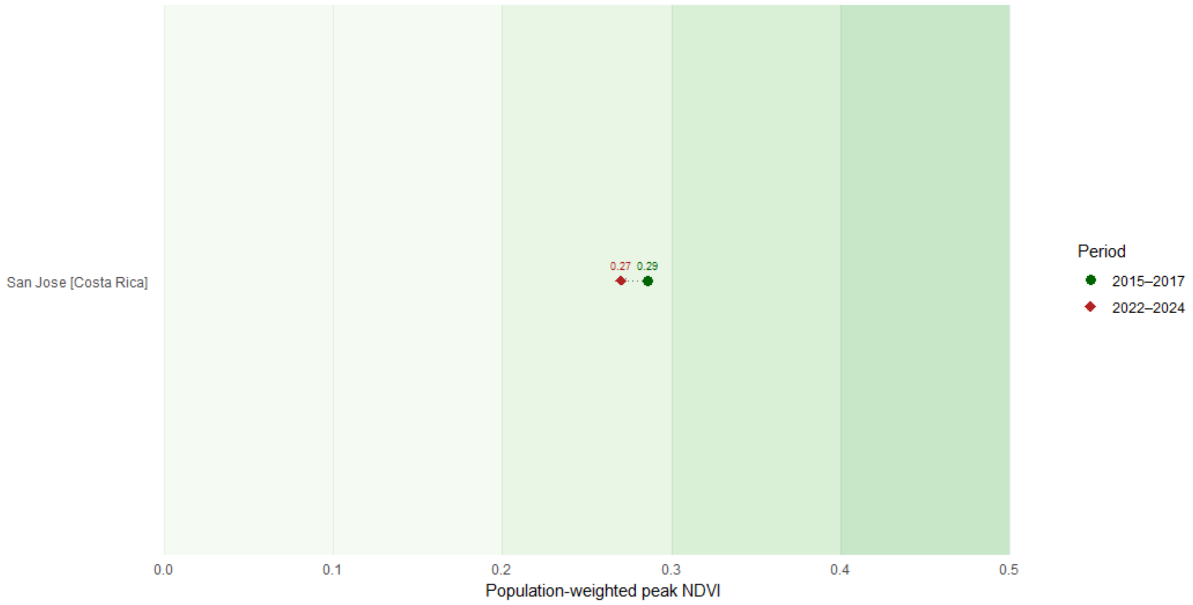

Figure A2.12 Population weighted average peak NDVI across all seasons in 2015-2017 (circle-green) versus 2022-2024 (diamond-red) in Costa Rica.

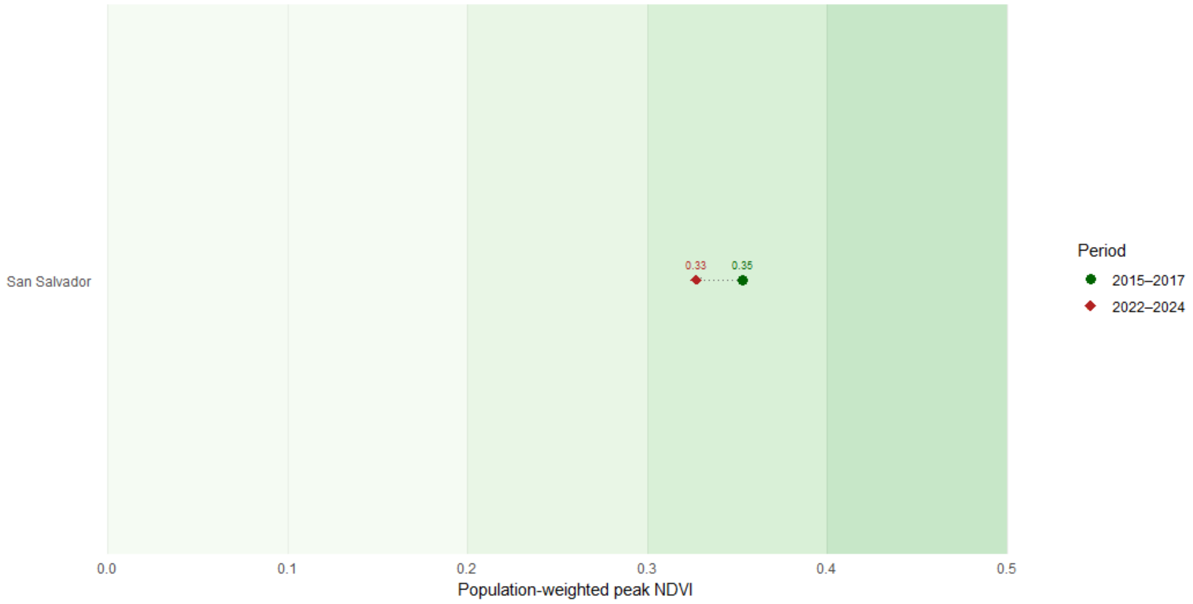

Figure A2.13 Population weighted average peak NDVI across all seasons in 2015-2017 (circle-green) versus 2022-2024 (diamond-red) in Ecuador.

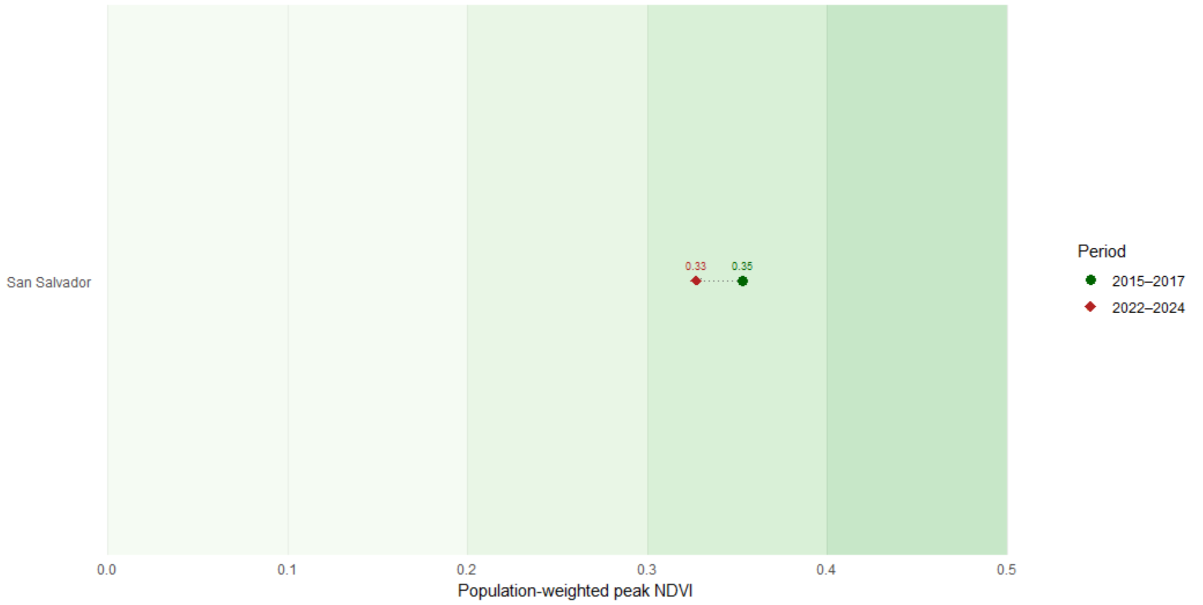

Figure A2.14 Population weighted average peak NDVI across all seasons in 2015-2017 (circle-green) versus 2022-2024 (diamond-red) in El Salvador.

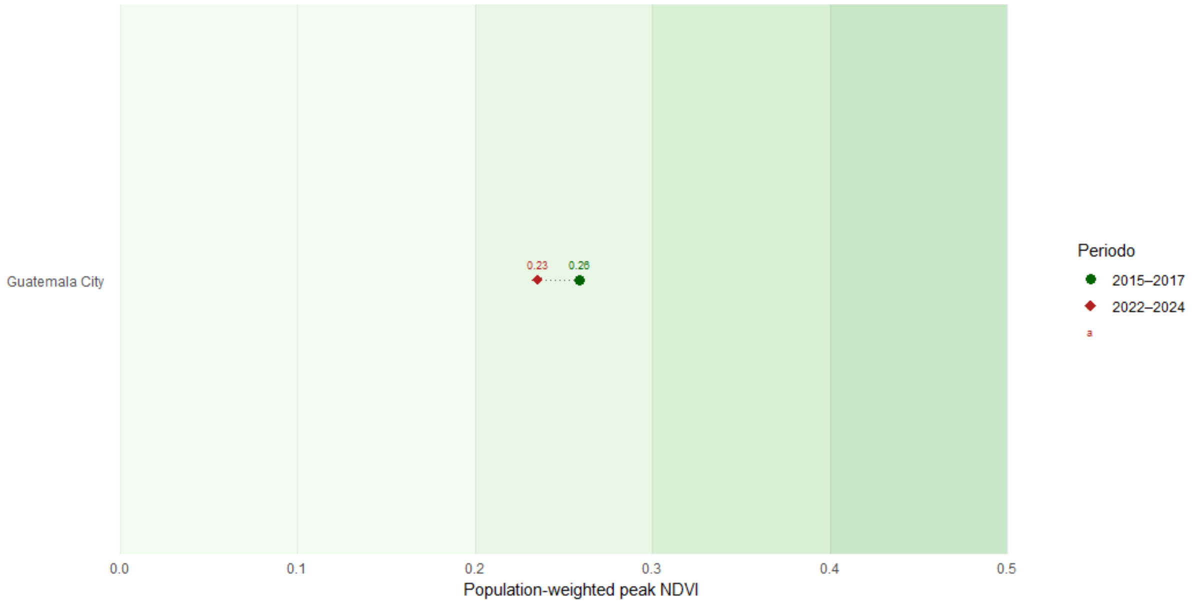

Figure A2.15 Population weighted average peak NDVI across all seasons in 2015-2017 (circle-green) versus 2022-2024 (diamond-red) in Guatemala.

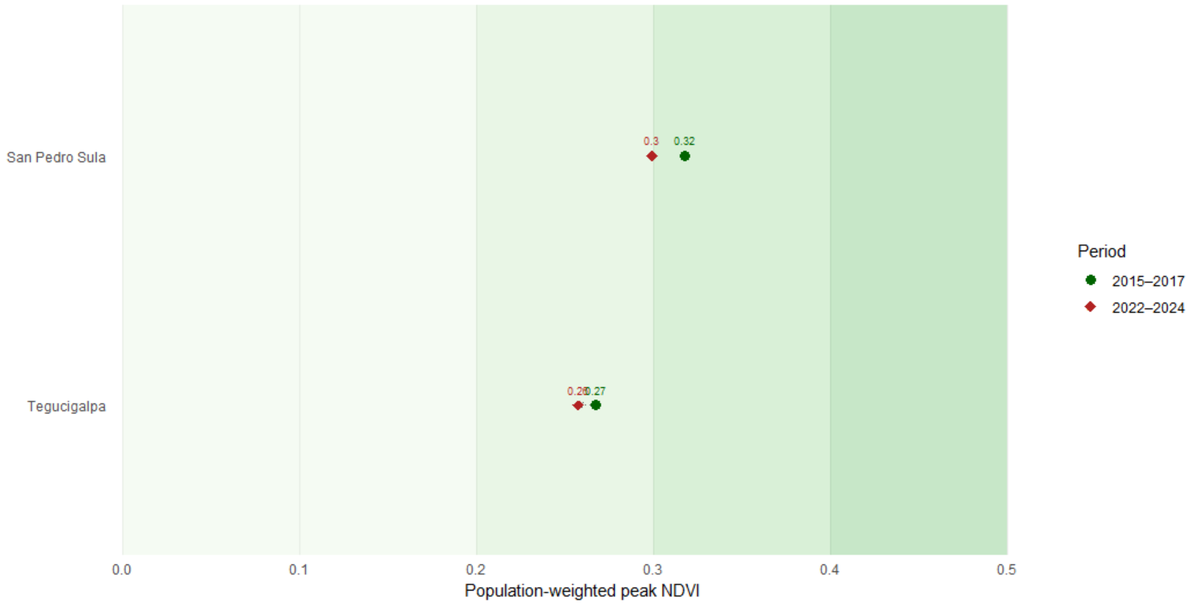

Figure A2.16 Population weighted average peak NDVI across all seasons in 2015-2017 (circle-green) versus 2022-2024 (diamond-red) in Honduras.

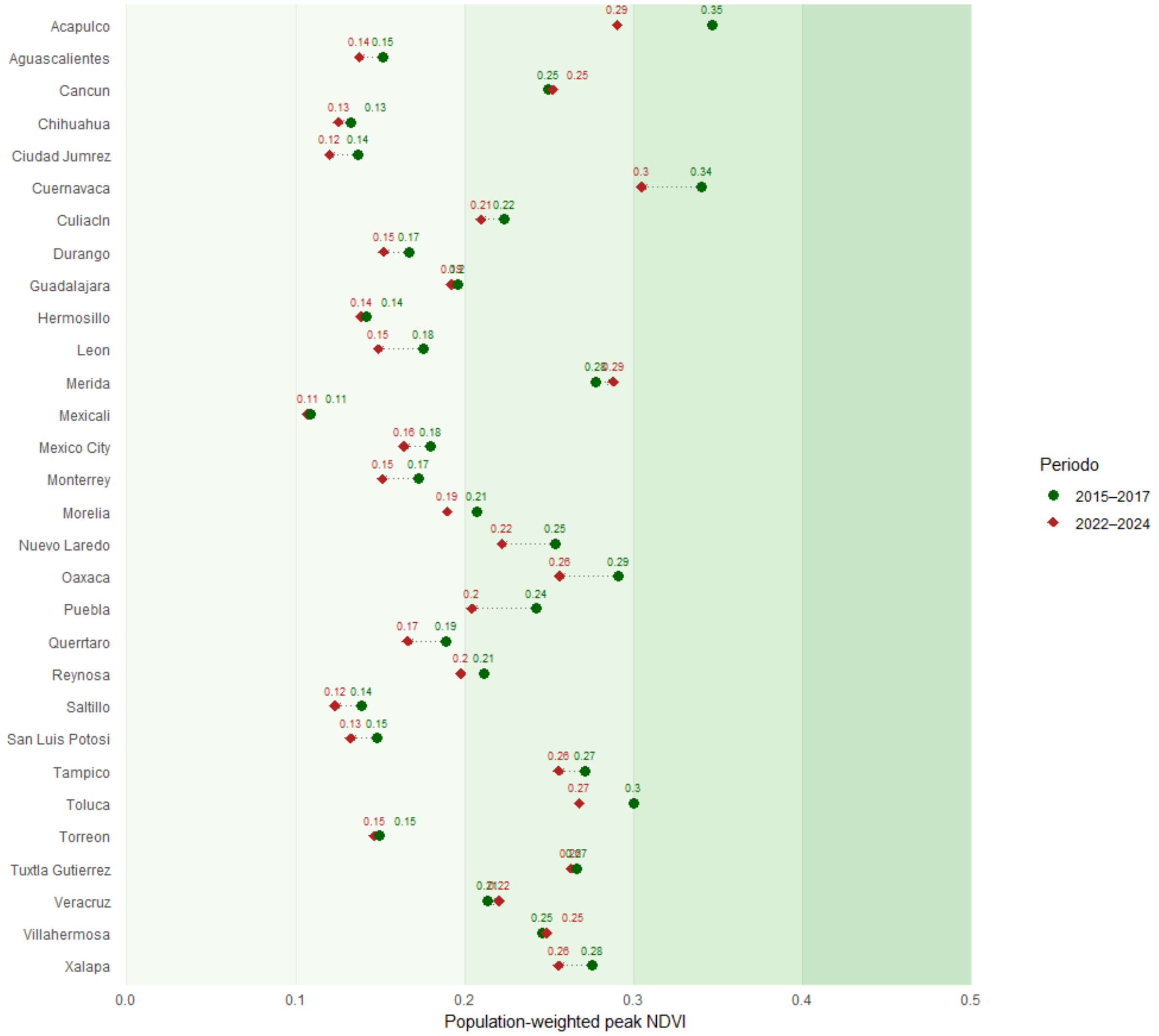

Figure A2.17 Population weighted average peak NDVI across all seasons in 2015-2017 (circle-green) versus 2022-2024 (diamond-red) in Mexico.

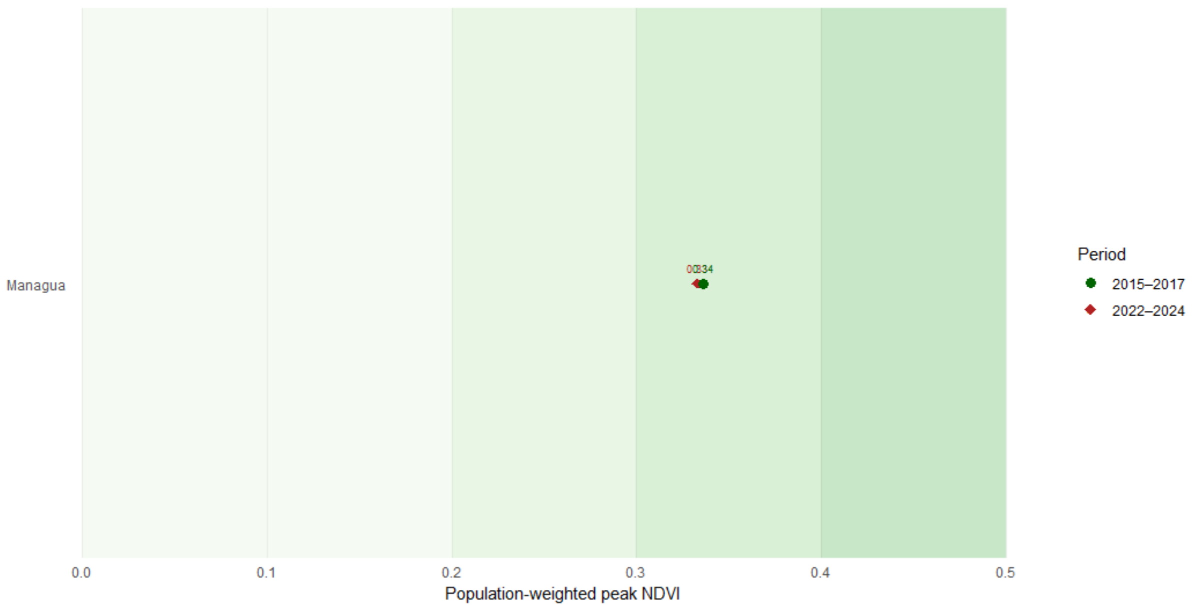

Figure A2.18 Population weighted average peak NDVI across all seasons in 2015-2017 (circle-green) versus 2022-2024 (diamond-red) in Nicaragua.

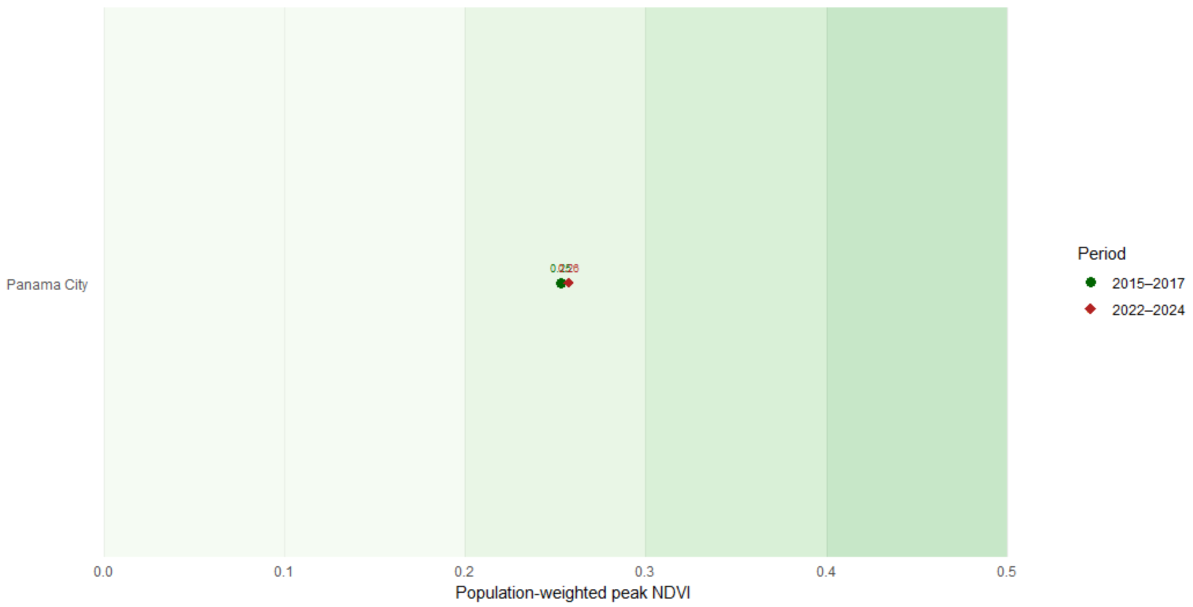

Figure A2.19 Population weighted average peak NDVI across all seasons in 2015-2017 (circle-green) versus 2022-2024 (diamond-red) in Panamá.

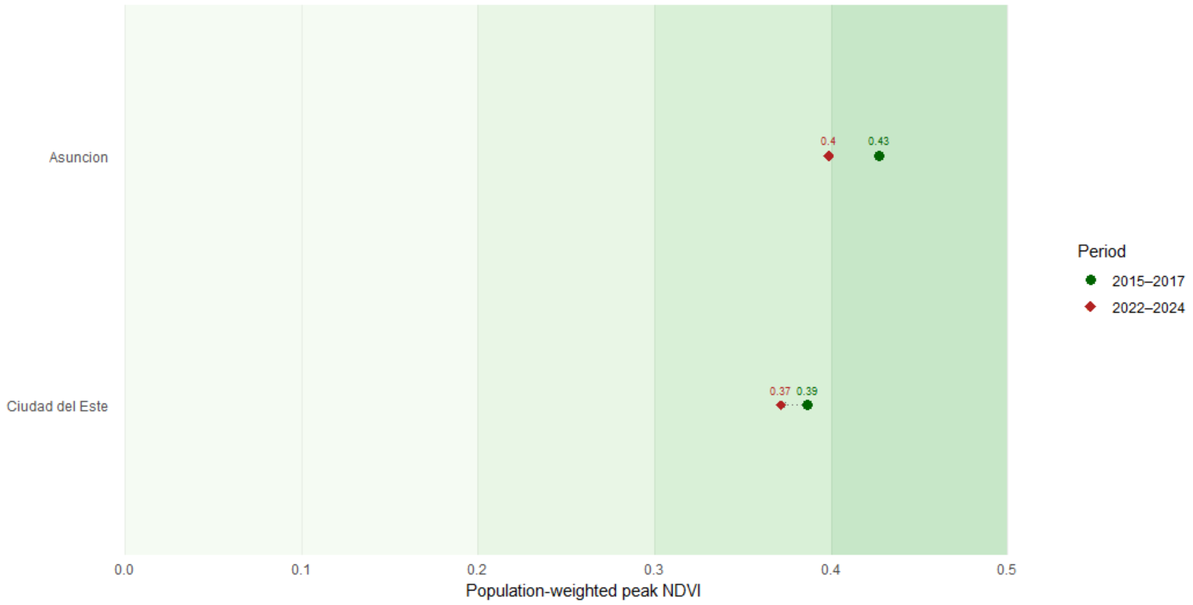

Figure A2.20 Population weighted average peak NDVI across all seasons in 2015-2017 (circle-green) versus 2022-2024 (diamond-red) in Paraguay.

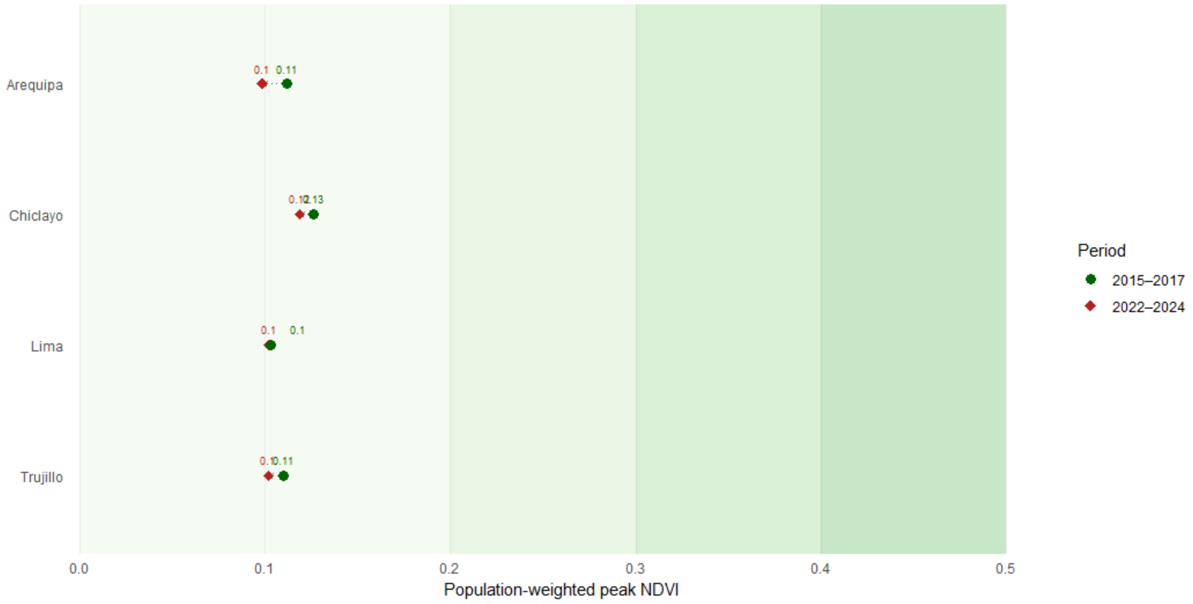

Figure A2.21 Population weighted average peak NDVI across all seasons in 2015-2017 (circle-green) versus 2022-2024 (diamond-red) in Peru.

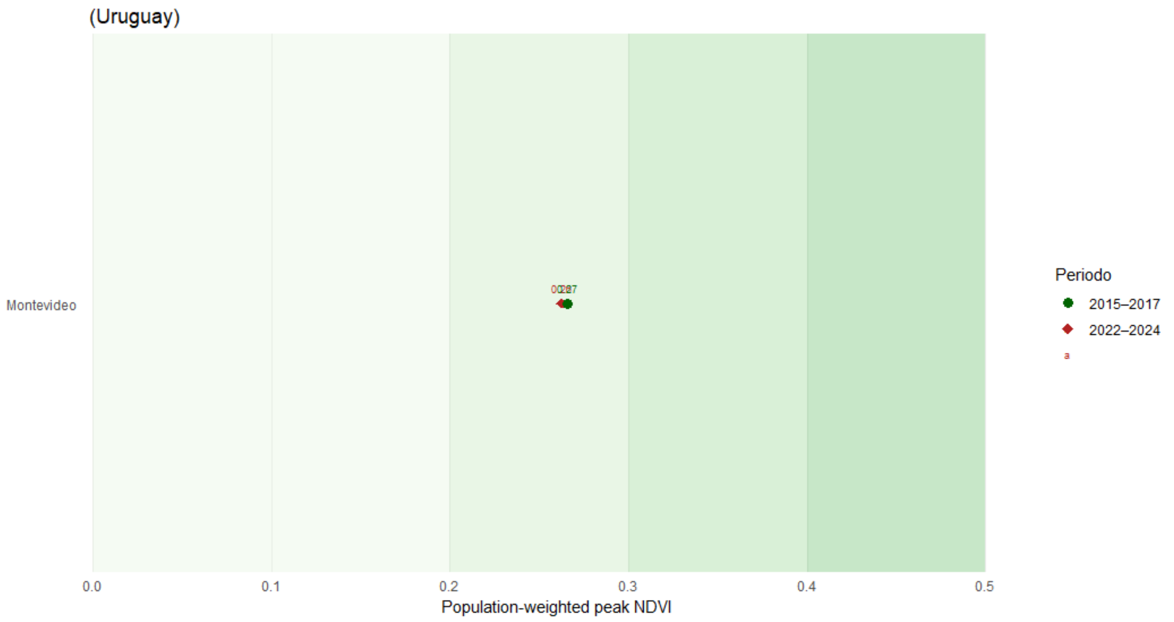

Figure A2.22 Population weighted average peak NDVI across all seasons in 2015-2017 (circle-green) versus 2022-2024 (diamond-red) in Uruguay.

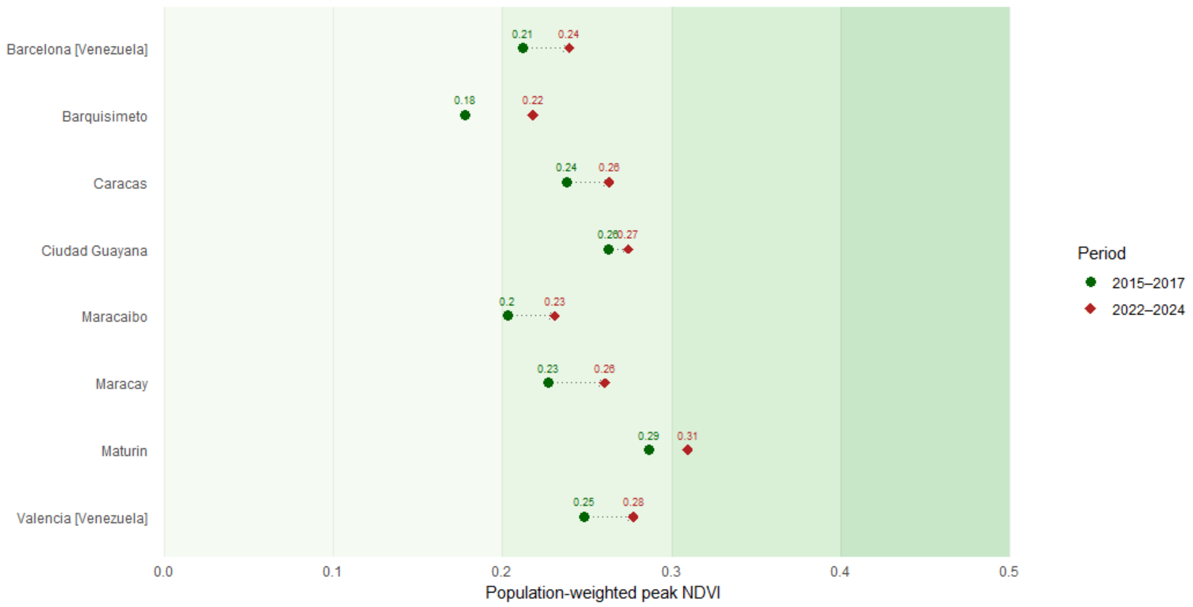

Figure A2.23 Population weighted average peak NDVI across all seasons in 2015-2017 (circle-green) versus 2022-2024 (diamond-red) in Venezuela.

Table A2.5. Five cities with the highest population-weighted (PW) peak NDVI during the most recent period (2021–2024)

N	**City**	**Country**	**PW peak NDVI**	**Category**
1	Asuncion	Paraguay	0.399	Low
2	Ciudad del Este	Paraguay	0.371	Low
3	Novo Hamburgo	Brazil	0.338	Low
4	Managua	Nicaragua	0.332	Low
5	Sao Goncalo	Brazil	0.328	Low

Table A2.6. Five cities with the lowest population-weighted (PW) peak NDVI during the most recent period (2021–2024)

N	**City**	**Country**	**PW peak NDVI**	**Category**

1	Arequipa	Peru	0.098	Extremely low
2	Trujillo	Peru	0.102	Extremely low
3	Lima	Peru	0.102	Extremely low
4	El Alto	Bolivia	0.104	Extremely low
5	Mexicali	Mexico	0.107	Extremely low

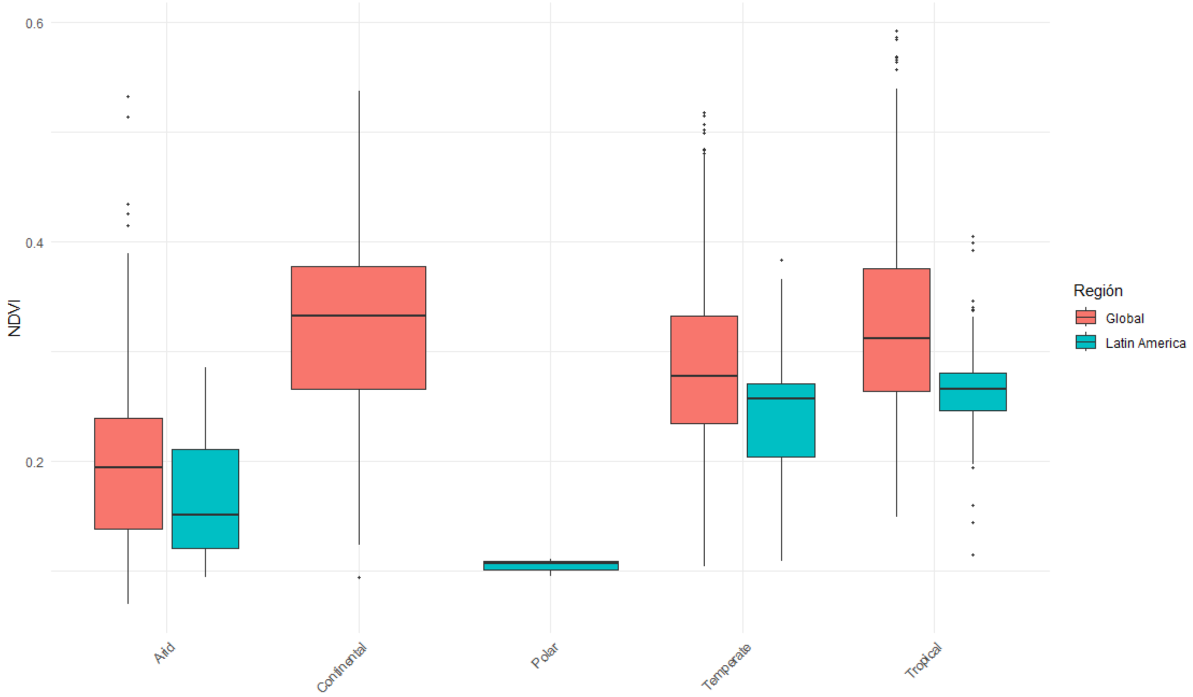

Figure A2.24. Population-weighted (PW) NDVI (2022-2024) in Latin America and Global by Köppen climatic zone *El Alto in Bolivia is the only city (>500,000 inhabitants) classified as “Polar”.

Caveats and limitations

For a detailed description of the methodological limitations of using NDVI to assess urban greenspace, please refer to the Appendix of the global Lancet Countdown report. Specifically for Latin America, a key limitation is that the indicator focuses on cities with populations exceeding 500,000 inhabitants. As a result, it may not fully represent the urban population of countries where a significant proportion of people live in smaller cities. This is particularly relevant for countries such as Paraguay, Guatemala, El Salvador and Venezuela, where urban populations are concentrated in medium-sized cities not captured by this analysis.

2.2.4: detection, preparedness, and response to health emergencies

Regional author(s)

Zaray Miranda Chacon, Camila Llerena

Methods

This indicator follows the same methodology as the 2025 global Lancet Countdown report.
This indicator tracks the implementation of core capacity 7 (Health Emergency Management) under the International Health Regulations (IHR). Data were retrieved in May 2025 from the electronic IHR State Parties Self-Assessment Annual reporting Tool (e-SPAR).[9] As some changes occurred in the survey over the years, results are not fully comparable between them.

The Lancet Countdown’s classification of the level of implementation of core capacity 7 of the IHR SPAR tool. This is the same classification of the previous Lancet Countdown global regional reports.

Table A2.7. Lancet Countdown classification of the core capacity 7 score

Score range	Lancet Countdown classification

0-20	Very low
21-40	Low
41-60	Medium
61-80	High
81-100	Very high

Additional analysis

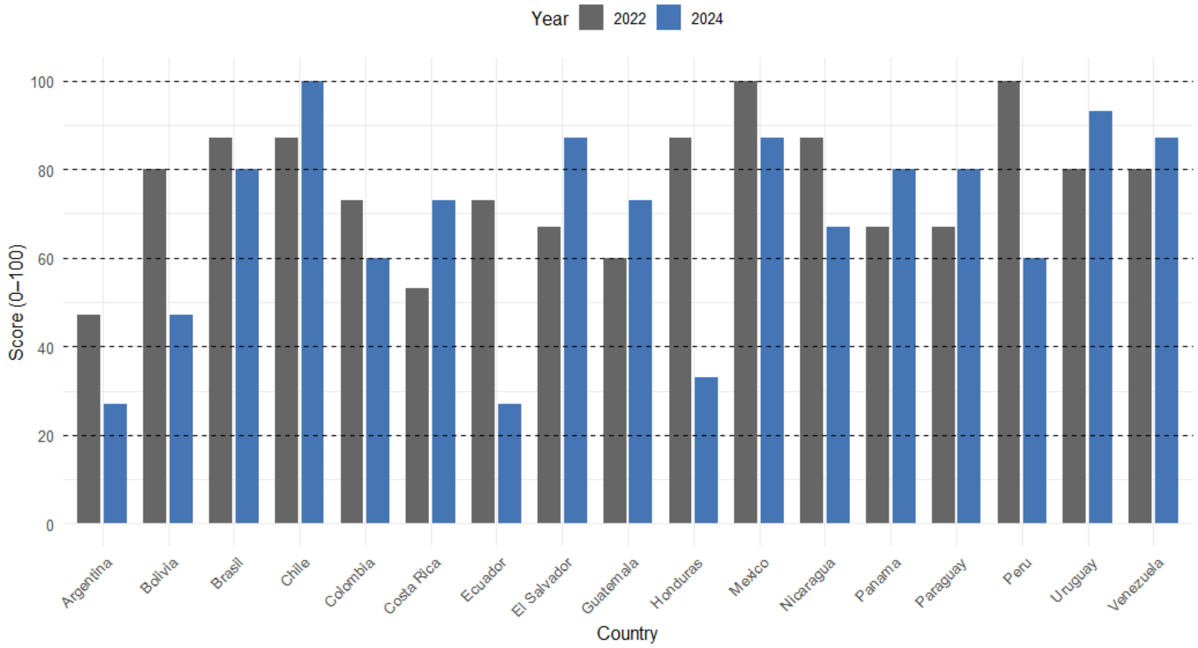

Figure A2.25. Level of implementation of core capacity 7 (Health Emergency Management) under the International Health Regulations (IHR) in 2022 and 2024.

Based on PAHO data, ten countries indicated having plans and procedures for weather- and climate-related disaster preparedness, either as stand-alone documents or integrated into broader climate-related policies.

Caveats and limitations

This indicator is based on self-reported responses to the IHR monitoring questionnaire, which may be subject to inaccuracies or inconsistencies in reporting. For a detailed description of the limitations of the IHR monitoring instrument, please refer to the Appendix of the global Lancet Countdown report.

2.2.5: climate and health education and training

Regional author(s)

Cecilia Sorensen

Methods

This indicator follows the same methodology as the 2025 global Lancet Countdown report.

Additional analysis

Table A2.7. Survey responses and reported climate and health education among Latin American countries during 2024-25

Latin America Countries	Number of responding institutions, n	Institutions reporting climate and health education, % (n)	Institutions reporting climate and health education as part of mandatory curricula, % (n)	Institutions reporting independent Climate and Health Concentration or Certificate program, n
**Public Health Institutions**				
Argentina	*9*	89 (8)	44 (4)	0 (0)
Bolivia	*1*	0 (0)	0 (0)	0 (0)
Brazil	*22*	59 (13)	36 (8)	9 (2)
Chile	*6*	33 (2)	0 (0)	0 (0)
Colombia	*9*	56 (5)	44 (4)	0 (0)
Costa Rica	*1*	0 (0)	0 (0)	0 (0)
Ecuador	*7*	29 (2)	14 (1)	0 (0)
El Salvador	*2*	0 (0)	0 (0)	0 (0)
Guatemala	*2*	100 (2)	50 (1)	0 (0)
Mexico	*5*	80 (4)	60 (3)	0 (0)
Panama	*1*	0 (0)	0 (0)	0 (0)
Paraguay	*3*	33 (1)	0 (0)	0 (0)
Peru	*10*	80 (8)	70 (7)	10 (1)
Venezuela	*1*	0 (0)	0 (0)	0 (0)
**Total**	***79***	**57 (45)**	**35 (28)**	**4 (3)**
*** There were no Public Health respondents from Honduras, Nicaragua, or Uruguay**				
**Medical Institutions**				
Argentina	*3*	33 (1)	33 (1)	33 (1)
Brazil	*3*	67 (2)	33 (1)	33 (1)
Colombia	*6*	67 (4)	67 (4)	0 (0)
Peru	*2*	50 (1)	50 (1)	0 (0)
**Total**	14	57 (8)	50 (7)	14 (2)
*** There were no Medical respondents from Bolivia, Chile, Costa Rica, Ecuador, El Salvador, Guatemal[illegible] Honduras, Mexico, Nicaragua, Panama, Paraguay, Uruguay, or Venezuela.**				

Table A2.8. Total number of students trained via mandatory curricular offerings across , based on UNDP HDI classification.

Latin America Countries	Number of students enrolled at responding institutions,	Students receiving C+H training, n (%)	Students receiving mandatory C+H training, n (%)	Degree program with C+H training, n			
				Vocational	Undergraduate	Master's	Doctoral
**Public Health Institutions**							
Argentina	12876	4667 (36)	3868 (30)	1029	2820	543	275
Bolivia	1501	0 (0)	0 (0)	0	0	0	0
Brazil	26935	9075 (34)	2818 (10)	1693	2905	2958	1519
Chile	4715	127 (3)	0 (0)	0	0	120	7
Colombia	11336	842 (7)	835 (7)	282	255	305	0
Costa Rica	550	0 (0)	0 (0)	0	0	0	0
Ecuador	7926	60 (1)	40 (1)	20	0	40	0
El Salvador	782	0 (0)	0 (0)	0	0	0	0
Guatemala	1601	33 (2)	13 (1)	0	0	13	20
Mexico	12804	268 (2)	268 (2)	0	80	161	27
Panama	83	0 (0)	0 (0)	0	0	0	0
Paraguay	1245	96 (8)	0 (0)	48	32	16	0
Peru	19879	2912 (15)	2878 (14)	0	2294	577	41
Venezuela	2466	0 (0)	0 (0)	0	0	0	0
**Total**	104699	18080 (17)	10720 (10)	3072	8386	4733	1889
*** There were no Public Health respondents from Honduras, Nicaragua, or Uruguay**							
**Medical Institutions**							

Argentina	1568	732 (47)	732 (47)
Brazil	2356	1377 (58)	720 (31)
Colombia	5304	3881 (73)	3881 (73)
Peru	1314	657 (50)	657 (50)
**Total**	*10542*	*6647 (63)*	*5990 (57)*
*** There were no Medical respondents from Bolivia, Chile, Costa Rica, Ecuador, El Salvador, Guatemala, Honduras, Mexico, Nicaragua, Panama, Paraguay, Uruguay, or Venezuela.**			

Table A2.9. Degree-level public health and medical programs offering climate and health education and type of training offered during 2024-25.

**Degree programs**	**Number of degree programs by type, n**	**Degree programs offering C+H training, % (n)**	**Required offerings**		**Elective offerings**		
			**Standalone required course, n**	**Part of the required core curriculum, n**	**Standalone elective course, n**	**Part of elective curriculum, n**	**Climate and Health Concentration or Certificate, n**
**Public Health Institutions**							
**Vocational**	28	39 (11)	4	5	6	3	2
**Undergraduate**	47	47 (22)	6	13	7	7	0
**Masters**	58	42 (30)	5	11	10	8	1
**Doctoral**	30	47 (14)	4	3	5	6	0
Total	*163*	*47 (77)*	*19*	*32*	*28*	*24*	*3*
**Medical Institutions**							
Total	*14*	*57 (8)*	*1*	*6*	*1*	*0*	*2*

Figure 1.

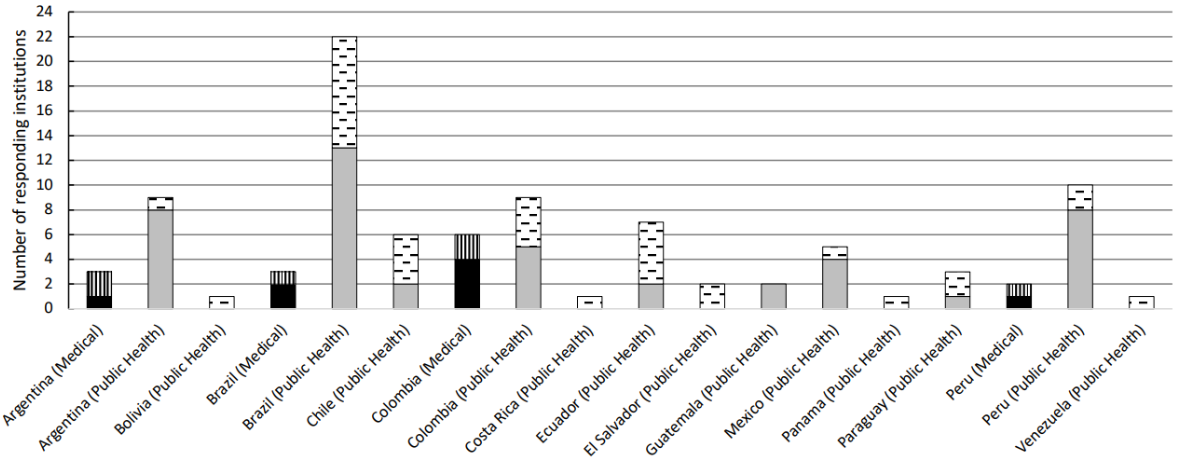

Figure A2.26 Number of public health and medical schools that responded to the survey, displayed by country. Each country is represented by two bars (when available): one for public health schools and one for medical schools. The bars are color-coded to indicate the proportion of schools offering climate and health education.

Caveats and limitations

This indicator is based on self-reported data from academic institutions, with a limited response rate (approximately 3% of Faculties of Medicine in the region.[10] As a result, the findings do not necessarily represent the actual state of climate change and health education in the region. For a detailed description of the methodological limitations of the underlying data collection instrument, please refer to the Appendix of the global Lancet Countdown report.

2.3: vulnerabilities, health risk, and resilience to climate change

2.3.1: risk to severe mosquito-borne diseases

Regional author(s)

Mauricio Santos-Vega, Juan D Umaña

Methods

The Mosquito Risk Index (MoRI) is estimated as the relationship of a threat (a normalized mosquito-borne viral suitability index P, sensitive to temperature, humidity, and precipitation)[11,12] a vulnerability (proportion of the urban population without basic drinking water management),[13] an exposure (proportion of the population living below 2000 meters above sea level)[14], and a resilience (healthcare access and quality index)[15]

$$MoRI = \frac{threat \cdot vulnerability \cdot exposure}{resilience} = \frac{indexP \cdot WASH \cdot \%[0-2000]masl}{HAQ} \quad (1)$$

Index P is proposed by Lourenço and collaborators as a mathematical expression (Equation 2) for the basic reproductive number of mosquito-borne viruses. It can be interpreted as the absolute outbreak potential of an adult female mosquito, and whereas it has no direct interpretation on epidemics thresholds, it is informative regarding the timing and amplitude of transmission.

$$P_{(u,t)} = \frac{{a_{(u)}^{V}}^{2} \phi_{(t)}^{V\to h} \phi^{h\to V} \gamma_{(t)}^{V} \gamma^{h}}{\mu_{(u,t)}^{V} (\sigma^{h} + \mu^{h})(\gamma^{h} + \mu^{h})(\gamma_{(t)}^{V} + \mu_{(u,t)}^{V})}$$

Where four parameters are climate independent: the human life span $\frac{1}{\mu^h}$, the transmission probability from infected human to mosquito per bite $\phi^{h\to V}$, the human infectious period $\frac{1}{\sigma^h}$, and the incubation period $\frac{1}{\gamma^h}$. And

four parameters are climate (humidity u and temperature t) dependent: the life span of adult mosquitoes $\frac{1}{\mu^{V}_{(u,t)}}$, the extrinsic incubation period $\frac{1}{\gamma^{V}_{(u,t)}}$, the daily biting rate a^{V}_{u} and the probability of transmission from infected mosquito to human per bite $\phi^{V \to h}_{(t)}$. The MoRI is estimated yearly for 2000 to 2023 (missing data for later years was replicated using the last observation recorded), and the percentage of change is reported between the average index between 2021 and 2023 and the average of the last five and ten years. We used data available for Argentina, Bolivia, Brazil, Chile, Colombia, Costa Rica, Ecuador, El Salvador, Guatemala, Honduras, Mexico, Nicaragua, Panama, Paraguay, Peru, Uruguay, and Venezuela (drinking water data available for total population only). Comparison with cases was performed using the cases from the PLISA platform.[16]

Additional analysis

Table A2.9. Percentages of change of 2021-2023 MoRI compared to the 2000-2023, the 2014-2023, and the 2019-2023 average

Average MoVI 2021 - 2023		Percentage of Change compared to		
Country		Average 2000 - 2023	Average 2014 - 2023	Average 2019 - 2023
Argentina	0.13	-70.2%	-45.7%	-4.8%
Bolivia	0.10	-81.1%	-58.6%	-25.8%
Brazil	0.09	-82.4%	-53.8%	-2.6%
Chile	NA	NA	NA	NA
Colombia	0.05	-90.0%	-75.0%	-55.2%
Costa Rica	0.25	-60.0%	-23.1%	-2.5%
Ecuador	0.00	-100.0%	-100.0%	NA
El Salvador	0.02	-95.1%	-80.6%	-50.4%
Guatemala	0.47	-33.3%	-12.7%	-3.3%
Honduras	0.27	-59.4%	-34.0%	-10.9%
México	0.00	-100.0%	-100.0%	-100.0%
Nicaragua	0.55	-22.0%	0.7%	0.7%
Panama	0.59	-27.7%	-14.2%	-1.6%
Paraguay	0.02	-95.7%	-79.8%	-1.2%
Peru	0.27	-55.7%	-30.8%	-10.6%
Uruguay	0.21	-55.6%	-22.6%	-1.8%
Venezuela	0.97	32.1%	7.5%	2.7%

2.3.2: lethality of extreme weather events

Regional author(s)

Yasna Palmeiro, Camila Llerena

Methods

This indicator follows the same methodology as the 2025 global Lancet Countdown report.

Additional analysis

This indicator has no additional analyses.

Caveats and limitations

The mortality rate presented in this indicator is calculated using the total national population, rather than the population actually exposed to extreme weather events, which may lead to under- or overestimations of lethality. Additionally, the underlying database relies on self-reported information, which tends to underrepresent Latin America due to known issues of underreporting in low- and middle-income countries. For a detailed description of the methodological limitations of the data source, please refer to the Appendix of the global Lancet Countdown report.

Section 3: mitigation actions and health co-benefits

3.1: energy use, energy generation, and health

3.1.1: energy system and health

Regional author(s)

Enzo Sauma, Yasna Palmeiro Silva

Methods

This indicator follows the same methodology as the 2025 global *Lancet* Countdown report.

The following types of coal are added to produce the total primary coal supply: 'Anthracite', 'Coking coal', 'Lignite', 'Other bituminous coal', 'sub-bituminous coal'.

The following International Energy Agency (IEA) variable names are added to produce total low carbon electricity generation: 'Nuclear, 'Hydro', 'Geothermal', 'Solar photovoltaics', 'Solar thermal', 'Tide, wave and ocean', 'Wind'.

The following IEA variable names are added to produce total modern renewable electricity generation: 'Geothermal', 'Solar photovoltaics', 'Solar thermal', 'Tide, wave and ocean', 'Wind'.

Additional analysis

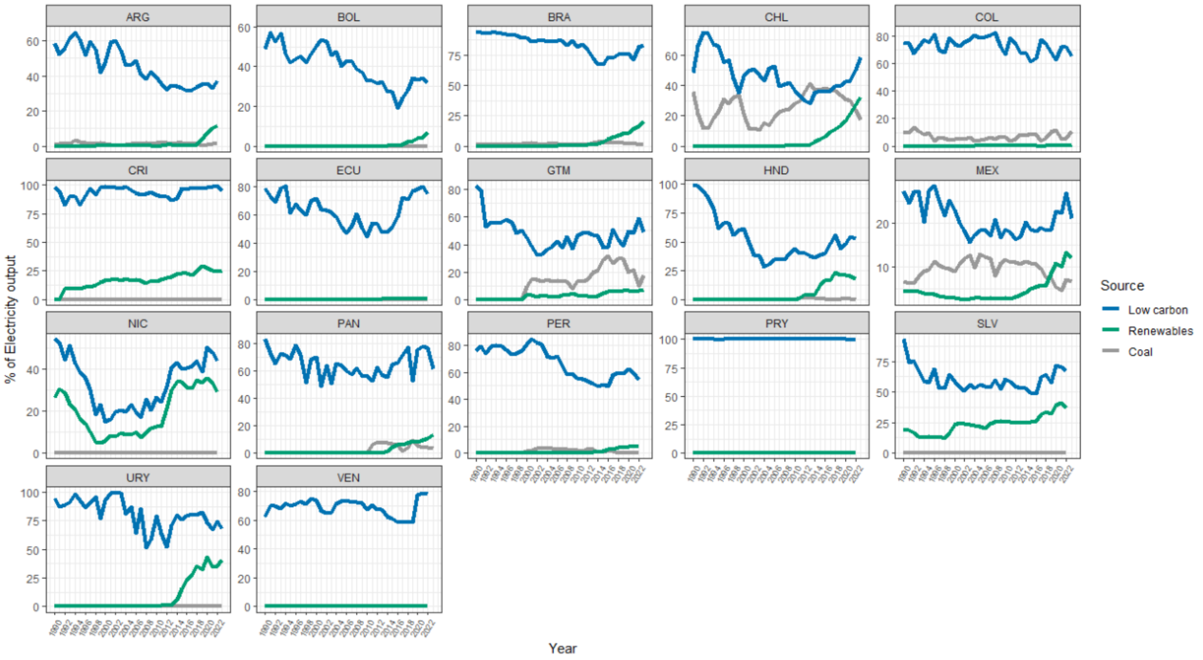

Figure A3.1. Percentage of electricity generation by source: low carbon, renewables, coal, and others, from 1990 to 2023, by Latin American country.

3.1.2: household energy use

Regional author(s)
Luciana Blanco, Stella Hartinger

Methods

This indicator is based on data from the World Health Organization's Sustainable Development Goal 7 (SDG 7) database,[17] which monitors the percentage of the population with access to clean fuels and technologies for cooking, such as electricity, gas (e.g., natural gas and liquefied petroleum gas), and other clean sources, following WHO air quality guidelines. In the dataset used, access to clean cooking energy is reflected in three variables: Gas, Electricity, and Other Clean (all considered clean cooking fuels according to WHO). In addition to tracking access to clean fuels, the dataset also reports on the use of polluting cooking fuels, which include biomass (e.g., wood, dung, crop waste), charcoal, coal, solid fuels, and kerosene.

Based on the WHO's methodology, *"the indicator is calculated as the number of people using clean fuels and technologies divided by total population, expressed as a percentage. Based on the recommendations included in the WHO Guidelines for indoor air quality: household fuel combustion, the fuels and technologies that are considered clean include electricity, natural gas, liquified petroleum gas, biogas, ethanol, and solar"*.

For this analysis, access to clean cooking fuels was examined across Latin American countries from 1990 to 2023. The data were first analyzed using national (overall) averages and grouped by subregion (Central and South America). Further disaggregation was performed by area of residence (urban and rural) to assess within-country disparities. In addition, country-level trends in the use of biomass and gas for cooking were visualized over time and by region to understand better patterns in the adoption of clean versus polluting fuels.

Data

World Health Organization's Sustainable Development Goal 7

Additional analysis
An additional analysis was conducted to examine differences in the use of biomass and gas for cooking between urban and rural populations across Latin American countries from 1990 to 2023. The analysis focused on two key fuels: biomass, a common source of polluting energy, and gas, which, although often considered a transitional fuel toward cleaner alternatives, remains a fossil fuel and a significant contributor to both household and climate-related air pollution. The analysis also examined trends in the use of clean cooking fuels (electricity, gas, and other clean sources) based on WHO criteria, focusing on the overall population across countries.

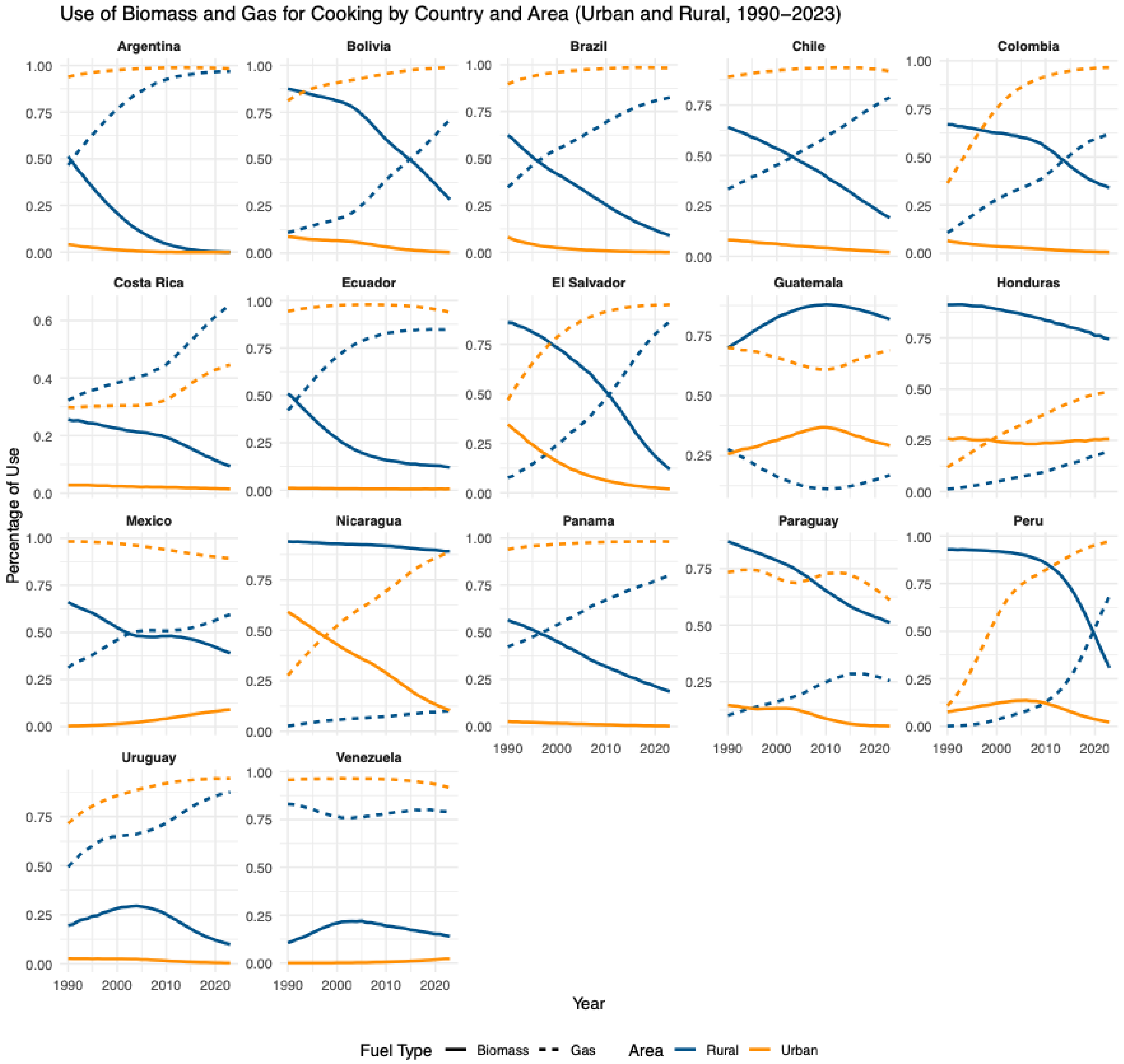

Figure A3.2. Use of Biomass and Gas for Cooking by Country, Area and Year (1990–2023)

Figure A3.2 shows the continued persistence of biomass use in rural areas. This trend is particularly evident in Central American countries such as Nicaragua, Guatemala, and Honduras, where biomass use in rural areas has remained consistently high throughout the period from 1990 to 2023, in some cases exceeding 75%. Even in 2023, biomass remains the primary cooking fuel in many rural parts of the region.

A pronounced urban–rural gap is also evident. In most countries, biomass use is significantly lower in urban areas and exhibits a more pronounced downward trend over time. Gas has become the dominant cooking fuel in urban settings since the early 2000s, with usage rates of 80% or higher in countries such as Chile, Uruguay, and Argentina.

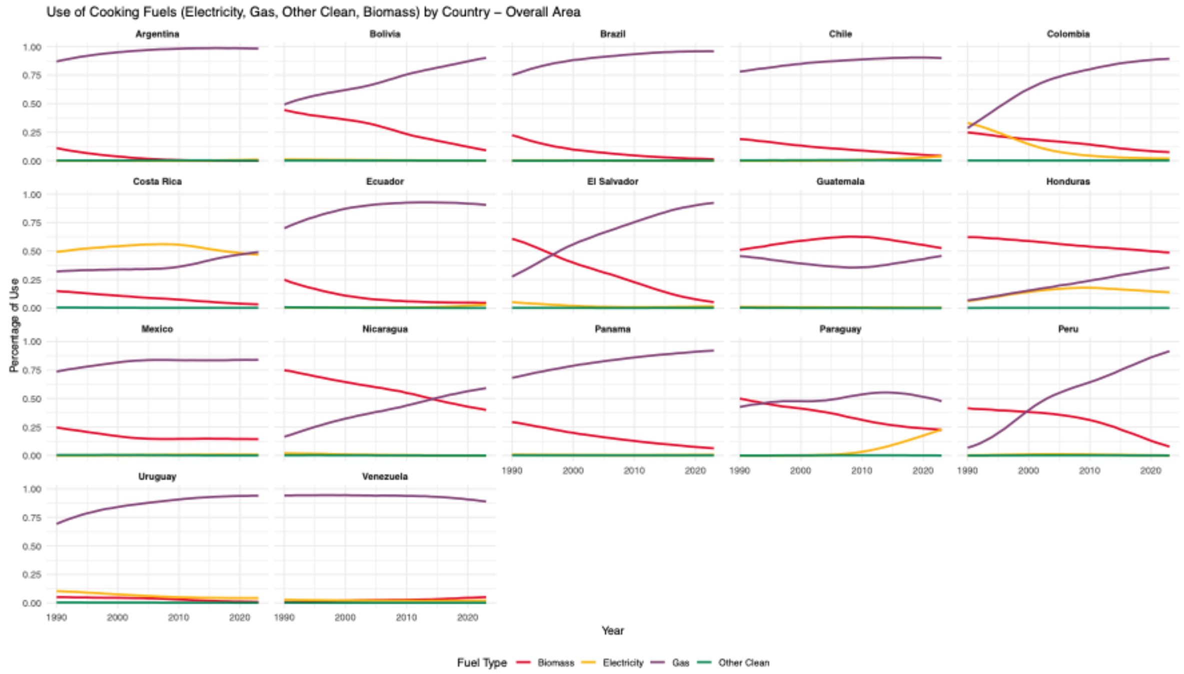

Figure A3.3. Use of Clean Cooking Fuels (Gas, Electricity, Other Clean) by Country – Overall Area (1990–2023)

In Figure A3.3, an analysis of clean energy use based on WHO criteria shows that most countries in the region continue to transition toward greater reliance on gas, a fossil fuel that, although often considered a transitional option, remains a source of pollution. Only Paraguay exhibits a clear trend of increasing electricity use alongside a reduction in gas use. No significant growth is observed in the adoption of other clean energy sources.

3.1.3: sustainable and healthy road transport

Regional author(s)
David Rojas, Yasna Palmeiro Silva

Methods

This indicator follows the same methodology as the 2025 global *Lancet* Countdown report.

Additional analysis

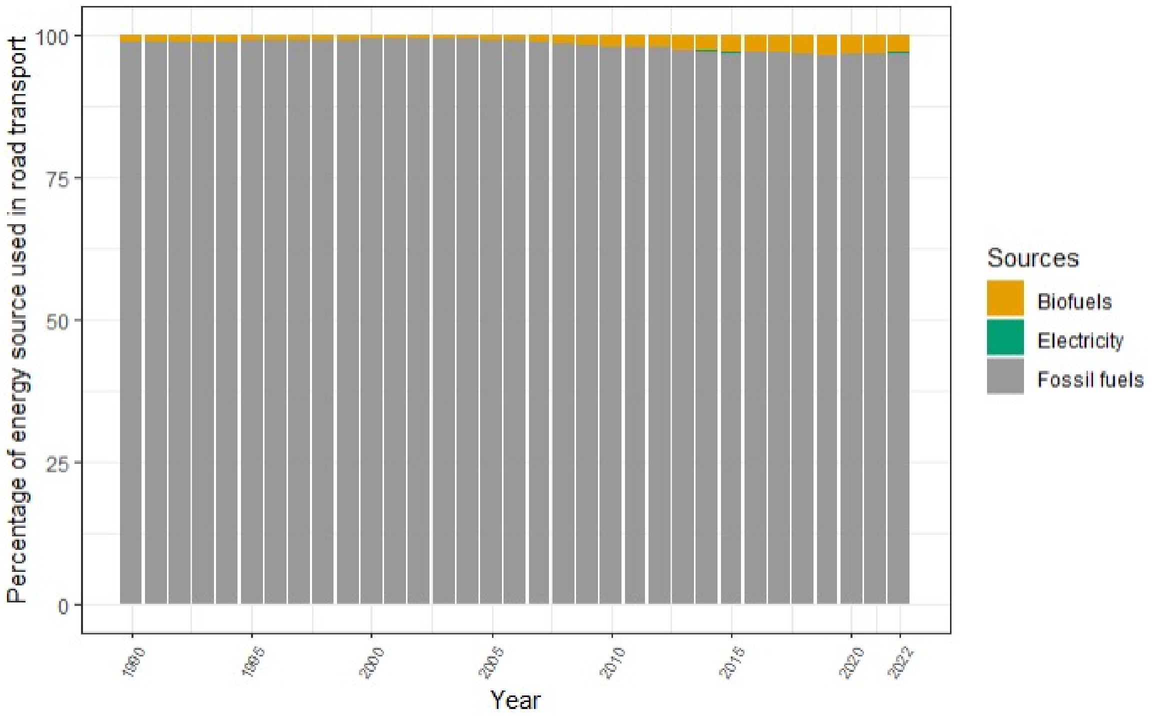

Figure A3.4. *Percentage of energy source used in road transport in Latin America by source, from 1990 to 2022*

3.2: air pollution and health co-benefits

3.2.1: premature mortality from ambient air pollution

Regional author(s)

Magali Hurtado

Methods

Table A3.1. The indicator covers 11 Latin American countries.

Countries included in the indicator	Argentina, Bolivia, Brasil, Chile, Colombia, Ecuador, México, Paraguay, Perú, Uruguay, Venezuela
Countries not included in the indicator	Belice, Costa Rica, El Salvador, Guatemala, Honduras, Nicaragua, Panamá, Guyana, Surinam,

This indicator uses a Greenhouse Gas and Air Pollution Interactions and Synergies (GAINS) model to estimate premature deaths attributable to $PM_{2.5}$ long-term exposure from different fuels. The indicator follows the same methodology as the 2025 global Lancet Countdown report.

Additional analysis

Further analysis presents data on premature mortality attributable to $PM_{2.5}$ from coal, liquid gas, and biomass combustion, analyzing trends across Latin America from 2007-2022 through the lens of Human Development Index (HDI) classifications.

Due to the heterogeneity of mortality rate estimates among countries, data were analyzed by grouping countries according to the Human Development Index (HDI). Two of the countries included in this analysis are classified in the Medium HDI band, seven in the High and Three countries in the Very High HDI group.

Between 2007-2022, Latin American countries achieved mixed results in reducing premature mortality from air pollution: deaths from coal and liquid gas combustion decreased by 10.2% and 8.7% respectively, while biomass-related mortality increased by 23.9%.

Despite ongoing reductions since 2007, liquid gas combustion remained the dominant contributor to $PM_{2.5}$-related mortality in Latin America in 2022, responsible for 67% of these deaths. The most significant reduction occurred in 2020 when COVID-19 lockdowns dramatically curtailed demand, particularly benefiting Medium HDI countries.

The overall regional increase (2007-2022) in biomass-related mortality varied substantially by development level. High HDI countries drove this increase, whereas Very High HDI nations like Chile and Argentina showed marked improvements after 2014, coinciding with policies replacing inefficient wood-burning heaters with cleaner alternatives (Figure A3.5).

Coal combustion contributed minimally to $PM_{2.5}$-related mortality across most Latin American countries and continued to decline during the study period. However, Chile and Colombia diverged from this pattern, showing concerning increases in coal-related mortality rates over the 15-year period.

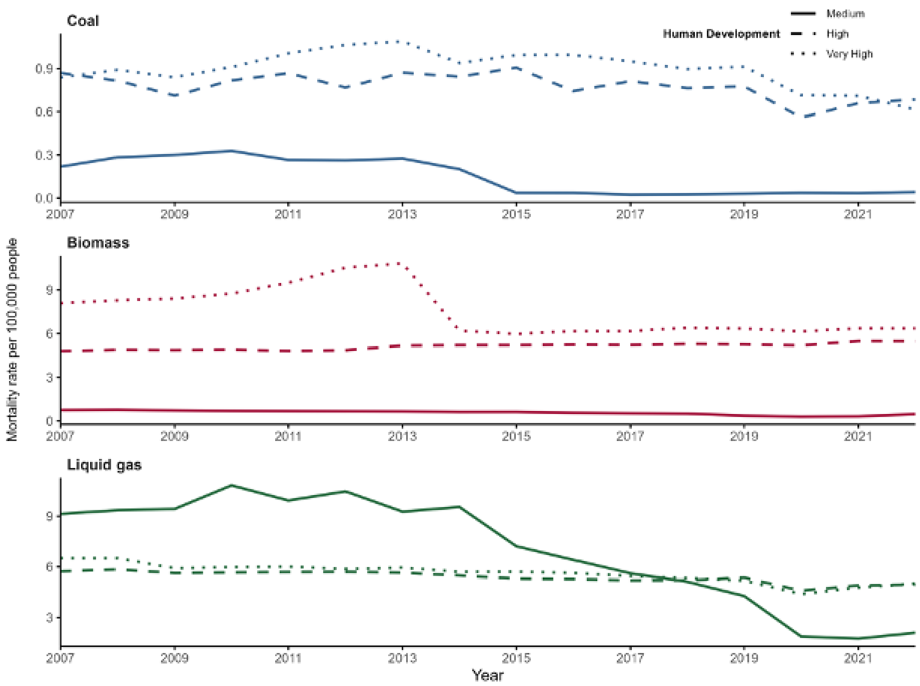

Figure A3.5. Annual estimates of mortality rate per 100,000 people from $PM_{2.5}$ air pollution exposure in countries grouped by HDI and according to fuel source in Latin America. Data from 2007-2022.

From 2007 to 2022, PM2.5 attributable deaths (per 100,000 people) from liquid gas combustion have a negative trend, especially in the last decade. However, from 2021 to 2022, premature mortality from liquid gas combustion increased by 11.6%, for both power plants and industry. In the case of PM2.5 attributable mortality from biomass combustion in the household sector, the same negative trend was observed in recent years, followed by a 24.8% increase (Figure A3.6).

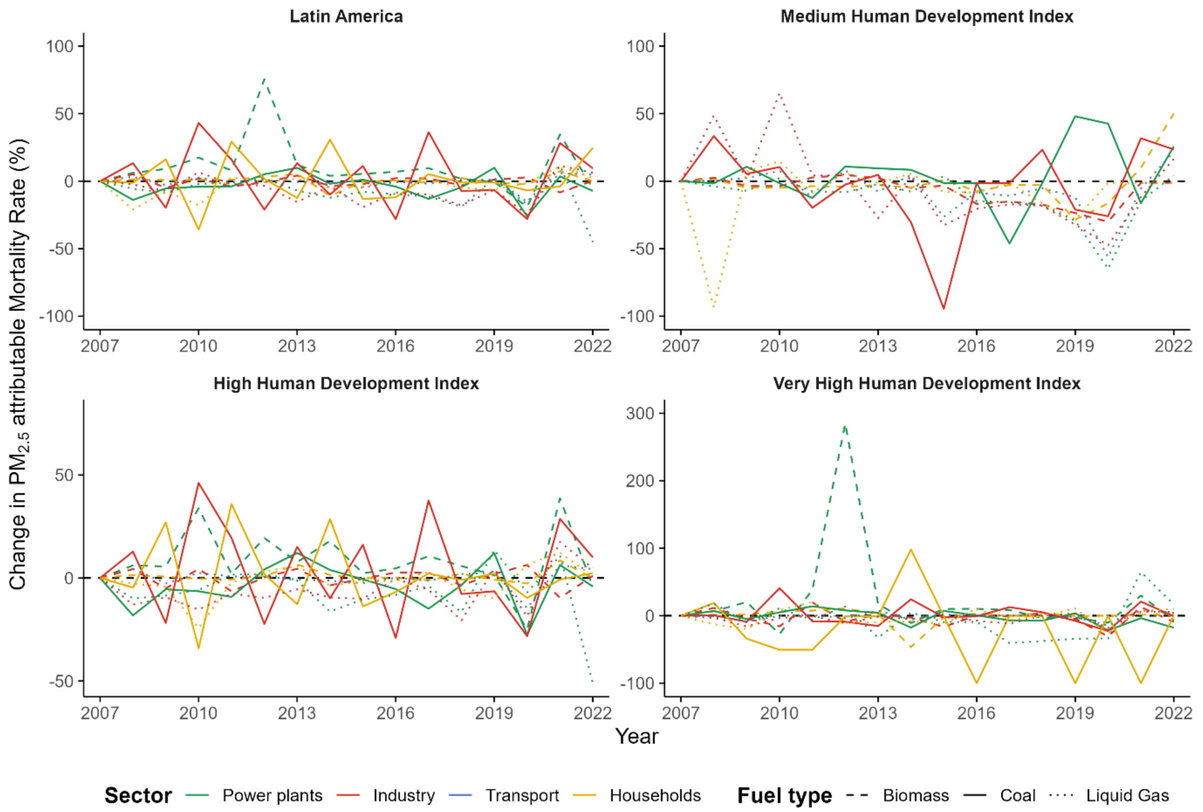

Figure A3.6. Changes in estimates of mortality rate attributable to $PM_{2.5}$ derived of coal, liquids gas and biomass combustion from economic sectors in countries grouped by HDI in Latin American. Data from 2007 to 2022. [a] Percentage change year over year

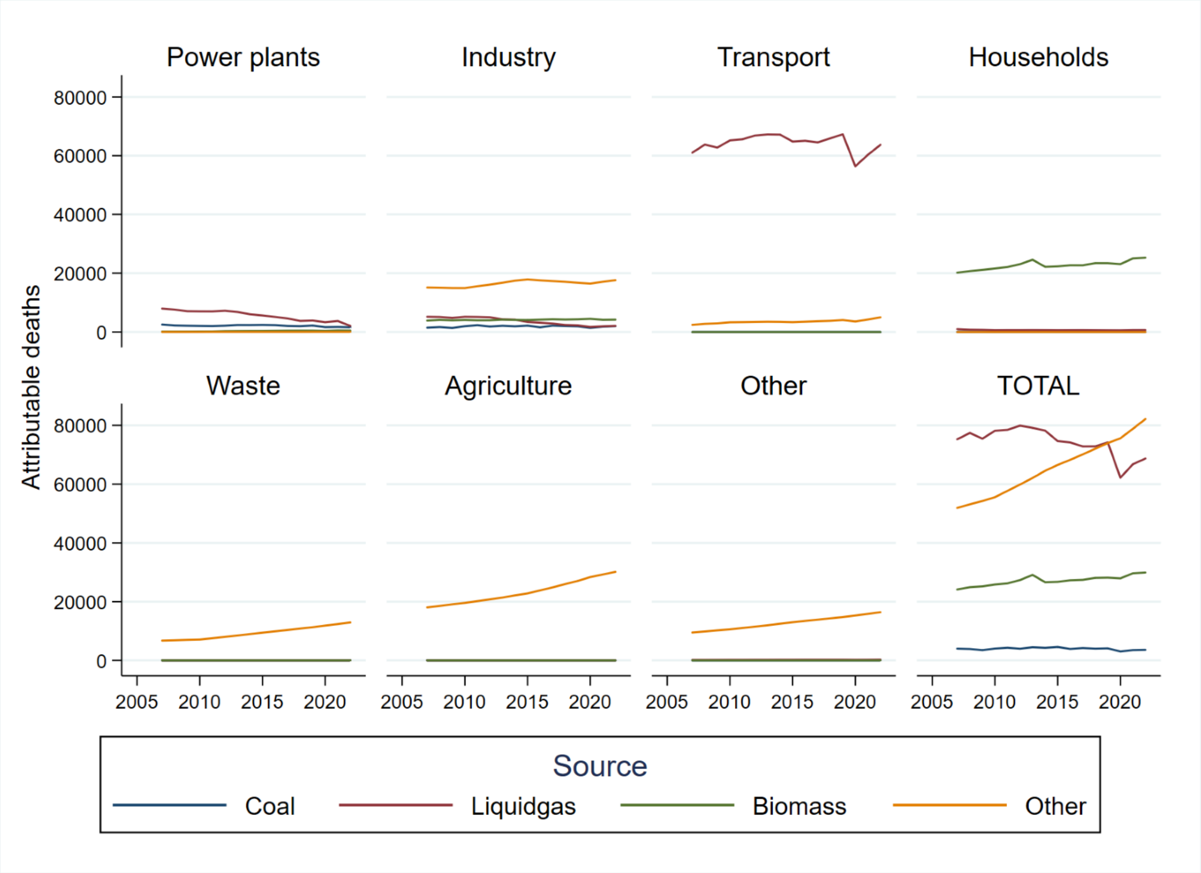

Figure A3.7. Annual attributable deaths per sector and fuel, from 2007 to 2022

3.2.2: exposure to household air pollution

Regional author(s)

Nahid Mohajeri

Methods

This indicator follows the same methodology as the 2025 global *Lancet* Countdown report. The analyses covered six South American countries (Bolivia, Ecuador, Peru, Argentina, Venezuela, and Colombia) and six Central American countries (Nicaragua, El Salvador, Mexico, Guatemala, Honduras, and Costa Rica).

Additional analysis

There are no additional analyses for this indicator.

3.3: food, agriculture, and health co-benefits

3.3.1: emissions from agricultural production and consumption

Regional author(s)

Tatiana de Camargo, Aline Martins de Carvalho

Methods

This indicator follows the same methodology as the 2025 global *Lancet* Countdown report.

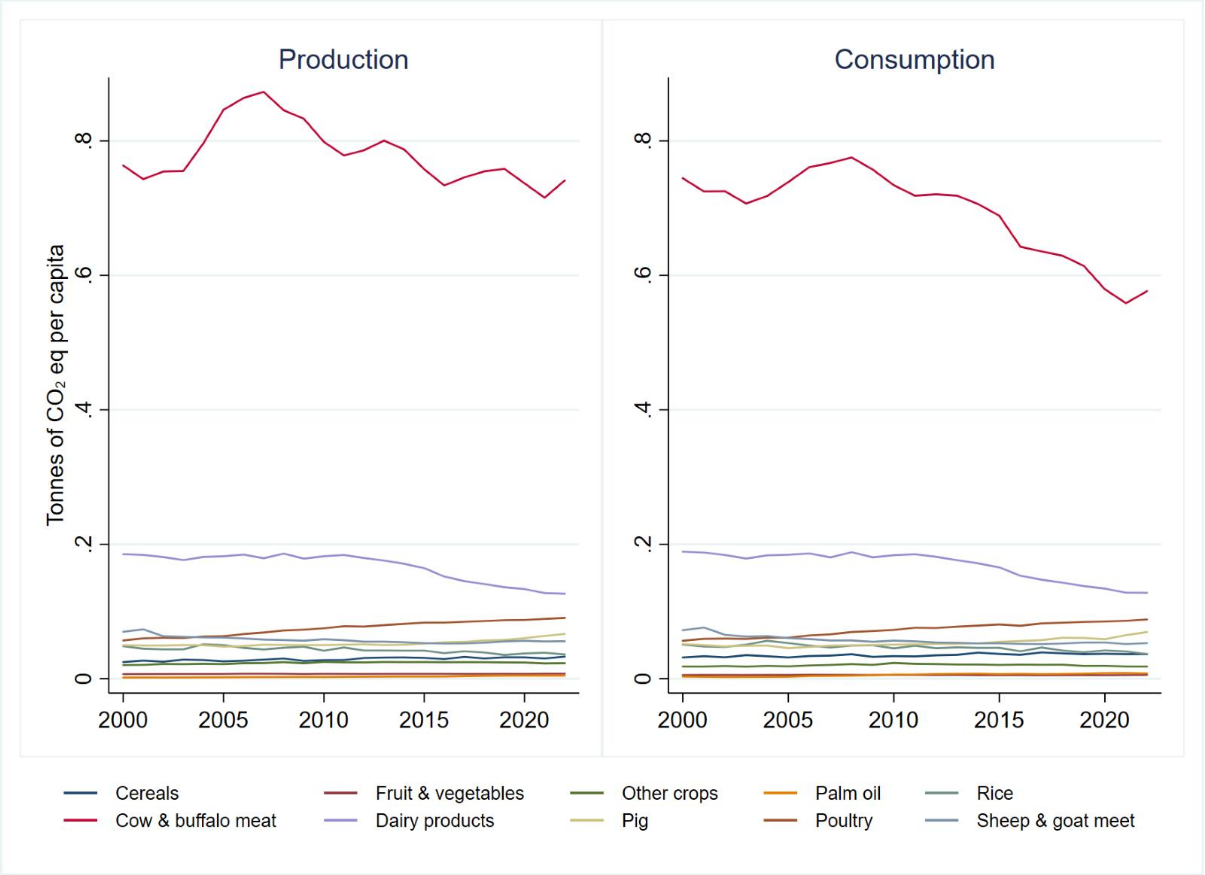

Figure A3.8. Tonnes of CO2e per capita per commodity for A) consumption and B) production, in Latin America, from 2000 to 2022.

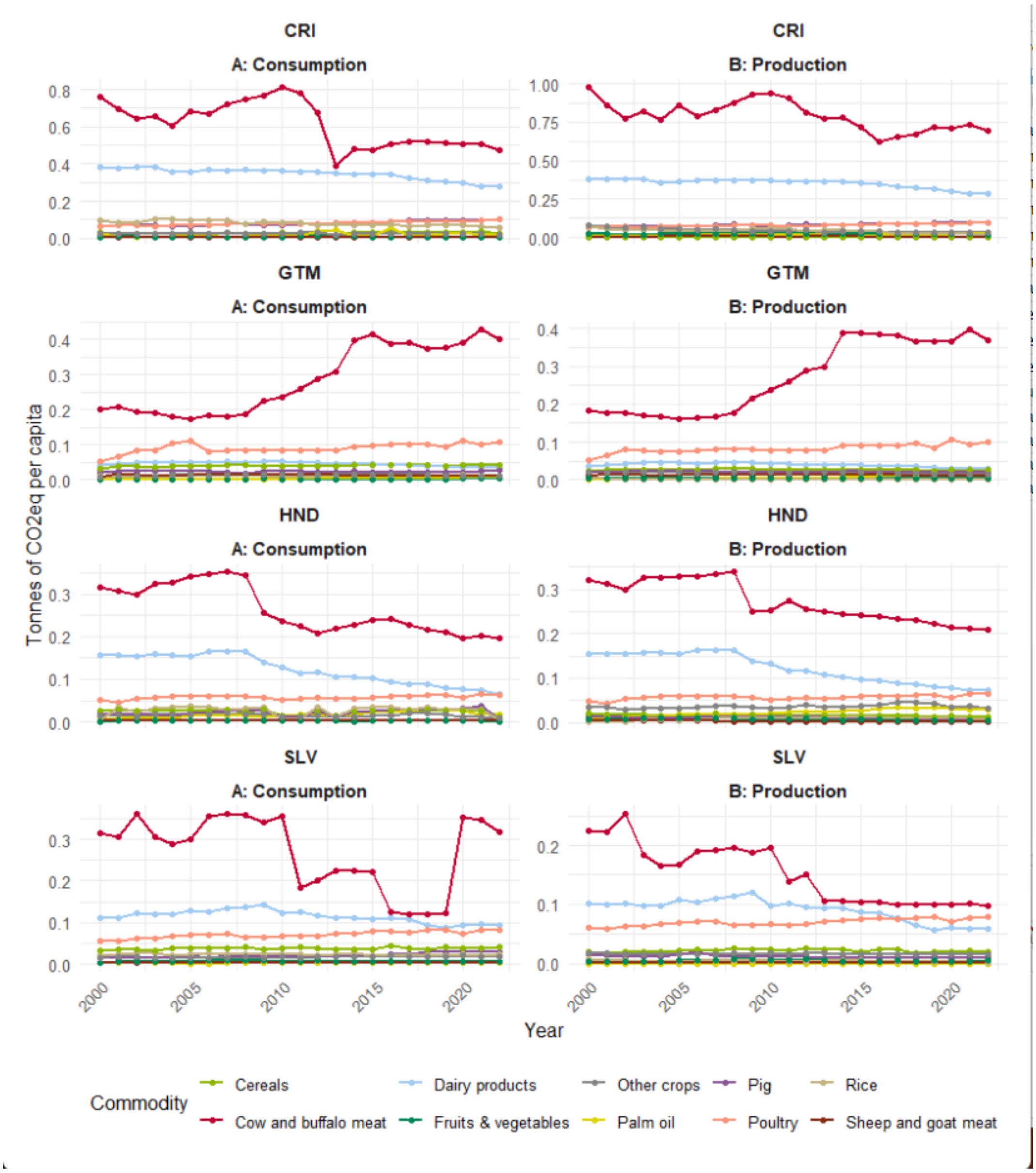

Figure A3.9. Tonnes of CO_2e emissions per capita from agricultural consumption and production in Latin America between 2000 and 2022.

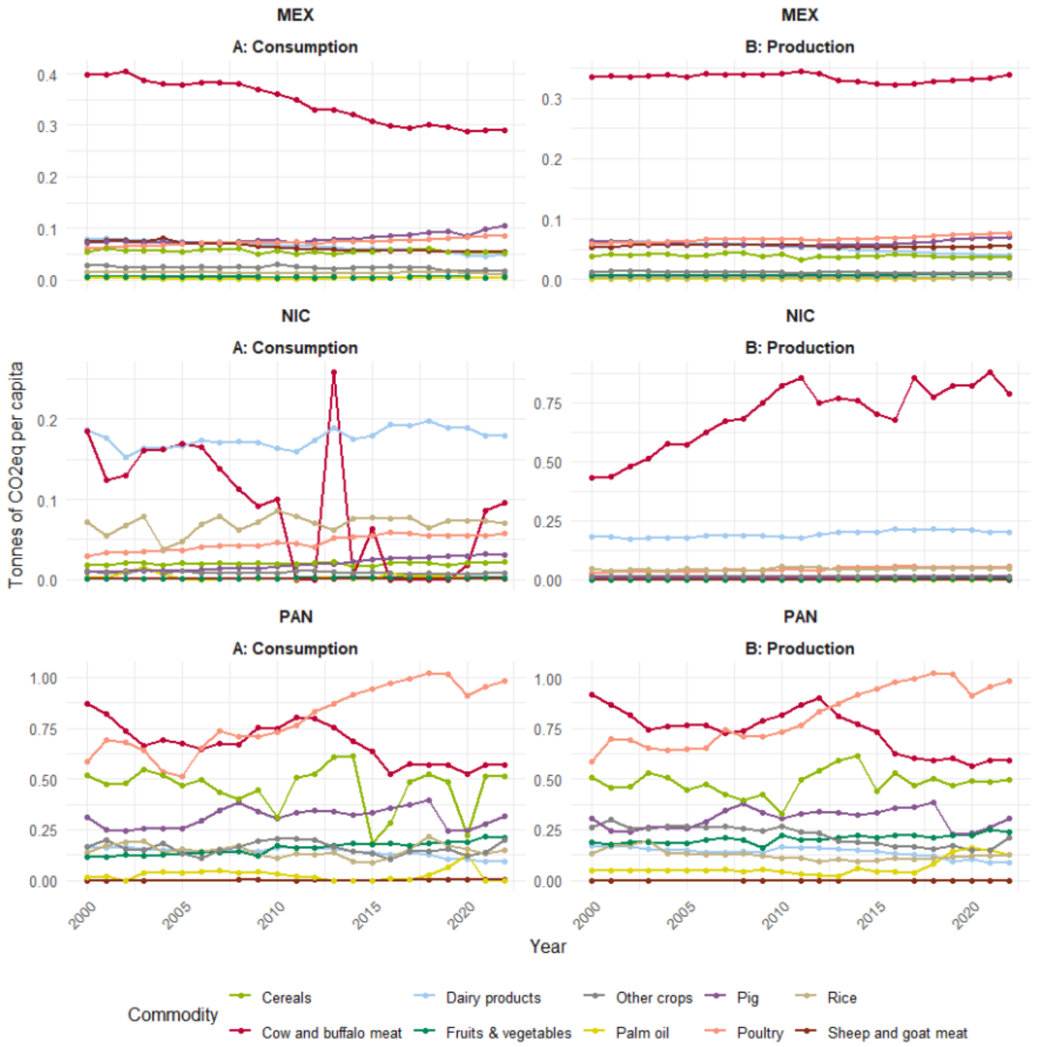

Figure A3.9. (cont) Tonnes of CO_2e emissions per capita from agricultural consumption and production in Latin America between 2000 and 2022.

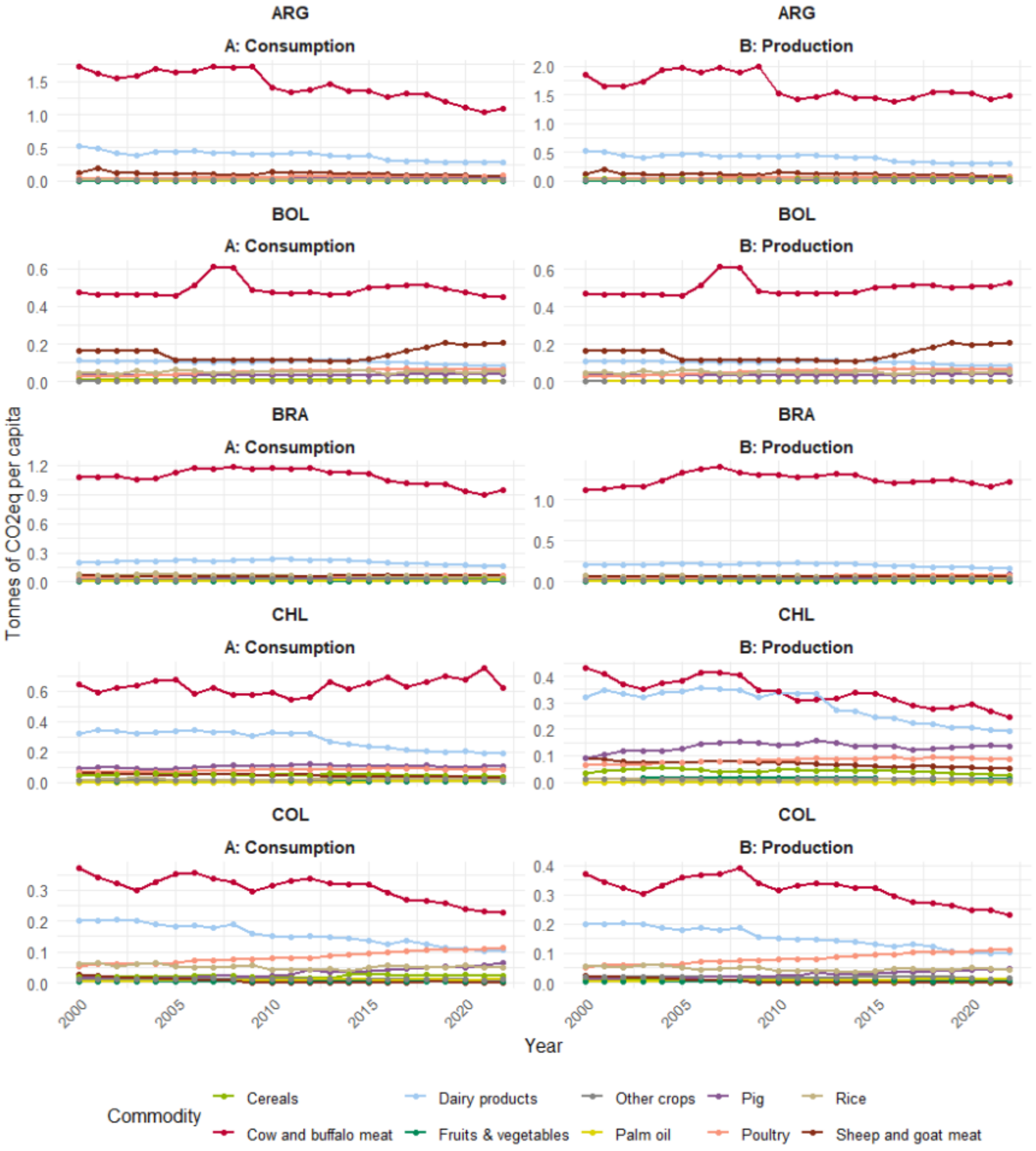

Figure A3.9. (cont) Tonnes of CO_2e emissions per capita from agricultural consumption and production in Latin America between 2000 and 2022.

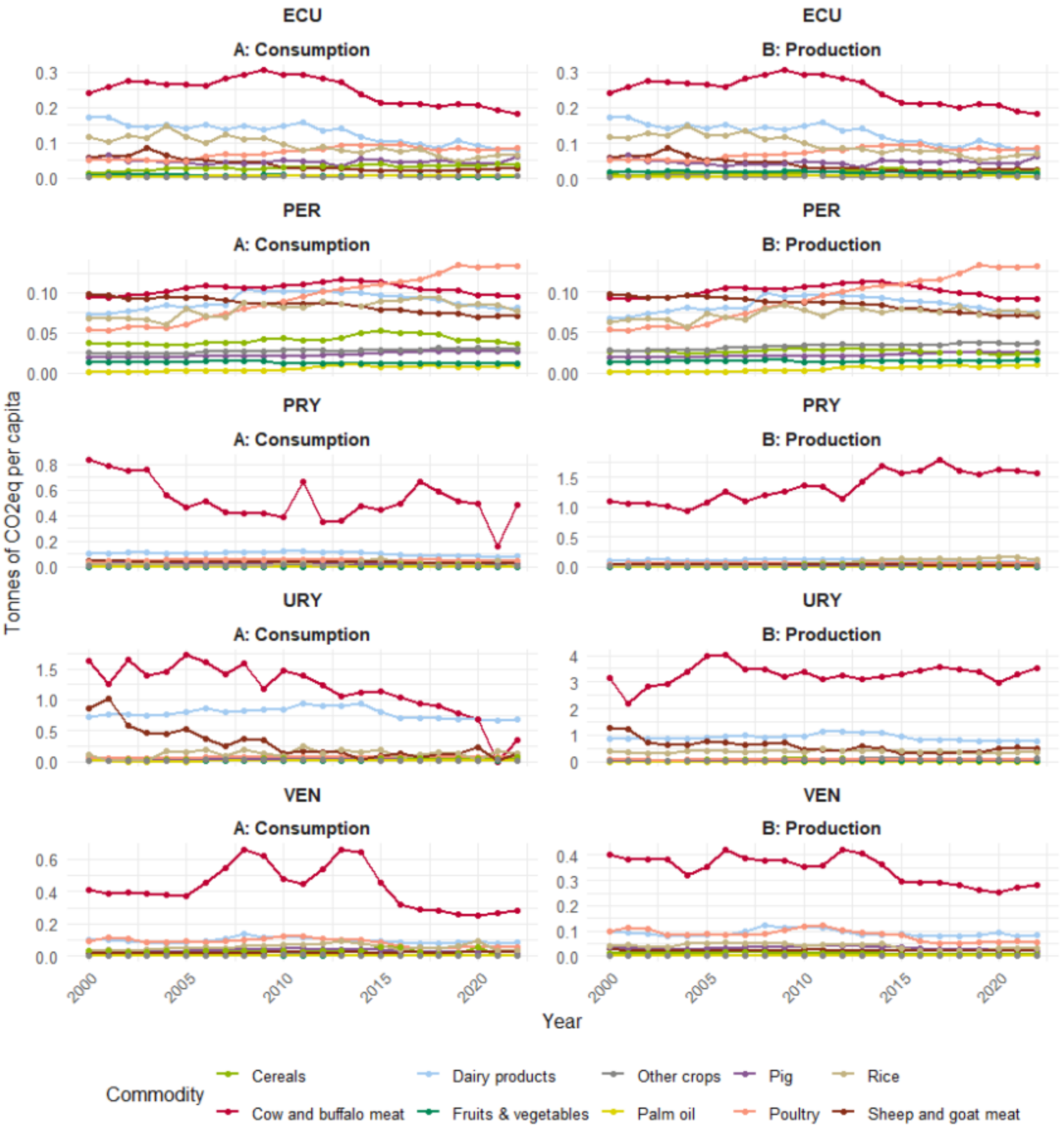

Figure A3.9. (cont) Tonnes of CO_2e emissions per capita from agricultural consumption and production in Latin America between 2000 and 2022.

3.3.2: diet and health co-benefits

Regional author(s)
Raquel Santiago

Methods

This indicator follows the same methodology as the 2025 global *Lancet* Countdown report.

Additional analysis

No additional analyses are included for this indicator.

3.4: tree cover loss and health

Regional author(s)

David Rojas Rueda, Nicolas Valdes

Methods

The rapid loss of tree cover is a pressing concern with profound implications for climate change, biodiversity, and human health. Forests are integral in stabilizing the climate, preserving unique ecosystems, and supporting human livelihoods. The decline of tree cover augments carbon emissions and limits carbon sink, intensifies global warming, and disrupts the habitat of countless species, which may also lead to increased disease transmission. This indicator evaluates tree cover loss in hectares using different data sources.[18,19]

The study used high-resolution Google Earth imagery to visually classify nearly 5,000 training sample cells. A decision-tree model was developed to predict the most likely cause of forest disturbance at any 10 km × 10 km grid cell globally from 2001 to 2015. The model distinguished between permanent conversion (deforestation) and temporary loss due to forestry or wildfire. The model's overall accuracy was 89%, with individual class accuracies ranging from 55% (urbanization) to 94% (deforestation). The methodology combined the accuracy and estimates of a sample-based approach with the spatial comprehensiveness of a wall-to-wall mapping approach.

The dataset classifies loss into five categories:

- *commodity-driven deforestation*, defined by the long-term, permanent conversion of forest and shrubland to a non-forest land use such as agriculture (including oil palm), mining, or energy infrastructure;
- *shifting agriculture*, defined as small- to medium-scale forest and shrubland conversion for agriculture that is later abandoned and followed by subsequent forest regrowth;
- *forestry*, defined as large-scale forestry operations occurring within managed forests and tree plantations with evidence of forest regrowth in subsequent years;
- *wildfire*, defined as large-scale forest loss resulting from the burning of forest vegetation with no visible human conversion or agricultural activity afterward; and
- *urbanization*, defined as forest and shrubland conversion for the expansion and intensification of existing urban centres. It is essential to note the distinction between temporary loss and permanent deforestation. Only commodity-driven deforestation and urbanization result in permanent loss. While this dataset offers valuable insights at the global and regional scale, care must be taken when interpreting results for smaller areas due to potential inaccuracies.

As limitation, the model does not include disturbances like insect outbreaks, wind and ice storms, flooding, or rivers changing course. Also, it struggled to distinguish between shifting agriculture and commodity-driven deforestation in sub-Saharan Africa due to similar spatial patterns.

The temporal and geographical coverage are:

- Temporal Coverage: The dataset covers the period from 2002 to 2022.

- Geographical Coverage: The coverage is global, with a specific focus on different regions such as North America, Latin America, Southeast Asia, and sub-Saharan Africa.

Databases

The primary data sources are datasets from Landsat satellite observations and Google Earth imagery.[18,19]

Collection Method: The study uses satellite imagery to develop a forest loss classification model, distinguishing between permanent forest conversion (deforestation) and temporary loss due to forestry or wildfire.[18,19]

Additional analyses

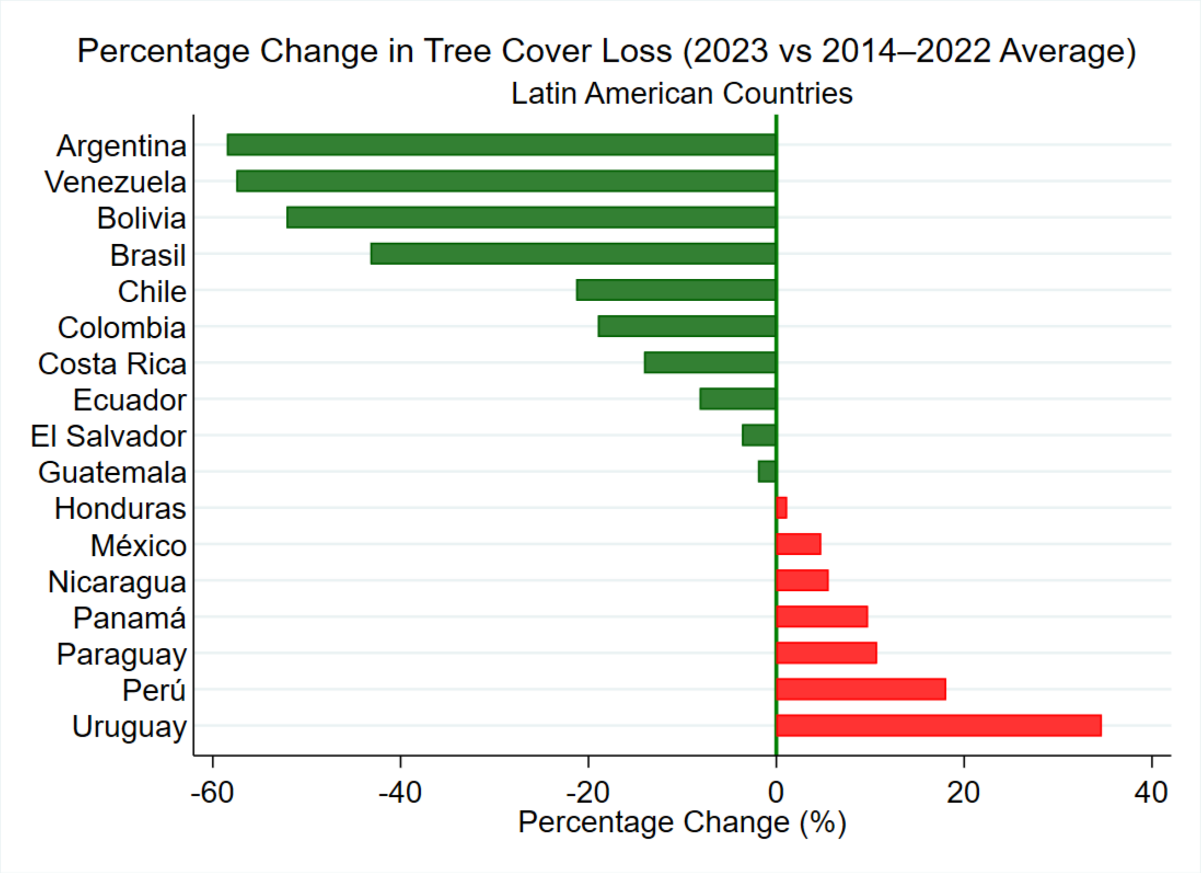

Figure A3.10. Percentage change tree cover loss 2023 vs average 2014-2022, Latin America

Caveats

Exclusion of Certain Disturbances: The model does not include disturbances such as insect outbreaks, wind and ice storms, flooding, or rivers changing course. These disturbances were found to be highly localized and temporally restricted, affecting only 1% of all model validation sample cells.

Misclassification Issues: There was low model accuracy for the commodity-driven deforestation class in Africa, with much of this deforestation misclassified as shifting agriculture. In northern forests, especially in Russia, distinguishing between drivers was challenging in areas where wildfires spread through previously logged areas or where logging occurred after a fire event.

Lack of Detailed Differentiation: The study did not map changes in forest conditions over time in landscapes dominated by shifting agriculture, nor did it differentiate primary from secondary forest clearing within this land-use class. Differentiating key drivers like row crops from pasturelands in South America or tree plantations from disturbed natural forests in Southeast Asia could enhance the analysis.

3.5: healthcare sector emissions

Regional author(s)

Nicolas Valdes, Yasna Palmeiro Silva

Methods

This indicator follows the same methodology as the 2025 global *Lancet* Countdown report.

Additional analysis

This indicator summarises healthcare system emissions, according to GHGs, ozone, and $PM_{2.5}$. It is derived using a top-down, spend-based approach that integrates health expenditure data with the environmentally-extended multi-region input-output (EE-MRIO) model EXIOBASE. For each country, we estimated the percentage change in average emissions for the period 2018-2022, using 2010-2014 as reference.

Section 4: economics and finance

4.1: The economic impact of climate change and its mitigation

4.1.1: Economic losses due to weather-related extreme events

Regional author(s)
Christian García-Witulski

Methods

Economic losses were obtained from the EM-DAT International Disaster Database, which reports direct damage for each registered disaster in thousands of constant-2024 US dollars. All extreme weather-related events (storms, floods, hail, droughts, wildfires and extreme-temperature episodes) recorded between 2010 and 2024 for the 17 Latin-American countries included in this report were selected. Reported damage was converted to billions of 2024 US dollars by dividing each loss figure by 1 000 000 and then summed by country and calendar year; regional losses are the simple sum of the 17 country totals.

Nominal GDP series (billions of current US dollars) for the same period were taken from the IMF World Economic Outlook (April 2025, indicator "Gross domestic product, current prices"). Regional GDP is the aggregate of the 17 national series. The indicator expresses losses as a share of economic output by dividing regional losses (billions of 2024 US$) by regional GDP (billions of current US$) and multiplying by 100. Five-year unweighted means—2010-2014 and 2018-2024—are reported to smooth the marked year-on-year variability that characterises disaster losses, following the precedent of previous editions of this annex. Loss estimates refer only to direct damage; indirect knock-on effects and the split between insured and uninsured losses cannot be derived from EM-DAT and are therefore not included.

Table A4.X. Data sources, unit conversions and calculation steps for the economic-loss indicator

Data source	Variable used	Original units	Transformation	Role in indicator
EM-DAT International Disaster Database (accessed May 2025)	Total Damage, Adjusted ('000 US$) for all weather-related disasters	Thousands of constant-2024 US$	÷ 1 000 000 → billions of 2024 US$; summed by country-year and region-year	Numerator (economic losses)
IMF *World Economic Outlook*, April 2025	Gross domestic product, current prices	Billions of current US$	None (already in billions)	Denominator (regional GDP)
Calculation	—	—	Losses ÷ GDP × 100	Express losses as % of GDP
Calculation	—	—	Simple unweighted means for 2010-2014 and 2018-2024	Five-year averages to reduce inter-annual volatility

Additional analysis

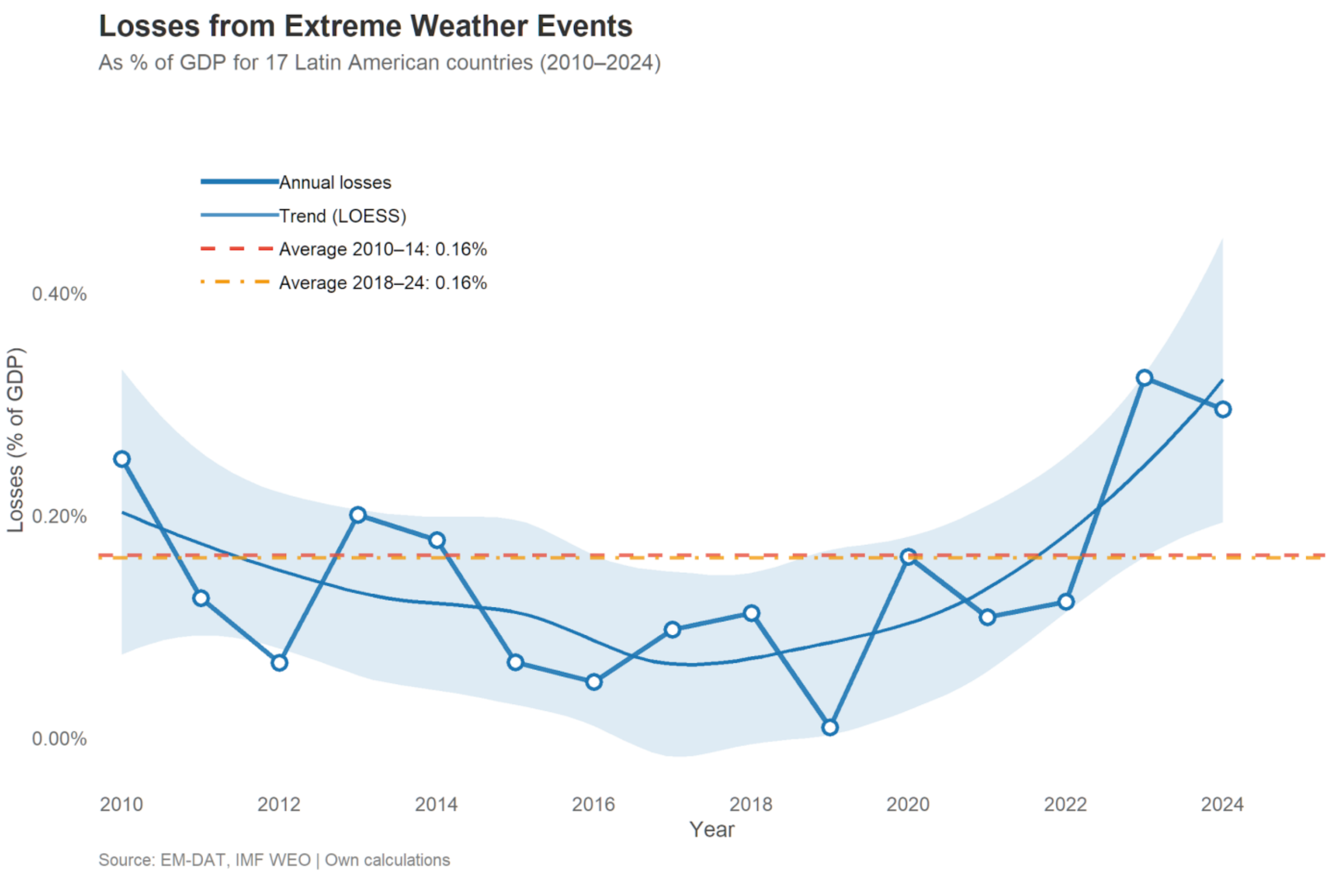

Figure A4.1. Extreme weather-related economic losses in Latin America, 2010-2024 (% of regional GDP)

4.1.2: Costs of heat-related mortality

Regional author(s)

Yasna Palmeiro Silva, Christian García-Witulski

Methods

This indicator follows the same methodology as the 2025 global Lancet Countdown report.

Additional analysis

There are no additional analyses for this indicator.

4.1.3: Loss of earnings from heat-related labour capacity reduction

Regional author(s)

Christian García-Witulski, Chrissie Pantoja

Methods

This indicator follows the same methodology as the 2025 global Lancet Countdown report.

Additional analysis

There are no additional analyses for this indicator.

4.1.4: costs of the health impacts of air pollution

Regional author(s)

Christian García-Witulski, Chrissie Pantoja

Methods

This indicator follows the same methodology as the 2025 global *Lancet* Countdown report.

Additional analysis

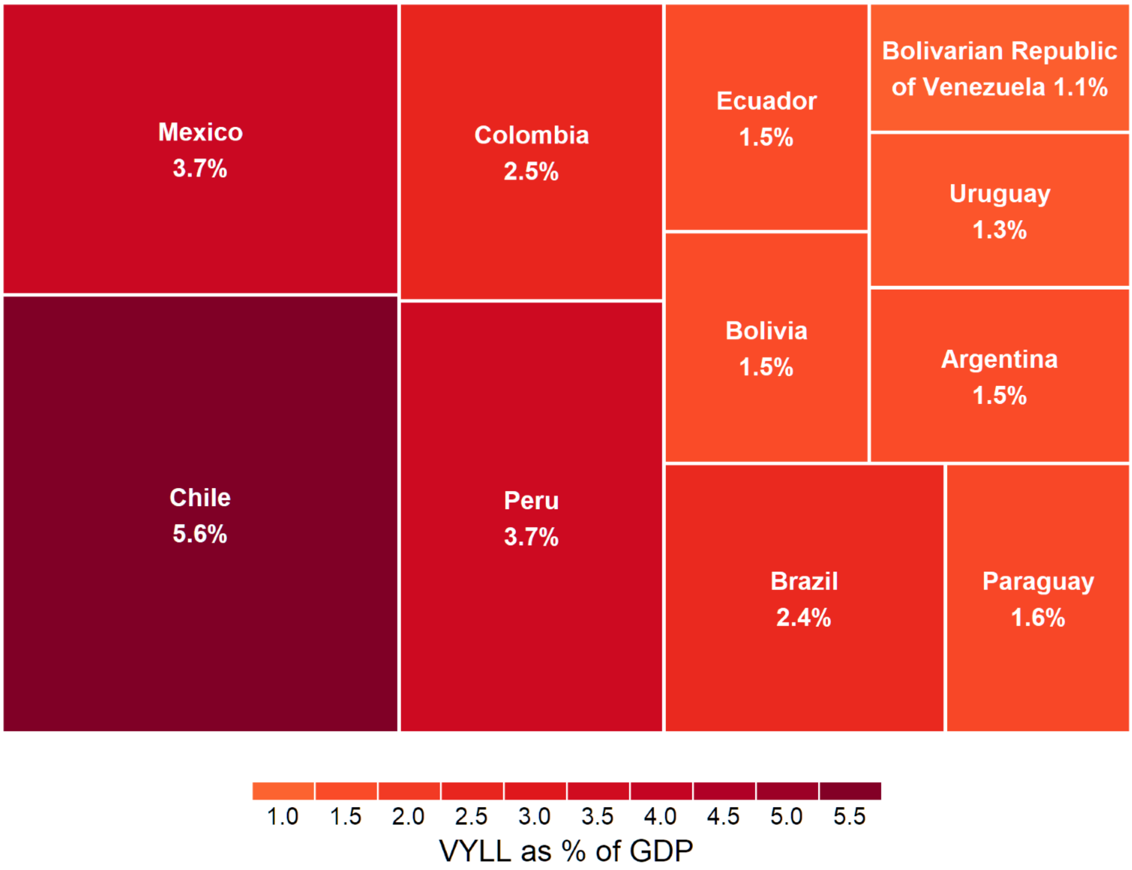

Figure A4.2. Value of Years of Life Lost due to air pollution-related health impacts as a percentage of GDP in 11 Latin American countries (2022)

4.2: The transition to net zero-carbon, health-supporting economies

4.2.1: country preparedness for the transition to net zero

Regional author(s)

Christian García-Witulski

Methods

This indicator follows the same methodology as the 2025 global Lancet Countdown report.

Additional analysis

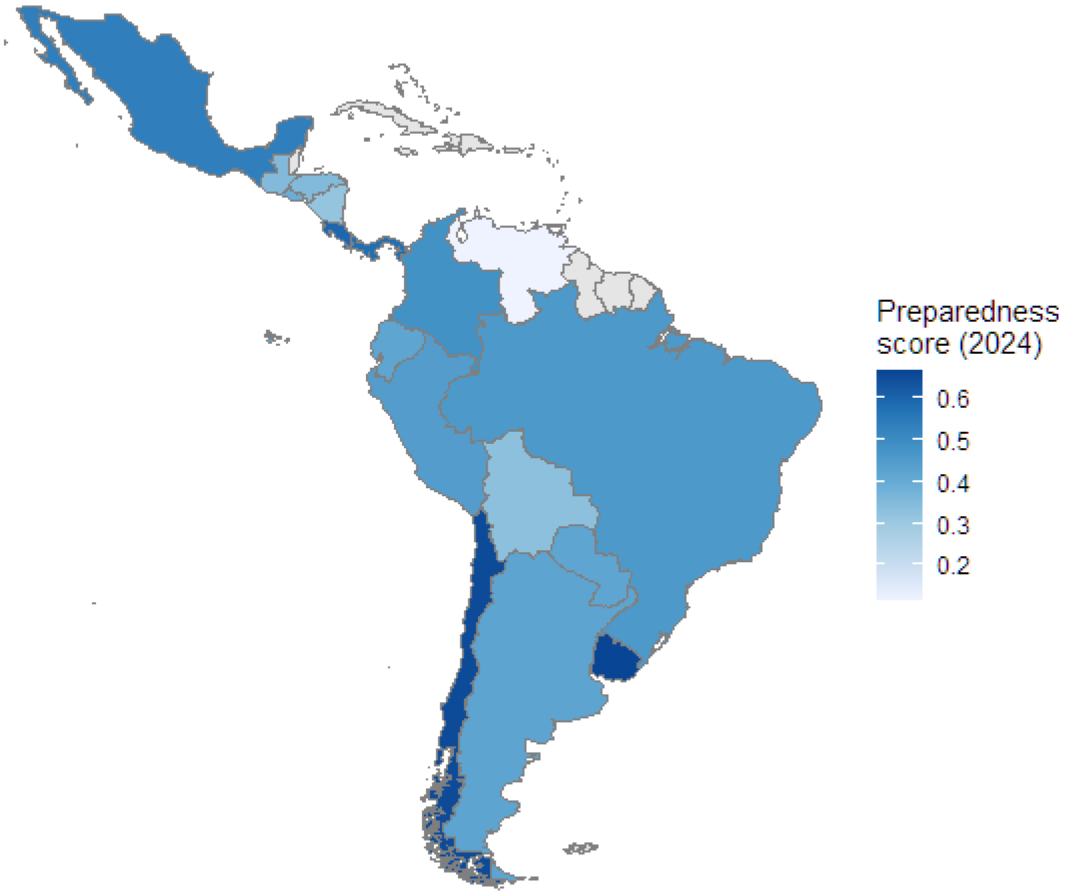

Figure A4.3. Climate-transition preparedness score (0 – 1) in Latin America (2024)

4.3: Financial transitions for a healthy future

4.3.1: Net value of fossil fuel subsidies and carbon prices

Regional author(s)

Christian García-Witulski, Chrissie Pantoja

Methods

This indicator follows the same methodology as the 2025 global Lancet Countdown report.

Additional analysis

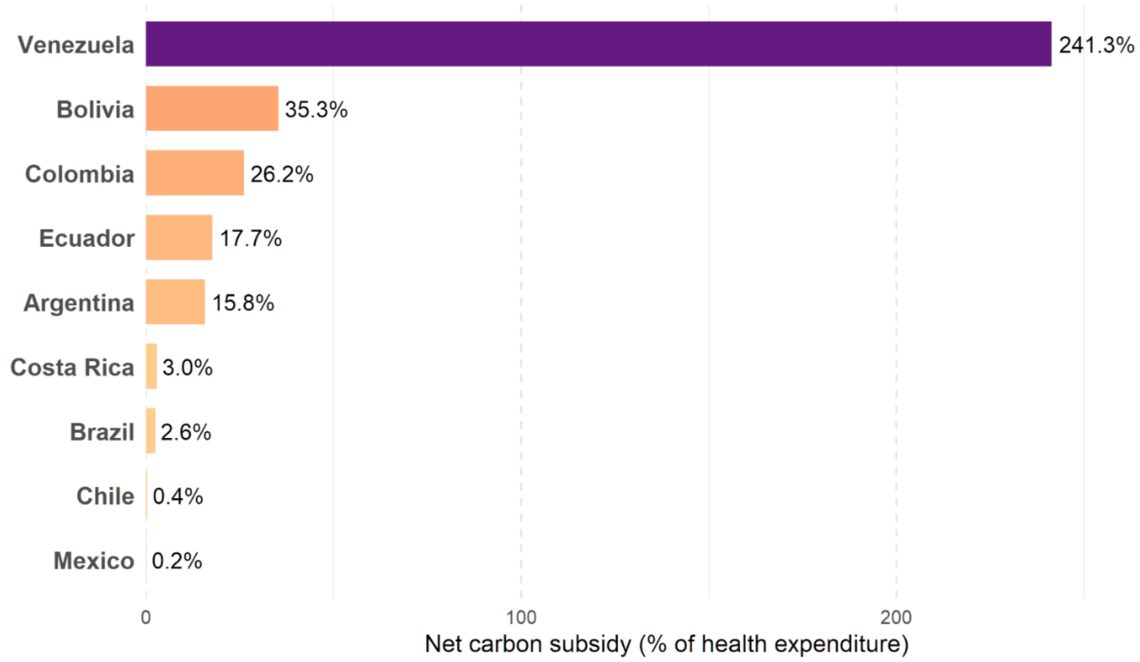

Figure A4.4. Net carbon revenue as % of current health expenditure in 9 Latin American countries (2023)

4.3.2: Health adaptation finance flows and disclosed needs

Regional author(s)

Yasna Palmeiro Silva, Christian García-Witulski

Methods

This indicator follows the same methodology as the 2025 global Lancet Countdown report.

Additional analysis

There are no additional analyses for this indicator.

Section 5: public and political engagement

5.1: media engagement with health and climate change

Regional author(s)
Carolina Gil Posse

Methods
This indicator follows the same methodology as the 2025 global *Lancet* Countdown report. Searches were conducted in Spanish (for Argentina, Bolivia, Chile, Colombia, Costa Rica, Ecuador, Mexico, Peru, and Uruguay) and Portuguese (for Brazil) in three databases (Nexis Uni, Proquest, and Factiva) accessed via the University of Colorado and University of York libraries.

This indicator monitors trends from 2007 to 2024 in 10 key newspapers from 10 countries in the region: *La Nación* in Argentina, *La Razón* in Bolivia, *O Globo* in Brazil, *El Mercurio* in Chile, *El Tiempo* in Colombia, *La Nación* in Costa Rica, *El Comercio* in Ecuador, *El Universal* in Mexico, *El Comercio* in Perú, and *El País* in Uruguay.

The searches were conducted with the following keywords in Spanish and Portuguese, respectively:

- Spanish: (cambio climático OR calentamiento global) AND (salud OR enfermedad OR epidemiología OR epidemiólog* OR desnutrición OR malnutrición OR morbosidad OR muert* OR diarrea* OR malaria OR paludismo OR chikungunya OR nilo del oeste OR nilo occidental OR virus del nilo OR dengue OR fiebre del heno OR rinitis alérgica OR zika)

- Portuguese: (mudanças climáticas OR aquecimento global) AND (saúde OR doença OR epidemiologi* OR desnutrição OR morbilidade OR fatalidade* OR diarr* OR malária OR chikungunya OR nilo do oeste OR vírus do nilo OR dengue OR febre dos fenos OR rinite alérgica OR zika)

Caveats
Since the number of newspapers included in the analysis is limited, the results are not representative of the media coverage in Latin America in general.

Future iterations

This indicator will continue to evolve, with future iterations of the report aiming to add more relevant and diverse media outlets from Latin American countries. In addition to the frequency analysis that this version of the indicator currently covers, a content analysis could provide information to differentiate between news articles that include health-related keywords but are not necessarily focused on the connection between health and climate change, and the number of articles that are entirely focused on this connection. This analysis could be useful to understand why and when a health and climate change event or topic is newsworthy, to explore strategies that would help to expand news coverage of this intersection.

Additional analysis

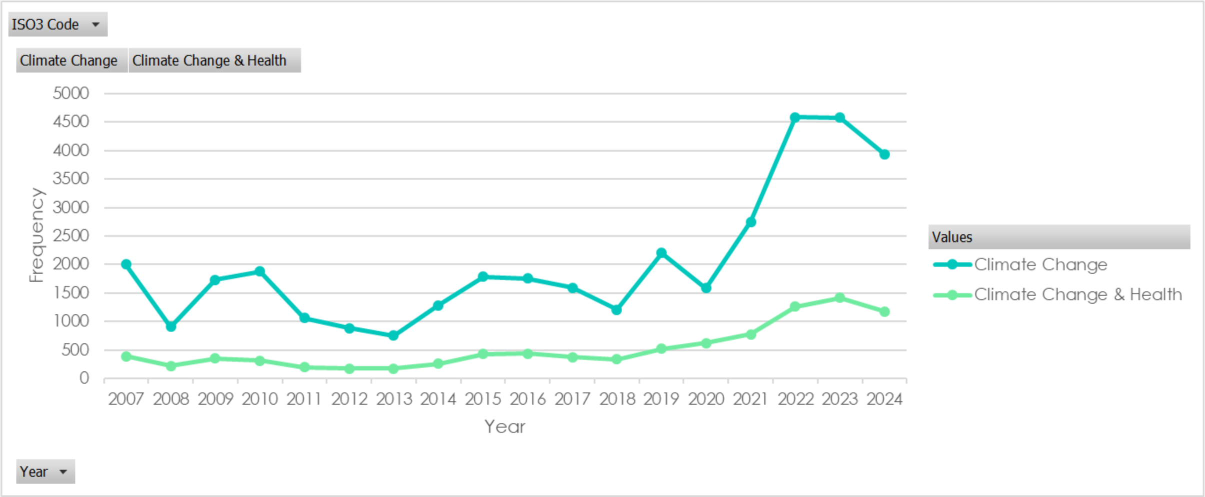

Figure A5.1. Newspaper engagement measured by total number of articles with climate change-related keywords, and health and climate-related terms, in ten key newspapers from ten countries in Latin America (2007–2024).

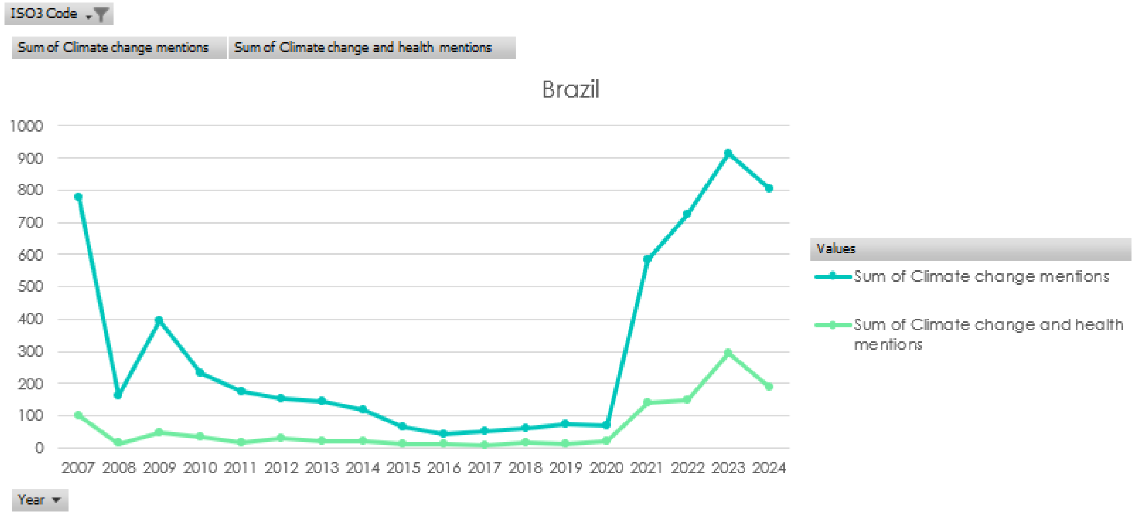

Figure A5.2. Newspaper engagement measured by total number of articles with climate change-related keywords, and health and climate-related terms, in Brazil (2007–2024).

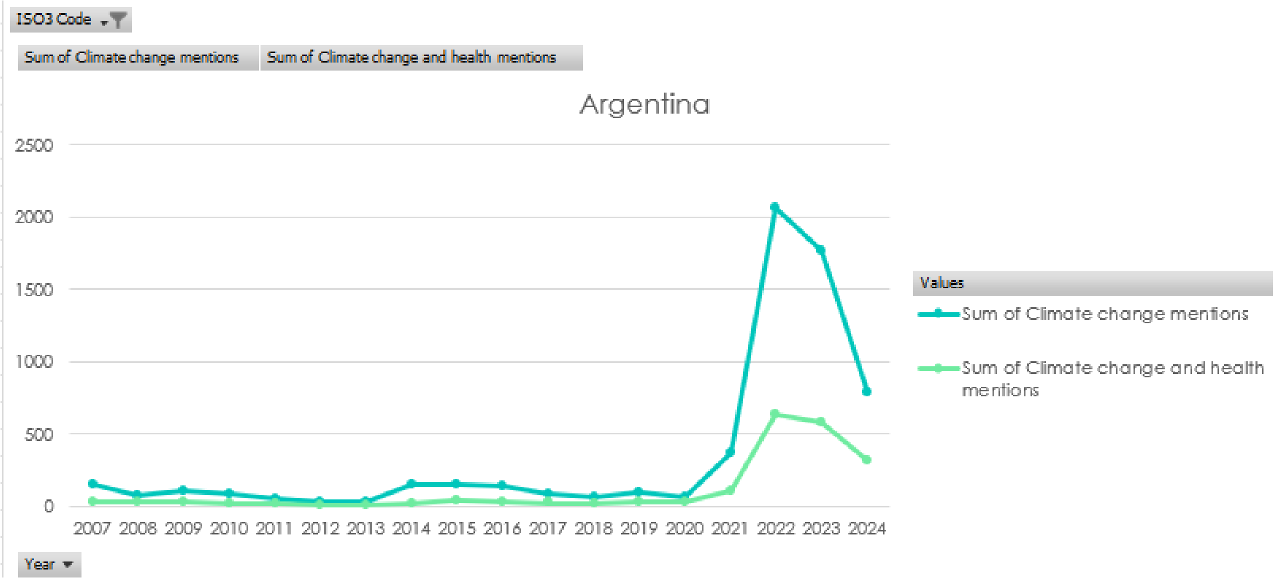

Figure A5.3. Newspaper engagement measured by total number of articles with climate change-related keywords, and health and climate-related terms, in Argentina (2007–2024).

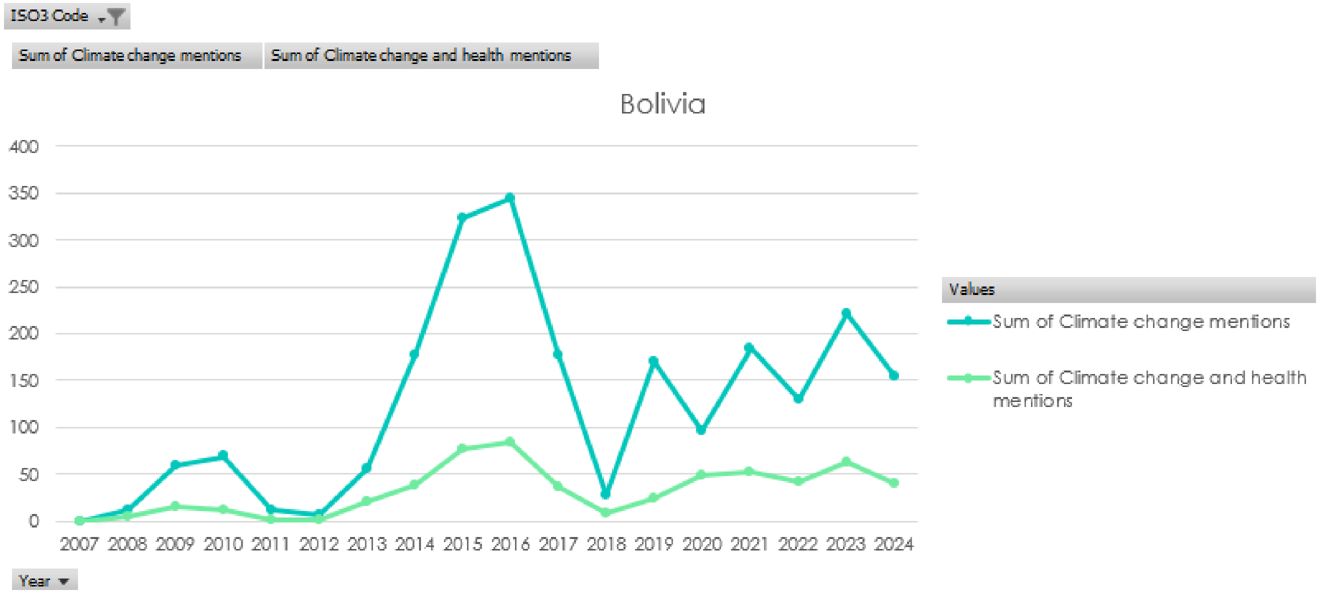

Figure A5.4. Newspaper engagement measured by total number of articles with climate change-related keywords, and health and climate-related terms, in Bolivia (2007–2024).

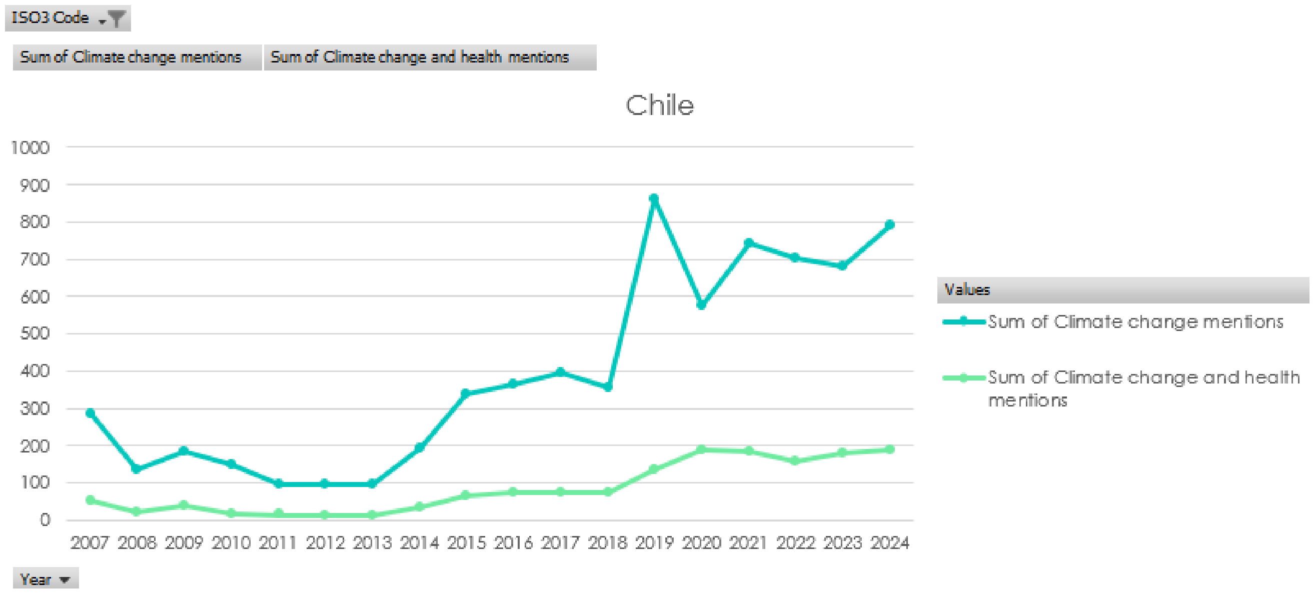

Figure A5.5. Newspaper engagement measured by total number of articles with climate change-related keywords, and health and climate-related terms, in Chile (2007–2024).

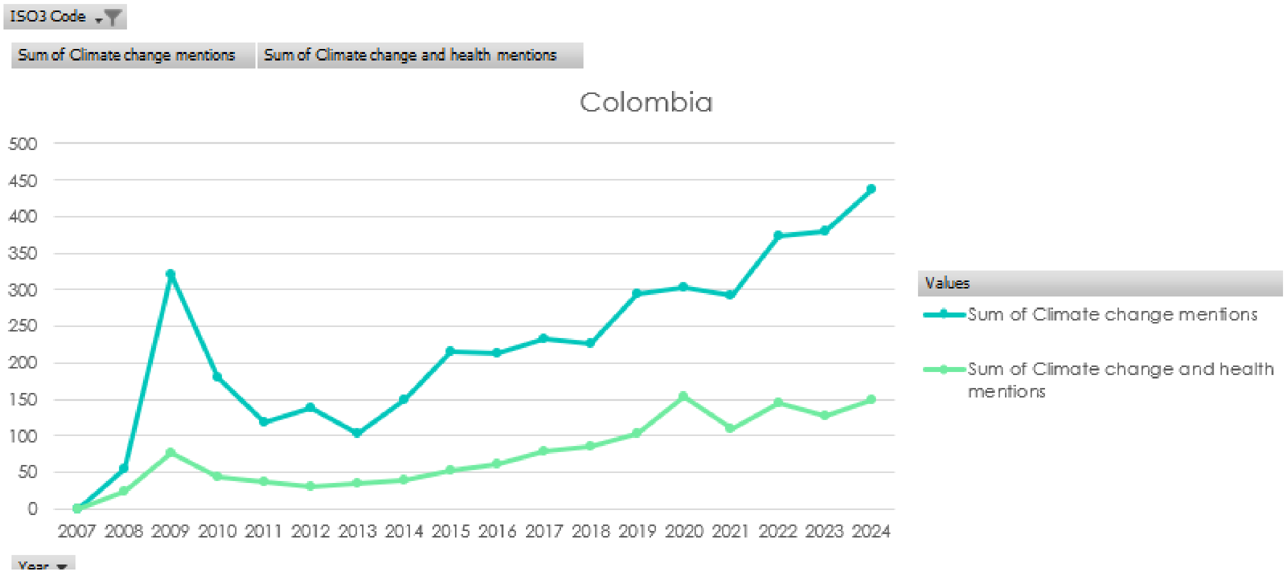

Figure A5.6. Newspaper engagement measured by total number of articles with climate change-related keywords, and health and climate-related terms, in Colombia (2007–2024).

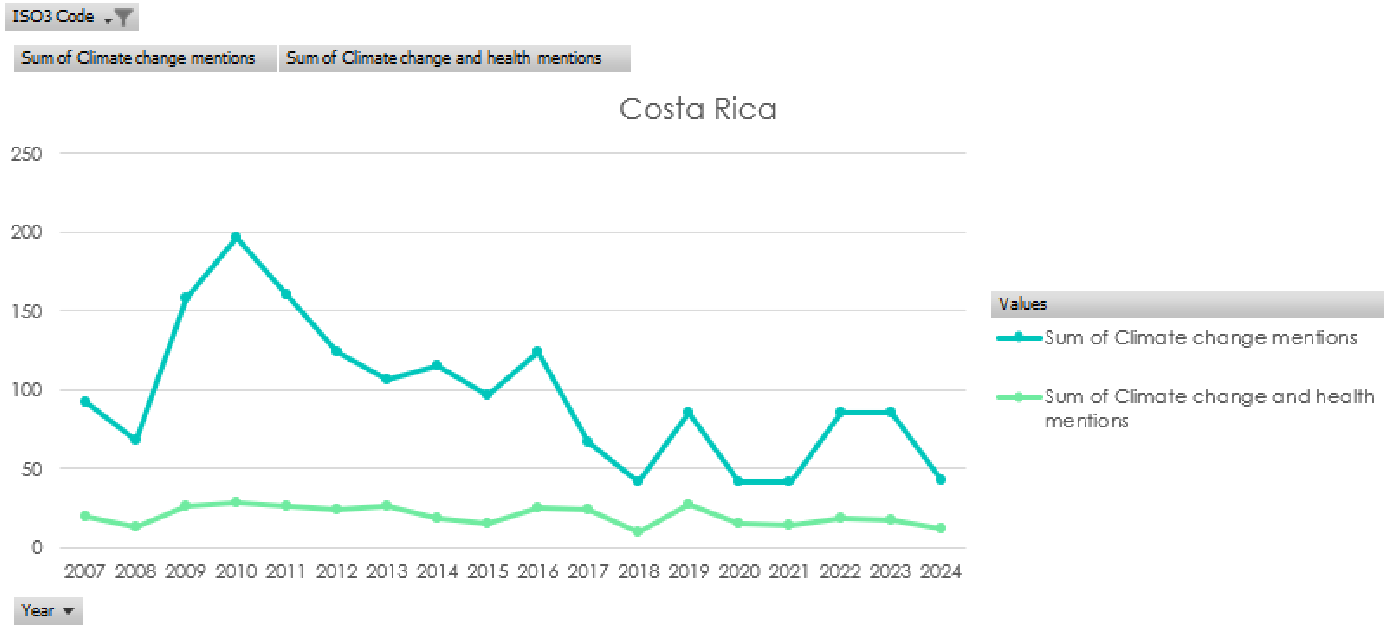

Figure A5.7. Newspaper engagement measured by total number of articles with climate change-related keywords, and health and climate-related terms, in Costa Rica (2007–2024).

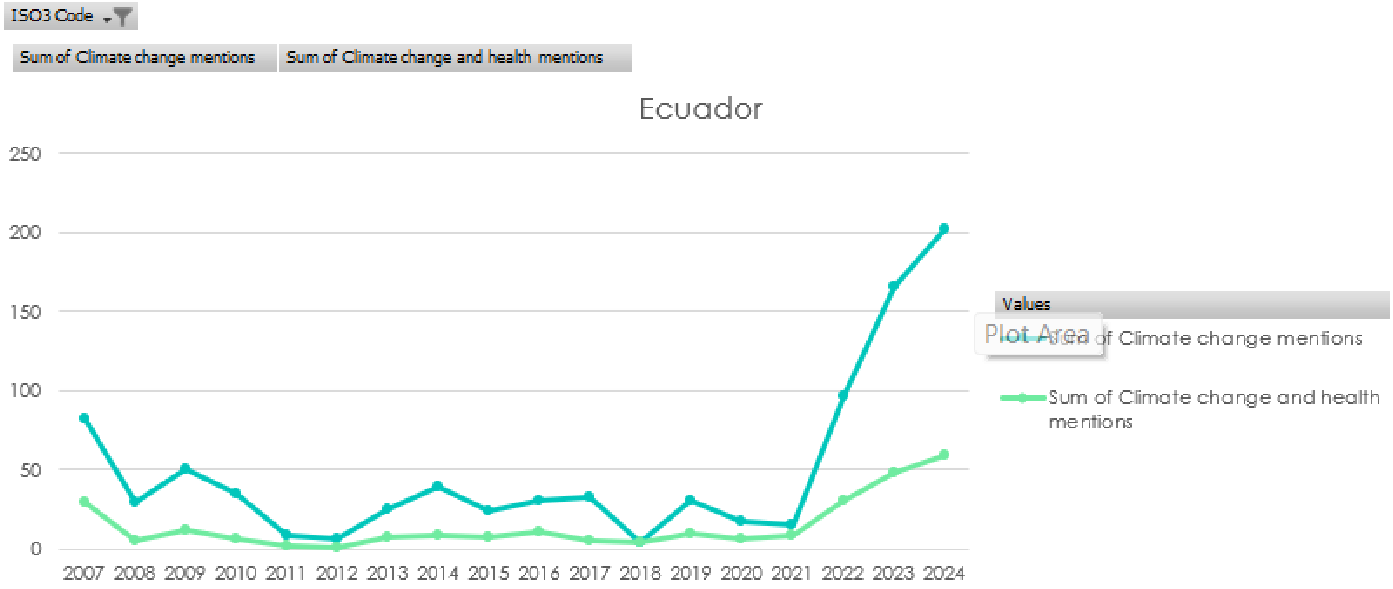

Figure A5.8. Newspaper engagement measured by total number of articles with climate change-related keywords, and health and climate-related terms, in Ecuador (2007–2024).

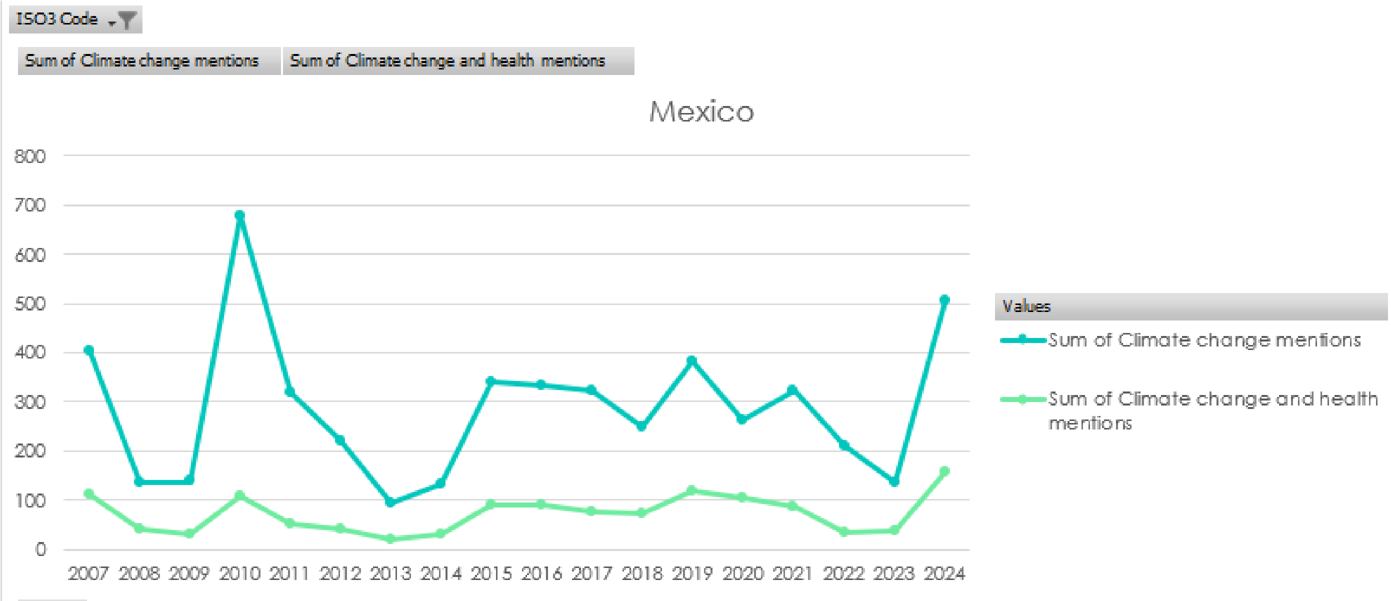

Figure A5.9. Newspaper engagement measured by total number of articles with climate change-related keywords, and health and climate-related terms, in Mexico (2007–2024).

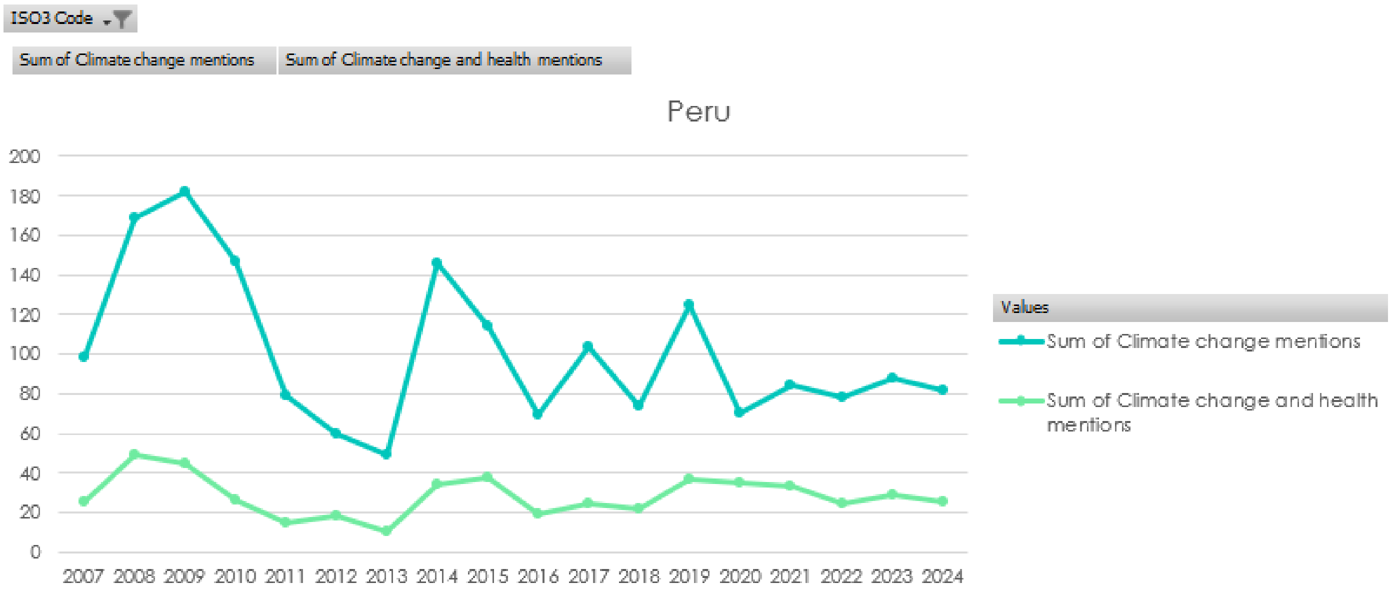

Figure A5.10. Newspaper engagement measured by total number of articles with climate change-related keywords, and health and climate-related terms, in Peru (2007–2024).

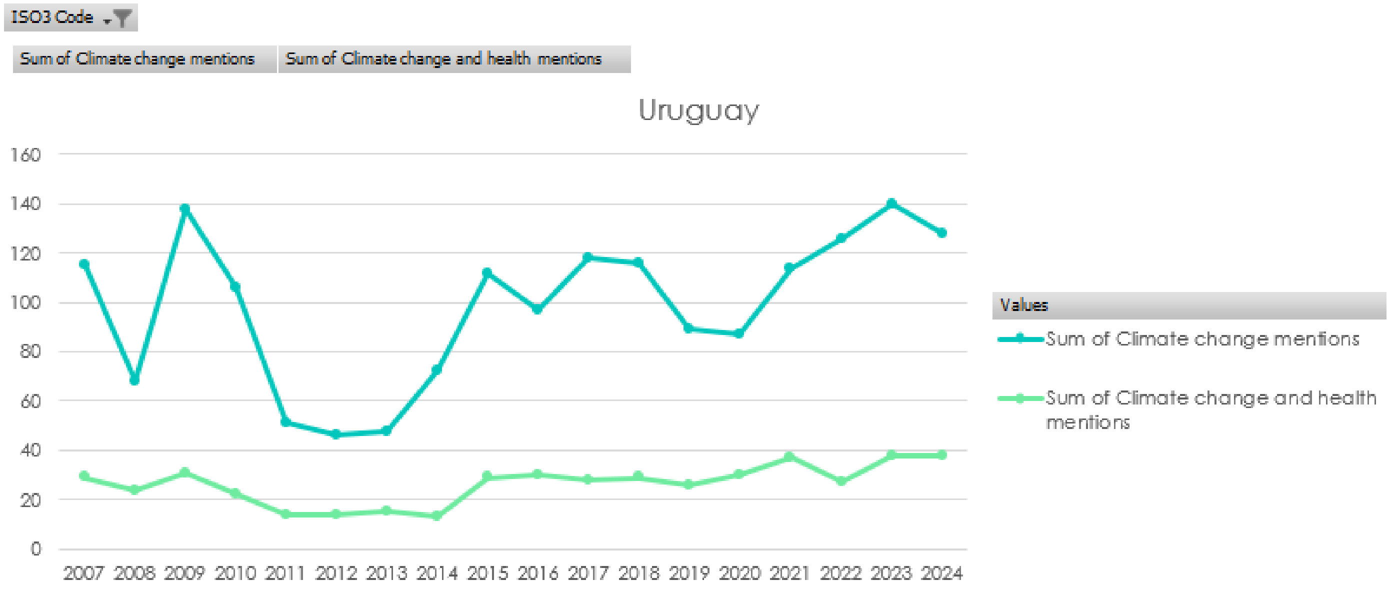

Figure A5.11. Newspaper engagement measured by total number of articles with climate change-related keywords, and health and climate-related terms, in Uruguay (2007–2024).

5.2: social media engagement with health and climate change

Regional author(s)
María Fernanda Salas

Methods

This indicator represents how much the public engages with the topic of health and climate change in social media, specifically. Facebook, the most used social media platform in the region.[20,21] This indicator tracks the proportion of climate related posts that also mention health related terms.

This indicator fills a gap in the scope of the first iteration of the regional report. Previously, the research team was not able to provide a regional outlook related to individual engagement. Tracking the use of social media in relation to health and climate change would provide insights into how much social media users in each country are focusing on these issues, how the conversation changes through time, and even what people are specifically saying about these topics.

Facebook has a 77.8% penetration rate across Latin America and the Caribbean with an estimated 367.4 million users in 2022.[20,21] This makes Facebook the most popular and visited social network in the region of interest. Certain groups might be underrepresented because of access to cellular technology or preference for other social media sites (e.g. younger people use Tik Tok and Instagram more than older adults).

In particular, this indicator analyses the number of posts per country across time (i.e. data is tracked daily by would be aggregated by month) related to the topic and the amount of engagement each one received. In this case, engagement is measured by the number of views to each post, number of comments, number and type of reactions (i.e. like, love, wow, *haha*, angry, sad, care), and number of shares per post. With the data from CrowdTangle a longitudinal analysis can be carried out to see how much Latin American Facebook users interact with the topic of health and climate change, and how these trends change overtime.

Databases

Crowtangle is the primary and official database to obtain data from social media belonging to Meta. This platform allows us to do keyword searches with specific parameters such as time frames and countries of publication. The results yield all the public posts relevant to the search. A CVS file can be downloaded with all the information related to the post such as engagement metrics, country of origin, date of publication, link, etc.

The present limitations are related to the demographics that interact with the sample. For example, it is possible to know specifically which account posted something and how much engagement it amassed, but Crowdtangle does not provide information about the age, gender, or location of the accounts that interacted with said post.

Additional analysis

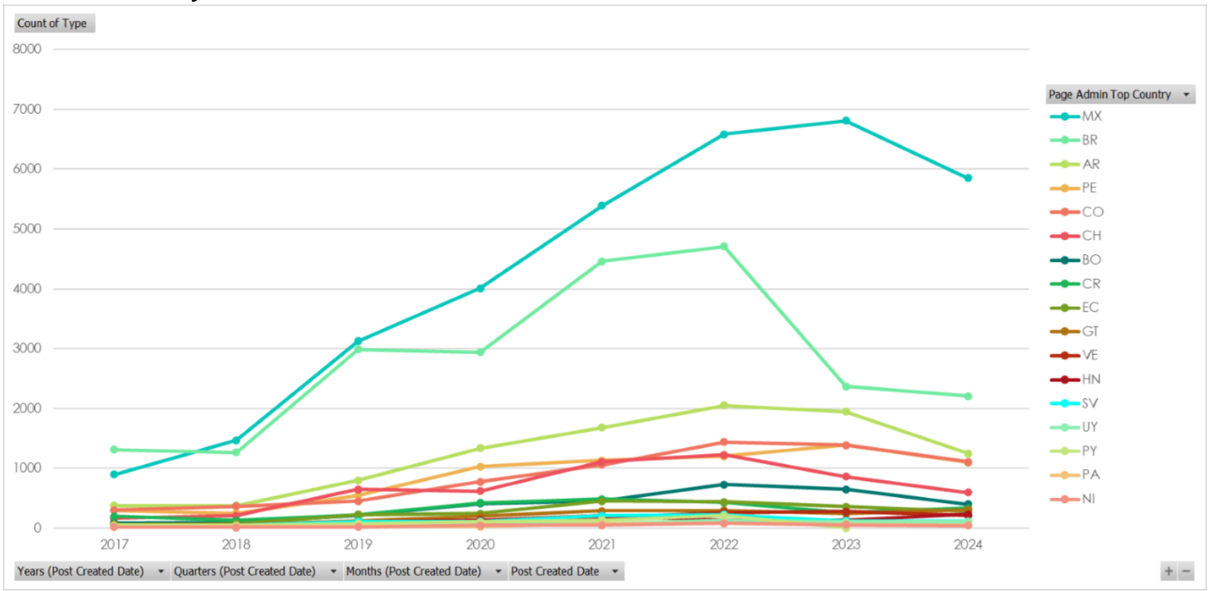

Figure A5.12. Number of posts related to climate change and health per country from 2017 to 2024.

5.3: Scientific articles on health and climate change

Indicator Authors

Dr Bruno Takahashi, Dr Tim Repke, María Fernanda Salas

Methods

The data presented in this annex are directly derived from Indicator 5.3 of the Global Report of The Lancet Countdown, which tracks the scientific literature at the intersection of human health and climate change. This indicator uses bibliographic records from the open scientific database OpenAlex. For further information about the methods and the full keyword list, refer to the annex of the global report.

For the regional report, we filtered for articles mentioning: Argentina, the Plurinational State of Bolivia, Brazil, Chile, Colombia, Costa Rica, Ecuador, Guatemala, Honduras, Mexico, Nicaragua, Panama, Peru, Paraguay, El Salvador, Uruguay, or the Bolivarian Republic of Venezuela.

There were 3,666 mentions of Latin American countries across 3,133 papers, representing 5% of the 56,998 global publications.

Scientific production in the region has increased significantly over the last 30 years:

- 8,233% increase from 1994 to 2024
- 681% increase in 2024 compared with 2004
- 82% increase in 2024 compared with 2014

However, the proportion of regional articles compared with the global trend has remained low. The highest share was in 2001, with 8% of global articles mentioning a Latin American country. In both 1990 (the first year) and 2024 (the most recent year), the share was 4%.

Using the same OpenAlex database as the global report, we also conducted a search using equity-related keywords. The keywords were categorized and combined using the Boolean operator OR, as follows:

General:

Extractivism OR Local communities OR Intercultural health OR Ethnic vulnerability OR Cultural identity OR Discrimination OR Discrimination based on gender or ethnicity OR Social justice OR Environmental injustice OR Environmental justice OR Just transition OR Colonialism OR Coloniality OR Rural communities OR Marginalized areas OR Sacrifice zones OR Vulnerable populations OR Peri-urban areas OR Coastal communities OR Isolated regions OR Poor urban populations OR Land delimitation OR Land rights OR Social exclusion OR Human rights OR Unequal access

Race:

Race OR Racism OR Racialized OR Ethnicity OR Ethnic OR Afro-descendants OR Quilombola OR Afro-Latinos OR Afro-Caribbeans

Indigenous:

Indigenous OR Indigenous peoples OR Indigenous territoriality OR Indigenous rights OR Ancestral knowledge OR Traditional knowledge OR Child health inequities OR Indigenous environmental justice OR Indigenous communities OR Native communities OR Original peoples OR Ethnic groups OR Natives

Gender:

Gender OR Women OR Machismo OR Misogyny OR Sexism OR Rural women OR Gender justice OR Gender rights OR Gender gap OR Maternal health OR Pregnancy OR Women's empowerment OR Female vulnerability OR Gender-based violence OR Violence Against Women OR Indigenous women OR Female leadership OR Gender perspective

Economic level:

Poverty OR Income inequality OR Unemployment OR Low income OR Homelessness OR Economic hardship OR Debt OR Affordable housing OR Extreme poverty OR Energy poverty OR Gentrification OR Economic inequality OR Inequity OR Resource scarcity OR Economic exclusion OR Limited access to basic services OR Socioeconomic vulnerability OR Economic marginalization OR Food security OR Social stratification OR Unequal development OR Universal health coverage OR Universal health access OR Environmental racism OR Structural inequality OR Health inequity OR Territorial inequality

Pregnancy:

Pregnancy OR Pregnant OR Prenatal OR Cesarean OR Miscarriage OR Abortion OR Spontaneous abortion OR Fetus OR Fetal OR Embryo OR Birth OR Childbirth OR Maternity

Although none of the categories were highly represented in the dataset, gender was the most prominent one, with 13.8% of Latin American abstracts including at least one gender-related keyword. The least represented category was indigenous, appearing in only 3.8% of abstracts.

5.4: Political engagement with health and climate change

5.4.1: government commitment with health and climate change in UNGA & NDCs

Regional author(s)

Milena Seergeva

Methods

This indicator follows the same methodology as the *2025 Global Lancet Countdown* report. Nationally Determined Contributions (NDCs) are submitted every five years to the UNFCCC secretariat, with the second round due in 2020. Accordingly, we considered NDCs presented around 2015 as part of the first round, while updated or new NDCs presented between 2019 and 2020 were treated as part of the second round.

Mentions of wellbeing and disease are the most frequent after general references to health, suggesting a growing orientation toward preventive approaches and the promotion of quality of life within climate mitigation and adaptation strategies. Only three countries—Chile, Panama, and Uruguay—mention mental health across all NDC iterations, and none address injury. Despite its increasing epidemiological and climate relevance in the region, extreme heat appears only in the NDCs of Uruguay and El Salvador.

In the third round of NDCs, Uruguay stands out for its sustained progress, integrating health across multiple climate-related dimensions, including disease, heat, mental health, and infrastructure. In contrast, Brazil has not developed a robust health narrative in its NDCs, limiting itself to isolated mentions without thematic expansion.

5.4.2: funding for science on health and climate in Latin America

National funding for climate change and health research

Indicator Authors

Luciana Blanco

Methods

This indicator evaluates national investment in research that addresses the intersection between climate change and health in Latin America. It tracks the number of projects and total funding allocated through national research funding agencies between 2019 and 2024.

The first step involved identifying national research councils or public agencies responsible for funding science and technology across 17 Latin American countries. Publicly available information on competitive funding calls and awarded projects was reviewed. Only countries with active national funding institutions and transparent reporting systems were included in the analysis.

From the accessible data, active calls for proposals between 2019 and 2024 were examined. The lists of awarded projects were extracted from each call, including title, year, awarded amount (converted to USD), and institution.

To identify projects situated at the intersection of climate change and health, a two-stage keyword-based filtering process was applied using the R programming language. In the first stage, projects were filtered based on a curated list of climate-related terms (e.g., “cambio climático”, “crisis climática”, “calentamiento global”),

as detailed in the keyword appendix. Case-insensitive regular expressions were used to detect both partial and exact matches. The resulting subset was then refined through a second filter using health-related keywords (e.g., “salud”, “enfermedades infecciosas”, “salud mental”, “nutricion”). The filtering was performed using the grepl() function in R, and searches were conducted in both Spanish and English to maximize capture. All projects identified through this dual-filtering process were manually reviewed to ensure thematic relevance and to eliminate false positives, such as projects in which the keywords appeared in unrelated contexts.

The final dataset includes the following metrics:

- Total number of research projects funded per year
- Number of projects on climate change
- Number of projects specifically on climate change and health

Data

- Public databases and official websites of national science and technology councils in Latin America
- Project titles, funding amounts, and program descriptions from national funding calls (2019–2024)
- Keyword lists for climate and health-related terminology (see Appendix for complete list)

Caveats

There are several limitations to this approach. First, transparency and data availability vary by country, and not all national funding institutions publish detailed information on the projects they have awarded. In some cases, such as Brazil, project records only include the name of the principal investigator, without providing the project title, which prevents the application of keyword-based filtering. Second, only projects that explicitly mentioned the selected keywords in their titles were included; relevant projects using alternate terminology or implicit references may have been excluded. Third, countries without centralized research funding mechanisms, such as Costa Rica, were excluded from the analysis.

Future form of the indicator

Future iterations of this indicator aim to expand coverage as more countries improve transparency in research funding. For countries that did not have publicly accessible research funding databases, as well as for those that did but did not include project titles, official letters will be sent to their national research institutions requesting access to this information. This effort aims to improve data completeness and ensure broader coverage of the indicator.

In addition to identifying projects that explicitly address the intersection between climate change and health, the indicator will also seek to capture those focused on adaptation and mitigation strategies that may generate co-benefits for health, even if not stated directly in their titles. This broader approach aims to more comprehensively reflect research efforts that, directly or indirectly, contribute to protecting population health in the context of climate change.

Keyword appendix

Climate-related keywords

Searches were conducted using the following climate-related terms (in Spanish and English where indicated), including compound phrases and individual keywords:

Compound phrases (Spanish and English): “cambio climático”, “crisis climática”, “emergencia climática”, “calentamiento global”,“efecto invernadero”, “variabilidad climática”, “evento climático extremo”, “fenómeno meteorológico severo”, “olas de calor”, “altas temperaturas”, “sequía prolongada”, “tormentas tropicales”, “deslizamientos de tierra”, “nevadas extremas”, “tormentas de nieve”, “erosión del suelo”, “global warming”, “climate change”, “climate emergency”,“climate crisis”, “greenhouse effect”, “extreme weather event”

Individual keywords: “cambio”, “climático”, “climática”, “climáticos”, “climáticas”, “clima”, “crisis”, “emergencia”, “calentamiento”, “global”, “efecto”, “invernadero”, “variabilidad”, “evento”, “extremo”, “fenómeno”, “meteorológico”, “severo”,“olas”, “calor”, “temperaturas”, “altas”, “sequía”, “desertificación”, “tormentas”, “tropicales”, “deslizamientos”, “tierra”, “nevadas”, “nieve”, “erosión”, “suelo”, “climate”, “change”, “warming”, “crisis”, “emergency”, “variability”, “greenhouse”, “effect”, “extreme”, “weather”, “event”, “phenomenon”, “heatwave”, “heat”, “drought”, “desertification”, “flood”, “storm”, “hurricane”, “cyclone”, “monsoon”, “landslide”, “erosion”, “snow”, “frost”, “cold”

Health-related keywords

The following terms were used to identify projects addressing health-related issues in the context of climate change. Searches were conducted in both Spanish and English.

Compound phrases (Spanish and English): “salud mental”, “enfermedades infecciosas”, “brotes epidémicos”, “enfermedades transmitidas por el agua”, “enfermedades transmitidas por los alimentos”, “trastornos del sueño”, “estrés postraumático”, “trastornos de salud mental”, “vector borne diseases”, “cambio en la producción de alimentos”, “seguridad alimentaria”, “inseguridad alimentaria”, “ecoansiedad”, “ansiedad climática”, “golpe de calor”, “mental health”, “infectious diseases”, “foodborne illness”, “waterborne illness”, “vector-borne diseases”, “climate anxiety”, “food security”, “malnutrition”, “post traumatic stress”

Individual keywords: “salud”, “mental”, “enfermedad”, “enfermedades”, “transmitidas”, “agua”, “alimentos”, “vectores”, “vector”, “epidemias”, “brotes”,“estrés”, “sueño”, “postraumático”, “ansiedad”, “depresión”, “duelo”, “suicidio”, “heridas”, “accidentes”, “quemaduras”, “fracturas”,“lesiones”, “desnutrición”, “malnutrición”, “hambre”, “hambruna”, “desplazamientos”, “refugiados”, “climáticos”, “nutrición”, “alimentaria”, “alimentación”, “micronutrientes”, “deficiencia”, “cardiovascular”, “hipertensión”, “infarto”, “asma”, “neumonía”, “bronquitis”, “EPOC”, “zika”, “dengue”, “malaria”, “leishmaniasis”, “salmonella”, “e coli”, “cólera”, “giardiasis”, “health”, “mental”, “disease”, “infection”, “epidemic”, “outbreak”, “water”, “food”, “vector”, “anxiety”, “stress”, “trauma”, “sleep”, “injury”, “wound”, “burn”, “fracture”, “malnutrition”, “undernutrition”, “hunger”, “famine”, “nutrition”, “security”, “deficiency”, “micronutrient”, “cardiovascular”, “hypertension”, “stroke”, “asthma”, “COPD”, “pneumonia”, “zika”, “dengue”, “malaria”, “cholera”, “salmonella”, “e.coli”, “giardiasis”, “rickettsia”, “encephalitis”

Additional analysis

Figure A5.13 shows that between 2020 and 2024, a total of 2,062 research projects were funded across the six countries analyzed. The overwhelming majority (92%) were not explicitly related to climate. Only 7.4% were climate-related without a health component, and a mere 0.6% integrated both climate and health.

The graph shows a steep rise in total funded projects in 2022, reaching 1,062 projects, with only 4 of those addressing both climate and health. This sharp increase may be partially explained by the delayed release of research funding following the disruptions caused by the COVID-19 pandemic. After initial interruptions in 2020 and 2021, many countries resumed or accelerated calls for proposals in 2022, potentially concentrating on a higher volume of funding that year.

These findings highlight a persistent gap in funding for projects at the intersection of climate and health. Despite the growing global recognition of the health impacts of climate change, investment in research that explicitly links the two remains extremely limited in the region.

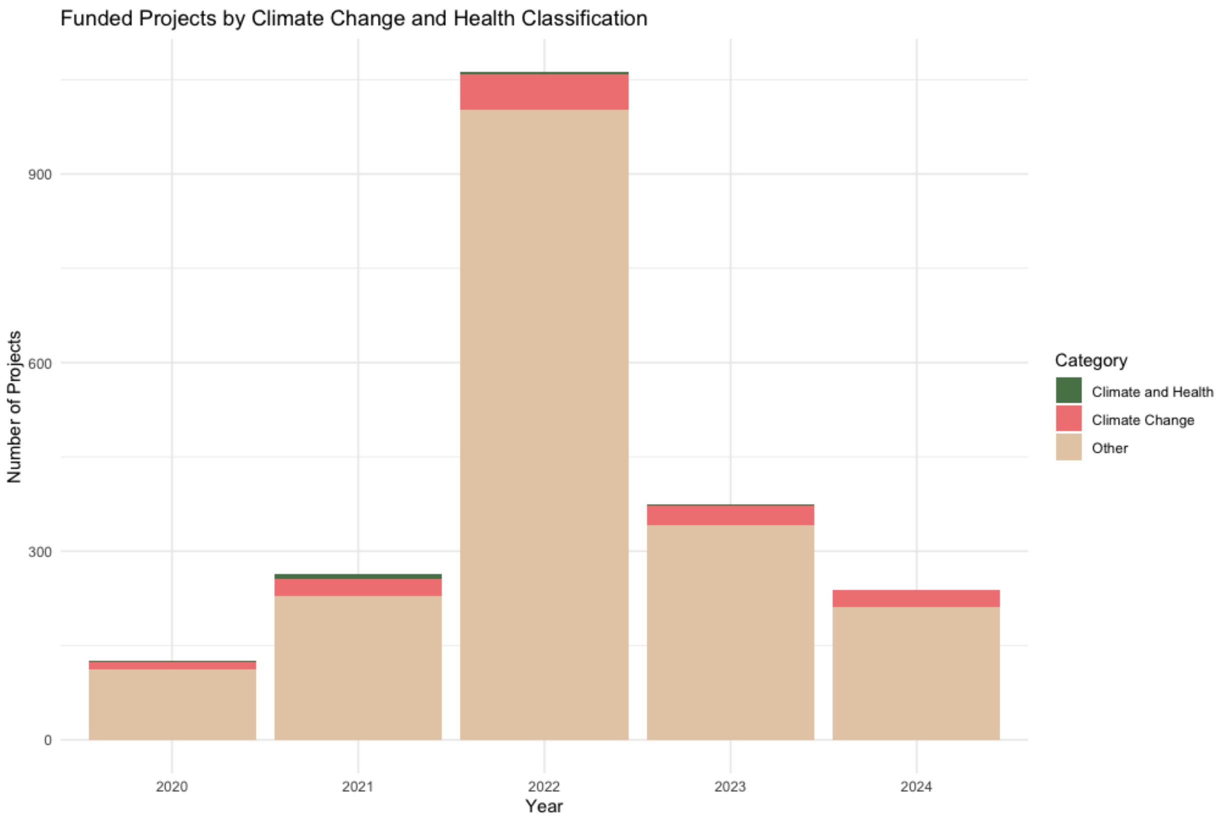

Figure A5.13. Number of funded research projects related to climate and climate + health in Latin America, by year (2020–2024).

Figure A5.14 shows the distribution of 2,063 funded research projects across the six Latin American countries analyzed. Overall, most funded studies were not related to climate change, and even fewer addressed the intersection of climate change and health. Mexico was the only country with a comparatively higher number of integrated projects, funding 8 out of 238 in this category. In contrast, Argentina accounted for the highest share of total funded projects, with 999 projects (48.4%), yet its investment in climate-related topics remained low, with only one project addressing both climate change and health. These differences reflect distinct national research priorities and underscore the persistent regional gap in funding for projects that address climate change, especially those that also consider health.

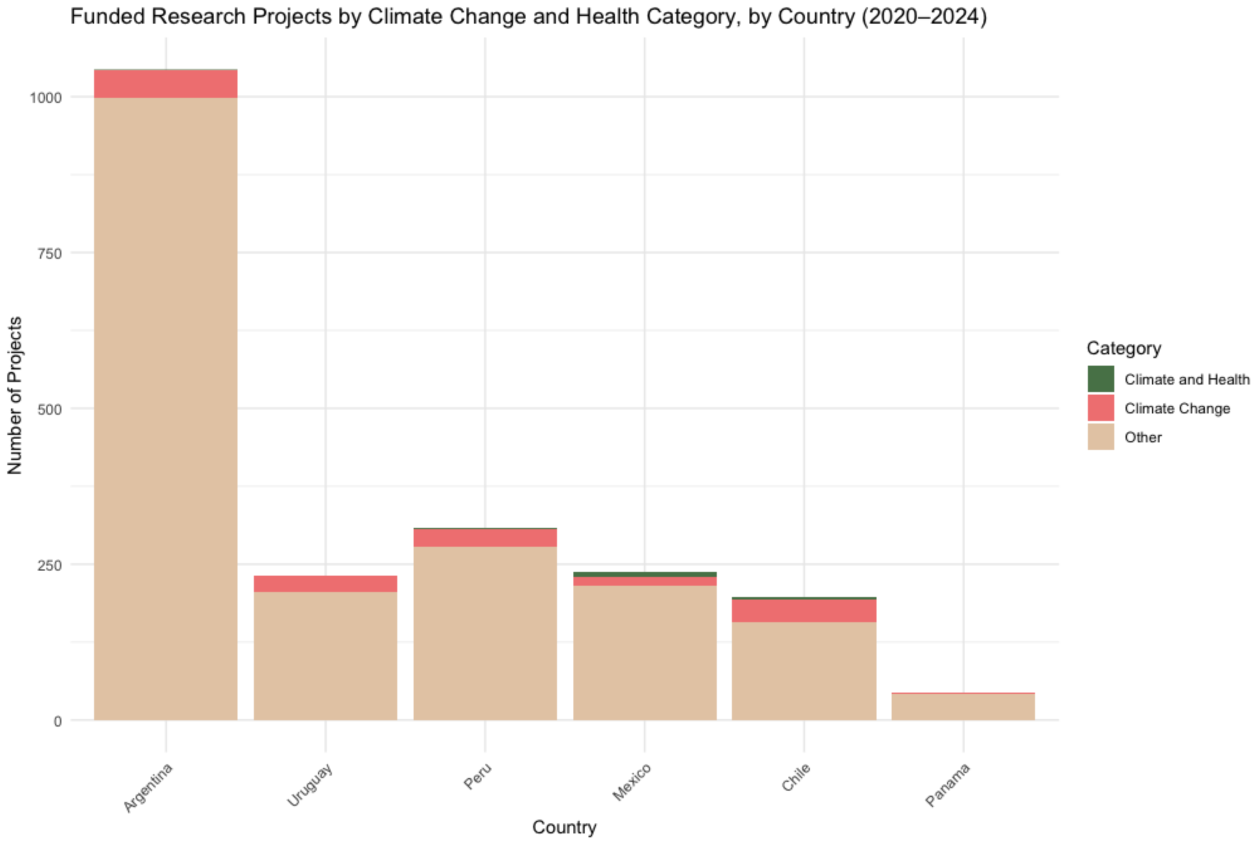

Figure A5.14. Number of funded research projects related to climate and climate + health across six Latin American countries (2020–2024).

5.5: corporate sector engagement with health and climate change

Regional author(s)

Bruno Takahashi, María Fernanda Salas

Methods

This indicator follows the same methodology as the 2025 global *Lancet* Countdown report.

Countries included are Argentina, Bolivia, Brazil, Chile, Colombia, Costa Rica, Ecuador, El Salvador, Guatemala, Honduras, Mexico, Nicaragua, Panama, Paraguay, Peru, Uruguay, and Venezuela. The indicator identified any of the climate change related terms that appeared in proximity — this was based on a search of the 25 words before and after — any public health terms in the GCCOP reports. This was based on a search of the 25 words before and after a reference to a public health related term.

Additional analysis

There are no additional analyses for this indicator.

References

1 CMES. Fire danger indices historical data from the Copernicus Emergency Management Service. 2019. DOI:10.24381/CDS.0E89C522.

2 Hänninen R, Sofiev M, Uppstu A, Kouznetsov R. Daily surface concentration of fire related PM2.5 for 2003-2024, modelled by SILAM CTM when using the MODIS satellite data for the fire radiative power. 2025; : 0 file, 0.0 B.

3 Gomes MS, Cavalcanti IF de A, Müller GV. 2019/2020 drought impacts on South America and atmospheric and oceanic influences. *Weather Clim Extrem* 2021; **34**: 100404.

4 Fernández FJ, Vásquez-Lavín F, Ponce RD, *et al*. The economics impacts of long-run droughts: Challenges, gaps, and way forward. *J Environ Manage* 2023; **344**: 118726.

5 Marengo JA, Cunha AP, Cuartas LA, *et al*. Extreme Drought in the Brazilian Pantanal in 2019–2020: Characterization, Causes, and Impacts. *Front Water* 2021; **3**. DOI:10.3389/frwa.2021.639204.

6 Associates M. Amazon navigation threatened by record low water levels. Menas Assoc. https://menas.co.uk/blog/amazon-navigation-threatened-by-record-low-water-levels (accessed May 31, 2025).

7 CGTN. Brazil's Amazon port hits lowest water level since 1902. https://news.cgtn.com/news/2024-10-05/Brazil-s-Amazon-port-hits-lowest-water-level-since-1902-1xrFRLdRFra/p.html (accessed May 31, 2025).

8 Carbon Disclosure Project. 2022 Cities Climate Risk and Vulnerability Assessments by Region and Existence of Adaptation Goal(s)/Plan | CDP Open Data Portal. 2022. https://data.cdp.net/Climate-Hazards/2022-Cities-Climate-Risk-and-Vulnerability-Assessm/x987-i6hs (accessed Sept 22, 2023).

9 World Health Organization. Electronic IHR States Parties Self-Assessment Annual Reporting Tool. 2023. https://extranet.who.int/e-spar/ (accessed Sept 25, 2023).

10 Miembros FEPAFEM. Google Docs. https://docs.google.com/spreadsheets/d/e/2PACX-1vRVicns6Ep-PhrcNUyFCG9G98HR9IYsyjR3ewpigPJTkWr7B_MxhRNNOC1dIJyAlTIqCEC0ssaAicXf/pubhtml?widget=true&headers=false&usp=embed_facebook (accessed May 15, 2025).

11 Obolski U, Perez PN, Villabona-Arenas CJ, Thézé J, Faria NR, Lourenço J. MVSE : An R-package that estimates a climate-driven mosquito-borne viral suitability index. *Methods Ecol Evol* 2019; **10**: 1357–70.

12 World Bank Open Data. Climate Change Knowledge Portal. https://climateknowledgeportal.worldbank.org/ (accessed April 24, 2025).

13 WHO/UNICEF Joint Monitoring Programme (JMP). WASH Data. https://washdata.org/data (accessed April 24, 2025).

14 Tremblay JC, Ainslie PN. Global and country-level estimates of human population at high altitude. *Proc Natl Acad Sci* 2021; **118**: e2102463118.

15 Global Burden of Disease Study 2019 (GBD 2019) Data Resources | GHDx. https://ghdx.healthdata.org/gbd-2019 (accessed April 24, 2025).

16 Dengue: analysis by country - PAHO/WHO | Pan American Health Organization. https://www.paho.org/en/arbo-portal/dengue-data-and-analysis/dengue-analysis-country (accessed May 31, 2025).

17 Proportion of population with primary reliance on clean fuels and technologies for cooking (%). https://www.who.int/data/gho/data/indicators/indicator-details/GHO/gho-phe-primary-reliance-on-clean-fuels-and-technologies-proportion (accessed May 31, 2025).

18 The Sustainability Consortium, World Resources Institute, University of Maryland. Tree Cover Loss by Driver. 2023. https://www.globalforestwatch.org/dashboards/global/ (accessed Sept 23, 2023).

19 Curtis PG, Slay CM, Harris NL, Tyukavina A, Hansen MC. Classifying drivers of global forest loss. *Science* 2018; **361**: 1108–11.

20 Internet World Stats. Facebook World Stats and Penetration in the World - Facebook Statistics. 2022. https://www.internetworldstats.com/facebook.htm (accessed Dec 26, 2023).

21 Ortiz-Ospina E, Roser M. The rise of social media. *Our World Data* 2023; published online Dec 11. https://ourworldindata.org/rise-of-social-media (accessed Dec 26, 2023).
