# Supplementary material for: The 2025 Lancet Countdown Latin America report: moving from promises to equitable climate action for a prosperous future
Source: Lancet Reg Health Am. 2025 Oct 29;52:101276. doi: 10.1016/j.lana.2025.101276 (PMC12801031; doi:10.1016/j.lana.2025.101276)
Supplement: Executive Summary in Spanish [file mmc2.pdf]

LANCET COUNTDOWN:
HEALTH AND
CLIMATE CHANGE
IN LATIN AMERICA

Informe 2025 de The *Lancet* Countdown para América Latina: De las promesas a la acción climática equitativa para un futuro próspero

Stella M. Hartinger, Yasna Palmeiro-Silva, Camila Llerena-Cayo, Rayana Santos Araujo Palharini, Christian García-Witulski, Maria Fernanda Salas, Nicolas Valdés-Ortega, Avriel Diaz, Luis E. Escobar, Carolina Gil Posse, Juliana Helo Sarmiento, Andres G. Lescano, Oscar Melo, Monica Pinilla-Roncancio, David Rojas-Rueda, Tatiana Souza de Camargo, Bruno Takahashi, Luciana Blanco-Villafuerte, Nicolas Borchers-Arriagada, Marcia Chame, Francisco Chesini, Carole Dalin, Francisco Estrada, Marcelo Firpo Porto, Renata Gracie, Nelson Gouveia, Magali Hurtado-Díaz, Harry Kennard, Eliane Lima e Silva, Aline Martins de Carvalho, Zaray Miranda-Chacon, Nahid Mohajeri, Romulo Paes-Sousa, Chrissie Pantoja, Tim Repke, Luiza Ribeiro Alves Cunha, Antonella Risso, Matilde Rusticucci, Alejandro Saez Reale, Raquel Santiago, Mauricio Santos-Vega, Enzo Sauma, Sol Saliva, Milena Sergeeva, Cecilia Sorensen, Juan D Umaña, Armando Valdes-Velasquez, Maria Walawender, Juliana W. Rulli Villardi, Daniel Buss, Marina Romanello

Resumen ejecutivo

A nivel mundial, el 2024 fue el año más cálido registrado, con una temperatura media cercana a la superficie de 1,55 °C por encima de los niveles preindustriales. Este récord forma parte de una tendencia continua de calentamiento, con temperaturas que se han mantenido por encima del umbral de 1,5 °C durante casi dos años. Este aumento, impulsado principalmente por las actividades económicas, está generando peligros concurrentes en toda América Latina —incluyendo olas de calor, incendios forestales e inundaciones— que devastan comunidades, afectan medios de vida y provocan múltiples consecuencias para la salud de las poblaciones de la región.

En medio de esta amenaza creciente, la salud humana se va convirtiendo en un eje central de la diplomacia climática internacional. Durante la COP28, en el Marco de los EAU, se incorporó una meta específica en salud y se operacionalizó el Objetivo Global de Adaptación. Si bien estos pasos son significativos, persisten obstáculos sustanciales. A pesar de que ha pasado una década desde el Acuerdo de París, el mundo continúa lejos de alcanzar sus metas climáticas. La continua dependencia de los combustibles fósiles y el incumplimiento de los compromisos de reducción de emisiones y financiamiento climático son algunos de los principales factores que socavan los esfuerzos por proteger la salud humana y avanzar hacia un futuro próspero.

El Informe 2025 de The *Lancet* Countdown América Latina (LCLA) —

una colaboración de 25 instituciones académicas regionales y agencias de la ONU— evalúa 41 indicadores en 17 países latinoamericanos, proporcionando evidencia clara sobre los crecientes impactos del cambio climático en la salud humana. En esta edición se introdujeron mejoras metodológicas, incluyendo la mejora de indicadores existentes y la incorporación de seis indicadores nuevos. Además, se consideró una perspectiva subnacional siempre que los datos lo permitieron, reconociendo que los impactos climáticos y la eficacia de las respuestas varían significativamente entre e intra-países. A continuación, se resumen los principales hallazgos, organizados en torno a tres mensajes clave que reflejan los temas centrales del informe.

Enfrentar el cambio climático es esencial para proteger la salud humana. La salud humana seguirá deteriorándose en el futuro debido a los efectos acumulativos del cambio climático.

El informe LCLA 2025 evidencia el aumento de los impactos sanitarios del cambio climático antropogénico en América Latina, mostrando tendencias alarmantes de intensificación de peligros climáticos que ponen en riesgo a las personas y a la sociedad. Las poblaciones están cada vez más expuestas al calor, a eventos climáticos extremos y a un mayor riesgo de transmisión de enfermedades infecciosas.

La temperatura ambiental media en América Latina ha seguido una tendencia de calentamiento persistente desde el año 2000. La exposición anual promedio aumentó de 23,3 °C en 2001–2010 a 23,8 °C en 2015–2024, alcanzando un récord de 24,3 °C en 2024. Estos incrementos no son homogéneos: se observaron mayores exposiciones térmicas en países como Bolivia (+2 °C), Venezuela (+1,7 °C), México (+1,6 °C), Paraguay (+1,5 °C), Ecuador (+1,4 °C), Guatemala (+1,3 °C), Brasil (+1,2 °C) y Colombia (+1 °C), así como extremos térmicos aún mayores dentro de estos países. Las consecuencias para la salud son profundas: los niños menores de un año estuvieron expuestos a 4,5 veces más de días de olas de calor, mientras que los adultos mayores de 65 años enfrentaron una exposición 10 veces mayor en comparación con el periodo base 1981–2000. En Venezuela y Colombia, estos aumentos fueron especialmente elevados, con exposiciones 51 y 59 veces mayores, respectivamente. En general, la mortalidad atribuida al calor aumentó en un 103 %, con aproximadamente 13 000 muertes anuales, lo que representó un costo monetario promedio anual de US$ 855 millones durante 2015–2024 (229 % más que en la década anterior). Las pérdidas laborales relacionadas con el calor en 2024 ascendieron a US$ 52 000 millones (12,6 % más que en 2023), afectando desproporcionadamente a los sectores agrícola y de la construcción.

El aumento en la frecuencia e intensidad de eventos extremos, como sequías e incendios forestales, ha impactado a la mayoría de los países de la región. La proporción de tierras latinoamericanas bajo condiciones de sequía meteorológica (un mes) aumentó en 275%, pasando de 15,8 % en 1981–1990 a 59,1% en 2015–2024, con Brasil, Bolivia y México entre los más afectados. Esta tendencia también se presenta en sequías prolongadas: la proporción de tierras con sequía agrícola (tres meses) aumentaron de 6,3% a 40,7%, y las que tienen sequías hidrológicas (seis meses) de 2,1 % a 20,8 % en Latinoamérica para el mismo período. Esto probablemente contribuyó al riesgo extremo de incendios forestales observado en 2024, con un aumento del 10 % en la región (en 9 de 17 países). Los mayores incrementos se registraron en Chile (30,5 días, +105 %), México (17,6 días, +28,5 %) y Bolivia (16,7 días, +82,6 %).

Estos eventos extremos generaron pérdidas económicas directas cercanas a US$ 19,2 mil millones en 2024 (0,3 % del PIB regional). Lamentablemente menos del 5 % de estas pérdidas están aseguradas. Brasil concentró dos tercios de las pérdidas totales, seguido de México y Chile. En relación con el PIB nacional, Chile y Brasil tuvieron las mayores pérdidas proporcionales (ambos cerca de 0,63 %), seguidos por México (0,14 %), Panamá (0,13 %), Ecuador (0,08 %) y Perú (0,07 %).

Cabe destacar que muchos de estos eventos climáticos extremos ocurren simultánea o consecutivamente (por ejemplo, sequías prolongadas, olas de calor intensas e incendios forestales). Enfrentar el cambio climático es, por tanto, esencial para proteger la salud humana para evitar los riesgos en cascada y choques económicos que ralentizan la recuperación y debilitan la resiliencia.

La adaptación ha dejado de ser opcional, es un requisito esencial y no negociable. Se debe priorizar una estrategia multinivel que reduzca los riesgos climáticos, incremente la resiliencia y aborde las desigualdades socioeconómicas existentes.

Las acciones a nivel gubernamental y de política nacional siguen siendo insuficientes para combatir el cambio climático, evidenciando una falla sistémica en priorizar la resiliencia sanitaria. El informe LCLA 2025 muestra que los esfuerzos de planificación son limitados: menos de la mitad de los países de la región (41,2 %) han informado públicamente haber completado una Evaluación de Vulnerabilidad y Adaptación desde 2020, y solo nueve países (53 %) han desarrollado un Plan Nacional de Adaptación en Salud. Además, la integración del componente de salud en las Contribuciones Determinadas a Nivel Nacional (NDC) sigue siendo insuficiente.

Esta falta de impulso político se refleja también en los foros internacionales y

en el financiamiento climático. Aunque las NDC incluyen cada vez más consideraciones de equidad, su visibilidad en la Asamblea General de las Naciones Unidas (AGNU) ha disminuido notablemente: las menciones a la salud en los discursos de países latinoamericanos cayeron de un máximo de 10 en 2010 a solo tres países (Bolivia, Brasil y Chile) en 2024, debilitando la presencia regional de los vínculos entre clima, salud y justicia ambiental en el escenario global.

Superar esta falta de priorización requiere inversión dirigida a la planificación e implementación de la adaptación en salud. Los donantes bilaterales comprometieron US$ 197 millones para proyectos de adaptación en salud en 2024, pero el 68 % se asignó únicamente a Brasil. De los US$ 3,4 mil millones aprobados por el Fondo Verde para el Clima desde 2017 para proyectos con componente sanitario, solo US$ 77,7 millones (2,3 %) se destinaron directamente a la adaptación en salud.

El fortalecimiento de los sistemas de salud exige datos robustos y colaboración interinstitucional. Aunque 10 de los 17 miembros latinoamericanos de la Organización Meteorológica Mundial informan proveer servicios climáticos para la salud, estos se enfocan principalmente en monitoreo y datos inmediatos, con poca atención a proyecciones climáticas de largo plazo, necesarias para una planificación estratégica. La preparación institucional sólida es un pilar fundamental para sistemas de salud resilientes, y funciona: los países con sistemas de alerta temprana climáticamente informados (HEWS) registraron una reducción del 92,5 % en mortalidad por inundaciones y tormentas. Sin embargo, la preparación ante emergencias autorreportada ha disminuido desde 2022, un problema crítico para naciones vulnerables al dengue como Bolivia, Brasil y Perú.

Al mismo tiempo, la limitada capacidad de recursos humanos y el escaso financiamiento para la generación de conocimiento también debilitan la respuesta regional. Solo el 17 % de los estudiantes de salud pública reciben formación sobre cambio climático, lo que restringe la preparación de la fuerza laboral. La generación de conocimiento también es limitada. Pese al aumento de publicaciones científicas sobre clima y salud desde 2015, América Latina representa solo el 5,5 % de la producción mundial. Además, gran parte de la investigación omite temas de equidad, reflejando las brechas también en la financiación de proyectos que abordan explícitamente la intersección entre clima y salud. Sin evidencia local relevante, resulta difícil posicionar las necesidades regionales en las agendas internacionales.

A nivel local y comunitario, la implementación de estrategias de adaptación sigue siendo limitada y poco estratégica. La planificación urbana deficiente es evidente: todas las ciudades latinoamericanas con más de

500 000 habitantes fueron clasificadas con niveles bajos o muy bajos de áreas verdes, perdiendo una oportunidad clave para fortalecer la resiliencia urbana mediante infraestructura natural. De las unidades administrativas locales encuestadas, solo 54 reconocieron problemas de salud vinculados a amenazas climáticas, centradas principalmente en inundaciones, tormentas y lluvias intensas, seguidas por sequías y temperaturas extremas.

Por el contrario, la integración de la agenda climática con los Objetivos de Desarrollo Sostenible (ODS) constituye una base esencial para la resiliencia. Las mejoras en los servicios básicos de agua y saneamiento, por ejemplo, impulsaron una reducción cercana al 60 % en el Índice de Riesgo de Mosquitos desde 2000.

A nivel individual, la protección depende con frecuencia del estatus económico. El creciente uso de aire acondicionado representa una clara disyuntiva entre mitigación y adaptación: su alto consumo energético aumenta las emisiones de GEI cuando no está vinculado a fuentes limpias. Su baja adopción (27 % de los hogares) refleja desigualdades significativas en quién puede protegerse del calor extremo. Esta brecha se replica en la contaminación del aire en interiores, mucho más alta en zonas rurales y hogares de bajos ingresos.

El compromiso mediático y social refleja la polarización política actual. Se observa una señal mixta, con riesgo de desinformación y una disminución general de la cobertura sobre el vínculo clima-salud. Aunque la cobertura sanitaria aumenta modestamente en medios y redes sociales (donde el interés creció sustancialmente desde 2017), la cobertura general sobre cambio climático disminuyó en 2024, probablemente debido al desplazamiento hacia temas más politizados.

La gobernanza climática y sanitaria efectiva debe definirse por avances tangibles. Los gobiernos deben tomar decisiones que generen acción, rendición de cuentas e impacto real frente al cambio climático y la salud.

En toda América Latina, el financiamiento, el apoyo a una transición energética justa y la acción climática colectiva siguen siendo críticamente bajos. Un factor central detrás de este estancamiento es la persistente dependencia de los combustibles fósiles. Los países latinoamericanos presentan un precio neto negativo del carbono, con subsidios fósiles equivalentes a US$ 38,6 mil millones, casi 50 veces superiores a los ingresos generados por precios al carbono.

Construir un futuro resiliente requiere transformar de manera fundamental los sistemas energéticos y reducir la dependencia de los combustibles fósiles, lo cual exige mayores compromisos de mitigación reflejados

explícitamente en las NDC. Esta transformación debe incluir el uso de energía en los hogares y la adopción de transporte sostenible y saludable, sectores clave por su vínculo directo con las emisiones de GEI y la salud. Además, la forma en que producimos y consumimos alimentos, y cómo gestionamos nuestros bosques, tiene un impacto crucial en nuestra capacidad de prosperar frente a esta crisis.

Si bien sustituir el carbón y otros combustibles fósiles por energías renovables es esencial para un futuro saludable y sostenible, el informe LCLA 2025 muestra avances dispares entre países y sectores. A nivel regional, la participación de fuentes bajas en carbono en la generación eléctrica cayó de 67,6 % a 58,9 %, mientras que las energías renovables (solar y eólica) crecieron de 2,7 % a 11,8 %, superando al carbón en 2014. Sin embargo, la generación eléctrica a base de carbón casi se duplicó (de 2,6 % a 5,2 %), con un repunte tras la pandemia. Los combustibles fósiles siguen dominando el transporte por carretera, representando el 96,7 % del sector.

El no abordar estas fuentes de emisión mantiene altos niveles de exposición a contaminación por PM2.5. La concentración promedio nacional de PM2.5 en interiores por combustibles sólidos contaminantes para cocinar y calefaccionar se estimó en 245 $\mu g/m^3$ en 2022, más del doble en hogares rurales (314 $\mu g/m^3$) que en urbanos (145 $\mu g/m^3$), evidenciando profundas desigualdades socioeconómicas. Esta disparidad se debe al acceso limitado a combustibles limpios y al uso persistente de biomasa (31 % en zonas rurales frente a 5 % en urbanas). Además, el 79 % de los hogares latinoamericanos utiliza gas licuado de petróleo (GLP) para cocinar, lo que representa una oportunidad perdida para transitar directamente a fuentes renovables y resalta los desafíos futuros para una región aun fuertemente dependiente de los combustibles fósiles.

Las emisiones fósiles de PM2.5 (carbón y gas) de fuentes ambientales causan 360 000 muertes prematuras en personas en edad laboral, imponiendo una pesada carga social y económica. Las muertes prematuras atribuibles a PM2.5 por biomasa se estimaron en 140 000 durante 2018–2022, un aumento de 17 000 respecto a 2007–2011. Los costos monetizados de los Años de Vida Perdidos atribuibles a PM2.5 en 11 países latinoamericanos alcanzaron US$ 160 mil millones, equivalentes al 2,8 % del PIB agregado y al ingreso anual promedio de unos 15,8 millones de personas en la región.

Otras fuentes regionales de emisiones —como la agricultura y la pérdida de cobertura forestal impulsada por la producción de productos básicos y la deforestación— ya han convertido las zonas orientales del Amazonas en una fuente neta de carbono. Las estrategias de mitigación, como la agricultura regenerativa, la ganadería sostenible y la

agroecología, ofrecen soluciones al evitar la expansión, restaurar suelos y conservar la biodiversidad esencial para mantener la salud humana. Asimismo, la transformación del sistema alimentario representa una estrategia de mitigación clave con co-beneficios inmediatos para la salud y el ambiente, garantizando acceso equitativo a alimentos sostenibles, promoviendo la biodiversidad y fortaleciendo la seguridad alimentaria.

América Latina no tiene el lujo de esperar una mayor voluntad política global; debe avanzar con acciones nacionales que protejan a las personas y a la naturaleza. Es hora de que los países cumplan sus NDC y Planes Nacionales de Adaptación mediante una gobernanza efectiva —definida por la acción, la rendición de cuentas y los impactos medibles en salud—, y no solo por promesas esperanzadoras. Esto es especialmente crucial ante las tensiones geopolíticas y el cambio en las prioridades de los donantes, que amenazan con no materializar los recursos prometidos al ritmo necesario que permita proteger la salud pública.

A medida que se acerca la COP30 en Belém do Pará, la región tiene una oportunidad única para liderar iniciativas de adaptación climática equitativas y centradas en la salud, e impulsar estrategias de mitigación rápidas, con co-beneficios sanitarios y justicia para todos.
