# Supplementary material for: The 2025 Lancet Countdown Latin America report: moving from promises to equitable climate action for a prosperous future
Source: Lancet Reg Health Am. 2025 Oct 29;52:101276. doi: 10.1016/j.lana.2025.101276 (PMC12801031; doi:10.1016/j.lana.2025.101276)
Supplement: Executive Summary in Portuguese [file mmc3.pdf]

Relatório 2025 do The *Lancet* Countdown para a América Latina: das promessas à ação climática equitativa para um futuro próspero

Stella M. Hartinger, Yasna Palmeiro-Silva, Camila Llerena-Cayo, Rayana Santos Araujo Palharini, Christian García-Witulski, Maria Fernanda Salas, Nicolas Valdés-Ortega, Avriel Diaz, Luis E. Escobar, Carolina Gil Posse, Juliana Helo Sarmiento, Andres G. Lescano, Oscar Melo, Monica Pinilla-Roncancio, David Rojas-Rueda, Tatiana Souza de Camargo, Bruno Takahashi, Luciana Blanco-Villafuerte, Nicolas Borchers-Arriagada, Marcia Chame, Francisco Chesini, Carole Dalin, Francisco Estrada, Marcelo Firpo Porto, Renata Gracie, Nelson Gouveia, Magali Hurtado-Díaz, Harry Kennard, Eliane Lima e Silva, Aline Martins de Carvalho, Zaray Miranda-Chacon, Nahid Mohajeri, Romulo Paes-Sousa, Chrissie Pantoja, Tim Repke, Luiza Ribeiro Alves Cunha, Antonella Risso, Matilde Rusticucci, Alejandro Saez Reale, Raquel Santiago, Mauricio Santos-Vega, Enzo Sauma, Sol Saliva, Milena Sergeeva, Cecilia Sorensen, Juan D Umaña, Armando Valdes-Velasquez, Maria Walawender, Juliana W. Rulli Villardi, Daniel Buss, Marina Romanello

Resumo executivo

A nível global, 2024 foi o ano mais quente já registrado, com uma temperatura média próxima à superfície de 1,55 °C acima dos níveis pré-industriais. Esse recorde faz parte de uma tendência contínua de aquecimento, com temperaturas que permaneceram acima do limite de 1,5 °C por quase dois anos. Esse aumento, impulsionado principalmente pelas atividades econômicas humanas, vem gerando riscos simultâneos em toda a América Latina — incluindo ondas de calor, incêndios florestais e inundações — que devastam comunidades, afetam meios de subsistência e provocam múltiplas consequências para a saúde das populações da região.

Em meio a essa ameaça crescente, a saúde humana vem se tornando um eixo central da diplomacia climática internacional. Durante a COP28, no Marco dos Emirados Árabes Unidos, foi incorporada uma meta específica em saúde e operacionalizado o Objetivo Global de Adaptação. Embora esses avanços sejam significativos, ainda persistem obstáculos substanciais. Mesmo após uma década desde o Acordo de Paris, o mundo continua longe de alcançar suas metas climáticas. A contínua dependência de combustíveis fósseis e o não cumprimento dos compromissos de redução de emissões e de financiamento climático estão entre os principais fatores que comprometem os esforços para proteger a saúde humana e avançar rumo a um futuro próspero.

O Relatório 2025 do The *Lancet* Countdown América Latina (LCLA) — uma colaboração entre 25 instituições acadêmicas regionais e agências das Nações Unidas — avalia 41 indicadores em 17 países latino-americanos, oferecendo evidências claras sobre os

crescentes impactos das mudanças climáticas na saúde humana. Nesta edição, foram introduzidas melhorias metodológicas, incluindo o aprimoramento de indicadores existentes e a incorporação de seis novos indicadores. Além disso, foi considerada uma perspectiva subnacional sempre que os dados permitiram, reconhecendo que os impactos climáticos e a eficácia das respostas variam significativamente entre e dentro dos países.

Enfrentar as mudanças climáticas é essencial para proteger a saúde humana, que continuará se deteriorando no futuro devido aos efeitos cumulativos das mudanças climáticas.

O relatório LCLA 2025 evidencia o aumento dos impactos sanitários das mudanças climáticas antropogênicas na América Latina, mostrando tendências alarmantes de intensificação dos perigos climáticos que colocam em risco as pessoas e a sociedade. As populações estão cada vez mais expostas ao calor, a eventos climáticos extremos e a um risco maior de transmissão de doenças infecciosas.

A temperatura média ambiental na América Latina vem apresentando uma tendência persistente de aquecimento desde o ano 2000. A exposição média anual aumentou de 23,3 °C entre 2001–2010 para 23,8 °C entre 2015–2024, atingindo um recorde de 24,3 °C em 2024. Esses aumentos não são homogêneos: foram observadas exposições térmicas mais elevadas em países como Bolívia (+2 °C), Venezuela (+1,7 °C), México (+1,6 °C), Paraguai (+1,5 °C), Equador (+1,4 °C), Guatemala (+1,3 °C), Brasil (+1,2 °C) e Colômbia (+1 °C), além de extremos térmicos ainda maiores dentro desses países. As consequências para a saúde são profundas: crianças com menos de um ano estiveram expostas a 4,5 vezes mais dias de ondas de calor, enquanto adultos acima de 65 anos enfrentaram uma exposição 10 vezes maior em comparação ao período base de 1981–2000. Na Venezuela e na Colômbia, esses aumentos foram especialmente altos, com exposições 51 e 59 vezes maiores, respectivamente. No geral, a mortalidade atribuída ao calor aumentou em 103%, com aproximadamente 13 mil mortes anuais, representando um custo monetário médio anual de US$ 855 milhões durante 2015–2024 (229% a mais que na década anterior). As perdas de produtividade relacionadas ao calor em 2024 somaram US$ 52 bilhões (12,6% a mais que em 2023), afetando desproporcionalmente os setores agrícola e da construção civil.

O aumento na frequência e na intensidade de eventos extremos, como secas e incêndios florestais, tem impactado a maioria dos países da região. A proporção de terras latino-americanas sob condições de seca meteorológica (maior ou igual a um mês) aumentou 275%, passando de 15,8% em 1981–1990 para 59,1% em 2015–2024, com Brasil, Bolívia e México entre os mais afetados. Essa tendência também é observada nas secas prolongadas: a proporção de terras com seca agrícola (maior ou igual a três meses) aumentou de 6,3% para 40,7%, e aquelas com seca hidrológica (maior ou igual a seis meses) de 2,1% para 20,8% no mesmo período. Isso provavelmente contribuiu para o risco extremo de incêndios florestais observado em 2024, com um aumento

de 10% na região (em 9 dos 17 países). Os maiores aumentos foram registrados no Chile (30,5 dias, +105%), México (17,6 dias, +28,5%) e Bolívia (16,7 dias, +82,6%).

Esses eventos extremos geraram perdas econômicas diretas próximas a US$ 19,2 bilhões em 2024 (0,3% do PIB regional). Infelizmente, menos de 5% dessas perdas estavam seguradas. O Brasil concentrou dois terços das perdas totais, seguido por México e Chile. Em relação ao PIB nacional, Chile e Brasil tiveram as maiores perdas proporcionais (ambos em torno de 0,63%), seguidos por México (0,14%), Panamá (0,13%), Equador (0,08%) e Peru (0,07%).

Vale destacar que muitos desses eventos climáticos extremos ocorrem de forma simultânea ou consecutiva (por exemplo, secas prolongadas, ondas de calor intensas e incêndios florestais). Enfrentar as mudanças climáticas é, portanto, essencial para proteger a saúde humana, evitando, riscos em cascata e choques econômicos que retardam a recuperação e enfraquecem a resiliência.

A adaptação deixou de ser opcional, é um requisito essencial e inegociável. Deve-se priorizar uma estratégia multinível que reduza os riscos climáticos, aumente a resiliência e enfrente as desigualdades socioeconômicas existentes.

As ações a nível governamental e de políticas nacionais continuam insuficientes para enfrentar as mudanças climáticas, evidenciando uma falha sistêmica em priorizar a resiliência em saúde. O Relatório LCLA 2025 mostra que os esforços de planejamento são limitados: menos da metade dos países da região (41,2%) declarou publicamente ter concluído uma Avaliação de Vulnerabilidade e Adaptação desde 2020, e apenas nove países (53%) desenvolveram um Plano Nacional de Adaptação em Saúde. Além disso, a integração do componente de saúde nas Contribuições Nacionalmente Determinadas (NDC) ainda é insuficiente.

Essa falta de impulso político também se reflete nos fóruns internacionais e no financiamento climático. Embora as NDC incluam cada vez mais considerações sobre equidade, sua visibilidade na Assembleia Geral das Nações Unidas (AGNU) diminuiu significativamente: as menções à saúde nos discursos de países latino-americanos caíram de um máximo de 10 em 2010 para apenas três países (Bolívia, Brasil e Chile) em 2024, enfraquecendo a presença regional dos vínculos entre clima, saúde e justiça ambiental no cenário global.

Superar essa falta de priorização requer investimento direcionado ao planejamento e à implementação da adaptação em saúde. Os doadores bilaterais comprometeram US$ 197 milhões para projetos de adaptação em saúde em 2024, mas 68% desse valor foram destinados exclusivamente ao Brasil. Dos US$ 3,4 bilhões aprovados pelo Fundo Verde para o Clima desde 2017 para projetos com componente de saúde, apenas US$ 77,7 milhões (2,3%) foram alocados diretamente à adaptação em saúde.

O fortalecimento dos sistemas de saúde exige dados robustos e colaboração

interinstitucional. Embora 10 dos 17 membros latino-americanos da Organização Meteorológica Mundial informem oferecer serviços climáticos para a saúde, esses se concentram principalmente em monitoramento e dados imediatos, com pouca atenção às projeções climáticas de longo prazo — fundamentais para o planejamento estratégico. Uma estrutura institucional sólida é um pilar essencial para sistemas de saúde resilientes, e funciona: países com sistemas de alerta precoce baseados em informações climáticas, *Health Early Warning Systems* (HEWS, siglas em inglês) registraram uma redução de 92,5% na mortalidade por inundações e tempestades. No entanto, a preparação para emergências, conforme autorrelatada, vem diminuindo desde 2022 — um problema crítico para países vulneráveis a doenças como a dengue, tais como Bolívia, Brasil e Peru.

Ao mesmo tempo, a limitada capacidade de recursos humanos e o baixo financiamento para a geração de conhecimento também enfraquecem a resposta regional. Apenas 17% dos estudantes de saúde pública recebem formação sobre mudanças climáticas, o que restringe a preparação da força de trabalho. A produção de conhecimento também é limitada. Apesar do aumento das publicações científicas sobre clima e saúde desde 2015, a América Latina representa apenas 5,5% da produção mundial. Além disso, grande parte das pesquisas ignora questões de equidade, refletindo as mesmas lacunas no financiamento de projetos que abordam explicitamente a interseção entre clima e saúde. Sem evidências locais relevantes, torna-se difícil posicionar as necessidades regionais nas agendas internacionais.

A nível local e comunitário, a implementação de estratégias de adaptação continua limitada e pouco estratégica. A deficiência do planejamento urbano é evidente: todas as cidades latino-americanas com mais de 500 mil habitantes foram classificadas com níveis baixos ou muito baixos de áreas verdes, perdendo uma oportunidade essencial de fortalecer a resiliência urbana por meio da infraestrutura natural. Das unidades administrativas locais pesquisadas, apenas 54 reconheceram problemas de saúde vinculados a ameaças climáticas, concentrando-se principalmente em inundações, tempestades e chuvas intensas, seguidas por secas e temperaturas extremas.

Por outro lado, a integração da agenda climática com os Objetivos de Desenvolvimento Sustentável (ODS) constitui uma base essencial para a resiliência. Melhorias nos serviços básicos de água e saneamento, por exemplo, impulsionaram uma redução de cerca de 60% no Índice de Risco de Mosquitos desde 2000.

A nível individual, a proteção frequentemente depende do “*status*” econômico. O crescente uso do ar-condicionado representa um claro dilema entre mitigação e adaptação: seu alto consumo de energia aumenta as emissões de GEE quando não vinculado a fontes limpas. Sua baixa adoção (27% dos domicílios) reflete desigualdades significativas em quem pode se proteger do calor extremo. Essa disparidade se repete na poluição do ar em ambientes internos, muito mais alta em áreas rurais e em lares de baixa renda.

O engajamento midiático e social reflete a atual polarização política. Observa-se um sinal misto, com risco de desinformação e uma diminuição geral na cobertura sobre a relação entre clima e saúde. Embora a cobertura sobre saúde tenha aumentado modestamente na mídia e nas redes sociais (onde o interesse cresceu substancialmente desde 2017), a cobertura geral sobre mudanças climáticas diminuiu em 2024, provavelmente devido ao deslocamento do foco para temas mais politizados.

A governança climática e sanitária eficaz deve ser definida por avanços tangíveis. Os governos precisam tomar decisões que gerem ação, responsabilização e impacto real diante das mudanças climáticas e da saúde.

Em toda a América Latina, o financiamento, o apoio à transição energética justa e a ação climática coletiva continuam criticamente baixos. Um fator central por trás dessa estagnação é a persistente dependência dos combustíveis fósseis. Os países latino-americanos apresentam um preço líquido negativo de carbono, com subsídios a combustíveis fósseis equivalentes a US$ 38,6 bilhões — quase 50 vezes superiores às receitas geradas pela precificação do carbono.

Construir um futuro resiliente exige transformar fundamentalmente os sistemas energéticos e reduzir a dependência dos combustíveis fósseis, o que requer compromissos mais ambiciosos de mitigação refletidos explicitamente nas NDC.

Essa transformação deve incluir o uso de energia nos lares e a adoção de transportes sustentáveis e saudáveis — setores-chave por sua relação direta com as emissões de GEE e a saúde. Além disso, a forma como produzimos e consumimos alimentos, e como gerimos nossas florestas, tem um impacto crucial em nossa capacidade de prosperar diante dessa crise.

Embora substituir o carvão e outros combustíveis fósseis por energias renováveis seja essencial para um futuro saudável e sustentável, o Relatório LCLA 2025 mostra avanços desiguais entre países e setores. A nível regional, a participação de fontes de baixa emissão de carbono na geração elétrica caiu de 67,6% para 58,9%, enquanto as energias renováveis (solar e eólica) cresceram de 2,7% para 11,8%, superando o carvão em 2014. No entanto, a geração elétrica a partir do carvão quase dobrou (de 2,6% para 5,2%), com um aumento após a pandemia. Os combustíveis fósseis continuam dominando o transporte rodoviário, representando 96,7% do setor.

A falta de enfrentamento dessas fontes de emissão mantém elevados níveis de exposição à poluição por material particulado fino - PM2,5 . A concentração média nacional de PM2,5 em ambientes internos, proveniente da queima de combustíveis sólidos poluentes para cozinhar e aquecer, foi estimada em 245 μg/m^3 em 2022 — mais que o dobro nas residências rurais (314 μg/m^3) em comparação às urbanas (145 μg/m^3) —, evidenciando profundas desigualdades socioeconômicas. Essa disparidade deve-se ao acesso limitado a combustíveis limpos e ao uso persistente de biomassa (31% nas áreas rurais versus 5% nas urbanas). Além disso, 79% dos lares latino-americanos

utilizam gás liquefeito de petróleo (GLP) para cozinhar, o que representa uma oportunidade perdida de transição direta para fontes renováveis e ressalta os desafios futuros de uma região ainda fortemente dependente dos combustíveis fósseis.

As emissões fósseis de PM2,5 (carvão e gás) provenientes de fontes ambientais causam cerca de 360 mil mortes prematuras entre pessoas em idade ativa, impondo um pesado ônus social e econômico. As mortes prematuras atribuíveis à exposição à PM2,5 por biomassa foram estimadas em 140 mil durante 2018–2022 — um aumento de 17 mil em relação a 2007–2011. Os custos monetizados dos Anos de Vida Perdidos atribuíveis à PM2,5 em 11 países latino-americanos chegaram a US$ 160 bilhões, o equivalente a 2,8% do PIB agregado e à renda anual média de cerca de 15,8 milhões de pessoas na região.

Outras fontes regionais de emissões — como a agricultura e a perda de cobertura florestal impulsionada pela produção de commodities e pelo desmatamento — já transformaram as zonas orientais da Amazônia em uma fonte líquida de carbono. Estratégias de mitigação como a agricultura regenerativa, a pecuária sustentável e a agroecologia oferecem soluções ao evitar a expansão, restaurar solos e conservar a biodiversidade essencial para manter a saúde humana. Da mesma forma, a transformação do sistema alimentar representa uma estratégia de mitigação fundamental, com co-benefícios imediatos para a saúde e o meio ambiente, garantindo acesso equitativo a alimentos sustentáveis, promovendo a biodiversidade e fortalecendo a segurança alimentar.

A América Latina não tem o luxo de esperar uma maior vontade política global; deve avançar com ações nacionais que protejam as pessoas e a natureza. É hora de os países cumprirem suas NDC e Planos Nacionais de Adaptação por meio de uma governança eficaz — definida pela ação, pela responsabilização e por impactos mensuráveis na saúde —, e não apenas por promessas esperançosas. Isso é especialmente crucial diante das tensões geopolíticas e das mudanças nas prioridades dos doadores, que ameaçam atrasar a concretização dos recursos prometidos no ritmo necessário para proteger a saúde pública.

À medida que se aproxima a COP30 em Belém do Pará, a região tem uma oportunidade única de liderar iniciativas de adaptação climática equitativas e centradas na saúde, além de impulsionar estratégias rápidas de mitigação, com co-benefícios sanitários e justiça para todos.
